# CHD1 loss negatively influences metastasis-free survival in R0-resected prostate cancer patients and promotes spontaneous metastasis in vivo

**DOI:** 10.1038/s41417-020-00288-z

**Published:** 2021-01-07

**Authors:** Su Jung Oh-Hohenhorst, Derya Tilki, Ann-Kristin Ahlers, Anna Suling, Oliver Hahn, Pierre Tennstedt, Christiane Matuszcak, Hanna Maar, Vera Labitzky, Sandra Hanika, Sarah Starzonek, Simon Baumgart, Steven A. Johnsen, Martina Kluth, Hüseyin Sirma, Ronald Simon, Guido Sauter, Hartwig Huland, Udo Schumacher, Tobias Lange

**Affiliations:** 1 Institute of Anatomy and Experimental Morphology, University Medical Center Hamburg-Eppendorf, Martinistrasse 52, 20246 Hamburg, Germany; 2 Martini-Klinik, Prostate Cancer Center, University Medical Center Hamburg-Eppendorf, Hamburg, Germany; 3 Department of Medical Biometry and Epidemiology, University Medical Center Hamburg-Eppendorf, Hamburg, Germany; 4 Department of Urology, University Medical Center Goettingen, Göttingen, Germany; 5 Department of General, Visceral and Pediatric Surgery, University Medical Center Göttingen, Robert-Koch-Strasse 40, 37075 Goettingen, Germany; 6 Gene Regulatory Mechanisms and Molecular Epigenetics Lab, Gastroenterology Research, Mayo Clinic, 200 First Street SW, Rochester, MN 55905 USA; 7 Institute of Pathology, University Medical Center Hamburg-Eppendorf, Hamburg, Germany; 8 Present Address: Research & Development, Pharmaceuticals, Bayer AG, Müllerstr. 178, 13353 Berlin, Germany

**Keywords:** Prostate cancer, Metastasis

## Abstract

The outcome of prostate cancer (PCa) patients is highly variable and depends on whether or not distant metastases occur. Multiple chromosomal deletions have been linked to early tumor marker PSA recurrence (biochemical relapse, BCR) after radical prostatectomy (RP), but their potential role for distant metastasis formation is largely unknown. Here, we specifically analyzed whether deletion of the tumor suppressor CHD1 (5q21) influences the post-surgical risk of distant metastasis and whether CHD1 loss directly contributes to metastasis formation in vivo. By considering >6800 patients we found that the CHD1 deletion negatively influences metastasis-free survival in R0 patients (HR: 2.32; 95% CI: 1.61, 3.33; *p* < 0.001) independent of preoperative PSA, pT stage, pN status, Gleason Score, and BCR. Moreover, CHD1 deletion predicts shortened BCR-free survival in pT2 patients and cancer-specific survival in all patients. In vivo, CHD1 loss increases spontaneous pulmonary metastasis formation in two distinct PCa models coupled with a higher number of multicellular colonies as compared to single-cell metastases. Transcriptome analyses revealed down-regulation of the PCa-specific metastasis suppressor and TGFβ signaling regulator PMEPA1 after CHD1 depletion in both tested PCa models. CHD1 loss increases the risk of postoperative metastasis in R0-resected PCa patients and promotes spontaneous metastasis formation in vivo.

## Introduction

The clinical behavior of prostate cancer (PCa) ranges from slowly growing indolent tumors to highly aggressive metastatic disease and accordingly leads to variable clinical outcomes for patients. While clinical factors, such as Gleason Score and prostate-specific antigen (PSA) levels, have proven useful for risk stratification and in guiding treatment decisions about PCa, significant clinical heterogeneity remains [1]. Thus, the identification and evaluation of new molecular markers that could be integrated with established clinical factors to improve the prognostication and estimation of individualized risk for metastatic progression are of major importance in current PCa research [2]. In this regard, recent research interest has been directed towards the collaborative nature of multiple genomic alterations underlying the critical process of PCa progression [3, 4]. Among others, chromosomal deletions resulting in the dysregulation of chromatin remodeling and repair of DNA damage have been suggested to negatively influence the prognosis of PCa patients [5, 6]. Large cohort analyses have identified concurrent deletions and/or genomic re-arrangements that cooperatively drive PCa progression and provide individualized prognostic potential for patients [7, 8].

Deletions in chromosome 5q21 are among the most frequent (~10%) chromosomal deletions in PCa and include the gene for chromodomain-helicase-DNA-binding protein-1 (CHD1). CHD1 acts as a chromatin remodeling protein that directs lineage-specific transcription and keeps DNA regulatory regions in an open and transcriptionally active state [9, 10]. In particular, CHD1 is critical for double-strand break (DSB) repair via homologous recombination, so decreased CHD1 expression leads to genomic instability and hence tumor progression [11, 12].

From a clinical perspective, the loss of CHD1 has been found to correlate with higher Gleason grade, tumor stage, and postoperative biochemical relapse (BCR) in a cohort of more than 2000 PCa patients and suggested as a predictor for poor prognosis [6]. Recent studies have also demonstrated that targeted depletion of CHD1 in PCa cells leads to a defect in the DSB repair pathway and thereby resulted in increased response to DNA-damaging therapies, such as ionizing radiation and PARP and PTEN inhibition [12, 13]. Similarly, increased sensitivity of CHD1-deleted PCa with SPOP mutation to the novel antiandrogen abiraterone has been reported [14]. Such observations indicate not only the prognostic role of CHD1 deletion but also the therapeutic potential of such a “genetic handicap” and thereby support the rationale for therapeutic targeting of DNA-repair defects in CHD1-deleted PCa.

Although considerable interest in the prognostic and therapeutic potential of CHD1 deletion in PCa is currently growing, the potential implications of CHD1 deletion for distant metastasis formation that are responsible for cancer-specific death in PCa patients remain to be determined.

In this study, we investigated an expanded cohort of more than 6800 PCa patients who underwent radical prostatectomy (RP) to determine by multivariate analyses the potential influence of the CHD1 deletion on the postoperative metastasis-free survival (MFS), BCR-free survival, and cancer-specific survival (CSS). To assess the potential functional role of CHD1 for metastasis, we investigated whether CHD1 loss promotes distant metastasis formation in vivo by using xenograft mouse models that reflect the entire metastatic cascade and develop spontaneous micro-metastases in the lungs of immunodeficient mice. Finally, RNA sequencing was performed to identify at the transcriptome level novel candidates that may explain the effect of CHD1 loss on PCa metastasis.

## Results

### Predictive value of CHD1 deletion for oncological outcome after RP

For the present study, fluorescence in situ hybridization (FISH) data for CHD1 were available from 7902 patients who underwent RP at our institution (incidence: 9.9%, homozygous: 2.5%, heterozygous 7.4%). By combining the FISH dataset with the clinical outcome database we determined the predictive value of the CHD1 deletion for the oncological outcome after RP. Patients with (neo)adjuvant androgen deprivation therapy (*n* = 502), implausible values (*n* = 57), and incomplete follow-up data (*n* = 512) were excluded resulting in 6831 patients available for adjusted survival analyses. Baseline characteristics of the study population are summarized in Table 1. Median follow-up after surgery was 6 years. Mean time from surgery to BCR was 36.6 months and mean time from BCR to metastasis was 41.4 months. Mean time from metastasis to cancer-specific death was 26 months. At last follow-up, 1725 of 7343 patients had developed a BCR (23.5%). In addition, 261 patients had been diagnosed with distant metastases (3.6%) and 128 patients had suffered from confirmed cancer-specific death (1.7%). In the subset of patients with CHD1 deletion, the incidence of BCR, metastasis, and cancer-specific death increased to 31.3%, 7.8%, and 3.1%, respectively. These descriptive associations are visualized by the cumulative incidence curves in Suppl. Fig. S1A–C, which consider patients until their respective event or time-point of censoring and with complete datasets in adjusting variables only (final cohort of 6831 patients).

**Table 1:** Baseline demographic and clinical characteristics of patients by CHD1 status.

	CHD1		
	Normal (*N* = 6608)	Deletion (*N* = 735)	Total (*N* = 7343)
*Patient age [years]*			
Mean ± SD	63.2 ± 6.3	64.6 ± 5.7	63.4 ± 6.3
Median [IQR]	63.9 [59.1; 68.0]	65.2 [61.0; 68.7]	64.0 [59.3; 68.0]
Range	37.1; 80.8	39.0; 77.1	37.1; 80.8
Missing	8/6608 (0.1%)	2/735 (0.3%)	10/7343 (0.1%)
*Follow-up [years]*			
Median^a^ [95% CI]	6.0 [5.8; 6.0]	5.6 [5.1; 6.0]	6.0 [5.8; 6.0]
Missing	412/6608 (6.2%)	42/735 (5.7%)	454/7343 (6.2%)
*Preoperative PSA [ng/mL]*			
Mean ± SD	9.2 ± 9.3	9.5 ± 8.8	9.2 ± 9.2
Median [IQR]	6.8 [4.8; 10.3]	7.4 [5.2; 11.0]	6.8 [4.9; 10.4]
Range	0.5; 192.0	0.6; 125.0	0.5; 192.0
Missing	48/6608 (0.7%)	8/735 (1.1%)	56/7343 (0.8%)
*pT stage*			
pT2	4381/6602 (66.4%)	444/734 (60.5%)	4825/7336 (65.8%)
pT3a	1470/6602 (22.3%)	186/734 (25.3%)	1656/7336 (22.6%)
pT3b/pT4	751/6602 (11.4%)	104/734 (14.2%)	855/7336 (11.7%)
Missing	6/6608 (0.1%)	1/735 (0.1%)	7/7343 (0.1%)
*Gleason Score*			
≤3 + 3	1649/6600 (25.0%)	94/734 (12.8%)	1743/7334 (23.8%)
3 + 4	3818/6600 (57.8%)	387/734 (52.7%)	4205/7334 (57.3%)
4 + 3	894/6600 (13.5%)	205/734 (27.9%)	1099/7334 (15%)
≥4 + 4	239/6600 (3.6%)	48/734 (6.5%)	287/7334 (3.9%)
Missing	8/6608 (0.1%)	1/735 (0.1%)	9/7343 (0.1%)
*pN stage*			
N0	3690/6608 (55.8%)	484/735 (65.9%)	4174/7343 (56.8%)
Nx	2614/6608 (39.6%)	217/735 (29.5%)	2831/7343 (38.6%)
N+	304/6608 (4.6%)	34/735 (4.6%)	338/7343 (4.6%)
*Surgical margin status*			
Positive	1298/6602 (19.7%)	143/733 (19.5%)	1441/7335 (19.6%)
Negative	5304 (80.2%)	590/733 (80.2%)	5894/7335 (80.3%)
Missing	6/6608 (0.1%)	2/735 (0.3%)	8/7343 (0.1%)
Biochemical recurrence (Yes)	1495 (22.6%)	230 (31.3%)	1725 (23.5%)
Metastasis (Yes)	204 (3.1%)	57 (7.8%)	261 (3.6%)
Cancer-specific death (Yes)	105 (1.6%)	23 (3.1%)	128 (1.7%)

^a^Estimated by the reverse-Kaplan–Meier method.

Cause-specific cox proportional hazards models were used to test the predictive value of CHD1 deletion for oncological outcome as summarized in Tables 2–4. The statistical models also tested potential two-way interactions between the CHD1 deletion status and further adjusting parameters. By this we revealed significant interactions of the CHD1 status with pT stage (BCR-free survival; Table 2) and of the CHD1 status with R status (MFS, Table 3). The CHD1 deletion predicted early BCR in patients with pT2 tumors (independent of all other adjusting variables, hazard ratios (HR) 1.54 [1.23,1.93], *p* < 0.001; Table 2) and shortened MFS in R0 patients (independent of all other adjusting variables, HR 2.32 [1.61, 3.33], *p* < 0.001; Table 3). The influence of the CHD1 deletion on CSS was independent of other variables (HR 1.66 [1.03, 2.68], *p* = 0.037; Table 4).

**Table 2:** Predictive value of CHD1 deletion for BCR-free survival (multivariate cause-specific Cox proportional hazards model, *n* = 6831, events = 1705).

	HR	95% CI	*P* value
pT Stage*CHD1			0.011
pT2: CHD1 (del. vs norm.)	1.54	[1.23, 1.93]	<0.001
pT3a: CHD1 (del. vs norm.)	0.93	[0.71, 1.20]	0.564
pT3b/pT4: CHD1 (del. vs norm.)	1.12	[0.87, 1.44]	0.380
Age [per 10 years]	0.99	[0.91, 1.07]	0.741
Surgical margin (pos. vs neg.)	1.60	[1.44, 1.78]	<0.001
Gleason group			<0.001
3 + 4 vs ≤3 + 3	2.15	[1.80, 2.57]	<0.001
4 + 3 vs ≤3 + 3	4.26	[3.47, 5.24]	<0.001
≥4 + 4 vs ≤3 + 3	5.59	[4.34, 7.21]	<0.001
pN status			<0.001
Nx vs N0	0.76	[0.67, 0.87]	<0.001
N+vs N0	1.66	[1.41, 1.95]	<0.001
PSA [per 10 units]	1.13	[1.09, 1.16]	<0.001

**Table 3:** Predictive value of CHD1 deletion for metastasis-free survival (multivariate cause-specific Cox proportional hazards model, *n* = 6831, events = 259).

	HR	95% CI	*P* value
Surgical margin*CHD1			0.030
Negative: CHD1 (del. vs norm.)	2.32	[1.61, 3.33]	<0.001
Positive: CHD1 (del. vs norm.)	1.04	[0.55, 1.95]	0.907
Age [per 10 years]	0.80	[0.65, 0.99]	0.040
pN status			0.060
Nx vs N0	0.72	[0.47, 1.11]	0.138
N + vs N0	1.34	[0.96, 1.87]	0.085
PSA [per 10 units]	1.00	[0.92, 1.08]	0.916
pT stage			0.061
pT3a vs pT2	0.98	[0.68, 1.41]	0.898
pT3b/pT4 vs pT2	1.37	[0.95, 1.98]	0.096
Gleason group			<0.001
3 + 4 vs ≤3 + 3	3.85	[1.54, 9.61]	0.004
4 + 3 vs ≤3 + 3	8.72	[3.42, 22.24]	<0.001
≥4 + 4 vs ≤3 + 3	14.01	[5.27, 37.22]	<0.001
Surgical margin*BCR			0.006
Negative: BCR (yes vs no)	264.27	[36.77, 1899.41]	<0.001
Positive: BCR (yes vs no)	12.85	[5.2, 31.77]	<0.001

**Table 4:** Predictive value of CHD1 deletion for cancer-specific survival (multivariate cause-specific cox proportional hazards model, *n* = 6831, events = 124).

	HR	95% CI	*P* value
CHD1 (del. vs norm.)	1.66	[1.03, 2.68]	0.037
Age [per 10 years]	0.80	[0.59, 1.08]	0.149
BCR (yes vs no)	4.40	[2.42, 8.03]	<0.001
Gleason Group			<0.001
3 + 4 vs ≤3 + 3	2.00	[0.85, 4.70]	0.111
4 + 3 vs ≤3 + 3	5.09	[2.05, 12.65]	<0.001
≥4 + 4 vs ≤3 + 3	17.86	[6.80, 46.85]	<0.001
pN status			0.364
Nx vs N0	1.01	[0.57, 1.80]	0.969
N+ vs N0	1.41	[0.88, 2.27]	0.156
PSA [per 10 units]	1.06	[0.97, 1.15]	0.209
Surgical margin (pos vs neg)	1.78	[1.22, 2.59]	0.003
pT stage			<0.001
pT3a vs pT2	0.83	[0.44, 1.57]	0.575
pT3b/pT4 vs pT2	2.17	[1.20, 3.93]	0.010
			

### Depletion of CHD1 promotes spontaneous pulmonary metastasis in PCa xenografts

**Fig. 1:**
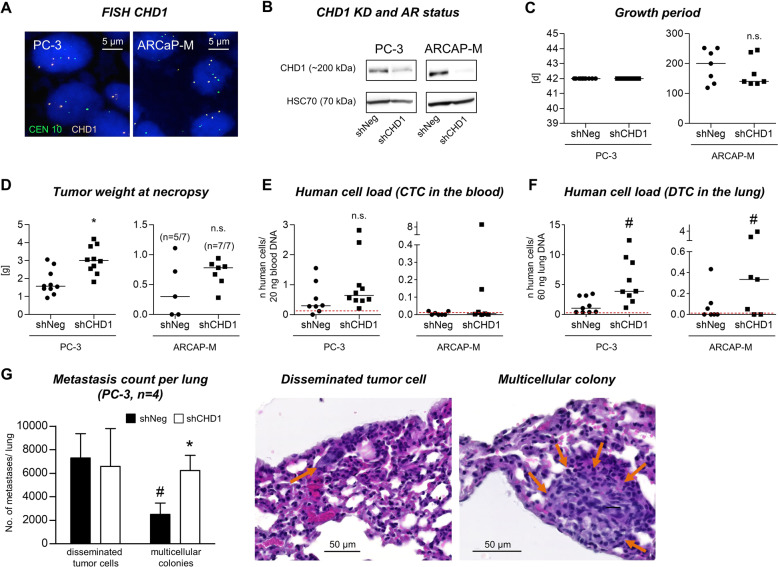
Functional role of CHD1 loss for spontaneous metastasis formation in vivo. The functional consequences of CHD1 depletion were studied in ARCAP-M (AR+, PTEN-wt) and PC-3 (AR−, PTEN−/−) xenografts, which show spontaneous lung metastasis formation in immunodeficient mice. CHD1 copy number and CHD1-KD status are shown in **A** and **B**, respectively (normal CHD1 copy number in both ARCAP-M and PC-3). S.c. xenograft primary tumor growth periods and tumor weights at necropsy are shown in **C** and **D**, respectively. While the human cell load in the blood was insignificantly elevated upon CHD1-KD (**E**), the number of metastatic cells in the lungs was significantly increased after CHD1 depletion in both models (**F**). Based on histology, CHD1 depletion mainly improved metastatic outgrowth in terms of more frequent multicellular colonies (**G**). Orange arrows in the H&E samples indicate representative samples of disseminated tumor cells (DTC, left picture) and metastatic colonies (right picture). The red dashed lines in **E** and **F** represent the detection threshold for human DNA in the respective Alu-PCR experiment. ^#^*p* = 0.004 vs shNeg considering both models (**F**); *p < 0.05 vs shNeg [multicellular colonies] (**G**).

Based on our clinical analyses, we next determined the functional role of CHD1 depletion for metastasis formation in vivo using ARCAP-M as well as PC-3 xenografts (Fig. 1). ARCAP-M represents an AR-positive, PTEN-wildtype PCa model while PC-3 represents an AR-negative, PTEN-deleted PCa model (Suppl. Fig. S2A, B). Xenograft tumors derived from both cell lines showed normal copy numbers of CHD1 as determined by FISH (Fig. 1a). After lentiviral transduction of either shRNA targeting CHD1 (shCHD1) or a non-targeting control shRNA (shNeg) into ARCAP-M and PC-3 cells, stable knockdown (KD) of CHD1 was confirmed by western blot (WB, Fig. 1b). Control and CHD1-KD cells were subcutaneously injected into immunodeficient mice and potential effects of the CHD1-KD on xenograft primary tumor growth and spontaneous metastatic spread were analyzed. After comparable growth periods (Fig. 1c), the CHD1-KD increased the tumor take rate of ARCAP-M xenografts (five of seven mice in the shNeg group and seven of seven mice in the shCHD1 group developed tumors) and caused significantly higher primary tumor weights in PC-3 xenografts (Fig. 1d). The number of circulating tumor cells (CTC) in the mouse blood was slightly, but insignificantly increased after CHD1-KD in both xenograft models (Fig. 1e). The human cell load in the mouse lungs as determined by Alu-PCR was significantly increased upon CHD1-KD in both models (mean values [shNeg vs shCHD1] for PC-3: 1.54 vs 5.62; for ARCAP-M: 0.12 vs 1.11; *p* = 0.004, Fig. 1f). Morphological analyses of the mouse lungs demonstrated an increased number of multicellular colonies in the CHD1-KD (*p* = 0.016) while control xenografts mainly developed single-cell metastases (Fig. 1g, *n* = 4 per group, 10 lung sections per mouse). Further samples of single disseminated tumor cells (DTC) and multicellular colonies in the lungs are shown in Suppl. Fig. S3.

### Metastasis suppressor PMEPA1 is down-regulated upon CHD1-KD in PCa tumoroids

**Fig. 2:**
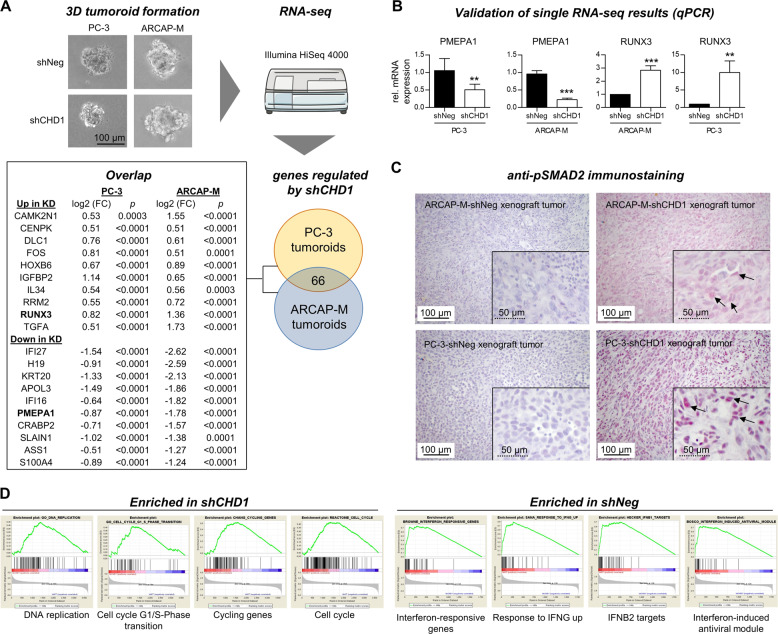
Coding transcriptome analyses of prostate cancer tumoroids. Control and CHD1-KD ARCAP-M and PC-3 cells were cultivated under 3D conditions and RNA was isolated from established tumoroids. RNA sequencing was performed and biostatistics analysis was focused on genes that were regulated in both models. The top 10 up- or down-regulated candidates are depicted in **A**. See Supplementary Excel files for all genes identified in the overlap and for full lists of genes regulated either in the PC-3 or ARCAP-M model. Note the down-regulation of PMEPA1 and up-regulation of RUNX3 after CHD1-KD in both models (validated by qPCR) (**B**). Accordingly, pSMAD2 levels are enhanced upon CHD1-KD in xenograft primary tumors of both models (**C**). Gene set enrichment analyses demonstrate up-regulation of cell cycle-promoting genes in the CHD1-KD while interferon response genes are enriched in the control (**D**). **p < 0.001; ***p < 0.0001.

In order to elucidate the underlying molecular effects eliciting the phenotype of increased spontaneous lung metastasis in vivo, which was common among both tested models after CHD1-KD, ARCAP-M and PC-3 control and CHD1-KD samples were analyzed by RNA-seq. As illustrated in Fig. 2. only tumoroid spheres (derived from 3D culture of PC-3 and ARCAP-M cells), but not xenograft tissues were used for this purpose because of a high proportion of tumor necrosis in the ARCAP-M xenografts; therefore, the total RNA extracted from ARCAP-M xenograft tumor tissues was frequently degraded and useless for RNA-seq. Tumor cells from conventional (2D) cell culture were also not used for RNA-seq since the pro-metastatic effect of the CHD1-KD observed in vivo was not reflected in vitro. By specifically examining the overlap in gene regulation achieved by CHD1-KD, we identified 66 genes concordantly regulated in both models (see Fig. 2a for top 10 and Supplementary Excel Files for full lists of overlapping and unique genes). Most interestingly, the gene of prostate transmembrane protein androgen induced 1 (PMEPA1), a known suppressor of metastasis and negative regulator of transforming growth factor-β (TGF-β) signaling in PCa, was down-regulated after CHD1-KD in both models (Fig. 2a), which could be validated by qPCR (Fig. 2b). TGF-β target genes such as RUNX3 were up-regulated after CHD1-KD (Fig. 2a, b). pSMAD levels were increased upon CHD1-KD in xenograft primary tumors (Fig. 2c). Based on gene set enrichment analyses (GSEAs) we identified several cell cycle-related genes enriched in the CHD1-KD tumoroids while interferon targets were enriched in the control tumoroids (Fig. 2d). Enlarged versions of the GSEA data can be found in Suppl. Fig. S4.

### In vitro characterization of CHD1-depleted PCa cells

In sharp contrast to our in vivo findings, in vitro assays for tumor cell proliferation and metastatic properties rather indicated less metastatic potential of CHD1-KD PCa cells. In particular, there was no difference in cell proliferation under conventional cell culture conditions (2D) (Suppl. Fig. S2C), but less (ARCAP-M) or smaller (PC-3) colonies formed in 3D soft agar assays (Suppl. Fig. S2D). While the migratory behavior did not change (Suppl. Fig. S2E), we observed decreased invasiveness upon CHD1-KD (Suppl. Fig. S2F).

## Discussion

The present study confirms and considerably expands a previous publication from our group showing a correlation of CHD1 deletion with unfavorable tumor phenotype and early BCR [6]. In a first step, we investigated potential two-way interactions between CHD1 deletion and the adjusting variables. By this we revealed that the influence of the CHD1 deletion on BCR-free survival depends on the patient’s pT stage and is statistically significant in patients with pT2 tumors only, which comprise ~66% of the total cohort (independent of all other adjusting variables). This restriction might be explainable by a higher percentage of patients in the pT3/4 subsets who receive adjuvant radiation therapy (RT)—these have not been excluded from this study. Importantly, CHD1 loss sensitizes PCa cells to DNA-damaging therapy such as RT [12, 13]. Therefore, the influence of the CHD1 deletion on the BCR-free survival might get lost in the pT3/4 subset due to a higher fraction of irradiated patients, who specifically benefit from RT when CHD1 is deleted (preventing them from developing a BCR). The influence of the CHD1 deletion on MFS depends on the patient’s resection margin and is present in R0 patients only, which comprise ~80% of all patients (independent of all other adjusting variables). The influence of the CHD1 deletion on CSS is independent of any other variable, but significant two-way interactions might have been missed due to the low number of positive events.

The outcome of PCa patients is normally determined by the BCR-free survival [5–7, 15] as PSA recurrence can be determined in a quite standardized fashion (reference values and monitoring intervals are well-defined). Especially in R0 patients, PSA recurrence is an early and convenient indicator of disease progression, but on its own insufficient to predict lethal disease [16, 17]. Of note, despite only 259 cases with metastasis, CHD1 deletion was also statistically linked to MFS in our set of data; likewise, CHD1 deletion was linked to CSS despite only 124 cases with confirmed cancer-specific death. Although MFS would be the preferable endpoint in theory, this parameter suffers from variable motivation of attending physicians to positively identify metastases in case of elevated PSA. Moreover, there is a limitation in the currently used diagnostic tools, i.e. “occult” metastases might be present (as indicated by BCR), but too small to be detected by routine diagnostic procedures. This might change in the future with the increasing use of PSMA-PET/CT [18]. Furthermore, the metastasis-intrinsic capacity to grow out to a clinically detectable size differs individually.

In the present study, the event of BCR as well as higher Gleason Scores increase the risk of metastasis and cancer-specific death indicating that the used database is robust. Interestingly, a positive lymph node status (N+) alone has no significant influence on MFS or CSS, which might explain why the percentage of N+ patients is not increased in the CHD1-deleted subset. These findings suggest that lymph node and distant metastasis formation are not necessarily related to each other, which is supported by the literature [19]. In silico analyses of an independent cohort demonstrate an increase in CHD1 deletions (20% instead of 10%), when mainly metastasis samples from lethal, castration-resistant PCa are considered [20]. Nevertheless, we challenged our clinical findings and investigated whether CHD1 loss has functional consequences for metastasis formation in vivo.

Interestingly, the number of pulmonary metastases was significantly increased after CHD1-KD in two spontaneous metastasis PCa xenograft models, resembling the predictive value of CHD1 deletion for poor oncological outcome in the clinical setting. The PC-3 model represents AR-negative, PTEN-deleted PCa while ARCAP-M represents AR-positive, PTEN-wildtype PCa. Therefore, the increase in metastasis upon CHD1 loss was observed irrespective of the AR and PTEN status. Further studies are required to determine whether the prognostic role of CHD1 loss is independent of the AR signaling activity and PTEN status in patients since recent publications indicate a close relationship between CHD1 and AR, CHD1 and resistance to AR-targeted therapy as well as CHD1 and PTEN [21–24]. In a parallel study including 4986 patients with known CHD1 and PTEN status we determined the prognostic role of CHD1 loss in PTEN-wildtype vs -deleted patient subsets (Oh-Hohenhorst et al., in preparation).

Histological examinations of the lungs revealed a change in the morphology of spontaneous metastasis where, consistent with previous studies [25], the predominant phenotype of lung metastases was DTC in the control animals. In contrast, an increased number of multicellular lung metastases was observed in the CHD1-KD group. This finding implies that CHD1 suppresses metastatic outgrowth. Metastasis to other sites such as bone marrow can only rarely be found at a very low level in spontaneous metastasis xenografts and requires several modifications of the methodology [26]. As of yet, potential effects of the CHD1 loss on bone metastasis were therefore not determined, which is a limitation of this study. As an alternative, intracardiac injection models could have been used, but such models circumvent early steps of the metastatic cascade. For this reason, subcutaneous xenograft models that metastasize spontaneously to distant sites were used herein. In addition, our in vitro assays revealed a contrary phenotype of the tumor cells in vitro (decreased invasiveness and colony-forming capacity upon CHD1-KD as reported previously [6]). Therefore, in an intracardiac model the injected tumor cells would not represent the phenotype of tumor cells that spontaneously metastasize in the performed xenograft models. This discrepancy might be due to several differences between in vitro-cultivated and in vivo-grown tumor cells. For instance, it is known that remarkable epigenetic, transcriptomic, and glycosylation changes occur simply when tumor cells are taken from conventional 2D to more physiologic 3D culture [27–29].

Transcriptome analyses of 3D tumoroids revealed down-regulation of PMEPA1 upon CHD1-KD in both tested models. PMEPA1 is a known suppressor of PCa metastasis that regulates TGF-β signaling, a well-known determinant of metastasis [30]. Accordingly, we observed increased pSMAD2 levels (downstream target of the TGF-β pathway) in CHD1-KD xenograft tumors of both models. RNA-seq revealed an up-regulation of TGF-β targets like FOS, CAMK2N1, and RUNX3 [31–33]. C-FOS promotes the G0/G1 switch in the cell cycle [34], which might account for the improved outgrowth of lung metastases in the CHD1-KD xenografts. Correspondingly, several gene sets indicating cell cycle promotion (e.g., G1/S phase transition, DNA replication, cycling genes) were enriched in CHD1-depleted tumoroids. While our transcriptome analyses identified very promising candidates to explain the effect of CHD1 loss on PCa metastasis, future studies are required to mechanistically prove the suggested links such as the putative role of TGF-β signaling. On the other hand, further molecules such as TBX2 recently shown to be a downstream effector of CHD1 loss [24] and driver of PCa cell invasiveness in vivo [35] could not be found regulated upon CHD1-KD in this study.

Moreover, this study is limited by the lack of transcriptome data from the xenograft primary tumors (due to a high proportion of necrotic areas in ARCAP-M xenografts). Therefore, the transcriptome data revealed in this study (stemming from 3D tumoroids to mimic at least the 3D growth conditions in real tumors) cannot be directly linked to functional data. Nevertheless, the emerging picture of increased TGF-β signaling in the CHD1-KD tumoroids could be validated by increased pSMAD2 levels in the corresponding xenograft tumors of both models. Of note, PMEPA1 is also a direct AR target gene [36] and CHD1 loss alters the binding pattern and downstream signaling of AR [23]. Hence, at least in the ARCAP-M model, the PMEPA1 down-regulation could also result from altered AR signaling upon CHD1 loss (even though the similar observations with the PC-3 model argue against this hypothesis).

Our data show that determining the CHD1 deletion status in surgically treated patients helps to predict the risk of metastasis in R0 patients independent of established clinico-pathologic parameters such as preoperative PSA, pT stage, pN status, Gleason Score, and BCR. As a possible explanation we demonstrate improved metastatic outgrowth in CHD1-depleted human PCa xenografts. Transcriptome analyses uncovered candidate molecules known to regulate PCa metastasis formation that were affected by CHD1 depletion.

## Materials, patients, and methods

### Patient population, tissue microarray, and follow‐up

Patients underwent RP between 1998 and 2012 using an open retropubic approach or robot‐assisted laparoscopic approach (*n* = 7 902). Patients with (neo-)adjuvant androgen deprivation therapy (*n* = 502) and implausible values (*n* = 57) were excluded from analyses. For construction of PCa prognosis tissue microarrays (TMA), please see previous publications [5, 6]. Clinical follow-up data of patients were retrospectively analyzed. Data were collected prospectively into our institutional review board‐approved Martini Clinic database. Follow‐up data consisted of periodical PSA testing and postoperative imaging studies, which were performed according to PSA level and further clinical symptoms indicating recurrence. BCR was defined as PSA ≥ 0.2 ng/mL and rising after RP. MFS was defined as no radiological sign of metastasis in further performed imaging studies [37]. CSS was defined as the time from RP to death attributable to a PCa-related complication. All studies in humans have been carried out in accordance with the Declaration of Helsinki and written informed consents have been obtained from the patients.

### Statistical analyses

Baseline characteristics are presented for all 7343 patients. Continuous variables are reported as mean ± standard deviation (SD), and categorical variables are represented as frequencies and percentages. Because death was present as a competing risk, we used cause-specific Cox proportional hazards models to determine the effect of CHD1 on the three outcomes time to BCR, time to metastasis, and time to cancer-specific death. All models were further adjusted for age, PSA, Gleason Score, pN status, pT stage, year of RP, and surgical margin. For time to metastasis and time to cancer-specific death, preceding BCR was a further adjusting variable. Moreover, all significant two-way interactions of CHD1 with adjusting variables were added and kept in model if significant (backwards selection based on likelihood ratio tests). Results are presented as cause-specific HR together with 95% confidence intervals (CI). We assessed proportional hazards assumption by visual inspection of log–log plots and tested it on the basis of Schoenfeld residuals. As the proportional hazards assumption was violated for the year of surgery, all models were stratified for categorized year of surgery.

For the outcomes of in vivo studies, linear models were used with inclusion of interaction term (shNeg/shCHD1*PC-3/ARCAP-M) if significant and adjustment for growth period, tumor weight and number of CTC where applicable and significant.

All models present available case analyses. A two-tailed *p* < 0.05 was considered to be statistically significant. All analyses were performed using STATA 16 (StataCorp 2019).

### Fluorescence in situ hybridization

The molecular database of the Institute of Pathology of UKE included FISH data for the CHD1 deletion status, which were used for statistical analyses. Sample collection and FISH methodology on TMAs have been described before and were approved by the local ethics committee (Ärztekammer Hamburg, project number WF-049/09) [6]. FISH on PC-3 and ARCAP-M xenograft tumor sections has been performed accordingly.

### Cell culture and lentiviral transduction

PC-3 and ARCAP-M cells were obtained from ATCC and Novicure, respectively, and cultured as described before [25]. Cells were transduced with lentiviruses carrying either non-targeting control shRNA or shRNA against CHD1 and puromycin resistance. Knockdown (KD) and control cells were selected with puromycin (1.5 μg/mL). KD was validated by WB using anti-CHD1 (Santa Cruz Biotechnology, clone C-8, 1:1000) and anti-HSC70 (Santa Cruz Biotechnology, clone B-6, 1:4000) as a loading control.

### Spontaneous metastasis xenograft mouse model

CHD1-KD and control PC-3 and ARCaP-M cells were subcutaneously xenografted into immunodeficient pfp−/−/rag2−/− mice and primary tumors, blood and lungs were harvested as described before [25, 38]. The endpoint of the experiment was determined by the primary tumor size (~1.5 cm³). CTC in the blood and metastatic cell loads in the lung were quantified at necropsy by Alu-PCR (*n* = 9 per group in the PC-3 model, *n* = 7 per group in the ARCAP-M model) as previously described [25]. Histologic quantification of lung metastases in ten representative lung sections from four mice per group (PC-3 model) was performed as described [39]. Pulmonary metastases were categorized as single-cell metastases or multicellular colonies using a light microscope. Multicellular colonies were quantified by counting the total number of individual multicellular colonies (irrespective of the total number of single cells per multicellular colony). All animal experiments comply with the ARRIVE guidelines and have been approved by the local animal experiment approval committee (Behörde für Gesundheit und Verbraucherschutz, Hamburg).

### RNA sequencing of 3D tumoroids and qPCR validation

3D tumoroids were generated by cultivating PC-3 and ARCAP-M cells with or without CHD1-KD in poly-HEMA-treated cell culture flasks (*n* = 3 each). Established tumoroids were subjected to RNA extraction and RNA sequencing was performed as described [40]. Briefly, library preparation was done using a TruSeq RNA Library Prep Kit v2 (Illumina Inc., San Diego, CA, USA). Sequencing was done on an Illumina HiSeq 4000 platform. Sequences were mapped to UCSC hg19 as a reference genome. Differential gene expression was assessed using the Cuffdiff function of the Cufflinks package (v2.2.1). GSEA was performed with default settings (enrichment score was set to ≥0.45 and FDR *q*-value < 0.05). For validation, reverse transcription was performed using the Qiagen Omniscript RT Kit and PCR was performed with SsoFast EvaGreen Supermix on a CFX96 System (BioRad, Hercules, CA, USA). Primer sequences for RUNX3 were fw-GACTGTGATGGCAGGCAATG and rev-GGGTGAAACTCTTCCCTCGC, for PMEPA1 fw-GCAACTGCAAACGCTCTTTGT and rev-GGACCGTGCAGACAGCTTGTA. Gene expression was normalized to a control gene (SNRPD3) and displayed normalized to shNeg controls.

### Immunohistochemistry

pSMAD2 expression was determined on xenograft tumor sections using a polyclonal rabbit anti-pSMAD2(Ser465/467) antibody (Merck#ab3849-I) in a 1:640 working dilution (final concentration: 1 mg/mL). Dewaxed formalin-fixed paraffin-embedded sections were pre-treated with Fast Enzyme (Zytomed Systems, Berlin, Germany) for 5 min at room temperature. The primary antibody was incubated for 60 min at room temperature and unbound antibody was removed by multiple washing steps afterwards. Biotinylated swine-anti-rabbit was used as secondary antibody; antibody complexing, visualization, nuclei counterstaining, and isotype controls were conducted as described above.

## Supplementary information

Supplementary Information

Supplementary Figure S1

Supplementary Figure S2

Supplementary Figure S3

Supplementary Figure S4

Supplementary Material

Supplementary Material

Supplementary Material

Supplementary Material

uncropped WB

Ct values qPCR RUNX3

Ct values qPCR PMEPA1a
