# Supplementary material for: CHD1 loss negatively influences metastasis-free survival in R0-resected prostate cancer patients and promotes spontaneous metastasis in vivo
Source: Cancer Gene Ther. 2021 Jan 7;29(1):49–61. doi: 10.1038/s41417-020-00288-z (PMC8761572; doi:10.1038/s41417-020-00288-z)
Supplement: Supplementary file 1 — Supplementary Information [file 41417_2020_288_MOESM1_ESM.docx]

**Supplementary Information**

**Materials and Methods**

*Proliferation assay*

Effects of the CHD1-KD on tumor cell proliferation were assessed by seeding 1 x 10^4^ tumor cells per tissue culture dish and counting the number of viable tumor cells on d6 after seeding using a conventional cell counting chamber (trypan blue stain for exclusion of dead cells). This assay was performed in biological triplicates.

*Colony Formation assays*

The colony forming capacity of CHD1-KD and control tumor cells was compared in soft agar assays as described previously [26]. The number and diameter (µm) of all tumor spheres visible in one well were determined on day 19 (ARCAP-M) or day 17 (PC-3) after seeding. For each group, six wells were analyzed and the mean values per well were compared by Student’s t-test.

*Transwell invasion and migration assays*

The tumor cells’ migratory and invasive capacity was determined using Corning Fluroblok and CorningBioCoat transwell assays (8 µm pore size), respectively, as previously described [26]. The migration assays were performed in triplicates, the invasion assays in quadruplicates.

*Immunohistochemistry*

AR expression was determined by immunohistochemistry using the anti-AR clone AR441 (Dako #M3562) in a 1 : 25 working dilution (final concentration: 486.2 µg/ mL). Dewaxed FFPE sections of ARCAP-M xenograft primary tumors and positive control (human testis) were pre-treated with target retrieval solution (Dako #S2367, pH 9) for 10 min at 121 °C. The primary antibody was incubated for 60 min at room temperature (RT) and unbound antibody was removed by multiple washing steps afterwards. Biotinylated goat-anti-mouse was used as secondary antibody, incubated in a 1 : 200 dilution for 30 min at RT. Unbound secondary antibody was removed by washing and bound antibody complexes were visualized using the VectaStain ABC-AP Staining Kit (Vector Laboratories Inc., Burlingame, CA, USA) and Liquid Permanent Red (Dako). Nuclei were counterstained with Mayer’s hemalum solution for 5 sec. Isotype controls (mouse IgG1) were applied instead of the primary antibody on parallel sections.

**Supplementary figure legend**

**Suppl. Figure S1:** Cumulative incidence curves (Aalen-Johansen estimates) describing the association between *CHD1* deletion and time to BCR, metastasis, and cancer-specific death after radical prostatectomy of 6 831 prostate cancer patients (**A-C**). See Tables 1-4 for baseline characteristics of the study population and multivariate analyses.

**Suppl. Figure S2:** Immunohistochemical detection of AR expression in ARCAP-M xenograft tumors (**A**). Note the AR-positivity of the control tissue (human testis, left panel), which is absent in the isotype control (middle panel). Detection of normal *PTEN* copy numbers in ARCAP-M xenografts and homozygous deletion of *PTEN* in PC-3 cells as determined by FISH (**B**). *In vitro* characterization of tumor cell proliferation (**C**), colony formation in soft agar (**D**), transwell migration (**E**) and transwell invasion (**F**) of CHD1-KD and control PC-3 and ARCAP-M cells. Bar charts represent mean ± SD of n=3 (**C**), n=6 (**D**), n=3 (**E**), n=4 (**F**); **p*<0.05.

**Suppl. Figure S3:** HE-stained lung sections of PC-3 xenograft tumor-bearing mice providing further samples of single DTCs (as found in both control and CHD1-KD xenografts, left panel) and multicellular colonies (as predominantly found in CHD1-KD xenografts, right panel). White arrows point towards tumor cell nuclei.

**Suppl. Figure S4:** Enlarged version of Fig. 2D.
