# Supplementary figures and images for: CHD1 loss negatively influences metastasis-free survival in R0-resected prostate cancer patients and promotes spontaneous metastasis in vivo
Source: Cancer Gene Ther. 2021 Jan 7;29(1):49–61. doi: 10.1038/s41417-020-00288-z (PMC8761572; doi:10.1038/s41417-020-00288-z)

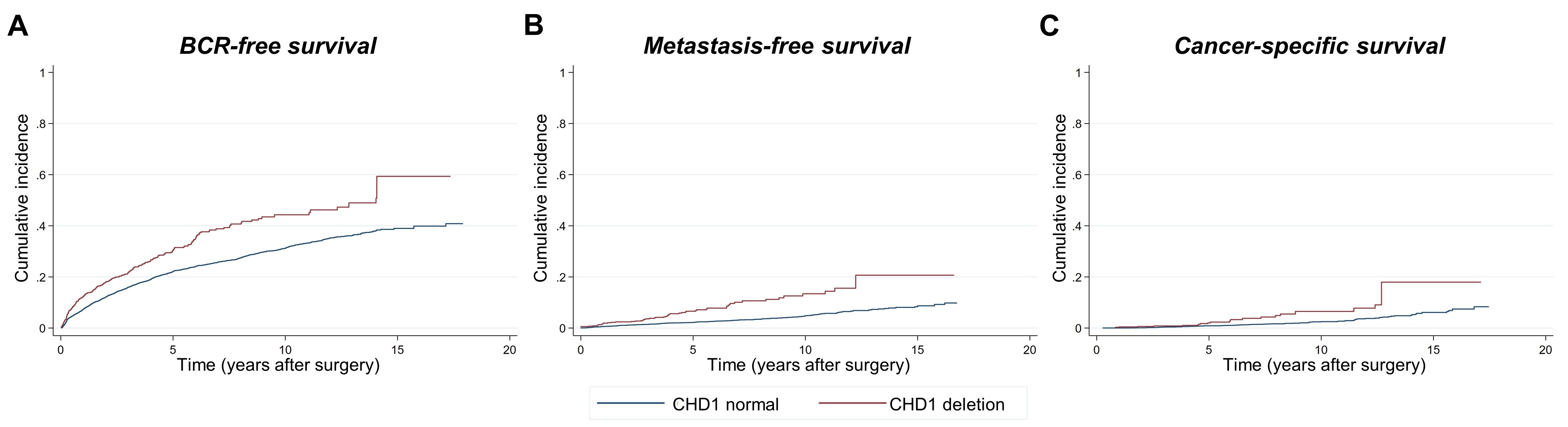

Supplement: Supplementary file 2 — Supplementary Figure S1 [file 41417_2020_288_MOESM2_ESM.jpg]

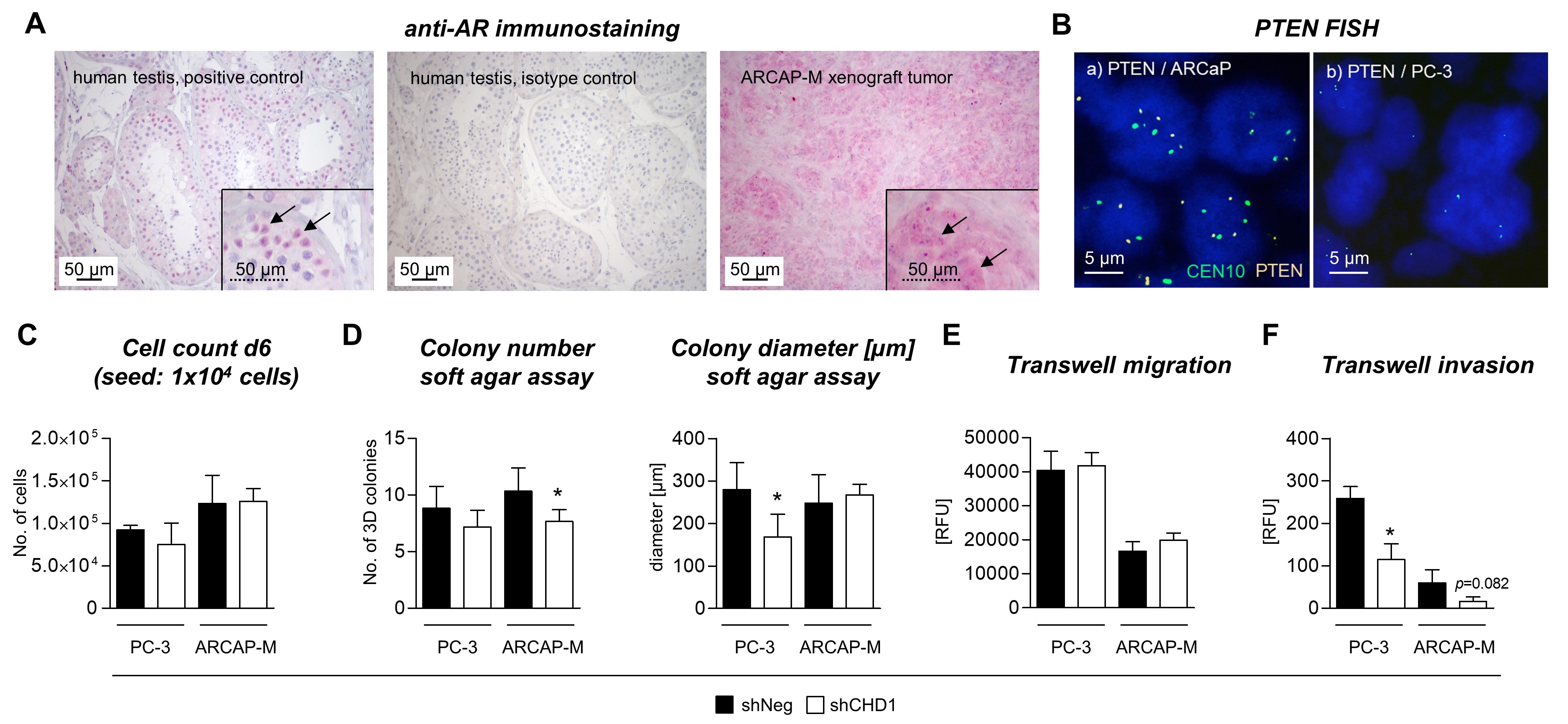

Supplement: Supplementary file 3 — Supplementary Figure S2 [file 41417_2020_288_MOESM3_ESM.jpg]

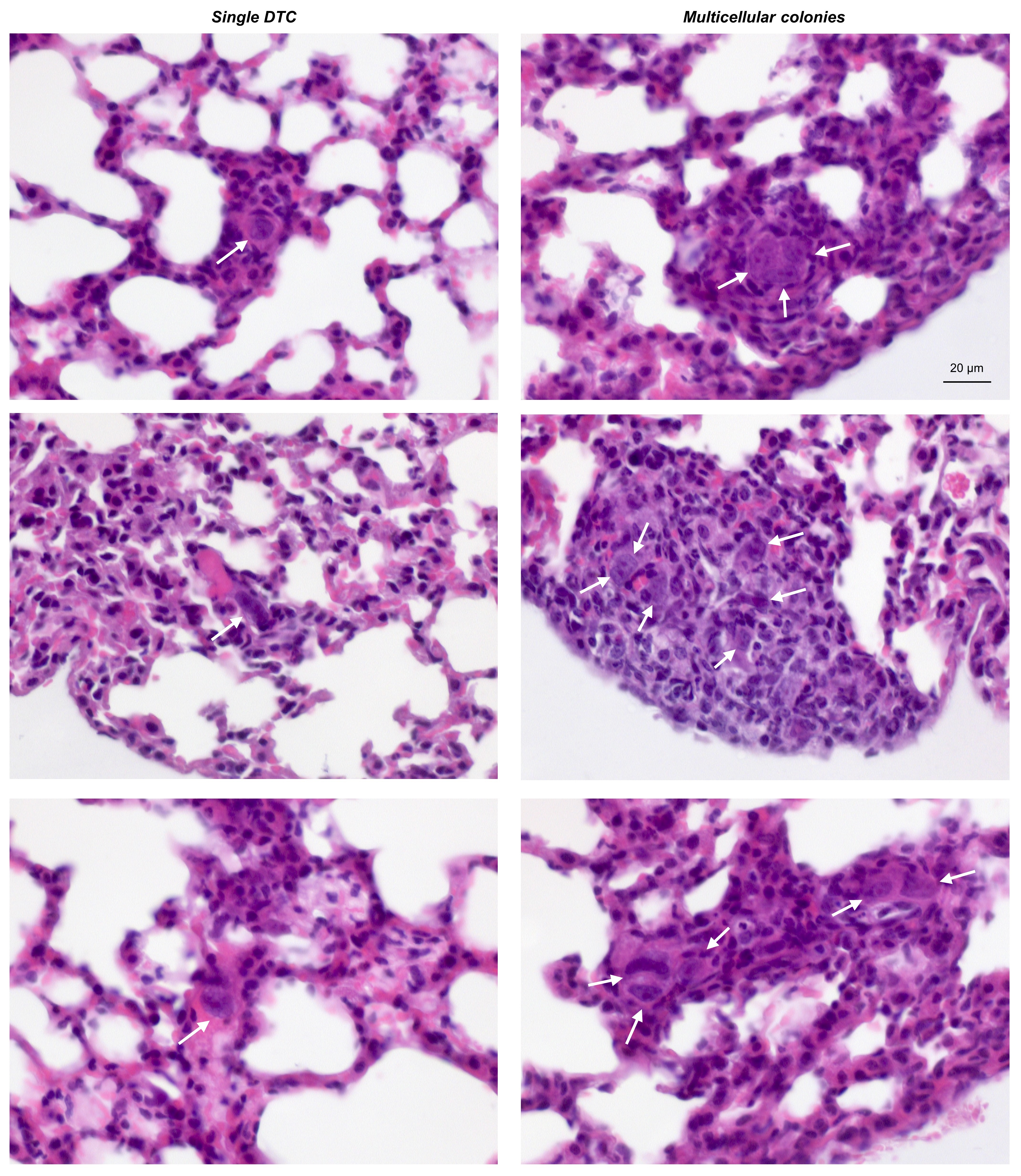

Supplement: Supplementary file 4 — Supplementary Figure S3 [file 41417_2020_288_MOESM4_ESM.jpg]

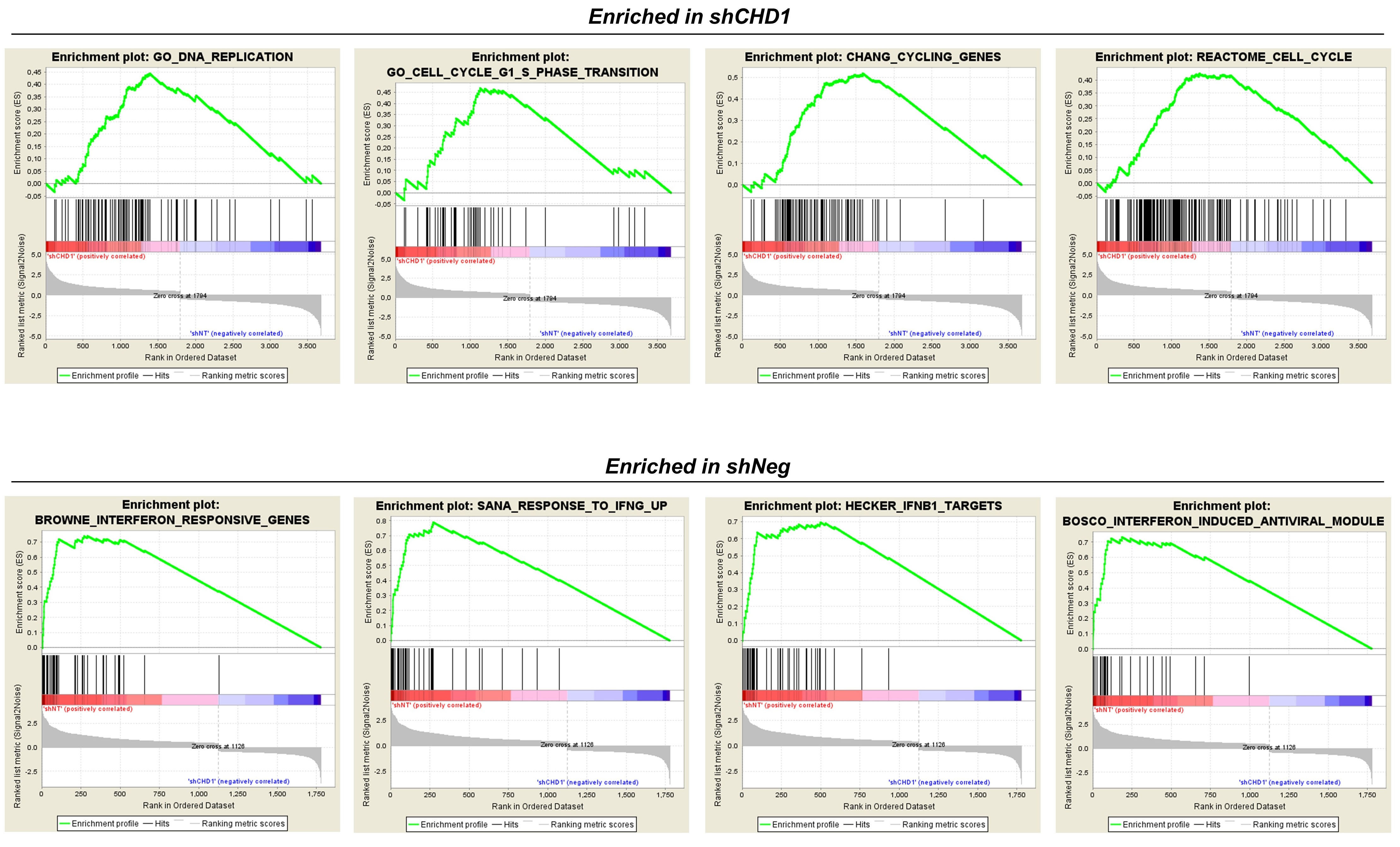

Supplement: Supplementary file 5 — Supplementary Figure S4 [file 41417_2020_288_MOESM5_ESM.jpg]

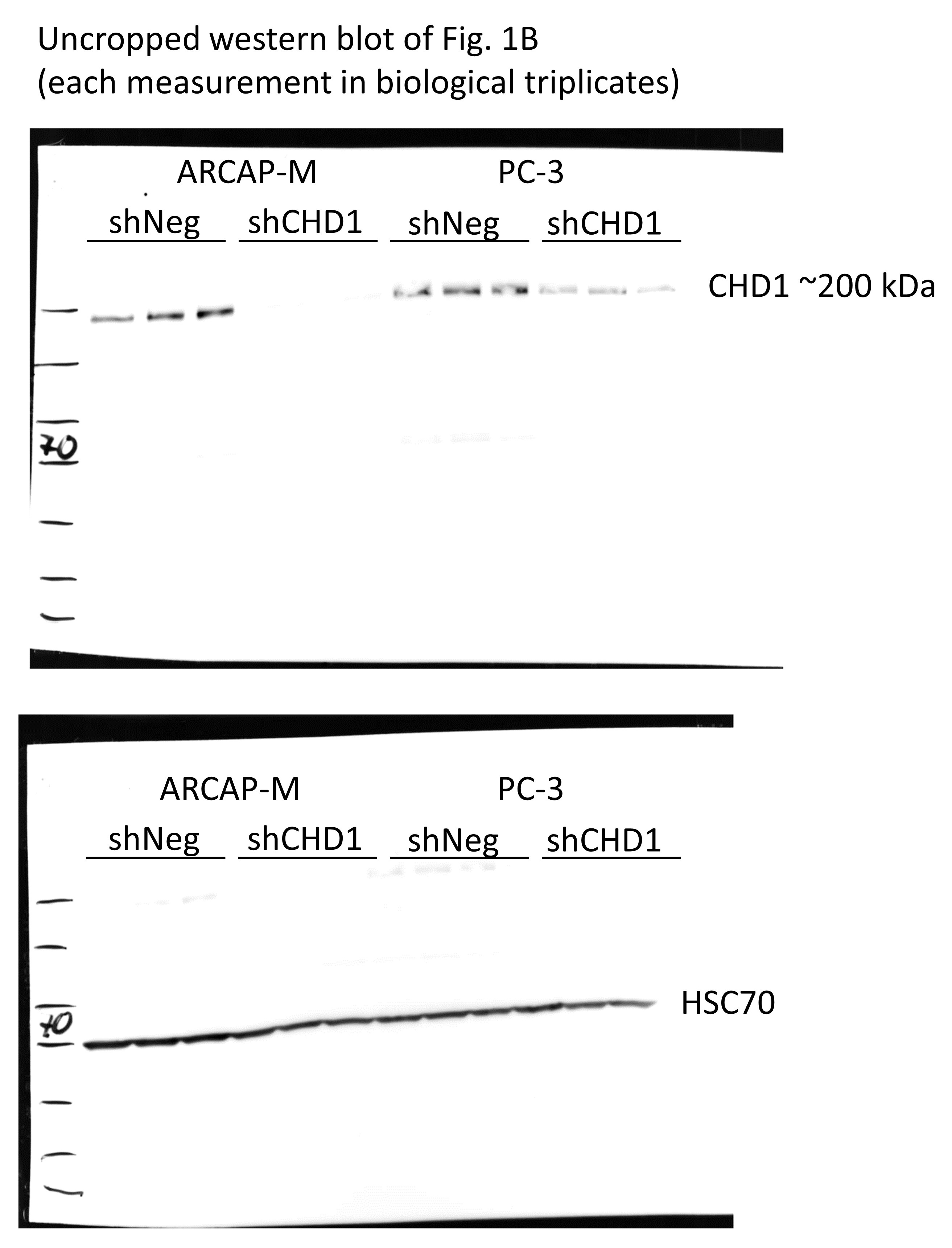

Supplement: Supplementary file 10 — uncropped WB [file 41417_2020_288_MOESM10_ESM.jpg]
