# Supplementary material for: CHD1 loss negatively influences metastasis-free survival in R0-resected prostate cancer patients and promotes spontaneous metastasis in vivo
Source: Cancer Gene Ther. 2021 Jan 7;29(1):49–61. doi: 10.1038/s41417-020-00288-z (PMC8761572; doi:10.1038/s41417-020-00288-z)
Supplement: Supplementary file 6 — Supplementary Material [file 41417_2020_288_MOESM6_ESM.pdf]

ARCAP_M	gene	locus	sample_1	sample_2	status	value_1	value_2	log2(fold_cha	test_stat	p_value	q_value	significant	PC-3	value_1	value_2	log2(fold_cha	p_value	q_value
	**CAMK2N1**	chr1:2080888	shneg	shCHD1	OK	6,1168	17,9863	1,55605	7,78751	5,00E-05	0,0004193	yes		2,33023	3,38226	0,537515	0,0003	0,00430043
	**CENPK**	chr5:6481359	shneg	shCHD1	OK	12,2553	17,537	0,517003	2,49888	5,00E-05	0,0004193	yes		6,78965	9,7096	0,516074	5,00E-05	0,00089885
	**DLC1**	chr8:1294087	shneg	shCHD1	OK	6,91712	10,5343	0,606854	3,18032	5,00E-05	0,0004193	yes		0,545804	0,926068	0,762734	5,00E-05	0,00089885
	**FOS**	chr14:757454	shneg	shCHD1	OK	23,1548	33,1391	0,517226	2,52422	0,0001	0,00078473	yes		1,33334	2,34842	0,816649	5,00E-05	0,00089885
	**HOXB6**	chr17:466678	shneg	shCHD1	OK	21,682	40,3377	0,895631	3,46301	5,00E-05	0,0004193	yes		2,54889	4,07785	0,677943	5,00E-05	0,00089885
	**IGFBP2**	chr2:2174981	shneg	shCHD1	OK	16,2426	25,5209	0,651894	3,19515	5,00E-05	0,0004193	yes		8,57374	18,9506	1,14425	5,00E-05	0,00089885
	**IL34**	chr16:706137	shneg	shCHD1	OK	3,10729	4,60057	0,566156	2,0828	0,0003	0,0020738	yes		9,55609	13,9277	0,543466	5,00E-05	0,00089885
	**RRM2**	chr2:1026269	shneg	shCHD1	OK	43,8166	72,2153	0,720827	4,12368	5,00E-05	0,0004193	yes		32,0104	47,1503	0,558727	5,00E-05	0,00089885
	**RUNX3**	chr1:2522600	shneg	shCHD1	OK	0,73754	1,90169	1,36649	4,49992	5,00E-05	0,0004193	yes		0,888451	1,57665	0,827495	5,00E-05	0,00089885
	**TGFA**	chr2:7067441	shneg	shCHD1	OK	10,8952	36,2993	1,73625	9,38679	5,00E-05	0,0004193	yes		2,04268	2,92052	0,51576	5,00E-05	0,00089885
	TLR8-AS1	chrX:1292093	shneg	shCHD1	OK	8,537	12,1892	0,513805	2,42957	5,00E-05	0,0004193	yes		39,1201	58,1679	0,572314	5,00E-05	0,00089885
	ZNF618	chr9:1166385	shneg	shCHD1	OK	4,92597	6,99357	0,505621	2,67955	0,00015	0,00112331	yes		1,5847	2,46513	0,637454	5,00E-05	0,00089885
