# Supplementary material for: CHD1 loss negatively influences metastasis-free survival in R0-resected prostate cancer patients and promotes spontaneous metastasis in vivo
Source: Cancer Gene Ther. 2021 Jan 7;29(1):49–61. doi: 10.1038/s41417-020-00288-z (PMC8761572; doi:10.1038/s41417-020-00288-z)
Supplement: Supplementary file 7 — Supplementary Material [file 41417_2020_288_MOESM7_ESM.pdf]

ARCAP_M	gene	locus	sample_1	sample_2	status	value_1	value_2	log2(fold_cha	test_stat	p_value	q_value	significant	PC-3	value_1	value_2	log2(fold_cha	p_value	q_value
	**IFI27**	chr14:945770	shneg	shCHD1	OK	120,055	19,46	-2,62511	-12,3472	5,00E-05	0,0004193	yes		66,4985	22,7318	-1,54861	5,00E-05	0,00089885
	**H19**	chr11:201640	shneg	shCHD1	OK	39,17	6,49447	-2,59247	-12,9506	5,00E-05	0,0004193	yes		2,29531	1,21959	-0,912297	5,00E-05	0,00089885
	**KRT20**	chr17:390321	shneg	shCHD1	OK	6,51106	1,45311	-2,16375	-6,70073	5,00E-05	0,0004193	yes		10,0302	3,98458	-1,33186	5,00E-05	0,00089885
	**APOL3**	chr22:365363	shneg	shCHD1	OK	0,988266	0,271575	-1,86355	-2,49183	5,00E-05	0,0004193	yes		11,1335	3,95788	-1,49211	5,00E-05	0,00089885
	**IFI16**	chr1:1589796	shneg	shCHD1	OK	0,826723	0,23382	-1,822	-2,90603	5,00E-05	0,0004193	yes		35,2272	22,5265	-0,645063	5,00E-05	0,00089885
	**PMEPA1**	chr20:562234	shneg	shCHD1	OK	49,337	14,2923	-1,78743	-9,84587	5,00E-05	0,0004193	yes		1,14319	0,622919	-0,875948	5,00E-05	0,00089885
	**CRABP2**	chr1:1566693	shneg	shCHD1	OK	132,954	44,7795	-1,57001	-8,42236	5,00E-05	0,0004193	yes		57,598	35,1687	-0,711725	5,00E-05	0,00089885
	**SLAIN1**	chr13:782719	shneg	shCHD1	OK	0,91839	0,352615	-1,38101	-1,98018	0,0001	0,00078473	yes		11,4386	5,61471	-1,02663	5,00E-05	0,00089885
	**ASS1**	chr9:1333200	shneg	shCHD1	OK	1416,89	586,617	-1,27223	-7,6941	5,00E-05	0,0004193	yes		20,6981	14,5521	-0,508268	5,00E-05	0,00089885
	**S100A4**	chr1:1535160	shneg	shCHD1	OK	3212,65	1352,19	-1,24846	-7,42528	5,00E-05	0,0004193	yes		377,797	203,229	-0,894504	5,00E-05	0,00089885
	DENND2D	chr1:1117298	shneg	shCHD1	OK	1,09222	0,469823	-1,21708	-2,30533	0,00115	0,00656221	yes		2,82679	1,6044	-0,817135	5,00E-05	0,00089885
	LMTK3	chr19:489885	shneg	shCHD1	OK	0,878442	0,394582	-1,15462	-3,11213	5,00E-05	0,0004193	yes		0,857073	0,595887	-0,524379	0,00575	0,0443088
	GBP3	chr1:8947235	shneg	shCHD1	OK	3,2245	1,48662	-1,11704	-4,14804	5,00E-05	0,0004193	yes		18,7682	11,342	-0,726624	5,00E-05	0,00089885
	SECTM1	chr17:802788	shneg	shCHD1	OK	22,7208	10,6579	-1,09209	-5,49835	5,00E-05	0,0004193	yes		21,4401	12,2113	-0,812095	5,00E-05	0,00089885
	FLT3LG	chr19:499774	shneg	shCHD1	OK	4,31578	2,02579	-1,09114	-2,73279	5,00E-05	0,0004193	yes		1,91351	1,01378	-0,916479	0,0055	0,0428566
	FAM129A	chr1:1847601	shneg	shCHD1	OK	4,13441	1,95103	-1,08344	-5,34223	5,00E-05	0,0004193	yes		3,25023	1,67978	-0,95227	5,00E-05	0,00089885
	EML6	chr2:5495214	shneg	shCHD1	OK	0,905352	0,430381	-1,07286	-3,6095	5,00E-05	0,0004193	yes		4,22228	2,68154	-0,654963	5,00E-05	0,00089885
	HES4	chr1:934341-	shneg	shCHD1	OK	9,29437	4,43999	-1,0658	-3,57226	5,00E-05	0,0004193	yes		18,0082	11,5216	-0,644313	5,00E-05	0,00089885
	UBA7	chr3:4984263	shneg	shCHD1	OK	1,86675	0,896102	-1,0588	-3,29627	5,00E-05	0,0004193	yes		5,20478	2,34421	-1,15073	5,00E-05	0,00089885
	TAPBPL	chr12:656117	shneg	shCHD1	OK	10,0709	4,99996	-1,01021	-2,5886	0,0001	0,00078473	yes		3,26329	1,66893	-0,9674	0,00035	0,00486945
	RNASE4	chr14:211523	shneg	shCHD1	OK	8,93406	4,53211	-0,979132	-2,93549	5,00E-05	0,0004193	yes		56,4083	39,0636	-0,530083	5,00E-05	0,00089885
	RAB31	chr18:970822	shneg	shCHD1	OK	1,36023	0,704383	-0,949419	-2,91173	5,00E-05	0,0004193	yes		5,11658	3,40492	-0,587556	5,00E-05	0,00089885
	APOL1	chr22:366491	shneg	shCHD1	OK	6,92312	3,59549	-0,945233	-4,30602	5,00E-05	0,0004193	yes		4,08238	1,11669	-1,87019	5,00E-05	0,00089885
	SH3PXD2A	chr10:105353	shneg	shCHD1	OK	1,54173	0,821919	-0,907482	-4,00479	5,00E-05	0,0004193	yes		4,8965	3,3197	-0,560699	5,00E-05	0,00089885
	NPW	chr16:206952	shneg	shCHD1	OK	22,5068	12,2868	-0,873254	-4,01047	5,00E-05	0,0004193	yes		6,02829	1,56357	-1,94691	5,00E-05	0,00089885
	C1R	chr12:718751	shneg	shCHD1	OK	3,08879	1,7018	-0,859983	-2,8965	5,00E-05	0,0004193	yes		2,13337	0,692733	-1,62276	5,00E-05	0,00089885
	TNFRSF14	chr1:2487207	shneg	shCHD1	OK	24,6876	13,8742	-0,831378	-4,08237	5,00E-05	0,0004193	yes		5,5427	2,87072	-0,949177	5,00E-05	0,00089885
	FLJ39653	chr4:1622828	shneg	shCHD1	OK	1,91828	1,08316	-0,82457	-2,42341	0,0001	0,00078473	yes		2,21572	1,47488	-0,587175	0,00065	0,00800759
	FHL1	chrX:1352288	shneg	shCHD1	OK	265,727	152,108	-0,804849	-4,74401	5,00E-05	0,0004193	yes		19,105	9,77813	-0,966323	5,00E-05	0,00089885
	NPTXR	chr22:392144	shneg	shCHD1	OK	22,7503	13,3411	-0,770006	-4,25098	5,00E-05	0,0004193	yes		2,23421	1,29748	-0,784055	5,00E-05	0,00089885
	XK	chrX:3754513	shneg	shCHD1	OK	2,2147	1,30583	-0,762146	-3,16261	5,00E-05	0,0004193	yes		11,4057	7,21173	-0,661337	5,00E-05	0,00089885
	HPGD	chr4:1754113	shneg	shCHD1	OK	225,068	133,644	-0,751963	-4,45274	5,00E-05	0,0004193	yes		33,5362	16,5993	-1,0146	5,00E-05	0,00089885
	NPNT	chr4:1068165	shneg	shCHD1	OK	114,309	68,7373	-0,733769	-4,30713	5,00E-05	0,0004193	yes		13,0651	7,44131	-0,812088	5,00E-05	0,00089885
	ST6GALNAC2	chr17:745614	shneg	shCHD1	OK	3,89257	2,34454	-0,731417	-2,67951	5,00E-05	0,0004193	yes		8,55943	4,8892	-0,807917	5,00E-05	0,00089885
	KLHL35	chr11:751334	shneg	shCHD1	OK	3,6394	2,23498	-0,703439	-2,42216	0,0001	0,00078473	yes		7,57512	5,01829	-0,594072	5,00E-05	0,00089885
	OAS1	chr12:113344	shneg	shCHD1	OK	76,6079	48,5128	-0,659127	-3,58846	5,00E-05	0,0004193	yes		28,1823	16,9771	-0,731201	5,00E-05	0,00089885
	MRC2	chr17:607047	shneg	shCHD1	OK	29,6336	18,8491	-0,652737	-3,66789	5,00E-05	0,0004193	yes		2,84216	1,5985	-0,830269	5,00E-05	0,00089885
	SLC40A1	chr2:1904253	shneg	shCHD1	OK	1,06242	0,686092	-0,630875	-1,70888	0,00415	0,0191853	yes		1,58098	0,877393	-0,84952	5,00E-05	0,00089885
	GJA3	chr13:207123	shneg	shCHD1	OK	3,7792	2,48731	-0,603496	-2,87015	5,00E-05	0,0004193	yes		1,56745	1,00821	-0,636626	5,00E-05	0,00089885
	B3GNT3	chr19:179059	shneg	shCHD1	OK	1,67561	1,10311	-0,603114	-1,80551	0,00295	0,0144329	yes		15,5281	9,72135	-0,675657	5,00E-05	0,00089885
	DDX60	chr4:1691374	shneg	shCHD1	OK	6,3125	4,16465	-0,600016	-3,01336	5,00E-05	0,0004193	yes		6,48649	2,78485	-1,21984	5,00E-05	0,00089885
	BTN3A2	chr6:2636539	shneg	shCHD1	OK	6,39248	4,21991	-0,599163	-2,83911	5,00E-05	0,0004193	yes		8,2177	4,70379	-0,804911	5,00E-05	0,00089885
	LMCD1	chr3:8543510	shneg	shCHD1	OK	11,6768	7,78416	-0,58503	-2,75568	5,00E-05	0,0004193	yes		2,52592	0,990512	-1,35056	5,00E-05	0,00089885
	CARD10	chr22:378863	shneg	shCHD1	OK	13,45	8,98175	-0,582539	-3,01688	5,00E-05	0,0004193	yes		10,7522	7,59665	-0,501198	5,00E-05	0,00089885
	KCNMB4	chr12:707600	shneg	shCHD1	OK	1,39937	0,93733	-0,578145	-2,04411	0,0005	0,00321686	yes		6,651	2,06264	-1,68908	5,00E-05	0,00089885
	KIAA1211	chr4:5703636	shneg	shCHD1	OK	5,81063	3,8935	-0,577628	-2,92761	5,00E-05	0,0004193	yes		6,9456	2,96439	-1,22837	5,00E-05	0,00089885
	TNFSF10	chr3:1722232	shneg	shCHD1	OK	6,10309	4,10199	-0,573216	-2,38321	5,00E-05	0,0004193	yes		2,01415	0,884046	-1,18798	0,0001	0,00166847
	SAMD9	chr7:9272882	shneg	shCHD1	OK	2,07533	1,39971	-0,568213	-2,6222	5,00E-05	0,0004193	yes		6,83899	4,6529	-0,555654	5,00E-05	0,00089885
	FYN	chr6:1119815	shneg	shCHD1	OK	1,16359	0,786967	-0,564205	-1,53451	0,00645	0,0276097	yes		5,75212	3,81265	-0,593301	5,00E-05	0,00089885
	UBE2L6	chr11:573191	shneg	shCHD1	OK	68,9381	46,8936	-0,55591	-2,97357	5,00E-05	0,0004193	yes		79,6735	34,7975	-1,19512	5,00E-05	0,00089885
	APOL6	chr22:360444	shneg	shCHD1	OK	4,77562	3,29304	-0,536267	-2,77187	5,00E-05	0,0004193	yes		11,6575	6,43603	-0,85701	5,00E-05	0,00089885
	NAGPA	chr16:507484	shneg	shCHD1	OK	5,45885	3,79445	-0,524706	-2,23973	0,0001	0,00078473	yes		9,14025	6,39933	-0,514312	5,00E-05	0,00089885
	GAS6	chr13:114523	shneg	shCHD1	OK	58,2301	40,9149	-0,50914	-2,71721	5,00E-05	0,0004193	yes		4,57102	3,01404	-0,600819	5,00E-05	0,00089885
