# Supplementary material for: CHD1 loss negatively influences metastasis-free survival in R0-resected prostate cancer patients and promotes spontaneous metastasis in vivo
Source: Cancer Gene Ther. 2021 Jan 7;29(1):49–61. doi: 10.1038/s41417-020-00288-z (PMC8761572; doi:10.1038/s41417-020-00288-z)
Supplement: Supplementary file 8 — Supplementary Material [file 41417_2020_288_MOESM8_ESM.pdf]

test_id	gene_id	gene	locus	sample_1	sample_2	status	value_1	value_2	log2(fold_cha	test_stat	p_value	q_value	significant
ODF3B	ODF3B	ODF3B	chr22:509688	shneg	shCHD1	OK	1,85366	0,926751	-1,00012	-1,62191	0,00545	0,0425924	yes
IFIH1	IFIH1	IFIH1	chr2:1631235	shneg	shCHD1	OK	5,85892	2,92759	-1,00092	-5,66427	5,00E-05	0,00089885	yes
HPGD	HPGD	HPGD	chr4:1754113	shneg	shCHD1	OK	33,5362	16,5993	-1,0146	-6,84826	5,00E-05	0,00089885	yes
SHC2	SHC2	SHC2	chr19:416582	shneg	shCHD1	OK	1,41443	0,695895	-1,02328	-2,59019	0,0001	0,00166847	yes
SLAIN1	SLAIN1	SLAIN1	chr13:782719	shneg	shCHD1	OK	11,4386	5,61471	-1,02663	-5,7845	5,00E-05	0,00089885	yes
DACH1	DACH1	DACH1	chr13:720120	shneg	shCHD1	OK	3,88411	1,89334	-1,03665	-5,83894	5,00E-05	0,00089885	yes
IL32	IL32	IL32	chr16:311531	shneg	shCHD1	OK	3,86776	1,88266	-1,03873	-2,10317	0,0001	0,00166847	yes
SLC22A31	SLC22A31	SLC22A31	chr16:892621	shneg	shCHD1	OK	2,7723	1,34895	-1,03924	-3,13785	5,00E-05	0,00089885	yes
SH2D1B	SH2D1B	SH2D1B	chr1:1623650	shneg	shCHD1	OK	0,847255	0,412192	-1,03948	-2,02696	0,0014	0,0148187	yes
NELL2	NELL2	NELL2	chr12:449020	shneg	shCHD1	OK	46,4963	22,6197	-1,03953	-6,55301	5,00E-05	0,00089885	yes
HLA-F	HLA-F	HLA-F	chr6_ssto_ha	shneg	shCHD1	OK	14,5962	7,04507	-1,05091	-5,07183	5,00E-05	0,00089885	yes
NLRC5	NLRC5	NLRC5	chr16:570509	shneg	shCHD1	OK	7,4115	3,5767	-1,05114	-6,84182	5,00E-05	0,00089885	yes
HSPB8	HSPB8	HSPB8	chr12:119616	shneg	shCHD1	OK	10,3145	4,97758	-1,05115	-5,8527	5,00E-05	0,00089885	yes
MLLT11	MLLT11	MLLT11	chr1:1510321	shneg	shCHD1	OK	3,15447	1,51635	-1,05679	-3,68619	5,00E-05	0,00089885	yes
TUBB2B	TUBB2B	TUBB2B	chr6:3224494	shneg	shCHD1	OK	6,37062	3,03941	-1,06764	-5,06367	5,00E-05	0,00089885	yes
PLA2G4C	PLA2G4C	PLA2G4C	chr19:485510	shneg	shCHD1	OK	1,01117	0,480171	-1,07441	-2,08459	0,00045	0,00597786	yes
ATF3	ATF3	ATF3	chr1:2127386	shneg	shCHD1	OK	3,8102	1,79573	-1,0853	-3,45852	5,00E-05	0,00089885	yes
IL20RA	IL20RA	IL20RA	chr6:1373211	shneg	shCHD1	OK	1,79721	0,84191	-1,09402	-3,76844	5,00E-05	0,00089885	yes
IL15RA	IL15RA	IL15RA	chr10:599433	shneg	shCHD1	OK	11,0176	5,14854	-1,09758	-5,34799	5,00E-05	0,00089885	yes
HLA-C	HLA-C	HLA-C	chr6_ssto_ha	shneg	shCHD1	OK	102,936	47,9698	-1,10155	-6,94944	5,00E-05	0,00089885	yes
SRGN	SRGN	SRGN	chr10:708478	shneg	shCHD1	OK	4,35469	2,02283	-1,10619	-3,18239	5,00E-05	0,00089885	yes
TLR4	TLR4	TLR4	chr9:1204664	shneg	shCHD1	OK	1,65107	0,764501	-1,11081	-4,1319	5,00E-05	0,00089885	yes
BATF2	BATF2	BATF2	chr11:647554	shneg	shCHD1	OK	2,43409	1,12554	-1,11277	-3,34382	5,00E-05	0,00089885	yes
WARS	WARS	WARS	chr14:100800	shneg	shCHD1	OK	154,565	71,2155	-1,11795	-7,34363	5,00E-05	0,00089885	yes
IFI44	IFI44	IFI44	chr1:7911547	shneg	shCHD1	OK	19,8569	9,13848	-1,11961	-6,87018	5,00E-05	0,00089885	yes
UBA7	UBA7	UBA7	chr3:4984263	shneg	shCHD1	OK	5,20478	2,34421	-1,15073	-5,93794	5,00E-05	0,00089885	yes
IFI35	IFI35	IFI35	chr17:411587	shneg	shCHD1	OK	20,3724	9,16992	-1,15164	-6,63193	5,00E-05	0,00089885	yes
MUC13	MUC13	MUC13	chr3:1246242	shneg	shCHD1	OK	9,13743	4,07374	-1,16544	-7,07324	5,00E-05	0,00089885	yes
BASP1	BASP1	BASP1	chr5:1721774	shneg	shCHD1	OK	5,16872	2,30242	-1,16666	-4,56074	5,00E-05	0,00089885	yes
IFIT2	IFIT2	IFIT2	chr10:910617	shneg	shCHD1	OK	8,37402	3,72941	-1,16697	-7,13108	5,00E-05	0,00089885	yes
B4GALNT1	B4GALNT1	B4GALNT1	chr12:580136	shneg	shCHD1	OK	4,56728	2,02641	-1,17241	-5,30023	5,00E-05	0,00089885	yes
SYT12	SYT12	SYT12	chr11:667901	shneg	shCHD1	OK	1,37363	0,609286	-1,1728	-3,36182	5,00E-05	0,00089885	yes
VGF	VGF	VGF	chr7:1008057	shneg	shCHD1	OK	3,23303	1,4249	-1,18203	-4,45677	5,00E-05	0,00089885	yes
CYP1B1	CYP1B1	CYP1B1	chr2:3829474	shneg	shCHD1	OK	5,7084	2,50762	-1,18677	-7,56896	5,00E-05	0,00089885	yes
TNFSF10	TNFSF10	TNFSF10	chr3:1722232	shneg	shCHD1	OK	2,01415	0,884046	-1,18798	-2,58275	0,0001	0,00166847	yes
UBE2L6	UBE2L6	UBE2L6	chr11:573191	shneg	shCHD1	OK	79,6735	34,7975	-1,19512	-7,88104	5,00E-05	0,00089885	yes
THBS2	THBS2	THBS2	chr6:1696158	shneg	shCHD1	OK	0,789777	0,344394	-1,19739	-3,42235	5,00E-05	0,00089885	yes
SYNGR3	SYNGR3	SYNGR3	chr16:203994	shneg	shCHD1	OK	1,318	0,572191	-1,20379	-2,52549	0,00025	0,00369169	yes
NPY1R	NPY1R	NPY1R	chr4:1642451	shneg	shCHD1	OK	5,83723	2,52719	-1,20775	-6,0926	5,00E-05	0,00089885	yes
RSAD2	RSAD2	RSAD2	chr2:7017795	shneg	shCHD1	OK	1,42396	0,61186	-1,21863	-3,57242	5,00E-05	0,00089885	yes
PARM1	PARM1	PARM1	chr4:7585828	shneg	shCHD1	OK	2,94523	1,2652	-1,21902	-5,94728	5,00E-05	0,00089885	yes
DDX60	DDX60	DDX60	chr4:1691374	shneg	shCHD1	OK	6,48649	2,78485	-1,21984	-7,68109	5,00E-05	0,00089885	yes
KIAA1211	KIAA1211	KIAA1211	chr4:5703636	shneg	shCHD1	OK	6,9456	2,96439	-1,22837	-7,81312	5,00E-05	0,00089885	yes
TNFRSF1B	TNFRSF1B	TNFRSF1B	chr1:1222705	shneg	shCHD1	OK	2,61487	1,11457	-1,23026	-4,99976	5,00E-05	0,00089885	yes
IFI6	IFI6	IFI6	chr1:2799257	shneg	shCHD1	OK	113,443	48,2717	-1,23272	-7,91565	5,00E-05	0,00089885	yes
IFIT3	IFIT3	IFIT3	chr10:910876	shneg	shCHD1	OK	16,6931	7,09399	-1,23459	-7,76458	5,00E-05	0,00089885	yes
HLA-G	HLA-G	HLA-G	chr6_ssto_ha	shneg	shCHD1	OK	2,94838	1,24962	-1,23843	-3,31286	5,00E-05	0,00089885	yes
MST4	MST4	MST4	chrX:1311572	shneg	shCHD1	OK	1,5536	0,657822	-1,23984	-3,25973	5,00E-05	0,00089885	yes
PLA2G7	PLA2G7	PLA2G7	chr6:4665561	shneg	shCHD1	OK	4,65642	1,95411	-1,25271	-4,3006	5,00E-05	0,00089885	yes
ACSL5	ACSL5	ACSL5	chr10:114133	shneg	shCHD1	OK	1,65187	0,690829	-1,2577	-3,57532	5,00E-05	0,00089885	yes
OAS2	OAS2	OAS2	chr12:113416	shneg	shCHD1	OK	21,2854	8,86511	-1,26366	-7,64385	5,00E-05	0,00089885	yes
PCSK5	PCSK5	PCSK5	chr9:7850555	shneg	shCHD1	OK	0,987646	0,411101	-1,2645	-2,93961	5,00E-05	0,00089885	yes
COL8A1	COL8A1	COL8A1	chr3:9927315	shneg	shCHD1	OK	3,12375	1,29945	-1,26538	-2,12972	0,00085	0,00989996	yes
HERC6	HERC6	HERC6	chr4:8929989	shneg	shCHD1	OK	3,88954	1,61798	-1,26541	-6,00864	5,00E-05	0,00089885	yes
MYL10	MYL10	MYL10	chr7:1012566	shneg	shCHD1	OK	6,7708	2,78255	-1,28292	-3,99413	5,00E-05	0,00089885	yes
TMC5	TMC5	TMC5	chr16:194220	shneg	shCHD1	OK	2,7982	1,14503	-1,28911	-5,94402	5,00E-05	0,00089885	yes
LRG1	LRG1	LRG1	chr19:453722	shneg	shCHD1	OK	1,53961	0,629746	-1,28972	-2,65522	0,0002	0,00303812	yes
EFCAB1	EFCAB1	EFCAB1	chr8:4962747	shneg	shCHD1	OK	0,825352	0,33381	-1,30598	-1,82835	0,002	0,0196597	yes
IFI44L	IFI44L	IFI44L	chr1:7908608	shneg	shCHD1	OK	5,69311	2,29653	-1,30976	-7,98037	5,00E-05	0,00089885	yes
LIPG	LIPG	LIPG	chr18:470884	shneg	shCHD1	OK	4,1627	1,66981	-1,31784	-6,57404	5,00E-05	0,00089885	yes
UTS2D	UTS2D	UTS2D	chr3:1909849	shneg	shCHD1	OK	25,1285	10,0484	-1,32236	-2,65638	5,00E-05	0,00089885	yes
KRT20	KRT20	KRT20	chr17:390321	shneg	shCHD1	OK	10,0302	3,98458	-1,33186	-6,57619	5,00E-05	0,00089885	yes
CACNA1H	CACNA1H	CACNA1H	chr16:120324	shneg	shCHD1	OK	6,47349	2,55519	-1,34111	-8,54293	5,00E-05	0,00089885	yes
LMCD1	LMCD1	LMCD1	chr3:8543510	shneg	shCHD1	OK	2,52592	0,990512	-1,35056	-3,45683	5,00E-05	0,00089885	yes
XAF1	XAF1	XAF1	chr17:665915	shneg	shCHD1	OK	5,01314	1,92476	-1,38104	-6,83852	5,00E-05	0,00089885	yes
GBP5	GBP5	GBP5	chr1:8972463	shneg	shCHD1	OK	1,24673	0,474909	-1,39243	-3,53077	5,00E-05	0,00089885	yes
HLA-B	HLA-B	HLA-B	chr6_ssto_ha	shneg	shCHD1	OK	107,673	40,9902	-1,39331	-8,72354	5,00E-05	0,00089885	yes
GRAMD3	GRAMD3	GRAMD3	chr5:1256957	shneg	shCHD1	OK	2,76523	1,0465	-1,40183	-4,28809	5,00E-05	0,00089885	yes
PSMB8	PSMB8	PSMB8	chr6_ssto_ha	shneg	shCHD1	OK	46,895	17,6858	-1,40684	-4,24391	5,00E-05	0,00089885	yes
ABCG1	ABCG1	ABCG1	chr21:436197	shneg	shCHD1	OK	13,7365	5,1599	-1,4126	-8,81077	5,00E-05	0,00089885	yes
IFIT1	IFIT1	IFIT1	chr10:911523	shneg	shCHD1	OK	16,9545	6,33491	-1,42027	-8,62683	5,00E-05	0,00089885	yes
SAMD9L	SAMD9L	SAMD9L	chr7:9275936	shneg	shCHD1	OK	5,21714	1,94069	-1,42669	-8,84621	5,00E-05	0,00089885	yes
GLB1L3	GLB1L3	GLB1L3	chr11:134146	shneg	shCHD1	OK	6,43654	2,38948	-1,42959	-7,20702	5,00E-05	0,00089885	yes
MMP13	MMP13	MMP13	chr11:102813	shneg	shCHD1	OK	8,42448	3,0967	-1,44386	-7,82989	5,00E-05	0,00089885	yes
SORCS2	SORCS2	SORCS2	chr4:7194373	shneg	shCHD1	OK	1,31913	0,4744	-1,47541	-4,98853	5,00E-05	0,00089885	yes
APOL3	APOL3	APOL3	chr22:365363	shneg	shCHD1	OK	11,1335	3,95788	-1,49211	-7,87712	5,00E-05	0,00089885	yes
RGS4	RGS4	RGS4	chr1:1630383	shneg	shCHD1	OK	24,2051	8,32819	-1,53923	-9,42629	5,00E-05	0,00089885	yes
IFI27	IFI27	IFI27	chr14:945770	shneg	shCHD1	OK	66,4985	22,7318	-1,54861	-9,41283	5,00E-05	0,00089885	yes
BATF3	BATF3	BATF3	chr1:2128597	shneg	shCHD1	OK	4,09272	1,37881	-1,56964	-3,46591	5,00E-05	0,00089885	yes
C1R	C1R	C1R	chr12:718751	shneg	shCHD1	OK	2,13337	0,692733	-1,62276	-4,11312	5,00E-05	0,00089885	yes
KCNMB4	KCNMB4	KCNMB4	chr12:707600	shneg	shCHD1	OK	6,651	2,06264	-1,68908	-9,99062	5,00E-05	0,00089885	yes
TAP1	TAP1	TAP1	chr6_ssto_ha	shneg	shCHD1	OK	53,1371	16,435	-1,69295	-9,00649	5,00E-05	0,00089885	yes
FCGBP	FCGBP	FCGBP	chr19:403539	shneg	shCHD1	OK	24,9973	7,6752	-1,7035	-11,0979	5,00E-05	0,00089885	yes
TMEM71	TMEM71	TMEM71	chr8:1337221	shneg	shCHD1	OK	1,53898	0,464015	-1,72973	-2,61978	5,00E-05	0,00089885	yes
RTP4	RTP4	RTP4	chr3:1870861	shneg	shCHD1	OK	1,83518	0,548795	-1,74158	-2,26293	0,00325	0,0284397	yes
LGR5	LGR5	LGR5	chr12:718338	shneg	shCHD1	OK	3,6062	1,0754	-1,7456	-6,39951	5,00E-05	0,00089885	yes
LYPD5	LYPD5	LYPD5	chr19:443000	shneg	shCHD1	OK	1,9156	0,569548	-1,74991	-4,16669	5,00E-05	0,00089885	yes
MX2	MX2	MX2	chr21:427339	shneg	shCHD1	OK	1,89364	0,555681	-1,76883	-4,72472	5,00E-05	0,00089885	yes
TGM2	TGM2	TGM2	chr20:367568	shneg	shCHD1	OK	8,59742	2,48912	-1,78827	-8,48268	5,00E-05	0,00089885	yes
LOC201651	LOC201651	LOC201651	chr3:1513473	shneg	shCHD1	OK	1,13839	0,316301	-1,84762	-2,07192	0,00375	0,0316834	yes
APOL1	APOL1	APOL1	chr22:366491	shneg	shCHD1	OK	4,08238	1,11669	-1,87019	-5,70182	5,00E-05	0,00089885	yes
SYT13	SYT13	SYT13	chr11:452618	shneg	shCHD1	OK	0,925763	0,250596	-1,88528	-4,48274	5,00E-05	0,00089885	yes

TCN1	TCN1	TCN1	chr11:596202	shneg	shCHD1	OK	305,235	82,2122	-1,89249	-12,3282	5,00E-05	0,00089885	yes
NPW	NPW	NPW	chr16:206952	shneg	shCHD1	OK	6,02829	1,56357	-1,94691	-4,76443	5,00E-05	0,00089885	yes
CFB	CFB	CFB	chr6_ssto_ha	shneg	shCHD1	OK	2,31284	0,599646	-1,94748	-5,13433	5,00E-05	0,00089885	yes
IFITM1	IFITM1	IFITM1	chr11:313990	shneg	shCHD1	OK	120,386	31,1724	-1,94932	-11,7271	5,00E-05	0,00089885	yes
PLAT	PLAT	PLAT	chr8:4203223	shneg	shCHD1	OK	32,8112	8,47843	-1,95232	-12,5459	5,00E-05	0,00089885	yes
CMPK2	CMPK2	CMPK2	chr2:6980683	shneg	shCHD1	OK	3,53318	0,907355	-1,96123	-4,65273	5,00E-05	0,00089885	yes
REG4	REG4	REG4	chr1:1203366	shneg	shCHD1	OK	92,0758	23,5957	-1,96429	-12,6134	5,00E-05	0,00089885	yes
KCNK12	KCNK12	KCNK12	chr2:4774791	shneg	shCHD1	OK	2,32936	0,587982	-1,98609	-3,56565	5,00E-05	0,00089885	yes
FRY	FRY	FRY	chr13:326054	shneg	shCHD1	OK	1,33323	0,328876	-2,01931	-8,01922	5,00E-05	0,00089885	yes
HCP5	HCP5	HCP5	chr6_ssto_ha	shneg	shCHD1	OK	1,87661	0,457105	-2,03753	-4,62695	5,00E-05	0,00089885	yes
IDO1	IDO1	IDO1	chr8:3977132	shneg	shCHD1	OK	11,5895	2,81724	-2,04046	-9,27214	5,00E-05	0,00089885	yes
RARRES3	RARRES3	RARRES3	chr11:630146	shneg	shCHD1	OK	15,7474	3,71789	-2,08256	-6,33893	5,00E-05	0,00089885	yes
MX1	MX1	MX1	chr21:427925	shneg	shCHD1	OK	16,0402	3,78629	-2,08283	-12,5908	5,00E-05	0,00089885	yes
TCEA3	TCEA3	TCEA3	chr1:2370755	shneg	shCHD1	OK	3,5568	0,838601	-2,08453	-4,96256	5,00E-05	0,00089885	yes
GBP1	GBP1	GBP1	chr1:8951798	shneg	shCHD1	OK	20,7526	4,73816	-2,1309	-12,6667	5,00E-05	0,00089885	yes
PSMB9	PSMB9	PSMB9	chr6_ssto_ha	shneg	shCHD1	OK	31,3594	6,7145	-2,22355	-10,9664	5,00E-05	0,00089885	yes
C1S	C1S	C1S	chr12:716797	shneg	shCHD1	OK	1,76516	0,344372	-2,35776	-4,64719	5,00E-05	0,00089885	yes
GBP2	GBP2	GBP2	chr1:8957330	shneg	shCHD1	OK	23,281	4,4621	-2,38336	-14,0445	5,00E-05	0,00089885	yes
GBP1P1	GBP1P1	GBP1P1	chr1:8987323	shneg	shCHD1	OK	1,79015	0,33072	-2,43639	-2,77785	0,00075	0,00894793	yes
RCAN2	RCAN2	RCAN2	chr6:4618846	shneg	shCHD1	OK	7,25246	1,23112	-2,5585	-10,6647	5,00E-05	0,00089885	yes
TRIM22	TRIM22	TRIM22	chr11:571081	shneg	shCHD1	OK	3,89119	0,648437	-2,58517	-7,47499	5,00E-05	0,00089885	yes
CD74	CD74	CD74	chr5:1497811	shneg	shCHD1	OK	1,768	0,283267	-2,64189	-2,82976	0,0001	0,00166847	yes
APOBEC3G	APOBEC3G	APOBEC3G	chr22:394730	shneg	shCHD1	OK	1,54749	0,237946	-2,70122	-3,87142	0,0002	0,00303812	yes
EPSTI1	EPSTI1	EPSTI1	chr13:434621	shneg	shCHD1	OK	1,20644	0,179559	-2,74822	-2,54751	0,0004	0,00541699	yes
GBP4	GBP4	GBP4	chr1:8964683	shneg	shCHD1	OK	6,68015	0,906813	-2,881	-14,2978	5,00E-05	0,00089885	yes
TUB	TUB	TUB	chr11:806017	shneg	shCHD1	OK	1,9492	0,235074	-3,05169	-8,08045	5,00E-05	0,00089885	yes
PROKR1	PROKR1	PROKR1	chr2:6887295	shneg	shCHD1	OK	0,795486	0,0897883	-3,14724	-1,79109	0,00525	0,0414208	yes
NNMT	NNMT	NNMT	chr11:114166	shneg	shCHD1	OK	3,45395	0,38687	-3,15832	-5,02454	5,00E-05	0,00089885	yes
