# Supplementary material for: CHD1 loss negatively influences metastasis-free survival in R0-resected prostate cancer patients and promotes spontaneous metastasis in vivo
Source: Cancer Gene Ther. 2021 Jan 7;29(1):49–61. doi: 10.1038/s41417-020-00288-z (PMC8761572; doi:10.1038/s41417-020-00288-z)
Supplement: Supplementary file 9 — Supplementary Material [file 41417_2020_288_MOESM9_ESM.pdf]

test_id	gene_id	gene	locus	sample_1	sample_2	status	value_1	value_2	log2(fold_cha	test_stat	p_value	q_value	significant
ANKRD30B	ANKRD30B	ANKRD30B	chr18:147482	shneg	shCHD1	OK	1,29585	0	#NAME?	#NAME?	5,00E-05	0,0004193	yes
MIR200A	MIR200A	MIR200A	chr1:1103242	shneg	shCHD1	OK	38,5493	0	#NAME?	#NAME?	0,00135	0,00748275	yes
OSTN	OSTN	OSTN	chr3:1909303	shneg	shCHD1	OK	2,69153	0	#NAME?	#NAME?	5,00E-05	0,0004193	yes
SELL	SELL	SELL	chr1:1696598	shneg	shCHD1	OK	0,0934067	3,87067	5,37291	4,82412	0,00035	0,00236888	yes
PDE4B	PDE4B	PDE4B	chr1:6625819	shneg	shCHD1	OK	0,0571313	2,10767	5,20522	4,36868	0,0001	0,00078473	yes
SPATA21	SPATA21	SPATA21	chr1:1672513	shneg	shCHD1	OK	0,184036	3,3301	4,1775	5,07617	5,00E-05	0,0004193	yes
PPP1R1A	PPP1R1A	PPP1R1A	chr12:549730	shneg	shCHD1	OK	0,182682	3,10856	4,08884	4,85728	5,00E-05	0,0004193	yes
SLCO2A1	SLCO2A1	SLCO2A1	chr3:1336515	shneg	shCHD1	OK	0,125547	1,40741	3,48674	5,82731	5,00E-05	0,0004193	yes
SUCNR1	SUCNR1	SUCNR1	chr3:1515914	shneg	shCHD1	OK	1,48257	16,5054	3,47677	10,8129	5,00E-05	0,0004193	yes
RYR2	RYR2	RYR2	chr1:2372057	shneg	shCHD1	OK	0,150402	1,57095	3,38474	11,0382	5,00E-05	0,0004193	yes
SGIP1	SGIP1	SGIP1	chr1:6699982	shneg	shCHD1	OK	1,08562	11,0128	3,34258	13,7186	5,00E-05	0,0004193	yes
GZMB	GZMB	GZMB	chr14:251001	shneg	shCHD1	OK	17,1512	172,996	3,33435	17,1676	5,00E-05	0,0004193	yes
PIK3C2G	PIK3C2G	PIK3C2G	chr12:184144	shneg	shCHD1	OK	0,177883	1,62054	3,18748	6,70491	5,00E-05	0,0004193	yes
USH1C	USH1C	USH1C	chr11:175154	shneg	shCHD1	OK	0,177898	1,59069	3,16053	4,10091	5,00E-05	0,0004193	yes
CDH26	CDH26	CDH26	chr20:585334	shneg	shCHD1	OK	0,233938	1,93813	3,05047	3,93507	5,00E-05	0,0004193	yes
RASGRF1	RASGRF1	RASGRF1	chr15:792522	shneg	shCHD1	OK	0,113956	0,942898	3,04863	4,59269	5,00E-05	0,0004193	yes
RLN1	RLN1	RLN1	chr9:5334968	shneg	shCHD1	OK	0,0996326	0,786799	2,9813	1,58077	0,00675	0,0286999	yes
NEB	NEB	NEB	chr2:1523418	shneg	shCHD1	OK	0,31643	2,3982	2,922	12,8171	5,00E-05	0,0004193	yes
DOC2B	DOC2B	DOC2B	chr17:6010-3:	shneg	shCHD1	OK	1,19684	8,80449	2,87901	6,7242	5,00E-05	0,0004193	yes
ATP8B1	ATP8B1	ATP8B1	chr18:552975	shneg	shCHD1	OK	0,470489	3,45322	2,87571	8,15337	5,00E-05	0,0004193	yes
EBI3	EBI3	EBI3	chr19:422953	shneg	shCHD1	OK	4,37974	31,7145	2,85622	11,048	5,00E-05	0,0004193	yes
BCL2	BCL2	BCL2	chr18:607905	shneg	shCHD1	OK	0,275023	1,94081	2,81903	2,71525	0,0009	0,00533451	yes
ATP6V0A4	ATP6V0A4	ATP6V0A4	chr7:1383910	shneg	shCHD1	OK	0,539368	3,62341	2,74801	6,7219	5,00E-05	0,0004193	yes
OLR1	OLR1	OLR1	chr12:103108	shneg	shCHD1	OK	0,259462	1,69394	2,70678	4,31696	5,00E-05	0,0004193	yes
LIX1	LIX1	LIX1	chr5:9642757	shneg	shCHD1	OK	0,377659	2,44552	2,69499	7,15811	5,00E-05	0,0004193	yes
CA14	CA14	CA14	chr1:1502302	shneg	shCHD1	OK	1,92438	12,4377	2,69225	9,26988	5,00E-05	0,0004193	yes
SLC19A2	SLC19A2	SLC19A2	chr1:1694331	shneg	shCHD1	OK	4,15499	26,5303	2,67472	13,5905	5,00E-05	0,0004193	yes
SDPR	SDPR	SDPR	chr2:1926990	shneg	shCHD1	OK	0,175663	1,07759	2,61692	4,01121	5,00E-05	0,0004193	yes
C6orf58	C6orf58	C6orf58	chr6:1278983	shneg	shCHD1	OK	0,64271	3,85758	2,58545	4,47817	5,00E-05	0,0004193	yes
LOC10050580	LOC10050580	LOC10050580	chr5:9546311	shneg	shCHD1	OK	0,187978	1,11974	2,57453	2,66415	0,0019	0,00996052	yes
TNFRSF19	TNFRSF19	TNFRSF19	chr13:241445	shneg	shCHD1	OK	0,972476	5,5103	2,5024	7,72203	5,00E-05	0,0004193	yes
KLRC4	KLRC4	KLRC4	chr12:105249	shneg	shCHD1	OK	0,36184	2,04772	2,50059	2,13258	0,006	0,0260761	yes
GAL	GAL	GAL	chr11:684519	shneg	shCHD1	OK	0,7946	4,44037	2,48238	3,48059	5,00E-05	0,0004193	yes
UNC5A	UNC5A	UNC5A	chr5:1762375	shneg	shCHD1	OK	7,3897	40,5923	2,45762	12,877	5,00E-05	0,0004193	yes
TSLP	TSLP	TSLP	chr5:1104057	shneg	shCHD1	OK	0,510963	2,80212	2,45523	5,56846	5,00E-05	0,0004193	yes
ARHGDIB	ARHGDIB	ARHGDIB	chr12:150949	shneg	shCHD1	OK	0,224723	1,14013	2,34298	2,26231	0,0024	0,0121606	yes
CD82	CD82	CD82	chr11:445871	shneg	shCHD1	OK	15,0618	75,6841	2,32909	12,0083	5,00E-05	0,0004193	yes
RLN2	RLN2	RLN2	chr9:5299867	shneg	shCHD1	OK	1,08406	5,40665	2,31828	3,49296	5,00E-05	0,0004193	yes
FA2H	FA2H	FA2H	chr16:747468	shneg	shCHD1	OK	1,78923	8,84436	2,30542	8,68544	5,00E-05	0,0004193	yes
CYP3A7	CYP3A7	CYP3A7	chr7:9928230	shneg	shCHD1	OK	17,4027	85,1764	2,29114	12,0341	5,00E-05	0,0004193	yes
IGF2BP1	IGF2BP1	IGF2BP1	chr17:470747	shneg	shCHD1	OK	2,77549	13,1952	2,2492	11,7142	5,00E-05	0,0004193	yes
MKX	MKX	MKX	chr10:279618	shneg	shCHD1	OK	0,279723	1,30165	2,21826	4,81694	5,00E-05	0,0004193	yes
KLRC2	KLRC2	KLRC2	chr12:105832	shneg	shCHD1	OK	0,514029	2,37096	2,20555	3,37325	5,00E-05	0,0004193	yes
ELF3	ELF3	ELF3	chr1:2019796	shneg	shCHD1	OK	0,765475	3,50491	2,19495	6,80313	5,00E-05	0,0004193	yes
ITM2A	ITM2A	ITM2A	chrX:7861588	shneg	shCHD1	OK	0,352162	1,60291	2,18638	2,85257	5,00E-05	0,0004193	yes
FOXN4	FOXN4	FOXN4	chr12:109715	shneg	shCHD1	OK	2,2189	9,90432	2,15821	9,51564	5,00E-05	0,0004193	yes
SGK223	SGK223	SGK223	chr8:8175257	shneg	shCHD1	OK	0,991586	4,39021	2,14648	8,31311	5,00E-05	0,0004193	yes
TNF	TNF	TNF	chr6_ssto_ha	shneg	shCHD1	OK	15,4061	68,0923	2,14399	11,0003	5,00E-05	0,0004193	yes
CLMP	CLMP	CLMP	chr11:122943	shneg	shCHD1	OK	0,53052	2,33826	2,13996	5,28045	5,00E-05	0,0004193	yes
CCNJL	CCNJL	CCNJL	chr5:1596786	shneg	shCHD1	OK	1,07664	4,58325	2,08984	7,4909	5,00E-05	0,0004193	yes
SEMA5B	SEMA5B	SEMA5B	chr3:1226280	shneg	shCHD1	OK	0,236764	0,997488	2,07485	3,98649	5,00E-05	0,0004193	yes
GABRD	GABRD	GABRD	chr1:1950767	shneg	shCHD1	OK	1,19487	5,02597	2,07255	6,20508	5,00E-05	0,0004193	yes
DOCK2	DOCK2	DOCK2	chr5:1690642	shneg	shCHD1	OK	0,694927	2,88966	2,05597	7,53855	5,00E-05	0,0004193	yes
CRH	CRH	CRH	chr8:6708861	shneg	shCHD1	OK	6,67919	27,5175	2,04261	9,37005	5,00E-05	0,0004193	yes
MYBPH	MYBPH	MYBPH	chr1:2031369	shneg	shCHD1	OK	2,05986	8,423	2,03179	7,21255	5,00E-05	0,0004193	yes
BCL2A1	BCL2A1	BCL2A1	chr15:802532	shneg	shCHD1	OK	1,84755	7,49929	2,02115	4,89039	5,00E-05	0,0004193	yes
EPHA4	EPHA4	EPHA4	chr2:2222827	shneg	shCHD1	OK	2,71474	10,8579	1,99986	10,0979	5,00E-05	0,0004193	yes
TMEM27	TMEM27	TMEM27	chrX:1564544	shneg	shCHD1	OK	1,26516	5,00444	1,98389	5,47682	5,00E-05	0,0004193	yes
SLC1A1	SLC1A1	SLC1A1	chr9:4490426	shneg	shCHD1	OK	2,1295	8,32594	1,96709	8,80189	5,00E-05	0,0004193	yes
NOX3	NOX3	NOX3	chr6:1557165	shneg	shCHD1	OK	1,18345	4,59107	1,95583	5,96896	5,00E-05	0,0004193	yes
CHI3L1	CHI3L1	CHI3L1	chr1:2031480	shneg	shCHD1	OK	0,355508	1,36855	1,9447	3,32578	0,00015	0,00112331	yes
Sep 10	Sep 10	Sep 10	chr2:1103003	shneg	shCHD1	OK	1,48448	5,61477	1,91927	7,45988	5,00E-05	0,0004193	yes
ASB2	ASB2	ASB2	chr14:944004	shneg	shCHD1	OK	1,56185	5,86077	1,90784	7,27866	5,00E-05	0,0004193	yes
IQGAP2	IQGAP2	IQGAP2	chr5:7569914	shneg	shCHD1	OK	0,244525	0,913526	1,90146	4,06875	5,00E-05	0,0004193	yes
ZNF488	ZNF488	ZNF488	chr10:483550	shneg	shCHD1	OK	1,03744	3,85741	1,89461	6,87989	5,00E-05	0,0004193	yes
AUTS2	AUTS2	AUTS2	chr7:6906390	shneg	shCHD1	OK	0,543659	2,01663	1,89117	6,1922	5,00E-05	0,0004193	yes
FGF20	FGF20	FGF20	chr8:1685033	shneg	shCHD1	OK	1,78434	6,59259	1,88546	4,80424	5,00E-05	0,0004193	yes
RNF145	RNF145	RNF145	chr5:1585844	shneg	shCHD1	OK	23,2656	85,5361	1,87833	10,4363	5,00E-05	0,0004193	yes
SKAP1	SKAP1	SKAP1	chr17:462108	shneg	shCHD1	OK	2,06994	7,50792	1,85882	6,2169	5,00E-05	0,0004193	yes
NPTX2	NPTX2	NPTX2	chr7:9824659	shneg	shCHD1	OK	2,20849	7,90813	1,84028	7,54315	5,00E-05	0,0004193	yes
KLRC3	KLRC3	KLRC3	chr12:105649	shneg	shCHD1	OK	0,571899	2,04646	1,8393	2,47088	0,0004	0,00266202	yes
AOC2	AOC2	AOC2	chr17:409966	shneg	shCHD1	OK	0,498144	1,77439	1,83269	4,1519	5,00E-05	0,0004193	yes
RGS9	RGS9	RGS9	chr17:631334	shneg	shCHD1	OK	0,56517	1,99429	1,81912	3,30157	5,00E-05	0,0004193	yes
PROK2	PROK2	PROK2	chr3:7182080	shneg	shCHD1	OK	4,99443	17,519	1,81053	7,8424	5,00E-05	0,0004193	yes
LINGO1	LINGO1	LINGO1	chr15:779053	shneg	shCHD1	OK	0,419963	1,47289	1,81032	4,13201	5,00E-05	0,0004193	yes
ABLIM3	ABLIM3	ABLIM3	chr5:1485210	shneg	shCHD1	OK	0,276303	0,968316	1,80923	4,11948	5,00E-05	0,0004193	yes
DNAH12	DNAH12	DNAH12	chr3:5732772	shneg	shCHD1	OK	0,347512	1,20089	1,78896	5,00202	5,00E-05	0,0004193	yes
MMP7	MMP7	MMP7	chr11:102391	shneg	shCHD1	OK	154,262	530,73	1,78259	10,3174	5,00E-05	0,0004193	yes
HEG1	HEG1	HEG1	chr3:1246845	shneg	shCHD1	OK	1,20368	4,0998	1,7681	8,55283	5,00E-05	0,0004193	yes
ADCYAP1	ADCYAP1	ADCYAP1	chr18:904943	shneg	shCHD1	OK	1,14399	3,88094	1,76233	6,29646	5,00E-05	0,0004193	yes
ACE2	ACE2	ACE2	chrX:1557915	shneg	shCHD1	OK	3,08171	10,4073	1,75579	8,35029	5,00E-05	0,0004193	yes
KRT17	KRT17	KRT17	chr17:397756	shneg	shCHD1	OK	3,90552	13,1584	1,7524	7,09125	5,00E-05	0,0004193	yes
SNCG	SNCG	SNCG	chr10:887182	shneg	shCHD1	OK	2,15771	7,22104	1,7427	4,27423	5,00E-05	0,0004193	yes
CLEC4E	CLEC4E	CLEC4E	chr12:868590	shneg	shCHD1	OK	0,590732	1,97693	1,74269	3,99138	5,00E-05	0,0004193	yes
TGFA	TGFA	TGFA	chr2:7067441	shneg	shCHD1	OK	10,8952	36,2993	1,73625	9,38679	5,00E-05	0,0004193	yes
HDAC9	HDAC9	HDAC9	chr7:1812657	shneg	shCHD1	OK	0,58648	1,93564	1,72266	4,90777	5,00E-05	0,0004193	yes
TMEM37	TMEM37	TMEM37	chr2:1201894	shneg	shCHD1	OK	0,258363	0,849176	1,71666	2,09894	0,004	0,018603	yes
PDE9A	PDE9A	PDE9A	chr21:440738	shneg	shCHD1	OK	1,49246	4,89985	1,71505	4,49052	5,00E-05	0,0004193	yes
OSR1	OSR1	OSR1	chr2:1955124	shneg	shCHD1	OK	0,94528	3,09863	1,71282	4,5252	5,00E-05	0,0004193	yes
HAPLN1	HAPLN1	HAPLN1	chr5:8293401	shneg	shCHD1	OK	2,7435	8,93191	1,70295	8,45329	5,00E-05	0,0004193	yes
C12orf59	C12orf59	C12orf59	chr12:103315	shneg	shCHD1	OK	1,50851	4,91019	1,70266	6,41635	5,00E-05	0,0004193	yes
BTNL9	BTNL9	BTNL9	chr5:1804672	shneg	shCHD1	OK	0,518131	1,6839	1,70042	4,59613	5,00E-05	0,0004193	yes

MGC39372	MGC39372	MGC39372	chr6:2854890	shneg	shCHD1	OK	3,55468	11,4657	1,68953	6,23476	5,00E-05	0,0004193	yes
TLE3	TLE3	TLE3	chr15:703405	shneg	shCHD1	OK	0,909141	2,93053	1,68858	6,61215	5,00E-05	0,0004193	yes
PXDC1	PXDC1	PXDC1	chr6:3722835	shneg	shCHD1	OK	1,30155	4,12125	1,66285	5,31621	5,00E-05	0,0004193	yes
CXCR4	CXCR4	CXCR4	chr2:1368719	shneg	shCHD1	OK	4,52133	14,3125	1,66245	7,14794	5,00E-05	0,0004193	yes
SCARF1	SCARF1	SCARF1	chr17:153715	shneg	shCHD1	OK	4,60484	14,4954	1,65438	8,25403	5,00E-05	0,0004193	yes
CX3CL1	CX3CL1	CX3CL1	chr16:574064	shneg	shCHD1	OK	2,18057	6,83059	1,64731	7,07791	5,00E-05	0,0004193	yes
STYK1	STYK1	STYK1	chr12:107715	shneg	shCHD1	OK	0,583818	1,79943	1,62395	4,26169	5,00E-05	0,0004193	yes
TGM2	TGM2	TGM2	chr20:367568	shneg	shCHD1	OK	1,60564	4,87015	1,60082	5,74919	5,00E-05	0,0004193	yes
LEMD1	LEMD1	LEMD1	chr1:2053423	shneg	shCHD1	OK	3,72721	11,2986	1,59998	3,5847	5,00E-05	0,0004193	yes
IL37	IL37	IL37	chr2:1136705	shneg	shCHD1	OK	1,13695	3,44621	1,59984	2,09462	0,00055	0,00350455	yes
CYP26A1	CYP26A1	CYP26A1	chr10:948332	shneg	shCHD1	OK	0,609867	1,84121	1,59409	3,57519	5,00E-05	0,0004193	yes
LINC00052	LINC00052	LINC00052	chr15:881201	shneg	shCHD1	OK	1,60754	4,8038	1,57932	4,99247	5,00E-05	0,0004193	yes
TFPI2	TFPI2	TFPI2	chr7:9351574	shneg	shCHD1	OK	27,5261	82,1306	1,57712	8,19513	5,00E-05	0,0004193	yes
GPX7	GPX7	GPX7	chr1:5306804	shneg	shCHD1	OK	1,33357	3,96145	1,57073	3,83016	5,00E-05	0,0004193	yes
PKDCC	PKDCC	PKDCC	chr2:4227516	shneg	shCHD1	OK	6,96266	20,5282	1,5599	7,74213	5,00E-05	0,0004193	yes
CAMK2N1	CAMK2N1	CAMK2N1	chr1:2080888	shneg	shCHD1	OK	6,1168	17,9863	1,55605	7,78751	5,00E-05	0,0004193	yes
IL4I1	IL4I1	IL4I1	chr19:503929	shneg	shCHD1	OK	13,1207	38,259	1,54396	3,40995	5,00E-05	0,0004193	yes
NGFR	NGFR	NGFR	chr17:475726	shneg	shCHD1	OK	2,8441	8,24518	1,53558	7,10388	5,00E-05	0,0004193	yes
SCG2	SCG2	SCG2	chr2:2244616	shneg	shCHD1	OK	0,945846	2,74114	1,5351	4,59597	5,00E-05	0,0004193	yes
GCNT3	GCNT3	GCNT3	chr15:599039	shneg	shCHD1	OK	1,90074	5,49124	1,53057	5,65799	5,00E-05	0,0004193	yes
CCR6	CCR6	CCR6	chr6:1675252	shneg	shCHD1	OK	0,371068	1,05692	1,51011	3,29407	5,00E-05	0,0004193	yes
MGLL	MGLL	MGLL	chr3:1274079	shneg	shCHD1	OK	0,594395	1,68596	1,50408	4,54039	5,00E-05	0,0004193	yes
CLDN2	CLDN2	CLDN2	chrX:1061432	shneg	shCHD1	OK	30,608	86,5464	1,49957	8,48117	5,00E-05	0,0004193	yes
SYT17	SYT17	SYT17	chr16:191796	shneg	shCHD1	OK	1,45265	4,06307	1,48388	4,6009	5,00E-05	0,0004193	yes
CALCR	CALCR	CALCR	chr7:9305379	shneg	shCHD1	OK	1,80179	4,99803	1,47193	6,11351	5,00E-05	0,0004193	yes
SPATA13	SPATA13	SPATA13	chr13:247348	shneg	shCHD1	OK	3,37549	9,3419	1,46862	7,43054	5,00E-05	0,0004193	yes
LOC284100	LOC284100	LOC284100	chr17:362025	shneg	shCHD1	OK	0,388533	1,07008	1,4616	2,85865	5,00E-05	0,0004193	yes
ALCAM	ALCAM	ALCAM	chr3:1050855	shneg	shCHD1	OK	9,91844	27,2862	1,45999	7,84753	5,00E-05	0,0004193	yes
HES1	HES1	HES1	chr3:1938539	shneg	shCHD1	OK	3,82364	10,5129	1,45914	5,72022	5,00E-05	0,0004193	yes
IL1A	IL1A	IL1A	chr2:1135314	shneg	shCHD1	OK	12,4589	34,2183	1,4576	7,68797	5,00E-05	0,0004193	yes
C2CD4A	C2CD4A	C2CD4A	chr15:623591	shneg	shCHD1	OK	1,42105	3,89908	1,45618	5,73079	5,00E-05	0,0004193	yes
CDC6	CDC6	CDC6	chr17:384441	shneg	shCHD1	OK	13,7385	37,6523	1,45452	7,78079	5,00E-05	0,0004193	yes
NTN1	NTN1	NTN1	chr17:892485	shneg	shCHD1	OK	0,728606	1,98635	1,44691	5,48568	5,00E-05	0,0004193	yes
FAM81A	FAM81A	FAM81A	chr15:597303	shneg	shCHD1	OK	1,6073	4,37555	1,44483	5,8405	5,00E-05	0,0004193	yes
IL32	IL32	IL32	chr16:311531	shneg	shCHD1	OK	35,7422	97,1887	1,44316	7,34796	5,00E-05	0,0004193	yes
CTSS	CTSS	CTSS	chr1:1507026	shneg	shCHD1	OK	2,12013	5,65367	1,41503	6,35261	5,00E-05	0,0004193	yes
COL13A1	COL13A1	COL13A1	chr10:715616	shneg	shCHD1	OK	0,586856	1,54355	1,39517	3,09229	5,00E-05	0,0004193	yes
RIBC2	RIBC2	RIBC2	chr22:458095	shneg	shCHD1	OK	1,1648	3,05228	1,38981	3,33509	5,00E-05	0,0004193	yes
PODXL	PODXL	PODXL	chr7:1311850	shneg	shCHD1	OK	1,63011	4,25257	1,38337	6,4073	5,00E-05	0,0004193	yes
SERPINB8	SERPINB8	SERPINB8	chr18:616372	shneg	shCHD1	OK	1,17264	3,05251	1,38024	4,44435	5,00E-05	0,0004193	yes
SCN4B	SCN4B	SCN4B	chr11:118004	shneg	shCHD1	OK	0,383839	0,997308	1,37754	3,26496	5,00E-05	0,0004193	yes
PHYHIPL	PHYHIPL	PHYHIPL	chr10:609363	shneg	shCHD1	OK	3,46005	8,96162	1,37297	5,85253	5,00E-05	0,0004193	yes
WWC1	WWC1	WWC1	chr5:1677190	shneg	shCHD1	OK	7,11561	18,4029	1,37087	7,41593	5,00E-05	0,0004193	yes
RUNX3	RUNX3	RUNX3	chr1:2522600	shneg	shCHD1	OK	0,73754	1,90169	1,36649	4,49992	5,00E-05	0,0004193	yes
JDP2	JDP2	JDP2	chr14:758945	shneg	shCHD1	OK	0,49834	1,25997	1,33818	3,37191	5,00E-05	0,0004193	yes
C3orf52	C3orf52	C3orf52	chr3:1118051	shneg	shCHD1	OK	0,375482	0,944799	1,33126	2,28135	0,00035	0,00236888	yes
PDE2A	PDE2A	PDE2A	chr11:722871	shneg	shCHD1	OK	0,573672	1,44327	1,33105	3,80001	5,00E-05	0,0004193	yes
KIAA1217	KIAA1217	KIAA1217	chr10:239836	shneg	shCHD1	OK	0,386296	0,967382	1,32438	3,99595	5,00E-05	0,0004193	yes
FAM71F2	FAM71F2	FAM71F2	chr7:1283123	shneg	shCHD1	OK	1,2554	3,13898	1,32215	2,66984	5,00E-05	0,0004193	yes
IGFBP4	IGFBP4	IGFBP4	chr17:385996	shneg	shCHD1	OK	5,3674	13,3851	1,31833	6,30215	5,00E-05	0,0004193	yes
MDGA2	MDGA2	MDGA2	chr14:473088	shneg	shCHD1	OK	1,40329	3,48252	1,31131	5,56552	5,00E-05	0,0004193	yes
CELF5	CELF5	CELF5	chr19:322470	shneg	shCHD1	OK	0,757183	1,86116	1,29749	3,18048	5,00E-05	0,0004193	yes
LRRN2	LRRN2	LRRN2	chr1:2045863	shneg	shCHD1	OK	0,380337	0,929062	1,2885	2,82669	5,00E-05	0,0004193	yes
CCNE2	CCNE2	CCNE2	chr8:9583553	shneg	shCHD1	OK	2,19733	5,35402	1,28487	2,89221	5,00E-05	0,0004193	yes
TRIM55	TRIM55	TRIM55	chr8:6703927	shneg	shCHD1	OK	3,63709	8,84628	1,28228	5,34119	5,00E-05	0,0004193	yes
GLDC	GLDC	GLDC	chr9:6532463	shneg	shCHD1	OK	0,830806	2,01321	1,27691	4,19228	5,00E-05	0,0004193	yes
GDF15	GDF15	GDF15	chr19:184969	shneg	shCHD1	OK	98,4864	238,385	1,2753	7,14338	5,00E-05	0,0004193	yes
LINC00152	LINC00152	LINC00152	chr2:8775497	shneg	shCHD1	OK	10,4797	25,1188	1,26117	4,25579	5,00E-05	0,0004193	yes
ASRGL1	ASRGL1	ASRGL1	chr11:621047	shneg	shCHD1	OK	7,56248	18,0519	1,25522	6,20866	5,00E-05	0,0004193	yes
ONECUT2	ONECUT2	ONECUT2	chr18:551029	shneg	shCHD1	OK	0,83067	1,96708	1,24371	6,1692	5,00E-05	0,0004193	yes
BIRC3	BIRC3	BIRC3	chr11:102188	shneg	shCHD1	OK	3,7028	8,7302	1,2374	5,35532	5,00E-05	0,0004193	yes
SYT7	SYT7	SYT7	chr11:612811	shneg	shCHD1	OK	0,78749	1,85225	1,23395	4,79468	5,00E-05	0,0004193	yes
HTR6	HTR6	HTR6	chr1:1999177	shneg	shCHD1	OK	0,44795	1,05069	1,22992	2,30841	0,00075	0,00455593	yes
LOC285972	LOC285972	LOC285972	chr7:1501307	shneg	shCHD1	OK	1,19077	2,7858	1,2262	3,56254	5,00E-05	0,0004193	yes
PXDN	PXDN	PXDN	chr2:1635658	shneg	shCHD1	OK	1,20092	2,79886	1,2207	5,36751	5,00E-05	0,0004193	yes
CDHR2	CDHR2	CDHR2	chr5:1759695	shneg	shCHD1	OK	0,422956	0,983467	1,21737	3,15444	5,00E-05	0,0004193	yes
MBOAT1	MBOAT1	MBOAT1	chr6:2010093	shneg	shCHD1	OK	1,24359	2,88889	1,216	4,39814	5,00E-05	0,0004193	yes
MMP1	MMP1	MMP1	chr11:102654	shneg	shCHD1	OK	3720,09	8625,88	1,21333	5,15413	5,00E-05	0,0004193	yes
PCBP3	PCBP3	PCBP3	chr21:472698	shneg	shCHD1	OK	3,11744	7,21565	1,21077	5,00078	5,00E-05	0,0004193	yes
C1QTNF1	C1QTNF1	C1QTNF1	chr17:770152	shneg	shCHD1	OK	0,497814	1,15142	1,20974	2,63755	5,00E-05	0,0004193	yes
TNFAIP3	TNFAIP3	TNFAIP3	chr6:1381885	shneg	shCHD1	OK	0,97805	2,25789	1,207	4,48246	5,00E-05	0,0004193	yes
N4BP3	N4BP3	N4BP3	chr5:1775405	shneg	shCHD1	OK	1,23855	2,85009	1,20236	5,24686	5,00E-05	0,0004193	yes
CALCRL	CALCRL	CALCRL	chr2:1882078	shneg	shCHD1	OK	4,60586	10,5841	1,20035	6,0069	5,00E-05	0,0004193	yes
JAG1	JAG1	JAG1	chr20:106183	shneg	shCHD1	OK	10,9254	24,9273	1,19004	6,55757	5,00E-05	0,0004193	yes
RAP2A	RAP2A	RAP2A	chr13:980864	shneg	shCHD1	OK	7,78603	17,7422	1,18823	6,22012	5,00E-05	0,0004193	yes
SEL1L3	SEL1L3	SEL1L3	chr4:2574904	shneg	shCHD1	OK	1,12157	2,54452	1,18187	4,6524	5,00E-05	0,0004193	yes
ELOVL5	ELOVL5	ELOVL5	chr6:5313219	shneg	shCHD1	OK	1,66214	3,7653	1,17973	3,59538	5,00E-05	0,0004193	yes
FGGY	FGGY	FGGY	chr1:5976262	shneg	shCHD1	OK	12,6056	28,3962	1,17163	5,77865	5,00E-05	0,0004193	yes
XKR7	XKR7	XKR7	chr20:305558	shneg	shCHD1	OK	4,635	10,4395	1,17141	5,73324	5,00E-05	0,0004193	yes
MSRB3	MSRB3	MSRB3	chr12:656724	shneg	shCHD1	OK	20,6736	46,5122	1,16982	6,51736	5,00E-05	0,0004193	yes
MPP3	MPP3	MPP3	chr17:418781	shneg	shCHD1	OK	5,18799	11,6415	1,16603	5,81461	5,00E-05	0,0004193	yes
TMEM173	TMEM173	TMEM173	chr5:1388551	shneg	shCHD1	OK	85,8273	191,73	1,15957	6,71081	5,00E-05	0,0004193	yes
TMEM132A	TMEM132A	TMEM132A	chr11:606919	shneg	shCHD1	OK	25,5222	56,8006	1,15415	6,29229	5,00E-05	0,0004193	yes
PLA2G4C	PLA2G4C	PLA2G4C	chr19:485510	shneg	shCHD1	OK	1,55484	3,45038	1,14999	3,99415	5,00E-05	0,0004193	yes
MID1	MID1	MID1	chrX:1041334	shneg	shCHD1	OK	1,81405	4,02423	1,1495	4,03362	5,00E-05	0,0004193	yes
HRH3	HRH3	HRH3	chr20:607900	shneg	shCHD1	OK	0,343216	0,760762	1,14833	2,18504	0,001	0,0058183	yes
ADORA1	ADORA1	ADORA1	chr1:2030968	shneg	shCHD1	OK	6,75759	14,9659	1,1471	5,59813	5,00E-05	0,0004193	yes
VNN1	VNN1	VNN1	chr6:1330019	shneg	shCHD1	OK	6,29329	13,9119	1,14443	5,79345	5,00E-05	0,0004193	yes
TTC25	TTC25	TTC25	chr17:400868	shneg	shCHD1	OK	1,53496	3,3879	1,14219	3,65252	5,00E-05	0,0004193	yes
MSI1	MSI1	MSI1	chr12:120779	shneg	shCHD1	OK	1,49415	3,28562	1,13685	4,19927	5,00E-05	0,0004193	yes
BCRP3	BCRP3	BCRP3	chr22:250288	shneg	shCHD1	OK	4,06135	8,90716	1,13301	3,77088	5,00E-05	0,0004193	yes
RGS2	RGS2	RGS2	chr1:1927781	shneg	shCHD1	OK	129,439	283,174	1,12942	6,4927	5,00E-05	0,0004193	yes
KCNE3	KCNE3	KCNE3	chr11:741658	shneg	shCHD1	OK	2,03967	4,44216	1,12293	4,67832	5,00E-05	0,0004193	yes

ADA	ADA	ADA	chr20:432481	shneg	shCHD1	OK	8,70792	18,9474	1,1216	5,38765	5,00E-05	0,0004193	yes
VANGL2	VANGL2	VANGL2	chr1:1603703	shneg	shCHD1	OK	1,77027	3,85042	1,12105	5,08452	5,00E-05	0,0004193	yes
TERT	TERT	TERT	chr5:1253286	shneg	shCHD1	OK	0,348863	0,758745	1,12095	2,33597	0,0001	0,00078473	yes
KCNH4	KCNH4	KCNH4	chr17:403089	shneg	shCHD1	OK	1,5446	3,3532	1,1183	4,63769	5,00E-05	0,0004193	yes
RUNX2	RUNX2	RUNX2	chr6:4479646	shneg	shCHD1	OK	0,478741	1,03312	1,10969	2,80515	5,00E-05	0,0004193	yes
IGF2	IGF2	IGF2	chr11:215034	shneg	shCHD1	OK	1,66413	3,59029	1,10933	4,20994	5,00E-05	0,0004193	yes
VAV3	VAV3	VAV3	chr1:1081137	shneg	shCHD1	OK	2,47105	5,3306	1,10917	4,83821	5,00E-05	0,0004193	yes
RAB3C	RAB3C	RAB3C	chr5:5787893	shneg	shCHD1	OK	2,27577	4,87422	1,09882	2,92343	5,00E-05	0,0004193	yes
PHLDA2	PHLDA2	PHLDA2	chr11:294950	shneg	shCHD1	OK	37,7901	80,8902	1,09796	5,60816	5,00E-05	0,0004193	yes
CAMK4	CAMK4	CAMK4	chr5:1105599	shneg	shCHD1	OK	0,73504	1,57254	1,0972	2,68262	5,00E-05	0,0004193	yes
CEACAM1	CEACAM1	CEACAM1	chr19:430114	shneg	shCHD1	OK	2,88978	6,14478	1,0884	4,92245	5,00E-05	0,0004193	yes
KLF9	KLF9	KLF9	chr9:7299951	shneg	shCHD1	OK	1,66089	3,52745	1,08667	4,81696	5,00E-05	0,0004193	yes
ROBO4	ROBO4	ROBO4	chr11:124754	shneg	shCHD1	OK	15,7657	33,4431	1,08492	5,922	5,00E-05	0,0004193	yes
SAMD4A	SAMD4A	SAMD4A	chr14:550343	shneg	shCHD1	OK	3,44744	7,31259	1,08486	5,40708	5,00E-05	0,0004193	yes
DSP	DSP	DSP	chr6:7541869	shneg	shCHD1	OK	10,1439	21,3683	1,07486	5,92376	5,00E-05	0,0004193	yes
KLK10	KLK10	KLK10	chr19:515159	shneg	shCHD1	OK	1,41643	2,97281	1,06956	3,78531	5,00E-05	0,0004193	yes
OLFM2	OLFM2	OLFM2	chr19:996439	shneg	shCHD1	OK	2,58429	5,42261	1,06922	3,98463	5,00E-05	0,0004193	yes
TMEM99	TMEM99	TMEM99	chr17:389743	shneg	shCHD1	OK	3,31237	6,94637	1,0684	2,37102	0,00035	0,00236888	yes
MIR7-3HG	MIR7-3HG	MIR7-3HG	chr19:476911	shneg	shCHD1	OK	1,15838	2,42449	1,06557	1,7361	0,0037	0,0174466	yes
NKAIN1	NKAIN1	NKAIN1	chr1:3165259	shneg	shCHD1	OK	3,09941	6,44979	1,05726	4,69292	5,00E-05	0,0004193	yes
MMP20	MMP20	MMP20	chr11:102447	shneg	shCHD1	OK	0,437963	0,906618	1,04968	1,87705	0,00365	0,0172576	yes
RHOB	RHOB	RHOB	chr2:2064683	shneg	shCHD1	OK	32,8536	67,8133	1,04552	5,77812	5,00E-05	0,0004193	yes
CABP1	CABP1	CABP1	chr12:121078	shneg	shCHD1	OK	3,86175	7,94683	1,04112	3,26429	5,00E-05	0,0004193	yes
HSPA2	HSPA2	HSPA2	chr14:650071	shneg	shCHD1	OK	0,81278	1,6722	1,04081	2,92061	5,00E-05	0,0004193	yes
NFASC	NFASC	NFASC	chr1:2047977	shneg	shCHD1	OK	1,17107	2,40104	1,03583	3,14489	5,00E-05	0,0004193	yes
KCP	KCP	KCP	chr7:1285169	shneg	shCHD1	OK	1,503	3,08061	1,03537	3,66089	5,00E-05	0,0004193	yes
ARHGEF4	ARHGEF4	ARHGEF4	chr2:1316742	shneg	shCHD1	OK	0,618423	1,26402	1,03135	2,95727	5,00E-05	0,0004193	yes
AQP1	AQP1	AQP1	chr7:3095141	shneg	shCHD1	OK	1,06164	2,1693	1,03093	2,96596	5,00E-05	0,0004193	yes
SGK1	SGK1	SGK1	chr6:1344903	shneg	shCHD1	OK	65,4698	133,415	1,02702	5,894	5,00E-05	0,0004193	yes
PGBD5	PGBD5	PGBD5	chr1:2304573	shneg	shCHD1	OK	4,56423	9,28909	1,02517	5,07462	5,00E-05	0,0004193	yes
CISD1	CISD1	CISD1	chr10:600288	shneg	shCHD1	OK	14,9905	30,4855	1,02408	5,27424	5,00E-05	0,0004193	yes
PHKG1	PHKG1	PHKG1	chr7:5614867	shneg	shCHD1	OK	0,735242	1,49438	1,02326	1,9696	0,00235	0,0119595	yes
BAMBI	BAMBI	BAMBI	chr10:289664	shneg	shCHD1	OK	0,694831	1,412	1,023	2,12887	0,0011	0,0063157	yes
DPYSL2	DPYSL2	DPYSL2	chr8:2637170	shneg	shCHD1	OK	2,68217	5,4453	1,02161	4,84084	5,00E-05	0,0004193	yes
BRCA1	BRCA1	BRCA1	chr17:411963	shneg	shCHD1	OK	10,5396	21,3662	1,01951	5,00675	5,00E-05	0,0004193	yes
FZD3	FZD3	FZD3	chr8:2835172	shneg	shCHD1	OK	0,849626	1,71769	1,01557	4,84392	5,00E-05	0,0004193	yes
C15orf48	C15orf48	C15orf48	chr15:457227	shneg	shCHD1	OK	3,31891	6,7092	1,01543	2,77553	5,00E-05	0,0004193	yes
THEG	THEG	THEG	chr19:362056	shneg	shCHD1	OK	3,80519	7,68547	1,01417	3,68384	5,00E-05	0,0004193	yes
VIP	VIP	VIP	chr6:1530719	shneg	shCHD1	OK	48,0373	96,564	1,00733	5,5493	5,00E-05	0,0004193	yes
THBS1	THBS1	THBS1	chr15:398732	shneg	shCHD1	OK	4,7303	9,5077	1,00716	5,16296	5,00E-05	0,0004193	yes
PILRA	PILRA	PILRA	chr7:9997106	shneg	shCHD1	OK	1,19467	2,40012	1,0065	2,06735	0,00045	0,00293585	yes
YTHDC2	YTHDC2	YTHDC2	chr5:1128493	shneg	shCHD1	OK	13,1526	26,4088	1,00567	5,57846	5,00E-05	0,0004193	yes
GHRLOS2	GHRLOS2	GHRLOS2	chr3:1032610	shneg	shCHD1	OK	0,589152	1,18129	1,00365	1,61602	0,00565	0,0248732	yes
SYNPO	SYNPO	SYNPO	chr5:1499806	shneg	shCHD1	OK	1,24434	2,48735	0,999233	4,21113	5,00E-05	0,0004193	yes
TRIM16L	TRIM16L	TRIM16L	chr17:186254	shneg	shCHD1	OK	3,60102	7,19084	0,997755	4,25468	5,00E-05	0,0004193	yes
HK2	HK2	HK2	chr2:7505978	shneg	shCHD1	OK	1,50026	2,9951	0,997392	4,62435	5,00E-05	0,0004193	yes
PHGDH	PHGDH	PHGDH	chr1:1202544	shneg	shCHD1	OK	43,6603	87,0222	0,99506	5,536	5,00E-05	0,0004193	yes
PLEKHA5	PLEKHA5	PLEKHA5	chr12:192826	shneg	shCHD1	OK	8,39817	16,7292	0,994223	5,04897	5,00E-05	0,0004193	yes
RIMKLA	RIMKLA	RIMKLA	chr1:4284646	shneg	shCHD1	OK	0,929681	1,85092	0,993431	4,55098	5,00E-05	0,0004193	yes
AP3B2	AP3B2	AP3B2	chr15:832119	shneg	shCHD1	OK	12,3118	24,49	0,992145	5,2717	5,00E-05	0,0004193	yes
GJB2	GJB2	GJB2	chr13:207616	shneg	shCHD1	OK	3,19428	6,34695	0,990571	4,29707	5,00E-05	0,0004193	yes
FAM13C	FAM13C	FAM13C	chr10:609363	shneg	shCHD1	OK	1,21737	2,40896	0,984641	1,99037	0,0011	0,0063157	yes
NOSTRIN	NOSTRIN	NOSTRIN	chr2:1696430	shneg	shCHD1	OK	0,861531	1,70425	0,984158	2,24413	0,0004	0,00266202	yes
ABO	ABO	ABO	chr9:1361305	shneg	shCHD1	OK	2,17269	4,29308	0,982529	3,14628	5,00E-05	0,0004193	yes
ZNF506	ZNF506	ZNF506	chr19:199035	shneg	shCHD1	OK	0,808609	1,59473	0,979802	2,93187	5,00E-05	0,0004193	yes
LRRC1	LRRC1	LRRC1	chr6:5365977	shneg	shCHD1	OK	7,24253	14,2723	0,978652	4,88554	5,00E-05	0,0004193	yes
MGC16121	MGC16121	MGC16121	chrX:1336774	shneg	shCHD1	OK	3,69785	7,28323	0,977892	2,74451	5,00E-05	0,0004193	yes
PHC2	PHC2	PHC2	chr1:3378922	shneg	shCHD1	OK	38,3713	75,2619	0,971891	5,43958	5,00E-05	0,0004193	yes
DGCR9	DGCR9	DGCR9	chr22:190053	shneg	shCHD1	OK	1,65608	3,2482	0,971872	3,38316	5,00E-05	0,0004193	yes
CPA6	CPA6	CPA6	chr8:6833440	shneg	shCHD1	OK	0,634847	1,24087	0,966869	1,99624	0,0014	0,00768951	yes
GGT5	GGT5	GGT5	chr22:246156	shneg	shCHD1	OK	0,406567	0,793963	0,96558	1,73067	0,00235	0,0119595	yes
C12orf34	C12orf34	C12orf34	chr12:110152	shneg	shCHD1	OK	2,00109	3,90782	0,965576	3,73722	5,00E-05	0,0004193	yes
CYP3A4	CYP3A4	CYP3A4	chr7:9935458	shneg	shCHD1	OK	0,74401	1,45266	0,965301	2,59438	0,0001	0,00078473	yes
STAT3	STAT3	STAT3	chr17:404653	shneg	shCHD1	OK	23,4274	45,6628	0,962821	5,41766	5,00E-05	0,0004193	yes
DRD2	DRD2	DRD2	chr11:113280	shneg	shCHD1	OK	2,06697	4,02378	0,961036	3,82007	5,00E-05	0,0004193	yes
PLS3	PLS3	PLS3	chrX:1147951	shneg	shCHD1	OK	84,3373	164,082	0,960174	5,64566	5,00E-05	0,0004193	yes
LIPC	LIPC	LIPC	chr15:587241	shneg	shCHD1	OK	0,661898	1,28582	0,958004	1,86609	0,00305	0,0148336	yes
C1orf180	C1orf180	C1orf180	chr1:8509391	shneg	shCHD1	OK	0,439819	0,853771	0,956939	2,06478	0,0012	0,00680293	yes
MAP3K8	MAP3K8	MAP3K8	chr10:307229	shneg	shCHD1	OK	1,94012	3,76583	0,956818	3,86228	5,00E-05	0,0004193	yes
RAPGEF3	RAPGEF3	RAPGEF3	chr12:481284	shneg	shCHD1	OK	2,51793	4,88475	0,956048	4,69013	5,00E-05	0,0004193	yes
PSMD3	PSMD3	PSMD3	chr17:381370	shneg	shCHD1	OK	77,0226	149,422	0,956038	5,49163	5,00E-05	0,0004193	yes
RIMS4	RIMS4	RIMS4	chr20:433744	shneg	shCHD1	OK	2,41755	4,6865	0,954964	4,15207	5,00E-05	0,0004193	yes
C1orf130	C1orf130	C1orf130	chr1:2488256	shneg	shCHD1	OK	18,0115	34,9097	0,954707	5,26264	5,00E-05	0,0004193	yes
CNTNAP1	CNTNAP1	CNTNAP1	chr17:408346	shneg	shCHD1	OK	16,7423	32,3595	0,950694	5,30833	5,00E-05	0,0004193	yes
E2F8	E2F8	E2F8	chr11:192456	shneg	shCHD1	OK	3,95723	7,64369	0,94978	4,6457	5,00E-05	0,0004193	yes
LONRF1	LONRF1	LONRF1	chr8:1257940	shneg	shCHD1	OK	1,41532	2,73152	0,94857	3,62135	5,00E-05	0,0004193	yes
CAMKV	CAMKV	CAMKV	chr3:4989542	shneg	shCHD1	OK	3,20129	6,17574	0,94796	4,26425	5,00E-05	0,0004193	yes
ATF3	ATF3	ATF3	chr1:2127386	shneg	shCHD1	OK	0,585352	1,1254	0,94306	1,53683	0,00175	0,00931439	yes
SKOR1	SKOR1	SKOR1	chr15:681179	shneg	shCHD1	OK	1,60261	3,07744	0,941312	3,3988	5,00E-05	0,0004193	yes
CCDC99	CCDC99	CCDC99	chr5:1690106	shneg	shCHD1	OK	8,19876	15,7429	0,941219	4,66485	5,00E-05	0,0004193	yes
IPMK	IPMK	IPMK	chr10:599512	shneg	shCHD1	OK	3,08612	5,92522	0,941074	4,57776	5,00E-05	0,0004193	yes
NAGS	NAGS	NAGS	chr17:420820	shneg	shCHD1	OK	9,72768	18,6678	0,940384	4,59421	5,00E-05	0,0004193	yes
TOP2A	TOP2A	TOP2A	chr17:385447	shneg	shCHD1	OK	91,5038	175,405	0,938785	5,60483	5,00E-05	0,0004193	yes
HIVEP1	HIVEP1	HIVEP1	chr6:1201272	shneg	shCHD1	OK	1,94093	3,7163	0,937121	4,52994	5,00E-05	0,0004193	yes
KCNC1	KCNC1	KCNC1	chr11:177574	shneg	shCHD1	OK	0,878255	1,681	0,936606	3,35766	5,00E-05	0,0004193	yes
C2orf48	C2orf48	C2orf48	chr2:1028198	shneg	shCHD1	OK	1,38545	2,65053	0,935928	1,58246	0,0064	0,0274548	yes
PLEKHG1	PLEKHG1	PLEKHG1	chr6:1509209	shneg	shCHD1	OK	0,417734	0,798526	0,934754	2,95373	5,00E-05	0,0004193	yes
ZMYND15	ZMYND15	ZMYND15	chr17:464331	shneg	shCHD1	OK	0,641554	1,21535	0,921727	2,09076	0,001	0,0058183	yes
HSPA12A	HSPA12A	HSPA12A	chr10:118430	shneg	shCHD1	OK	10,8801	20,6017	0,921071	5,02625	5,00E-05	0,0004193	yes
KRT10	KRT10	KRT10	chr17:389743	shneg	shCHD1	OK	8,30739	15,7093	0,919152	3,62718	5,00E-05	0,0004193	yes
RPH3AL	RPH3AL	RPH3AL	chr17:62179-[illegible]	shneg	shCHD1	OK	1,3338	2,51962	0,91767	2,90961	5,00E-05	0,0004193	yes
LSM12	LSM12	LSM12	chr17:421120	shneg	shCHD1	OK	33,5529	63,3743	0,917461	5,09035	5,00E-05	0,0004193	yes
HLA-DMB	HLA-DMB	HLA-DMB	chr6_ssto_ha	shneg	shCHD1	OK	123,464	232,813	0,915076	5,25107	5,00E-05	0,0004193	yes

RAB23	RAB23	RAB23	chr6:5705358	shneg	shCHD1	OK	8,93996	16,8575	0,915052	4,60664	5,00E-05	0,0004193	yes
TUBG1	TUBG1	TUBG1	chr17:407315	shneg	shCHD1	OK	26,1356	49,0142	0,907183	3,33511	5,00E-05	0,0004193	yes
ANXA3	ANXA3	ANXA3	chr4:7947274	shneg	shCHD1	OK	8,11803	15,2124	0,906047	4,30036	5,00E-05	0,0004193	yes
CASC3	CASC3	CASC3	chr17:382965	shneg	shCHD1	OK	24,9965	46,7027	0,901781	5,07548	5,00E-05	0,0004193	yes
ROPN1L	ROPN1L	ROPN1L	chr5:1044197	shneg	shCHD1	OK	1,39316	2,60057	0,90047	1,79457	0,0022	0,011316	yes
AURKB	AURKB	AURKB	chr17:810804	shneg	shCHD1	OK	33,8806	63,2147	0,899801	4,72083	5,00E-05	0,0004193	yes
ASNS	ASNS	ASNS	chr7:9748142	shneg	shCHD1	OK	19,2256	35,84	0,898539	4,65201	5,00E-05	0,0004193	yes
HOXB6	HOXB6	HOXB6	chr17:466678	shneg	shCHD1	OK	21,682	40,3377	0,895631	3,46301	5,00E-05	0,0004193	yes
GRAPL	GRAPL	GRAPL	chr17:190307	shneg	shCHD1	OK	1,42431	2,64587	0,893484	1,84813	0,0028	0,0138449	yes
PTPRU	PTPRU	PTPRU	chr1:2956302	shneg	shCHD1	OK	5,47445	10,165	0,892821	4,61285	5,00E-05	0,0004193	yes
SMARCE1	SMARCE1	SMARCE1	chr17:387839	shneg	shCHD1	OK	80,0961	148,703	0,892633	5,16119	5,00E-05	0,0004193	yes
EIF4E3	EIF4E3	EIF4E3	chr3:7172843	shneg	shCHD1	OK	2,43847	4,52491	0,891912	4,4335	5,00E-05	0,0004193	yes
PTGIR	PTGIR	PTGIR	chr19:471237	shneg	shCHD1	OK	0,613148	1,13666	0,890497	1,89156	0,00265	0,0132017	yes
ADHFE1	ADHFE1	ADHFE1	chr8:6734471	shneg	shCHD1	OK	0,588191	1,08439	0,882522	1,76674	0,00425	0,0195438	yes
WDR36	WDR36	WDR36	chr5:1104278	shneg	shCHD1	OK	9,75617	17,986	0,882486	4,82671	5,00E-05	0,0004193	yes
GMNN	GMNN	GMNN	chr6:2477515	shneg	shCHD1	OK	9,68288	17,8359	0,881279	3,97013	5,00E-05	0,0004193	yes
TOX2	TOX2	TOX2	chr20:425434	shneg	shCHD1	OK	0,558893	1,02626	0,876747	1,73374	0,00105	0,00607369	yes
AQP7P3	AQP7P3	AQP7P3	chr9:4284436	shneg	shCHD1	OK	4,13292	7,58851	0,876654	1,88557	0,0021	0,0108578	yes
SLC25A40	SLC25A40	SLC25A40	chr7:8746381	shneg	shCHD1	OK	13,0313	23,9132	0,875829	3,59691	5,00E-05	0,0004193	yes
DERL3	DERL3	DERL3	chr22:241291	shneg	shCHD1	OK	1,91609	3,51114	0,873772	1,69747	0,00375	0,0176465	yes
ISL1	ISL1	ISL1	chr5:5066857	shneg	shCHD1	OK	0,837062	1,5338	0,873706	2,15245	0,00035	0,00236888	yes
KCNK5	KCNK5	KCNK5	chr6:3915674	shneg	shCHD1	OK	0,427439	0,783092	0,873463	2,08615	0,00115	0,00656221	yes
GSDMB	GSDMB	GSDMB	chr17:380608	shneg	shCHD1	OK	7,24845	13,2772	0,873202	4,02026	5,00E-05	0,0004193	yes
CDC25A	CDC25A	CDC25A	chr3:4819866	shneg	shCHD1	OK	3,47823	6,37053	0,873061	4,19956	5,00E-05	0,0004193	yes
STAMBPL1	STAMBPL1	STAMBPL1	chr10:906399	shneg	shCHD1	OK	8,77695	16,0707	0,872639	4,26505	5,00E-05	0,0004193	yes
KIAA1456	KIAA1456	KIAA1456	chr8:1280318	shneg	shCHD1	OK	2,44653	4,47165	0,870071	4,35872	5,00E-05	0,0004193	yes
ATF5	ATF5	ATF5	chr19:503929	shneg	shCHD1	OK	22,9248	41,8712	0,869052	2,55171	5,00E-05	0,0004193	yes
ASZ1	ASZ1	ASZ1	chr7:1170032	shneg	shCHD1	OK	0,442982	0,808533	0,86806	1,59274	0,00285	0,0140225	yes
ARRDC3	ARRDC3	ARRDC3	chr5:9066454	shneg	shCHD1	OK	23,5191	42,9203	0,867828	4,62589	5,00E-05	0,0004193	yes
CDC42SE2	CDC42SE2	CDC42SE2	chr5:1305997	shneg	shCHD1	OK	28,0378	51,1127	0,866306	4,85741	5,00E-05	0,0004193	yes
EFHB	EFHB	EFHB	chr3:1992096	shneg	shCHD1	OK	1,2184	2,2201	0,865633	2,81756	5,00E-05	0,0004193	yes
DCLK1	DCLK1	DCLK1	chr13:355164	shneg	shCHD1	OK	9,69361	17,6593	0,865322	3,25698	5,00E-05	0,0004193	yes
DIO3	DIO3	DIO3	chr14:102027	shneg	shCHD1	OK	1,8393	3,35058	0,865256	2,91996	5,00E-05	0,0004193	yes
SH2D7	SH2D7	SH2D7	chr15:783849	shneg	shCHD1	OK	0,526807	0,959252	0,864636	1,62293	0,0062	0,0267699	yes
OGDHL	OGDHL	OGDHL	chr10:509426	shneg	shCHD1	OK	6,42072	11,6315	0,857233	4,24393	5,00E-05	0,0004193	yes
RHOBTB3	RHOBTB3	RHOBTB3	chr5:9506684	shneg	shCHD1	OK	6,31212	11,4203	0,855402	4,45868	5,00E-05	0,0004193	yes
CCDC149	CCDC149	CCDC149	chr4:2480773	shneg	shCHD1	OK	1,71843	3,10897	0,855345	3,59895	5,00E-05	0,0004193	yes
HSD17B13	HSD17B13	HSD17B13	chr4:8822494	shneg	shCHD1	OK	0,514556	0,929176	0,852624	1,72914	0,00335	0,0160296	yes
PSG4	PSG4	PSG4	chr19:436968	shneg	shCHD1	OK	1,43358	2,5882	0,852333	2,52762	5,00E-05	0,0004193	yes
CCDC138	CCDC138	CCDC138	chr2:1094032	shneg	shCHD1	OK	3,8043	6,86393	0,851403	3,77744	5,00E-05	0,0004193	yes
ARHGAP5-AS:	ARHGAP5-AS:	ARHGAP5-AS:	chr14:325446	shneg	shCHD1	OK	0,95719	1,727	0,851394	1,706	0,0064	0,0274548	yes
CSRP2	CSRP2	CSRP2	chr12:772524	shneg	shCHD1	OK	26,8372	48,3254	0,848544	4,14747	5,00E-05	0,0004193	yes
SLC6A16	SLC6A16	SLC6A16	chr19:497928	shneg	shCHD1	OK	1,38367	2,4905	0,847931	2,93101	5,00E-05	0,0004193	yes
BTG3	BTG3	BTG3	chr21:189659	shneg	shCHD1	OK	8,28489	14,8888	0,845674	3,88062	5,00E-05	0,0004193	yes
DOCK4	DOCK4	DOCK4	chr7:1113661	shneg	shCHD1	OK	4,69243	8,42506	0,844352	4,44701	5,00E-05	0,0004193	yes
KLK14	KLK14	KLK14	chr19:515811	shneg	shCHD1	OK	1,32609	2,37746	0,842242	1,83543	0,0032	0,015482	yes
H2AFY2	H2AFY2	H2AFY2	chr10:718123	shneg	shCHD1	OK	8,86903	15,8808	0,840439	3,09805	5,00E-05	0,0004193	yes
LPAR1	LPAR1	LPAR1	chr9:1136360	shneg	shCHD1	OK	9,94257	17,7733	0,83802	4,33204	5,00E-05	0,0004193	yes
COL27A1	COL27A1	COL27A1	chr9:1169182	shneg	shCHD1	OK	5,99664	10,7036	0,835867	4,32732	5,00E-05	0,0004193	yes
ASB9	ASB9	ASB9	chrX:1526210	shneg	shCHD1	OK	4,58215	8,17186	0,83464	3,61538	5,00E-05	0,0004193	yes
G6PC3	G6PC3	G6PC3	chr17:421480	shneg	shCHD1	OK	61,3717	109,3	0,83265	4,61069	5,00E-05	0,0004193	yes
GP6	GP6	GP6	chr19:555250	shneg	shCHD1	OK	0,521909	0,929328	0,83239	1,57129	0,00435	0,019951	yes
MLKL	MLKL	MLKL	chr16:747057	shneg	shCHD1	OK	14,3172	25,4693	0,831007	4,3096	5,00E-05	0,0004193	yes
CAMKK1	CAMKK1	CAMKK1	chr17:376361	shneg	shCHD1	OK	6,09703	10,8336	0,829331	4,03754	5,00E-05	0,0004193	yes
VASH2	VASH2	VASH2	chr1:2131238	shneg	shCHD1	OK	0,796707	1,4154	0,829086	2,61321	0,0001	0,00078473	yes
KLRAP1	KLRAP1	KLRAP1	chr12:107410	shneg	shCHD1	OK	0,846426	1,50226	0,827675	2,0486	0,001	0,0058183	yes
HOXB5	HOXB5	HOXB5	chr17:466678	shneg	shCHD1	OK	13,88	24,6163	0,826602	2,65192	5,00E-05	0,0004193	yes
MYO5B	MYO5B	MYO5B	chr18:473491	shneg	shCHD1	OK	0,41371	0,731989	0,823201	2,86731	5,00E-05	0,0004193	yes
ATP6V0A1	ATP6V0A1	ATP6V0A1	chr17:406108	shneg	shCHD1	OK	21,6131	38,2405	0,823195	4,56846	5,00E-05	0,0004193	yes
LOC10029414	LOC10029414	LOC10029414	chr6_ssto_ha	shneg	shCHD1	OK	38,1686	67,4929	0,822351	4,67998	5,00E-05	0,0004193	yes
FAS	FAS	FAS	chr10:906948	shneg	shCHD1	OK	22,9025	40,4455	0,82047	3,46576	5,00E-05	0,0004193	yes
PAK6	PAK6	PAK6	chr15:404532	shneg	shCHD1	OK	17,1367	30,2563	0,820144	3,1509	5,00E-05	0,0004193	yes
KIF26B	KIF26B	KIF26B	chr1:2453182	shneg	shCHD1	OK	1,32575	2,33927	0,819245	3,72395	5,00E-05	0,0004193	yes
DEK	DEK	DEK	chr6:1822439	shneg	shCHD1	OK	39,025	68,8269	0,818573	4,604	5,00E-05	0,0004193	yes
MED24	MED24	MED24	chr17:381753	shneg	shCHD1	OK	21,6175	38,1179	0,818268	4,53967	5,00E-05	0,0004193	yes
PSMC3IP	PSMC3IP	PSMC3IP	chr17:407190	shneg	shCHD1	OK	23,236	40,9621	0,817931	2,55134	5,00E-05	0,0004193	yes
GSG2	GSG2	GSG2	chr17:361791	shneg	shCHD1	OK	4,03735	7,11363	0,817175	3,14586	5,00E-05	0,0004193	yes
TGFBR3	TGFBR3	TGFBR3	chr1:9214589	shneg	shCHD1	OK	9,97872	17,5659	0,815854	4,42086	5,00E-05	0,0004193	yes
FAM167A	FAM167A	FAM167A	chr8:1119714	shneg	shCHD1	OK	0,776564	1,36638	0,815181	2,58092	5,00E-05	0,0004193	yes
PAPSS2	PAPSS2	PAPSS2	chr10:894194	shneg	shCHD1	OK	2,29291	4,02491	0,811776	3,57654	5,00E-05	0,0004193	yes
EPHX4	EPHX4	EPHX4	chr1:9249553	shneg	shCHD1	OK	2,23324	3,91799	0,810976	2,47483	5,00E-05	0,0004193	yes
FBLN7	FBLN7	FBLN7	chr2:1128959	shneg	shCHD1	OK	1,7331	3,0381	0,809809	2,7374	5,00E-05	0,0004193	yes
C21orf58	C21orf58	C21orf58	chr21:477210	shneg	shCHD1	OK	5,84751	10,2487	0,809549	3,88402	5,00E-05	0,0004193	yes
RSPH1	RSPH1	RSPH1	chr21:438925	shneg	shCHD1	OK	1,4598	2,55678	0,808553	1,99927	0,00125	0,00702633	yes
CCR10	CCR10	CCR10	chr17:408314	shneg	shCHD1	OK	3,48013	6,09477	0,808433	2,74389	5,00E-05	0,0004193	yes
GPM6A	GPM6A	GPM6A	chr4:1765540	shneg	shCHD1	OK	2,45679	4,30258	0,808426	3,37826	5,00E-05	0,0004193	yes
ADAMTS7	ADAMTS7	ADAMTS7	chr15:790515	shneg	shCHD1	OK	1,67735	2,93646	0,807894	3,61915	5,00E-05	0,0004193	yes
HPCA	HPCA	HPCA	chr1:3335209	shneg	shCHD1	OK	5,31214	9,29852	0,807709	1,64953	0,00445	0,020336	yes
MIR17HG	MIR17HG	MIR17HG	chr13:920000	shneg	shCHD1	OK	1,49927	2,62302	0,806972	1,52691	0,0062	0,0267699	yes
P2RX5	P2RX5	P2RX5	chr17:353976	shneg	shCHD1	OK	6,57385	11,4964	0,806377	1,85783	0,0013	0,00726051	yes
HOTAIRM1	HOTAIRM1	HOTAIRM1	chr7:2713571	shneg	shCHD1	OK	6,57676	11,496	0,805682	3,03322	5,00E-05	0,0004193	yes
ELL2	ELL2	ELL2	chr5:9522080	shneg	shCHD1	OK	23,4246	40,9418	0,805553	4,59611	5,00E-05	0,0004193	yes
LOC283788	LOC283788	LOC283788	chrUn_gl0002	shneg	shCHD1	OK	6,65614	11,6272	0,804747	4,03028	5,00E-05	0,0004193	yes
BMI1	BMI1	BMI1	chr10:226053	shneg	shCHD1	OK	7,79748	13,6132	0,803927	1,9565	0,00065	0,0040329	yes
C4orf32	C4orf32	C4orf32	chr4:1130665	shneg	shCHD1	OK	0,59338	1,03484	0,802382	1,90446	0,0019	0,00996052	yes
HOXB4	HOXB4	HOXB4	chr17:466528	shneg	shCHD1	OK	14,5699	25,3777	0,800565	4,07965	5,00E-05	0,0004193	yes
RELB	RELB	RELB	chr19:455047	shneg	shCHD1	OK	11,4035	19,8575	0,800208	4,01895	5,00E-05	0,0004193	yes
APOBEC3B	APOBEC3B	APOBEC3B	chr22:393535	shneg	shCHD1	OK	13,2429	23,0339	0,798535	3,83318	5,00E-05	0,0004193	yes
HMGA2	HMGA2	HMGA2	chr12:661518	shneg	shCHD1	OK	63,5056	110,413	0,797951	4,58059	5,00E-05	0,0004193	yes
GPR137C	GPR137C	GPR137C	chr14:530198	shneg	shCHD1	OK	0,704392	1,22418	0,797367	2,37224	0,0002	0,00144867	yes
PSME3	PSME3	PSME3	chr17:409854	shneg	shCHD1	OK	39,2139	68,1082	0,796464	4,51064	5,00E-05	0,0004193	yes
CCRN4L	CCRN4L	CCRN4L	chr4:1399369	shneg	shCHD1	OK	5,4582	9,47565	0,795799	3,64272	5,00E-05	0,0004193	yes
TST	TST	TST	chr22:374069	shneg	shCHD1	OK	25,1135	43,5925	0,79562	3,99733	5,00E-05	0,0004193	yes

SERPINA6	SERPINA6	SERPINA6	chr14:947705	shneg	shCHD1	OK	22,218	38,4988	0,793086	4,07482	5,00E-05	0,0004193	yes
TAF5	TAF5	TAF5	chr10:105127	shneg	shCHD1	OK	2,01013	3,47829	0,791087	1,45043	0,0122	0,0469301	yes
FBXO43	FBXO43	FBXO43	chr8:1011455	shneg	shCHD1	OK	0,969353	1,67224	0,786685	2,49818	5,00E-05	0,0004193	yes
NUP153	NUP153	NUP153	chr6:1761526	shneg	shCHD1	OK	11,1029	19,1463	0,786124	4,28523	5,00E-05	0,0004193	yes
TPI1P2	TPI1P2	TPI1P2	chr7:1286952	shneg	shCHD1	OK	1,03124	1,77664	0,784767	2,02516	0,00135	0,00748275	yes
SAMD5	SAMD5	SAMD5	chr6:1478298	shneg	shCHD1	OK	14,1729	24,4063	0,784116	4,36985	5,00E-05	0,0004193	yes
PGRMC1	PGRMC1	PGRMC1	chrX:1183702	shneg	shCHD1	OK	28,2905	48,6687	0,782676	4,20049	5,00E-05	0,0004193	yes
CYTH4	CYTH4	CYTH4	chr22:376784	shneg	shCHD1	OK	0,459316	0,788856	0,780275	1,74906	0,0042	0,0193522	yes
DEGS2	DEGS2	DEGS2	chr14:100612	shneg	shCHD1	OK	0,848768	1,45749	0,780041	1,53722	0,01105	0,0432225	yes
SLC16A9	SLC16A9	SLC16A9	chr10:614105	shneg	shCHD1	OK	5,00926	8,59489	0,778881	3,81266	5,00E-05	0,0004193	yes
MARCKSL1	MARCKSL1	MARCKSL1	chr1:3279943	shneg	shCHD1	OK	53,7571	91,9519	0,774424	4,26754	5,00E-05	0,0004193	yes
ETS1	ETS1	ETS1	chr11:128328	shneg	shCHD1	OK	33,6617	57,5679	0,774158	4,4299	5,00E-05	0,0004193	yes
MYO1B	MYO1B	MYO1B	chr2:1921101	shneg	shCHD1	OK	5,30141	9,06596	0,774083	3,89396	5,00E-05	0,0004193	yes
DGCR5	DGCR5	DGCR5	chr22:189580	shneg	shCHD1	OK	3,49404	5,97235	0,773403	3,58814	5,00E-05	0,0004193	yes
RGS20	RGS20	RGS20	chr8:5476436	shneg	shCHD1	OK	2,87869	4,92016	0,773294	2,81937	5,00E-05	0,0004193	yes
EMR1	EMR1	EMR1	chr19:688755	shneg	shCHD1	OK	7,89623	13,4769	0,771253	3,78302	5,00E-05	0,0004193	yes
ZNF695	ZNF695	ZNF695	chr1:2471088	shneg	shCHD1	OK	6,34303	10,8209	0,770582	1,92157	0,0015	0,00814879	yes
IL29	IL29	IL29	chr19:397869	shneg	shCHD1	OK	1,54808	2,63727	0,76856	1,49789	0,013	0,0492861	yes
ACLY	ACLY	ACLY	chr17:400231	shneg	shCHD1	OK	47,9503	81,6472	0,767863	4,44224	5,00E-05	0,0004193	yes
TAX1BP3	TAX1BP3	TAX1BP3	chr17:353976	shneg	shCHD1	OK	22,4118	38,1512	0,767474	2,46156	5,00E-05	0,0004193	yes
IKBKE	IKBKE	IKBKE	chr1:2066435	shneg	shCHD1	OK	2,16767	3,68723	0,766397	3,29642	5,00E-05	0,0004193	yes
MMP25	MMP25	MMP25	chr16:309668	shneg	shCHD1	OK	1,44806	2,46307	0,766341	2,90755	5,00E-05	0,0004193	yes
PSORS1C3	PSORS1C3	PSORS1C3	chr6_ssto_ha	shneg	shCHD1	OK	9,69681	16,4936	0,766323	2,6481	5,00E-05	0,0004193	yes
COMMD3-BM	COMMD3-BM	COMMD3-BM	chr10:226053	shneg	shCHD1	OK	9,48872	16,1272	0,765212	2,01168	0,002	0,0104181	yes
MEX3A	MEX3A	MEX3A	chr1:1560418	shneg	shCHD1	OK	3,17533	5,39595	0,764968	3,7331	5,00E-05	0,0004193	yes
VPS25	VPS25	VPS25	chr17:409254	shneg	shCHD1	OK	39,7149	67,4641	0,764439	4,00787	5,00E-05	0,0004193	yes
CHRNB2	CHRNB2	CHRNB2	chr1:1545402	shneg	shCHD1	OK	6,11433	10,375	0,762841	3,97484	5,00E-05	0,0004193	yes
NFE2L3	NFE2L3	NFE2L3	chr7:2619184	shneg	shCHD1	OK	10,5575	17,9049	0,762078	3,99807	5,00E-05	0,0004193	yes
PVRL1	PVRL1	PVRL1	chr11:119508	shneg	shCHD1	OK	1,69161	2,86746	0,761377	2,74779	5,00E-05	0,0004193	yes
NDRG1	NDRG1	NDRG1	chr8:1342494	shneg	shCHD1	OK	54,1647	91,7737	0,760727	4,36219	5,00E-05	0,0004193	yes
LAMC2	LAMC2	LAMC2	chr1:1831551	shneg	shCHD1	OK	45,6937	77,3924	0,760197	4,42169	5,00E-05	0,0004193	yes
AGBL3	AGBL3	AGBL3	chr7:1346712	shneg	shCHD1	OK	0,775992	1,3119	0,757546	2,25277	0,0004	0,00266202	yes
OSR2	OSR2	OSR2	chr8:9995663	shneg	shCHD1	OK	1,12044	1,89391	0,757303	1,88003	0,0019	0,00996052	yes
CTPS	CTPS	CTPS	chr1:4144500	shneg	shCHD1	OK	17,5979	29,7325	0,756634	4,0832	5,00E-05	0,0004193	yes
PPIF	PPIF	PPIF	chr10:811072	shneg	shCHD1	OK	16,9037	28,5382	0,755557	3,94073	5,00E-05	0,0004193	yes
RASGEF1C	RASGEF1C	RASGEF1C	chr5:1795277	shneg	shCHD1	OK	0,74143	1,25155	0,75533	1,77877	0,0037	0,0174466	yes
ARSB	ARSB	ARSB	chr5:7807303	shneg	shCHD1	OK	8,13959	13,7109	0,752297	3,47866	5,00E-05	0,0004193	yes
DSCC1	DSCC1	DSCC1	chr8:1208461	shneg	shCHD1	OK	5,19415	8,74884	0,752205	3,48318	5,00E-05	0,0004193	yes
SPNS3	SPNS3	SPNS3	chr17:433721	shneg	shCHD1	OK	1,08595	1,8282	0,751458	1,91284	0,0013	0,00726051	yes
PAK1IP1	PAK1IP1	PAK1IP1	chr6:1069518	shneg	shCHD1	OK	7,52747	12,6723	0,751438	3,44674	5,00E-05	0,0004193	yes
FARP1	FARP1	FARP1	chr13:987954	shneg	shCHD1	OK	4,77385	8,02525	0,749393	3,54013	5,00E-05	0,0004193	yes
IL17D	IL17D	IL17D	chr13:212774	shneg	shCHD1	OK	0,868237	1,45694	0,746783	1,70873	0,00405	0,0187979	yes
C6orf228	C6orf228	C6orf228	chr6:1109426	shneg	shCHD1	OK	3,6097	6,04834	0,744658	3,58892	5,00E-05	0,0004193	yes
TMEM145	TMEM145	TMEM145	chr19:428174	shneg	shCHD1	OK	12,0058	20,0848	0,742364	3,61613	5,00E-05	0,0004193	yes
SLC2A10	SLC2A10	SLC2A10	chr20:453382	shneg	shCHD1	OK	2,45758	4,11015	0,741955	3,38399	5,00E-05	0,0004193	yes
SAV1	SAV1	SAV1	chr14:511003	shneg	shCHD1	OK	7,21215	12,0614	0,741892	3,65948	5,00E-05	0,0004193	yes
FAM105A	FAM105A	FAM105A	chr5:1458189	shneg	shCHD1	OK	1,16051	1,9407	0,741812	3,31936	5,00E-05	0,0004193	yes
MFHAS1	MFHAS1	MFHAS1	chr8:8641998	shneg	shCHD1	OK	3,1896	5,33235	0,741397	3,59743	5,00E-05	0,0004193	yes
TYMS	TYMS	TYMS	chr18:596997	shneg	shCHD1	OK	27,1742	45,405	0,740615	2,4349	0,00015	0,00112331	yes
PLEKHH3	PLEKHH3	PLEKHH3	chr17:408199	shneg	shCHD1	OK	12,9593	21,6419	0,739843	3,87892	5,00E-05	0,0004193	yes
ADNP2	ADNP2	ADNP2	chr18:778669	shneg	shCHD1	OK	8,38675	14,002	0,739452	3,93508	5,00E-05	0,0004193	yes
HSPG2	HSPG2	HSPG2	chr1:2213875	shneg	shCHD1	OK	6,4697	10,7976	0,738939	4,08251	5,00E-05	0,0004193	yes
VRK1	VRK1	VRK1	chr14:972636	shneg	shCHD1	OK	9,25566	15,4442	0,738655	3,53669	5,00E-05	0,0004193	yes
MUC20	MUC20	MUC20	chr3:1954477	shneg	shCHD1	OK	2,22114	3,69959	0,736065	3,51054	5,00E-05	0,0004193	yes
RALA	RALA	RALA	chr7:3966315	shneg	shCHD1	OK	19,2191	32,0088	0,735927	3,95268	5,00E-05	0,0004193	yes
CAMK2A	CAMK2A	CAMK2A	chr5:1495990	shneg	shCHD1	OK	21,6825	36,0868	0,734942	4,10951	5,00E-05	0,0004193	yes
RNF122	RNF122	RNF122	chr8:3340527	shneg	shCHD1	OK	1,07963	1,79617	0,734391	1,86331	0,0019	0,00996052	yes
PITPNA	PITPNA	PITPNA	chr17:142021	shneg	shCHD1	OK	18,1575	30,1517	0,731672	3,88768	5,00E-05	0,0004193	yes
HOXB-AS3	HOXB-AS3	HOXB-AS3	chr17:466678	shneg	shCHD1	OK	32,206	53,4425	0,730656	1,49903	0,0099	0,0395347	yes
TXK	TXK	TXK	chr4:4806840	shneg	shCHD1	OK	1,05444	1,74863	0,729758	2,23601	0,0001	0,00078473	yes
NRTN	NRTN	NRTN	chr19:582381	shneg	shCHD1	OK	1,6009	2,6542	0,729396	1,66851	0,006	0,0260761	yes
WDR17	WDR17	WDR17	chr4:1769869	shneg	shCHD1	OK	1,39877	2,31896	0,729323	3,31811	5,00E-05	0,0004193	yes
F5	F5	F5	chr1:1694811	shneg	shCHD1	OK	1,23602	2,048	0,728513	3,41236	5,00E-05	0,0004193	yes
MCM10	MCM10	MCM10	chr10:132035	shneg	shCHD1	OK	4,90583	8,12736	0,728288	3,61073	5,00E-05	0,0004193	yes
SASH1	SASH1	SASH1	chr6:1486637	shneg	shCHD1	OK	6,57171	10,8777	0,72703	3,90364	5,00E-05	0,0004193	yes
CCNB1	CCNB1	CCNB1	chr5:6846283	shneg	shCHD1	OK	105,075	173,781	0,725853	4,21137	5,00E-05	0,0004193	yes
CTSH	CTSH	CTSH	chr15:792140	shneg	shCHD1	OK	17,5966	29,1004	0,725744	3,6252	5,00E-05	0,0004193	yes
SHMT2	SHMT2	SHMT2	chr12:576233	shneg	shCHD1	OK	40,0143	66,08	0,723697	3,95505	5,00E-05	0,0004193	yes
SPC25	SPC25	SPC25	chr2:1697274	shneg	shCHD1	OK	8,62751	14,2206	0,720969	3,30884	5,00E-05	0,0004193	yes
EVPL	EVPL	EVPL	chr17:740029	shneg	shCHD1	OK	6,80211	11,2112	0,720881	3,83268	5,00E-05	0,0004193	yes
RRM2	RRM2	RRM2	chr2:1026269	shneg	shCHD1	OK	43,8166	72,2153	0,720827	4,12368	5,00E-05	0,0004193	yes
SLC17A1	SLC17A1	SLC17A1	chr6:2578312	shneg	shCHD1	OK	5,7277	9,4399	0,720814	3,25928	5,00E-05	0,0004193	yes
DNAJC7	DNAJC7	DNAJC7	chr17:401187	shneg	shCHD1	OK	65,4237	107,733	0,719577	2,64074	5,00E-05	0,0004193	yes
HAVCR2	HAVCR2	HAVCR2	chr5:1565128	shneg	shCHD1	OK	2,69682	4,43766	0,718536	2,92275	5,00E-05	0,0004193	yes
C18orf54	C18orf54	C18orf54	chr18:518851	shneg	shCHD1	OK	1,09785	1,80566	0,717841	2,79131	5,00E-05	0,0004193	yes
KLKB1	KLKB1	KLKB1	chr4:1871486	shneg	shCHD1	OK	1,10775	1,82061	0,716795	1,98707	0,0011	0,0063157	yes
ERI1	ERI1	ERI1	chr8:8860313	shneg	shCHD1	OK	2,86691	4,71103	0,716543	3,44146	5,00E-05	0,0004193	yes
HUNK	HUNK	HUNK	chr21:332456	shneg	shCHD1	OK	0,540385	0,887132	0,715163	2,47451	5,00E-05	0,0004193	yes
BLM	BLM	BLM	chr15:912605	shneg	shCHD1	OK	4,68531	7,68221	0,713376	3,51195	5,00E-05	0,0004193	yes
SLC39A14	SLC39A14	SLC39A14	chr8:2222476	shneg	shCHD1	OK	13,8717	22,7283	0,712342	3,42012	5,00E-05	0,0004193	yes
NBR2	NBR2	NBR2	chr17:412775	shneg	shCHD1	OK	3,67333	6,01396	0,711227	2,55707	5,00E-05	0,0004193	yes
MIR210HG	MIR210HG	MIR210HG	chr11:565656	shneg	shCHD1	OK	1,76553	2,88607	0,709005	2,37349	0,00015	0,00112331	yes
TFCP2L1	TFCP2L1	TFCP2L1	chr2:1219741	shneg	shCHD1	OK	0,806218	1,31647	0,707429	3,07249	5,00E-05	0,0004193	yes
SKA3	SKA3	SKA3	chr13:217277	shneg	shCHD1	OK	8,40672	13,7224	0,706917	2,16411	0,00025	0,00176854	yes
MYH14	MYH14	MYH14	chr19:507068	shneg	shCHD1	OK	3,61059	5,8889	0,705763	3,55959	5,00E-05	0,0004193	yes
MMD	MMD	MMD	chr17:534699	shneg	shCHD1	OK	3,66435	5,97634	0,705706	3,29993	5,00E-05	0,0004193	yes
TUBB	TUBB	TUBB	chr6_ssto_ha	shneg	shCHD1	OK	604,449	985,635	0,705433	4,2683	5,00E-05	0,0004193	yes
SLCO2B1	SLCO2B1	SLCO2B1	chr11:748620	shneg	shCHD1	OK	6,24428	10,1793	0,70503	3,52365	5,00E-05	0,0004193	yes
SESTD1	SESTD1	SESTD1	chr2:1799664	shneg	shCHD1	OK	1,31201	2,13839	0,704738	3,43663	5,00E-05	0,0004193	yes
RARA	RARA	RARA	chr17:384654	shneg	shCHD1	OK	5,89686	9,58779	0,701251	3,40817	5,00E-05	0,0004193	yes
BIK	BIK	BIK	chr22:435067	shneg	shCHD1	OK	2,36343	3,83967	0,7001	1,75707	0,00345	0,0164347	yes
RPL13AP20	RPL13AP20	RPL13AP20	chr12:130284	shneg	shCHD1	OK	5,73371	9,28287	0,695103	2,06225	0,00055	0,00350455	yes
MIOX	MIOX	MIOX	chr22:509252	shneg	shCHD1	OK	6,31621	10,2224	0,694607	2,99034	5,00E-05	0,0004193	yes

ORMDL3	ORMDL3	ORMDL3	chr17:380772	shneg	shCHD1	OK	25,1463	40,615	0,691663	3,70958	5,00E-05	0,0004193	yes
ORC1	ORC1	ORC1	chr1:5283850	shneg	shCHD1	OK	5,63304	9,08846	0,690123	3,35018	5,00E-05	0,0004193	yes
CREM	CREM	CREM	chr10:354157	shneg	shCHD1	OK	19,0979	30,8035	0,689682	3,25448	5,00E-05	0,0004193	yes
TMEM107	TMEM107	TMEM107	chr17:807629	shneg	shCHD1	OK	5,51204	8,88436	0,68868	3,07471	5,00E-05	0,0004193	yes
FGF18	FGF18	FGF18	chr5:1708466	shneg	shCHD1	OK	1,6117	2,5966	0,688039	2,11173	0,00065	0,0040329	yes
AP1S2	AP1S2	AP1S2	chrX:1584392	shneg	shCHD1	OK	33,6102	54,1349	0,687658	3,77438	5,00E-05	0,0004193	yes
CXADR	CXADR	CXADR	chr21:188852	shneg	shCHD1	OK	2,40901	3,87867	0,687121	2,39726	5,00E-05	0,0004193	yes
HNRNPAB	HNRNPAB	HNRNPAB	chr5:1776315	shneg	shCHD1	OK	113,263	182,327	0,686857	3,69501	5,00E-05	0,0004193	yes
LOC10049948	LOC10049948	LOC10049948	chr10:227243	shneg	shCHD1	OK	1,43898	2,31633	0,686793	2,23346	0,00015	0,00112331	yes
TCOF1	TCOF1	TCOF1	chr5:1497372	shneg	shCHD1	OK	34,5792	55,6107	0,68546	3,91421	5,00E-05	0,0004193	yes
ANLN	ANLN	ANLN	chr7:3636375	shneg	shCHD1	OK	52,8945	85,0372	0,684978	3,702	5,00E-05	0,0004193	yes
EPPK1	EPPK1	EPPK1	chr8:1449399	shneg	shCHD1	OK	1,74524	2,80123	0,682635	3,26028	5,00E-05	0,0004193	yes
RPE65	RPE65	RPE65	chr1:6889450	shneg	shCHD1	OK	1,13535	1,82093	0,681534	2,05216	0,0006	0,00377139	yes
STX3	STX3	STX3	chr11:595225	shneg	shCHD1	OK	6,06445	9,72544	0,681386	3,59645	5,00E-05	0,0004193	yes
GALNT10	GALNT10	GALNT10	chr5:1535702	shneg	shCHD1	OK	10,8433	17,38	0,680616	3,62128	5,00E-05	0,0004193	yes
PHF7	PHF7	PHF7	chr3:5244452	shneg	shCHD1	OK	0,862899	1,38287	0,680407	1,62526	0,00525	0,0232593	yes
PDK1	PDK1	PDK1	chr2:1734207	shneg	shCHD1	OK	1,8651	2,98833	0,680086	3,05479	5,00E-05	0,0004193	yes
ARHGAP19	ARHGAP19	ARHGAP19	chr10:987577	shneg	shCHD1	OK	3,982	6,37387	0,678678	3,31795	5,00E-05	0,0004193	yes
LYPLA1	LYPLA1	LYPLA1	chr8:5495893	shneg	shCHD1	OK	27,6413	44,2412	0,678563	3,70346	5,00E-05	0,0004193	yes
ZNF608	ZNF608	ZNF608	chr5:1239726	shneg	shCHD1	OK	1,28491	2,05655	0,678563	2,89247	5,00E-05	0,0004193	yes
C1orf135	C1orf135	C1orf135	chr1:2616049	shneg	shCHD1	OK	3,82737	6,12036	0,677264	2,90875	5,00E-05	0,0004193	yes
SMPD3	SMPD3	SMPD3	chr16:683922	shneg	shCHD1	OK	1,57809	2,51851	0,674386	3,00555	5,00E-05	0,0004193	yes
MDC1	MDC1	MDC1	chr6_ssto_ha	shneg	shCHD1	OK	8,28206	13,2164	0,674272	3,66138	5,00E-05	0,0004193	yes
MMP15	MMP15	MMP15	chr16:580592	shneg	shCHD1	OK	6,66899	10,6373	0,673598	3,43548	5,00E-05	0,0004193	yes
NUDT15	NUDT15	NUDT15	chr13:486117	shneg	shCHD1	OK	14,393	22,9566	0,673543	3,4138	5,00E-05	0,0004193	yes
SLC25A5	SLC25A5	SLC25A5	chrX:1185999	shneg	shCHD1	OK	229,674	365,862	0,671716	3,91902	5,00E-05	0,0004193	yes
NKX3-1	NKX3-1	NKX3-1	chr8:2353620	shneg	shCHD1	OK	0,981641	1,56306	0,671108	1,9188	0,00065	0,0040329	yes
MLX	MLX	MLX	chr17:407190	shneg	shCHD1	OK	27,3794	43,5861	0,670776	2,97032	5,00E-05	0,0004193	yes
CHD7	CHD7	CHD7	chr8:6159132	shneg	shCHD1	OK	2,54957	4,04822	0,667034	3,4222	5,00E-05	0,0004193	yes
LOC541471	LOC541471	LOC541471	chr2:1121245	shneg	shCHD1	OK	11,42	18,1259	0,666488	2,35631	0,00015	0,00112331	yes
RAD51	RAD51	RAD51	chr15:409873	shneg	shCHD1	OK	6,74435	10,6971	0,665466	3,23602	5,00E-05	0,0004193	yes
SREK1IP1	SREK1IP1	SREK1IP1	chr5:6398613	shneg	shCHD1	OK	5,26794	8,35452	0,665316	3,45341	5,00E-05	0,0004193	yes
PRKAR1B	PRKAR1B	PRKAR1B	chr7:588833-	shneg	shCHD1	OK	7,51863	11,9195	0,664781	1,77464	0,00235	0,0119595	yes
LSM11	LSM11	LSM11	chr5:1571707	shneg	shCHD1	OK	1,497	2,37193	0,663985	2,20371	0,0004	0,00266202	yes
MYBL2	MYBL2	MYBL2	chr20:422957	shneg	shCHD1	OK	28,1421	44,5567	0,662914	3,638	5,00E-05	0,0004193	yes
ZWILCH	ZWILCH	ZWILCH	chr15:667974	shneg	shCHD1	OK	10,5189	16,647	0,662272	3,22976	5,00E-05	0,0004193	yes
CLSPN	CLSPN	CLSPN	chr1:3619771	shneg	shCHD1	OK	4,12637	6,52663	0,661468	3,43681	5,00E-05	0,0004193	yes
CD83	CD83	CD83	chr6:1411748	shneg	shCHD1	OK	6,5533	10,3649	0,661413	3,25293	5,00E-05	0,0004193	yes
CENPA	CENPA	CENPA	chr2:2700888	shneg	shCHD1	OK	12,8056	20,2509	0,661207	3,1836	5,00E-05	0,0004193	yes
LOC10050565	LOC10050565	LOC10050565	chr8:6710434	shneg	shCHD1	OK	2,97228	4,70021	0,661155	2,28203	5,00E-05	0,0004193	yes
SNRNP48	SNRNP48	SNRNP48	chr6:7590431	shneg	shCHD1	OK	3,39844	5,37282	0,660807	3,2098	5,00E-05	0,0004193	yes
UBLCP1	UBLCP1	UBLCP1	chr5:1586900	shneg	shCHD1	OK	17,6828	27,9415	0,660059	3,47197	5,00E-05	0,0004193	yes
HDAC5	HDAC5	HDAC5	chr17:421541	shneg	shCHD1	OK	16,3791	25,8807	0,66002	3,64931	5,00E-05	0,0004193	yes
MTBP	MTBP	MTBP	chr8:1214576	shneg	shCHD1	OK	4,57385	7,22399	0,659388	3,17935	5,00E-05	0,0004193	yes
RHOD	RHOD	RHOD	chr11:668242	shneg	shCHD1	OK	2,23797	3,53406	0,659134	1,75384	0,0028	0,0138449	yes
TBC1D7	TBC1D7	TBC1D7	chr6:1330518	shneg	shCHD1	OK	5,25801	8,30129	0,658818	2,4135	0,0001	0,00078473	yes
ETS2	ETS2	ETS2	chr21:401772	shneg	shCHD1	OK	0,663475	1,04723	0,658471	1,85583	0,00195	0,0102111	yes
PTCHD2	PTCHD2	PTCHD2	chr1:1153929	shneg	shCHD1	OK	2,67487	4,21453	0,655902	3,17738	5,00E-05	0,0004193	yes
SFXN2	SFXN2	SFXN2	chr10:104474	shneg	shCHD1	OK	2,76798	4,35953	0,65534	2,73141	5,00E-05	0,0004193	yes
INSM2	INSM2	INSM2	chr14:360032	shneg	shCHD1	OK	0,556449	0,875628	0,654068	1,53625	0,0102	0,0404429	yes
SDC4	SDC4	SDC4	chr20:439539	shneg	shCHD1	OK	25,3322	39,8618	0,654038	3,55579	5,00E-05	0,0004193	yes
CNP	CNP	CNP	chr17:401187	shneg	shCHD1	OK	21,6571	34,0674	0,653553	2,40698	5,00E-05	0,0004193	yes
SEMA7A	SEMA7A	SEMA7A	chr15:747016	shneg	shCHD1	OK	0,474679	0,746519	0,653228	1,42884	0,0094	0,0379295	yes
RUNDC1	RUNDC1	RUNDC1	chr17:411325	shneg	shCHD1	OK	8,97298	14,0996	0,651992	3,37741	5,00E-05	0,0004193	yes
IGFBP2	IGFBP2	IGFBP2	chr2:2174981	shneg	shCHD1	OK	16,2426	25,5209	0,651894	3,19515	5,00E-05	0,0004193	yes
LOC10050586	LOC10050586	LOC10050586	chr16:664424	shneg	shCHD1	OK	2,22011	3,48664	0,651203	1,53694	0,01	0,0398199	yes
PLAU	PLAU	PLAU	chr10:756697	shneg	shCHD1	OK	27,558	43,2612	0,650603	3,53478	5,00E-05	0,0004193	yes
GGCT	GGCT	GGCT	chr7:3053623	shneg	shCHD1	OK	26,1808	41,0524	0,648958	3,2102	5,00E-05	0,0004193	yes
DIRAS1	DIRAS1	DIRAS1	chr19:271456	shneg	shCHD1	OK	12,5782	19,7229	0,648949	3,41818	5,00E-05	0,0004193	yes
SOX13	SOX13	SOX13	chr1:2040422	shneg	shCHD1	OK	2,26209	3,54562	0,64838	2,89034	5,00E-05	0,0004193	yes
CECR6	CECR6	CECR6	chr22:175971	shneg	shCHD1	OK	2,11354	3,30896	0,646715	2,84223	5,00E-05	0,0004193	yes
BCAN	BCAN	BCAN	chr1:1566117	shneg	shCHD1	OK	5,04982	7,90505	0,646542	3,15008	5,00E-05	0,0004193	yes
RIOK1	RIOK1	RIOK1	chr6:7390061	shneg	shCHD1	OK	3,93977	6,15695	0,644102	2,94377	5,00E-05	0,0004193	yes
SHROOM3	SHROOM3	SHROOM3	chr4:7735625	shneg	shCHD1	OK	1,32639	2,07055	0,642507	3,16283	5,00E-05	0,0004193	yes
INTS8	INTS8	INTS8	chr8:9583553	shneg	shCHD1	OK	7,91605	12,3549	0,642231	2,88948	5,00E-05	0,0004193	yes
TCF7L2	TCF7L2	TCF7L2	chr10:114710	shneg	shCHD1	OK	5,55539	8,66351	0,641064	3,14293	5,00E-05	0,0004193	yes
SVOP	SVOP	SVOP	chr12:109304	shneg	shCHD1	OK	1,89204	2,95055	0,641042	2,5699	0,0001	0,00078473	yes
PLK2	PLK2	PLK2	chr5:5774980	shneg	shCHD1	OK	135,365	211,083	0,640963	3,8012	5,00E-05	0,0004193	yes
EXO1	EXO1	EXO1	chr1:2420114	shneg	shCHD1	OK	3,92039	6,11125	0,640469	3,0148	5,00E-05	0,0004193	yes
LIG1	LIG1	LIG1	chr19:486187	shneg	shCHD1	OK	19,2346	29,9636	0,639511	3,45555	5,00E-05	0,0004193	yes
MCM6	MCM6	MCM6	chr2:1365971	shneg	shCHD1	OK	25,9561	40,4173	0,638902	3,56371	5,00E-05	0,0004193	yes
AHR	AHR	AHR	chr7:1733827	shneg	shCHD1	OK	0,49452	0,769953	0,63874	1,97644	0,0012	0,00680293	yes
TUBB2A	TUBB2A	TUBB2A	chr6:3153901	shneg	shCHD1	OK	23,7162	36,9254	0,638739	3,31062	5,00E-05	0,0004193	yes
ESCO2	ESCO2	ESCO2	chr8:2763205	shneg	shCHD1	OK	2,41417	3,757	0,638054	2,7939	5,00E-05	0,0004193	yes
DNMT3B	DNMT3B	DNMT3B	chr20:313501	shneg	shCHD1	OK	2,33468	3,63258	0,637772	2,86934	5,00E-05	0,0004193	yes
BCL2L15	BCL2L15	BCL2L15	chr1:1143564	shneg	shCHD1	OK	1,14672	1,7838	0,637436	1,70464	0,0041	0,0189794	yes
IER5	IER5	IER5	chr1:1810576	shneg	shCHD1	OK	3,89323	6,05124	0,636262	2,86522	5,00E-05	0,0004193	yes
IL17RB	IL17RB	IL17RB	chr3:5388057	shneg	shCHD1	OK	2,10048	3,26386	0,63586	2,1861	0,0003	0,0020738	yes
CEP72	CEP72	CEP72	chr5:612404-	shneg	shCHD1	OK	5,57636	8,66334	0,635598	3,02407	5,00E-05	0,0004193	yes
CBX1	CBX1	CBX1	chr17:461474	shneg	shCHD1	OK	55,7985	86,6706	0,635318	3,59487	5,00E-05	0,0004193	yes
C11orf82	C11orf82	C11orf82	chr11:826127	shneg	shCHD1	OK	2,60596	4,04305	0,633628	2,89741	5,00E-05	0,0004193	yes
WIPF2	WIPF2	WIPF2	chr17:383755	shneg	shCHD1	OK	16,5256	25,6355	0,63344	3,52574	5,00E-05	0,0004193	yes
SOWAHC	SOWAHC	SOWAHC	chr2:1103719	shneg	shCHD1	OK	0,730673	1,13326	0,633181	2,02103	0,00105	0,00607369	yes
PANX2	PANX2	PANX2	chr22:506091	shneg	shCHD1	OK	7,14995	11,0878	0,632963	3,11726	5,00E-05	0,0004193	yes
FAM101B	FAM101B	FAM101B	chr17:289770	shneg	shCHD1	OK	18,217	28,2369	0,632296	3,43922	5,00E-05	0,0004193	yes
POLQ	POLQ	POLQ	chr3:1211502	shneg	shCHD1	OK	4,88027	7,56426	0,632237	3,34051	5,00E-05	0,0004193	yes
HILPDA	HILPDA	HILPDA	chr7:1280958	shneg	shCHD1	OK	10,5152	16,2968	0,632119	3,00498	5,00E-05	0,0004193	yes
UPP1	UPP1	UPP1	chr7:4812835	shneg	shCHD1	OK	54,1882	83,9633	0,63178	3,53235	5,00E-05	0,0004193	yes
RAD54B	RAD54B	RAD54B	chr8:9538418	shneg	shCHD1	OK	2,60723	4,03886	0,631432	1,7304	0,0038	0,0178336	yes
GABARAPL1	GABARAPL1	GABARAPL1	chr12:103654	shneg	shCHD1	OK	43,6486	67,6099	0,631297	3,47219	5,00E-05	0,0004193	yes
ALS2CL	ALS2CL	ALS2CL	chr3:4671048	shneg	shCHD1	OK	0,48936	0,757901	0,631112	1,52787	0,0052	0,0231261	yes
PRKD3	PRKD3	PRKD3	chr2:3747764	shneg	shCHD1	OK	17,4437	27,0152	0,63107	3,52891	5,00E-05	0,0004193	yes
RBPMS	RBPMS	RBPMS	chr8:3023963	shneg	shCHD1	OK	8,09853	12,5188	0,628362	2,72338	5,00E-05	0,0004193	yes

EHD1	EHD1	EHD1	chr11:646202	shneg	shCHD1	OK	12,3175	19,0207	0,626862	3,29919	5,00E-05	0,0004193	yes
FANCI	FANCI	FANCI	chr15:897871	shneg	shCHD1	OK	19,1628	29,5901	0,62681	2,51911	5,00E-05	0,0004193	yes
SHC2	SHC2	SHC2	chr19:416582	shneg	shCHD1	OK	5,77829	8,9214	0,626627	3,02056	5,00E-05	0,0004193	yes
MIPEP	MIPEP	MIPEP	chr13:243043	shneg	shCHD1	OK	8,90001	13,7377	0,626259	3,00629	5,00E-05	0,0004193	yes
CDCA2	CDCA2	CDCA2	chr8:2531651	shneg	shCHD1	OK	3,54818	5,47506	0,625794	2,98482	5,00E-05	0,0004193	yes
JUP	JUP	JUP	chr17:399108	shneg	shCHD1	OK	21,759	33,5447	0,624475	3,40473	5,00E-05	0,0004193	yes
NBEA	NBEA	NBEA	chr13:355164	shneg	shCHD1	OK	9,41061	14,5031	0,624002	2,20074	0,00025	0,00176854	yes
CCDC56	CCDC56	CCDC56	chr17:409496	shneg	shCHD1	OK	39,0339	60,1055	0,622768	3,08407	5,00E-05	0,0004193	yes
HOMER1	HOMER1	HOMER1	chr5:7866978	shneg	shCHD1	OK	1,44485	2,22333	0,621809	2,46616	5,00E-05	0,0004193	yes
DEPDC1B	DEPDC1B	DEPDC1B	chr5:5989273	shneg	shCHD1	OK	10,1937	15,6819	0,621427	3,1084	5,00E-05	0,0004193	yes
COLQ	COLQ	COLQ	chr3:1549163	shneg	shCHD1	OK	0,749188	1,15218	0,620964	1,5611	0,00505	0,0226106	yes
OVCA2	OVCA2	OVCA2	chr17:193343	shneg	shCHD1	OK	14,5033	22,3028	0,620845	1,75863	0,0024	0,0121606	yes
DNA2	DNA2	DNA2	chr10:701738	shneg	shCHD1	OK	4,62781	7,11469	0,620472	3,0192	5,00E-05	0,0004193	yes
XKR5	XKR5	XKR5	chr8:6666040	shneg	shCHD1	OK	0,664539	1,02141	0,620131	1,93647	0,00125	0,00702633	yes
SLC19A1	SLC19A1	SLC19A1	chr21:469346	shneg	shCHD1	OK	4,34215	6,66498	0,61819	2,73706	5,00E-05	0,0004193	yes
CBX2	CBX2	CBX2	chr17:777519	shneg	shCHD1	OK	5,1757	7,92681	0,614985	2,5459	5,00E-05	0,0004193	yes
TAGLN2	TAGLN2	TAGLN2	chr1:1598879	shneg	shCHD1	OK	37,3736	57,2238	0,614597	3,25379	5,00E-05	0,0004193	yes
MCM4	MCM4	MCM4	chr8:4887276	shneg	shCHD1	OK	36,2517	55,4635	0,61349	3,53844	5,00E-05	0,0004193	yes
GINS1	GINS1	GINS1	chr20:253883	shneg	shCHD1	OK	6,2003	9,4858	0,61343	2,98862	5,00E-05	0,0004193	yes
NFKBIE	NFKBIE	NFKBIE	chr6:4422590	shneg	shCHD1	OK	6,0943	9,32156	0,613111	2,94402	5,00E-05	0,0004193	yes
CPNE9	CPNE9	CPNE9	chr3:9745509	shneg	shCHD1	OK	2,05159	3,13794	0,613075	2,08274	0,00065	0,0040329	yes
MIS12	MIS12	MIS12	chr17:539024	shneg	shCHD1	OK	5,10427	7,80544	0,612776	2,87746	5,00E-05	0,0004193	yes
CDX2	CDX2	CDX2	chr13:285362	shneg	shCHD1	OK	2,32739	3,55863	0,61261	2,31064	5,00E-05	0,0004193	yes
STAT5B	STAT5B	STAT5B	chr17:403511	shneg	shCHD1	OK	11,5481	17,6563	0,612526	3,31244	5,00E-05	0,0004193	yes
LEPREL4	LEPREL4	LEPREL4	chr17:399582	shneg	shCHD1	OK	25,9556	39,6692	0,611974	3,31768	5,00E-05	0,0004193	yes
ETV4	ETV4	ETV4	chr17:416052	shneg	shCHD1	OK	21,0385	32,1532	0,61193	3,21981	5,00E-05	0,0004193	yes
PIWIL4	PIWIL4	PIWIL4	chr11:943004	shneg	shCHD1	OK	1,87165	2,85986	0,611633	2,40537	0,0001	0,00078473	yes
DTL	DTL	DTL	chr1:2121137	shneg	shCHD1	OK	7,14972	10,9221	0,611292	2,32851	0,0001	0,00078473	yes
COASY	COASY	COASY	chr17:407140	shneg	shCHD1	OK	31,2699	47,7627	0,611111	3,2014	5,00E-05	0,0004193	yes
EIF5A	EIF5A	EIF5A	chr17:721031	shneg	shCHD1	OK	151,847	231,93	0,611069	3,50348	5,00E-05	0,0004193	yes
MSL1	MSL1	MSL1	chr17:382787	shneg	shCHD1	OK	25,3184	38,6385	0,609852	3,39906	5,00E-05	0,0004193	yes
APOC1	APOC1	APOC1	chr19:454179	shneg	shCHD1	OK	40,1932	61,2743	0,608331	2,8116	5,00E-05	0,0004193	yes
NFKB2	NFKB2	NFKB2	chr10:104154	shneg	shCHD1	OK	21,5579	32,8448	0,60745	3,2685	5,00E-05	0,0004193	yes
CENPH	CENPH	CENPH	chr5:6848537	shneg	shCHD1	OK	17,629	26,8509	0,607023	2,99067	5,00E-05	0,0004193	yes
DLC1	DLC1	DLC1	chr8:1294087	shneg	shCHD1	OK	6,91712	10,5343	0,606854	3,18032	5,00E-05	0,0004193	yes
DENND4A	DENND4A	DENND4A	chr15:659529	shneg	shCHD1	OK	3,732	5,68217	0,606492	3,05172	5,00E-05	0,0004193	yes
PHTF2	PHTF2	PHTF2	chr7:7742810	shneg	shCHD1	OK	17,0579	25,967	0,606239	3,31542	5,00E-05	0,0004193	yes
ESRG	ESRG	ESRG	chr3:5415669	shneg	shCHD1	OK	49,1988	74,8764	0,605887	2,85121	5,00E-05	0,0004193	yes
ATP9A	ATP9A	ATP9A	chr20:502133	shneg	shCHD1	OK	0,896831	1,36489	0,605873	2,5198	5,00E-05	0,0004193	yes
HSP90AA1	HSP90AA1	HSP90AA1	chr14:102547	shneg	shCHD1	OK	228,897	348,291	0,605598	3,62607	5,00E-05	0,0004193	yes
WDR76	WDR76	WDR76	chr15:441191	shneg	shCHD1	OK	5,67372	8,63176	0,605358	2,9939	5,00E-05	0,0004193	yes
FAM134C	FAM134C	FAM134C	chr17:407315	shneg	shCHD1	OK	16,0553	24,4231	0,605199	2,38412	5,00E-05	0,0004193	yes
PHF21B	PHF21B	PHF21B	chr22:452770	shneg	shCHD1	OK	6,92758	10,5249	0,603378	3,01363	5,00E-05	0,0004193	yes
TNFAIP2	TNFAIP2	TNFAIP2	chr14:103592	shneg	shCHD1	OK	7,94499	12,0685	0,603129	3,11167	5,00E-05	0,0004193	yes
MATN2	MATN2	MATN2	chr8:9888131	shneg	shCHD1	OK	1,60392	2,43597	0,602899	2,45927	5,00E-05	0,0004193	yes
TMEM106A	TMEM106A	TMEM106A	chr17:413638	shneg	shCHD1	OK	2,83352	4,30078	0,602002	2,59591	5,00E-05	0,0004193	yes
KIAA0101	KIAA0101	KIAA0101	chr15:646572	shneg	shCHD1	OK	31,6504	48,0307	0,60173	3,176	5,00E-05	0,0004193	yes
UBXN8	UBXN8	UBXN8	chr8:3060168	shneg	shCHD1	OK	3,25326	4,93121	0,600057	2,08305	0,0003	0,0020738	yes
ZBTB33	ZBTB33	ZBTB33	chrX:1193846	shneg	shCHD1	OK	6,08869	9,22231	0,598996	3,08149	5,00E-05	0,0004193	yes
CENPV	CENPV	CENPV	chr17:162458	shneg	shCHD1	OK	11,2466	17,0266	0,598304	2,78271	5,00E-05	0,0004193	yes
ARL4D	ARL4D	ARL4D	chr17:414763	shneg	shCHD1	OK	2,71554	4,11021	0,597974	2,02455	0,00045	0,00293585	yes
RNF19A	RNF19A	RNF19A	chr8:1012692	shneg	shCHD1	OK	2,53999	3,84318	0,597476	2,77312	5,00E-05	0,0004193	yes
STRC	STRC	STRC	chr15:438917	shneg	shCHD1	OK	0,687953	1,04043	0,596803	1,99932	0,0006	0,00377139	yes
CDK1	CDK1	CDK1	chr10:625380	shneg	shCHD1	OK	40,3799	61,0495	0,596342	3,24806	5,00E-05	0,0004193	yes
PLEKHG3	PLEKHG3	PLEKHG3	chr14:651711	shneg	shCHD1	OK	4,566	6,90092	0,595857	2,92501	5,00E-05	0,0004193	yes
FANCD2	FANCD2	FANCD2	chr3:1006811	shneg	shCHD1	OK	9,16049	13,8438	0,595738	3,12619	5,00E-05	0,0004193	yes
KLHL29	KLHL29	KLHL29	chr2:2360829	shneg	shCHD1	OK	0,957695	1,44698	0,595407	2,17659	0,0001	0,00078473	yes
NDUFA1	NDUFA1	NDUFA1	chrX:1190044	shneg	shCHD1	OK	242,804	366,767	0,595074	3,05479	5,00E-05	0,0004193	yes
SNED1	SNED1	SNED1	chr2:2419382	shneg	shCHD1	OK	2,3224	3,50794	0,595005	1,79585	0,00285	0,0140225	yes
UBE2T	UBE2T	UBE2T	chr1:2023007	shneg	shCHD1	OK	26,0707	39,3776	0,594947	2,87989	5,00E-05	0,0004193	yes
BSN	BSN	BSN	chr3:4959192	shneg	shCHD1	OK	3,18381	4,80847	0,594823	3,18684	5,00E-05	0,0004193	yes
FRAS1	FRAS1	FRAS1	chr4:7897872	shneg	shCHD1	OK	4,92592	7,4392	0,594755	3,20402	5,00E-05	0,0004193	yes
SYP	SYP	SYP	chrX:4904426	shneg	shCHD1	OK	23,8598	36,0148	0,594004	3,19625	5,00E-05	0,0004193	yes
PLEKHF2	PLEKHF2	PLEKHF2	chr8:9614594	shneg	shCHD1	OK	2,00759	3,03008	0,593889	2,31528	0,0001	0,00078473	yes
C15orf37	C15orf37	C15orf37	chr15:801358	shneg	shCHD1	OK	4,51795	6,81799	0,593679	1,71739	0,00335	0,0160296	yes
LHFPL2	LHFPL2	LHFPL2	chr5:7778103	shneg	shCHD1	OK	3,72787	5,62562	0,593659	2,866	5,00E-05	0,0004193	yes
ATP5G1	ATP5G1	ATP5G1	chr17:469701	shneg	shCHD1	OK	112,311	169,352	0,592525	3,12649	5,00E-05	0,0004193	yes
C2orf76	C2orf76	C2orf76	chr2:1200600	shneg	shCHD1	OK	3,40196	5,12937	0,592416	1,70397	0,00325	0,0156858	yes
RAP1GAP	RAP1GAP	RAP1GAP	chr1:2192270	shneg	shCHD1	OK	3,57125	5,38459	0,592407	2,75315	5,00E-05	0,0004193	yes
ENOX2	ENOX2	ENOX2	chrX:1297573	shneg	shCHD1	OK	7,52681	11,3414	0,591486	3,04783	5,00E-05	0,0004193	yes
PTP4A3	PTP4A3	PTP4A3	chr8:1424320	shneg	shCHD1	OK	11,2482	16,9426	0,590963	2,79761	5,00E-05	0,0004193	yes
CASP3	CASP3	CASP3	chr4:1855488	shneg	shCHD1	OK	25,3662	38,2029	0,590777	3,17959	5,00E-05	0,0004193	yes
SPRED1	SPRED1	SPRED1	chr15:385450	shneg	shCHD1	OK	5,09552	7,6721	0,590392	3,07865	5,00E-05	0,0004193	yes
KAT2A	KAT2A	KAT2A	chr17:402651	shneg	shCHD1	OK	27,624	41,585	0,59014	3,26009	5,00E-05	0,0004193	yes
KIFC1	KIFC1	KIFC1	chr6_ssto_ha	shneg	shCHD1	OK	32,2872	48,6049	0,59014	3,2648	5,00E-05	0,0004193	yes
KIAA0664	KIAA0664	KIAA0664	chr17:259267	shneg	shCHD1	OK	15,6275	23,5207	0,589844	3,25175	5,00E-05	0,0004193	yes
FAM60A	FAM60A	FAM60A	chr12:314335	shneg	shCHD1	OK	26,9864	40,608	0,589529	3,25607	5,00E-05	0,0004193	yes
IL13RA1	IL13RA1	IL13RA1	chrX:1178615	shneg	shCHD1	OK	20,0839	30,2028	0,588642	3,23702	5,00E-05	0,0004193	yes
PDE7A	PDE7A	PDE7A	chr8:6662656	shneg	shCHD1	OK	5,0921	7,65507	0,588155	3,04672	5,00E-05	0,0004193	yes
PSTPIP2	PSTPIP2	PSTPIP2	chr18:435635	shneg	shCHD1	OK	2,48509	3,73429	0,587535	2,48843	5,00E-05	0,0004193	yes
CDCA5	CDCA5	CDCA5	chr11:648449	shneg	shCHD1	OK	26,031	39,0976	0,586846	3,19009	5,00E-05	0,0004193	yes
CENPJ	CENPJ	CENPJ	chr13:254564	shneg	shCHD1	OK	4,74411	7,12447	0,586645	2,93073	5,00E-05	0,0004193	yes
PCNA	PCNA	PCNA	chr20:509559	shneg	shCHD1	OK	33,776	50,71	0,586271	2,62854	5,00E-05	0,0004193	yes
PPP3R1	PPP3R1	PPP3R1	chr2:6840598	shneg	shCHD1	OK	15,8304	23,7654	0,586163	3,10858	5,00E-05	0,0004193	yes
TMEM101	TMEM101	TMEM101	chr17:420885	shneg	shCHD1	OK	27,3483	41,0029	0,584272	3,05112	5,00E-05	0,0004193	yes
COPS3	COPS3	COPS3	chr17:171499	shneg	shCHD1	OK	21,9652	32,9193	0,583714	3,03987	5,00E-05	0,0004193	yes
MTERFD1	MTERFD1	MTERFD1	chr8:9725164	shneg	shCHD1	OK	5,54551	8,30265	0,582254	2,42672	0,0001	0,00078473	yes
C8orf38	C8orf38	C8orf38	chr8:9603721	shneg	shCHD1	OK	1,95021	2,9181	0,581401	1,82091	0,00265	0,0132017	yes
PKP4	PKP4	PKP4	chr2:1593134	shneg	shCHD1	OK	26,4285	39,5226	0,580581	3,27698	5,00E-05	0,0004193	yes
SYTL3	SYTL3	SYTL3	chr6:1590710	shneg	shCHD1	OK	2,3287	3,48195	0,580371	2,13253	0,0003	0,0020738	yes
Sep 08	Sep 08	Sep 08	chr5:1320831	shneg	shCHD1	OK	18,6606	27,8728	0,578867	3,14774	5,00E-05	0,0004193	yes
SNX10	SNX10	SNX10	chr7:2633151	shneg	shCHD1	OK	3,94417	5,8763	0,575188	2,64863	5,00E-05	0,0004193	yes
TBC1D12	TBC1D12	TBC1D12	chr10:961621	shneg	shCHD1	OK	3,37537	5,02472	0,573998	2,78384	5,00E-05	0,0004193	yes

NXN	NXN	NXN	chr17:702552 shneg	shCHD1	OK	5,14587	7,6591	0,57376	2,69519	5,00E-05	0,0004193	yes
PIK3AP1	PIK3AP1	PIK3AP1	chr10:983530 shneg	shCHD1	OK	1,79332	2,668	0,573129	2,56104	5,00E-05	0,0004193	yes
POLE2	POLE2	POLE2	chr14:501102 shneg	shCHD1	OK	5,35133	7,96134	0,573114	2,59187	5,00E-05	0,0004193	yes
PRKCH	PRKCH	PRKCH	chr14:617885 shneg	shCHD1	OK	2,01748	3,00146	0,573112	2,38151	5,00E-05	0,0004193	yes
NQO2	NQO2	NQO2	chr6:3000066 shneg	shCHD1	OK	15,3199	22,7883	0,572881	2,74738	5,00E-05	0,0004193	yes
GPRIN1	GPRIN1	GPRIN1	chr5:1760228 shneg	shCHD1	OK	11,8495	17,6173	0,572163	3,05167	5,00E-05	0,0004193	yes
TRAF2	TRAF2	TRAF2	chr9:1397809 shneg	shCHD1	OK	15,9456	23,7015	0,571818	2,93202	5,00E-05	0,0004193	yes
MSRA	MSRA	MSRA	chr8:9911829 shneg	shCHD1	OK	3,72234	5,53107	0,571351	2,07753	0,00025	0,00176854	yes
NCAPH	NCAPH	NCAPH	chr2:9700148 shneg	shCHD1	OK	8,11066	12,05	0,571148	2,9738	5,00E-05	0,0004193	yes
MRPL15	MRPL15	MRPL15	chr8:5504778 shneg	shCHD1	OK	22,9908	34,1453	0,570632	2,97166	5,00E-05	0,0004193	yes
TUBG2	TUBG2	TUBG2	chr17:408112 shneg	shCHD1	OK	8,82643	13,1069	0,570422	2,71239	5,00E-05	0,0004193	yes
DUSP6	DUSP6	DUSP6	chr12:897418 shneg	shCHD1	OK	62,5602	92,883	0,57017	3,29707	5,00E-05	0,0004193	yes
FAM57B	FAM57B	FAM57B	chr16:300346 shneg	shCHD1	OK	4,64749	6,8987	0,569875	2,51354	5,00E-05	0,0004193	yes
SLC48A1	SLC48A1	SLC48A1	chr12:481669 shneg	shCHD1	OK	30,3281	45,018	0,569844	2,40983	5,00E-05	0,0004193	yes
TUBA1B	TUBA1B	TUBA1B	chr12:495215 shneg	shCHD1	OK	291,507	432,636	0,569623	3,38858	5,00E-05	0,0004193	yes
PPFIA4	PPFIA4	PPFIA4	chr1:2030203 shneg	shCHD1	OK	1,45447	2,15772	0,569013	2,40462	5,00E-05	0,0004193	yes
RASL10B	RASL10B	RASL10B	chr17:340586 shneg	shCHD1	OK	2,88212	4,27457	0,568648	2,57288	5,00E-05	0,0004193	yes
MLLT3	MLLT3	MLLT3	chr9:2034496 shneg	shCHD1	OK	5,36137	7,95134	0,568596	2,72754	5,00E-05	0,0004193	yes
GPS2	GPS2	GPS2	chr17:721597 shneg	shCHD1	OK	31,9318	47,3506	0,568386	2,92036	5,00E-05	0,0004193	yes
TMEM14B	TMEM14B	TMEM14B	chr6:1074799 shneg	shCHD1	OK	27,064	40,1318	0,56837	2,8048	5,00E-05	0,0004193	yes
PMS2	PMS2	PMS2	chr7:6012869 shneg	shCHD1	OK	8,21896	12,1872	0,568345	2,80063	5,00E-05	0,0004193	yes
PKI55	PKI55	PKI55	chr2:2170816 shneg	shCHD1	OK	5,86533	8,69696	0,568298	2,72354	5,00E-05	0,0004193	yes
CDT1	CDT1	CDT1	chr16:888701 shneg	shCHD1	OK	16,9363	25,1083	0,568049	2,99397	5,00E-05	0,0004193	yes
CCNG1	CCNG1	CCNG1	chr5:1628645 shneg	shCHD1	OK	61,9919	91,838	0,567011	3,21894	5,00E-05	0,0004193	yes
SLC7A2	SLC7A2	SLC7A2	chr8:1735459 shneg	shCHD1	OK	0,555581	0,823003	0,566901	1,97341	0,0009	0,00533451	yes
SKA1	SKA1	SKA1	chr18:479013 shneg	shCHD1	OK	3,18828	4,72075	0,566237	2,56819	5,00E-05	0,0004193	yes
IL34	IL34	IL34	chr16:706137 shneg	shCHD1	OK	3,10729	4,60057	0,566156	2,0828	0,0003	0,0020738	yes
RANBP9	RANBP9	RANBP9	chr6:1362172 shneg	shCHD1	OK	9,45731	13,9955	0,565457	2,87387	5,00E-05	0,0004193	yes
RIMS2	RIMS2	RIMS2	chr8:1045129 shneg	shCHD1	OK	0,867707	1,28347	0,564768	2,19377	0,00015	0,00112331	yes
FAM65A	FAM65A	FAM65A	chr16:675627 shneg	shCHD1	OK	15,3461	22,6839	0,563796	3,03802	5,00E-05	0,0004193	yes
PTPN21	PTPN21	PTPN21	chr14:889321 shneg	shCHD1	OK	1,38342	2,04152	0,56141	2,50763	5,00E-05	0,0004193	yes
MELK	MELK	MELK	chr9:3657285 shneg	shCHD1	OK	15,1217	22,3042	0,560696	2,87473	5,00E-05	0,0004193	yes
LUZP1	LUZP1	LUZP1	chr1:2341051 shneg	shCHD1	OK	13,0951	19,2999	0,559564	3,1373	5,00E-05	0,0004193	yes
PRELID1	PRELID1	PRELID1	chr5:1767308 shneg	shCHD1	OK	73,8617	108,841	0,559318	2,18401	0,0001	0,00078473	yes
EIF4A1	EIF4A1	EIF4A1	chr17:746530 shneg	shCHD1	OK	213,558	314,689	0,559296	3,13654	5,00E-05	0,0004193	yes
XRCC3	XRCC3	XRCC3	chr14:104095 shneg	shCHD1	OK	12,6113	18,5774	0,558827	1,62412	0,00515	0,0229918	yes
MIS18A	MIS18A	MIS18A	chr21:336405 shneg	shCHD1	OK	7,23326	10,6435	0,557249	2,62524	5,00E-05	0,0004193	yes
LOC10050557	LOC10050557	LOC10050557	chr17:370814 shneg	shCHD1	OK	2,19503	3,22987	0,557237	2,41888	5,00E-05	0,0004193	yes
CENPW	CENPW	CENPW	chr6:1266612 shneg	shCHD1	OK	27,1787	39,9864	0,55703	2,59433	5,00E-05	0,0004193	yes
TMEM198	TMEM198	TMEM198	chr2:2204087 shneg	shCHD1	OK	6,87037	10,1074	0,556956	2,70413	5,00E-05	0,0004193	yes
LURAP1L	LURAP1L	LURAP1L	chr9:1277501 shneg	shCHD1	OK	2,17124	3,19395	0,55682	2,1487	0,00015	0,00112331	yes
GEMIN4	GEMIN4	GEMIN4	chr17:647660 shneg	shCHD1	OK	10,937	16,0626	0,554487	2,91307	5,00E-05	0,0004193	yes
UCHL3	UCHL3	UCHL3	chr13:761239 shneg	shCHD1	OK	13,5746	19,9311	0,554109	2,50901	0,00015	0,00112331	yes
DDX12P	DDX12P	DDX12P	chr12:957028 shneg	shCHD1	OK	3,40869	5,00387	0,553824	2,63189	5,00E-05	0,0004193	yes
HOXA1	HOXA1	HOXA1	chr7:2713261 shneg	shCHD1	OK	1,24638	1,82786	0,552407	1,61727	0,00505	0,0226106	yes
CCDC64	CCDC64	CCDC64	chr12:120427 shneg	shCHD1	OK	10,2043	14,9592	0,551854	2,82231	5,00E-05	0,0004193	yes
EIF1	EIF1	EIF1	chr17:398451 shneg	shCHD1	OK	232,986	341,328	0,550917	3,21751	5,00E-05	0,0004193	yes
KIF20A	KIF20A	KIF20A	chr5:1375144 shneg	shCHD1	OK	23,2837	34,1025	0,550561	2,39829	0,0001	0,00078473	yes
MESDC1	MESDC1	MESDC1	chr15:812932 shneg	shCHD1	OK	11,2762	16,5143	0,550432	2,82785	5,00E-05	0,0004193	yes
SOX2	SOX2	SOX2	chr3:1813281 shneg	shCHD1	OK	3,59077	5,25784	0,550177	2,37801	0,0001	0,00078473	yes
NUAK2	NUAK2	NUAK2	chr1:2052711 shneg	shCHD1	OK	7,33553	10,7356	0,549426	2,73673	5,00E-05	0,0004193	yes
ANP32E	ANP32E	ANP32E	chr1:1501907 shneg	shCHD1	OK	30,3854	44,4675	0,549374	3,06463	5,00E-05	0,0004193	yes
NT5C3L	NT5C3L	NT5C3L	chr17:399813 shneg	shCHD1	OK	53,5161	78,3142	0,549301	2,9759	5,00E-05	0,0004193	yes
RFC2	RFC2	RFC2	chr7:7364583 shneg	shCHD1	OK	27,3008	39,9514	0,549301	2,89299	5,00E-05	0,0004193	yes
NNMT	NNMT	NNMT	chr11:114166 shneg	shCHD1	OK	38,8228	56,7856	0,54862	2,95249	5,00E-05	0,0004193	yes
NUDT1	NUDT1	NUDT1	chr7:2281856 shneg	shCHD1	OK	40,4625	59,1236	0,547148	2,63066	5,00E-05	0,0004193	yes
CCDC34	CCDC34	CCDC34	chr11:273600 shneg	shCHD1	OK	11,1703	16,3129	0,546336	2,59987	5,00E-05	0,0004193	yes
BASP1	BASP1	BASP1	chr5:1721774 shneg	shCHD1	OK	51,1016	74,6003	0,545814	3,01638	5,00E-05	0,0004193	yes
C16orf57	C16orf57	C16orf57	chr16:580352 shneg	shCHD1	OK	42,2246	61,6289	0,545524	3,02653	5,00E-05	0,0004193	yes
RBMS1	RBMS1	RBMS1	chr2:1611286 shneg	shCHD1	OK	9,44074	13,7768	0,545274	2,87076	5,00E-05	0,0004193	yes
CYR61	CYR61	CYR61	chr1:8604644 shneg	shCHD1	OK	86,8856	126,787	0,545217	3,13655	5,00E-05	0,0004193	yes
BRSK2	BRSK2	BRSK2	chr11:141112 shneg	shCHD1	OK	5,41764	7,90554	0,545199	2,67606	5,00E-05	0,0004193	yes
GGH	GGH	GGH	chr8:6392763 shneg	shCHD1	OK	19,4972	28,4363	0,544466	2,73952	5,00E-05	0,0004193	yes
PLEKHO1	PLEKHO1	PLEKHO1	chr1:1501221 shneg	shCHD1	OK	2,04484	2,98041	0,543525	1,64233	0,00585	0,0255919	yes
E2F3	E2F3	E2F3	chr6:2040213 shneg	shCHD1	OK	8,16315	11,8976	0,543474	2,7988	5,00E-05	0,0004193	yes
QPCT	QPCT	QPCT	chr2:3757175 shneg	shCHD1	OK	127,389	185,445	0,541754	3,12641	5,00E-05	0,0004193	yes
RAP1GAP2	RAP1GAP2	RAP1GAP2	chr17:269973 shneg	shCHD1	OK	3,46089	5,0381	0,541737	2,67494	5,00E-05	0,0004193	yes
PUS7	PUS7	PUS7	chr7:1050969 shneg	shCHD1	OK	17,7219	25,7943	0,541516	2,92265	5,00E-05	0,0004193	yes
HINT1	HINT1	HINT1	chr5:1304948 shneg	shCHD1	OK	275,048	400,102	0,540688	3,09392	5,00E-05	0,0004193	yes
RRM1	RRM1	RRM1	chr11:411592 shneg	shCHD1	OK	26,7985	38,9646	0,540009	2,98091	5,00E-05	0,0004193	yes
TFR2	TFR2	TFR2	chr7:1002180 shneg	shCHD1	OK	3,7845	5,50203	0,53986	2,43478	5,00E-05	0,0004193	yes
MEST	MEST	MEST	chr7:1301260 shneg	shCHD1	OK	4,05668	5,89658	0,53958	1,50407	0,00945	0,0380871	yes
SRPK1	SRPK1	SRPK1	chr6:3580081 shneg	shCHD1	OK	20,22	29,3863	0,539364	2,99579	5,00E-05	0,0004193	yes
ZNF519	ZNF519	ZNF519	chr18:140759 shneg	shCHD1	OK	1,36122	1,97817	0,539266	1,7017	0,0035	0,0166331	yes
PFAS	PFAS	PFAS	chr17:815259 shneg	shCHD1	OK	9,46852	13,7581	0,539076	2,87273	5,00E-05	0,0004193	yes
HLA-E	HLA-E	HLA-E	chr6_ssto_ha shneg	shCHD1	OK	64,5776	93,7792	0,538233	3,07651	5,00E-05	0,0004193	yes
ERCC6L	ERCC6L	ERCC6L	chrX:7140152 shneg	shCHD1	OK	6,33504	9,19217	0,537051	2,20782	0,0003	0,0020738	yes
RPS6KA5	RPS6KA5	RPS6KA5	chr14:913371 shneg	shCHD1	OK	4,20723	6,09689	0,535204	2,57183	5,00E-05	0,0004193	yes
AARSD1	AARSD1	AARSD1	chr17:411025 shneg	shCHD1	OK	13,6673	19,8037	0,535038	2,65404	5,00E-05	0,0004193	yes
CAMK2N2	CAMK2N2	CAMK2N2	chr3:1839674 shneg	shCHD1	OK	13,4876	19,5381	0,534659	2,17535	0,00025	0,00176854	yes
MSMP	MSMP	MSMP	chr9:3574927 shneg	shCHD1	OK	23,9888	34,7488	0,534599	1,5585	0,0102	0,0404429	yes
E2F2	E2F2	E2F2	chr1:2383291 shneg	shCHD1	OK	2,97549	4,30938	0,534351	2,57413	5,00E-05	0,0004193	yes
HSD17B1	HSD17B1	HSD17B1	chr17:407039 shneg	shCHD1	OK	7,02503	10,1728	0,534144	2,60294	5,00E-05	0,0004193	yes
PIF1	PIF1	PIF1	chr15:651078 shneg	shCHD1	OK	5,23059	7,57365	0,534014	2,55079	5,00E-05	0,0004193	yes
CALML4	CALML4	CALML4	chr15:684830 shneg	shCHD1	OK	2,95388	4,27539	0,533442	2,55156	5,00E-05	0,0004193	yes
DIO3OS	DIO3OS	DIO3OS	chr14:102018 shneg	shCHD1	OK	0,953759	1,38039	0,533383	1,64365	0,00575	0,0252576	yes
REEP4	REEP4	REEP4	chr8:2199553 shneg	shCHD1	OK	13,3616	19,3384	0,533371	2,60208	5,00E-05	0,0004193	yes
RPS6KA2	RPS6KA2	RPS6KA2	chr6:1668228 shneg	shCHD1	OK	0,564123	0,816218	0,532945	1,63887	0,0038	0,0178336	yes
NKRF	NKRF	NKRF	chrX:1187222 shneg	shCHD1	OK	5,60149	8,09917	0,531963	2,55735	5,00E-05	0,0004193	yes
PASK	PASK	PASK	chr2:2420455 shneg	shCHD1	OK	6,83656	9,88256	0,531613	2,70517	5,00E-05	0,0004193	yes
LOC285758	LOC285758	LOC285758	chr6:1141891 shneg	shCHD1	OK	4,11155	5,94102	0,531029	2,48776	5,00E-05	0,0004193	yes
MKI67	MKI67	MKI67	chr10:129894 shneg	shCHD1	OK	35,2656	50,9565	0,531002	3,15944	5,00E-05	0,0004193	yes
MPP2	MPP2	MPP2	chr17:419527 shneg	shCHD1	OK	6,18736	8,93902	0,530792	2,67935	5,00E-05	0,0004193	yes

FIGNL1	FIGNL1	FIGNL1	chr7:5051183	shneg	shCHD1	OK	11,2708	16,2825	0,530742	2,77416	5,00E-05	0,0004193	yes
RET	RET	RET	chr10:435725	shneg	shCHD1	OK	0,718444	1,03789	0,530701	1,59794	0,00685	0,0290629	yes
C1GALT1C1	C1GALT1C1	C1GALT1C1	chrX:1197595	shneg	shCHD1	OK	18,1871	26,2736	0,530699	2,71126	5,00E-05	0,0004193	yes
NAGLU	NAGLU	NAGLU	chr17:406879	shneg	shCHD1	OK	12,4483	17,9824	0,530634	2,73393	5,00E-05	0,0004193	yes
TNFRSF10B	TNFRSF10B	TNFRSF10B	chr8:2284492	shneg	shCHD1	OK	30,4435	43,963	0,530156	2,76914	5,00E-05	0,0004193	yes
FKBP4	FKBP4	FKBP4	chr12:290410	shneg	shCHD1	OK	37,4086	54,0109	0,52988	3,00522	5,00E-05	0,0004193	yes
PRIM1	PRIM1	PRIM1	chr12:571253	shneg	shCHD1	OK	9,20253	13,2778	0,528912	2,49641	5,00E-05	0,0004193	yes
FOXO3B	FOXO3B	FOXO3B	chr17:185617	shneg	shCHD1	OK	0,516373	0,744923	0,528676	1,52831	0,01005	0,0399619	yes
DLD	DLD	DLD	chr7:1075315	shneg	shCHD1	OK	32,0576	46,244	0,528598	2,9698	5,00E-05	0,0004193	yes
TFAP2A	TFAP2A	TFAP2A	chr6:1039691	shneg	shCHD1	OK	1,715	2,4726	0,527817	1,94279	0,0011	0,0063157	yes
RND2	RND2	RND2	chr17:411772	shneg	shCHD1	OK	6,04414	8,71368	0,527746	2,63721	5,00E-05	0,0004193	yes
PCP4	PCP4	PCP4	chr21:412393	shneg	shCHD1	OK	56,8921	82,0127	0,527618	2,5779	5,00E-05	0,0004193	yes
PAG1	PAG1	PAG1	chr8:8188004	shneg	shCHD1	OK	1,95432	2,81696	0,527471	2,58413	5,00E-05	0,0004193	yes
ODC1	ODC1	ODC1	chr2:1058050	shneg	shCHD1	OK	201,39	290,153	0,526824	3,11377	5,00E-05	0,0004193	yes
EZH2	EZH2	EZH2	chr7:1485044	shneg	shCHD1	OK	24,3103	35,021	0,526652	2,84197	5,00E-05	0,0004193	yes
KCNN3	KCNN3	KCNN3	chr1:1546699	shneg	shCHD1	OK	1,01461	1,46147	0,526488	2,52541	5,00E-05	0,0004193	yes
DNAJC1	DNAJC1	DNAJC1	chr10:220454	shneg	shCHD1	OK	7,95758	11,4604	0,526251	2,54315	0,0001	0,00078473	yes
GPX3	GPX3	GPX3	chr5:1503999	shneg	shCHD1	OK	2,27183	3,26982	0,525356	1,72289	0,003	0,0146313	yes
MICALL2	MICALL2	MICALL2	chr7:1473994	shneg	shCHD1	OK	4,5027	6,47896	0,524971	2,4966	5,00E-05	0,0004193	yes
C11orf75	C11orf75	C11orf75	chr11:932116	shneg	shCHD1	OK	8,35217	12,0122	0,524281	2,14592	0,0003	0,0020738	yes
FEN1	FEN1	FEN1	chr11:615601	shneg	shCHD1	OK	35,4557	50,9673	0,523555	2,87349	5,00E-05	0,0004193	yes
UBE2A	UBE2A	UBE2A	chrX:1187084	shneg	shCHD1	OK	33,9157	48,7521	0,523513	2,76898	5,00E-05	0,0004193	yes
ZNF532	ZNF532	ZNF532	chr18:565300	shneg	shCHD1	OK	5,5852	8,0246	0,52282	2,71804	5,00E-05	0,0004193	yes
FOXJ1	FOXJ1	FOXJ1	chr17:741324	shneg	shCHD1	OK	29,881	42,9198	0,522415	2,4327	5,00E-05	0,0004193	yes
LOC10013089	LOC10013089	LOC10013089	chr22:404283	shneg	shCHD1	OK	0,693409	0,99504	0,521048	1,54629	0,0091	0,036933	yes
SCRT1	SCRT1	SCRT1	chr8:1455544	shneg	shCHD1	OK	6,70213	9,616	0,520818	2,57318	5,00E-05	0,0004193	yes
RANGAP1	RANGAP1	RANGAP1	chr22:416416	shneg	shCHD1	OK	56,5903	81,1795	0,520561	2,97967	5,00E-05	0,0004193	yes
TUBA1C	TUBA1C	TUBA1C	chr12:496588	shneg	shCHD1	OK	106,094	152,172	0,520363	2,96608	5,00E-05	0,0004193	yes
KCTD11	KCTD11	KCTD11	chr17:725520	shneg	shCHD1	OK	3,70824	5,31773	0,520075	2,44227	0,0001	0,00078473	yes
PSMB3	PSMB3	PSMB3	chr17:369090	shneg	shCHD1	OK	200,437	287,37	0,519759	2,91909	5,00E-05	0,0004193	yes
IER3	IER3	IER3	chr6_ssto_ha	shneg	shCHD1	OK	125,376	179,722	0,519511	2,94536	5,00E-05	0,0004193	yes
H2AFY	H2AFY	H2AFY	chr5:1346700	shneg	shCHD1	OK	106,49	152,611	0,519137	2,97018	5,00E-05	0,0004193	yes
FLJ35282	FLJ35282	FLJ35282	chr9:2264619	shneg	shCHD1	OK	2,74784	3,93724	0,518885	2,05515	0,0008	0,0048114	yes
TRPV2	TRPV2	TRPV2	chr17:163188	shneg	shCHD1	OK	16,4027	23,4936	0,518337	2,73809	5,00E-05	0,0004193	yes
SIPA1L1	SIPA1L1	SIPA1L1	chr14:719960	shneg	shCHD1	OK	8,66071	12,4001	0,517799	2,77265	5,00E-05	0,0004193	yes
ANKS4B	ANKS4B	ANKS4B	chr16:212450	shneg	shCHD1	OK	0,912939	1,30685	0,5175	1,46474	0,0129	0,049027	yes
FOS	FOS	FOS	chr14:757454	shneg	shCHD1	OK	23,1548	33,1391	0,517226	2,52422	0,0001	0,00078473	yes
CENPK	CENPK	CENPK	chr5:6481359	shneg	shCHD1	OK	12,2553	17,537	0,517003	2,49888	5,00E-05	0,0004193	yes
RRAS2	RRAS2	RRAS2	chr11:142994	shneg	shCHD1	OK	16,2175	23,2025	0,516729	2,65738	5,00E-05	0,0004193	yes
FAM111B	FAM111B	FAM111B	chr11:588746	shneg	shCHD1	OK	6,90732	9,88232	0,516723	2,55232	5,00E-05	0,0004193	yes
PPP3CA	PPP3CA	PPP3CA	chr4:1019445	shneg	shCHD1	OK	10,3456	14,7952	0,516106	2,76685	5,00E-05	0,0004193	yes
RPL27	RPL27	RPL27	chr17:411504	shneg	shCHD1	OK	2016,59	2883,58	0,515941	3,08909	5,00E-05	0,0004193	yes
SLC1A4	SLC1A4	SLC1A4	chr2:6521557	shneg	shCHD1	OK	3,4369	4,91434	0,515889	2,47302	5,00E-05	0,0004193	yes
C1QBP	C1QBP	C1QBP	chr17:528934	shneg	shCHD1	OK	88,4398	126,311	0,514208	2,23024	5,00E-05	0,0004193	yes
HOXB8	HOXB8	HOXB8	chr17:466897	shneg	shCHD1	OK	27,2811	38,9615	0,514147	2,72325	5,00E-05	0,0004193	yes
TLR8-AS1	TLR8-AS1	TLR8-AS1	chrX:1292093	shneg	shCHD1	OK	8,537	12,1892	0,513805	2,42957	5,00E-05	0,0004193	yes
CDCA7	CDCA7	CDCA7	chr2:1742195	shneg	shCHD1	OK	24,8842	35,5283	0,513743	2,77235	5,00E-05	0,0004193	yes
C16orf48	C16orf48	C16orf48	chr16:676968	shneg	shCHD1	OK	3,13517	4,47134	0,512166	1,79127	0,0018	0,00951868	yes
DLL4	DLL4	DLL4	chr15:412215	shneg	shCHD1	OK	4,66655	6,65379	0,51182	2,46722	5,00E-05	0,0004193	yes
SF3A3	SF3A3	SF3A3	chr1:3842265	shneg	shCHD1	OK	35,1552	50,1232	0,511741	2,85035	5,00E-05	0,0004193	yes
CDKN2AIPNL	CDKN2AIPNL	CDKN2AIPNL	chr5:1337377	shneg	shCHD1	OK	28,3044	40,3539	0,511682	2,61756	5,00E-05	0,0004193	yes
ADM	ADM	ADM	chr11:103266	shneg	shCHD1	OK	45,9685	65,527	0,511445	2,75617	5,00E-05	0,0004193	yes
C21orf7	C21orf7	C21orf7	chr21:304528	shneg	shCHD1	OK	3,94279	5,61936	0,511189	2,15487	0,00015	0,00112331	yes
MYCBP	MYCBP	MYCBP	chr1:3932816	shneg	shCHD1	OK	5,50397	7,84366	0,511053	2,16678	0,00015	0,00112331	yes
RECQL4	RECQL4	RECQL4	chr8:1457366	shneg	shCHD1	OK	25,566	36,4273	0,510793	2,82697	5,00E-05	0,0004193	yes
N6AMT2	N6AMT2	N6AMT2	chr13:213030	shneg	shCHD1	OK	8,21563	11,7027	0,510395	1,97169	0,0009	0,00533451	yes
CDC34	CDC34	CDC34	chr19:531732	shneg	shCHD1	OK	38,8963	55,3715	0,509513	2,71616	5,00E-05	0,0004193	yes
ZNHIT6	ZNHIT6	ZNHIT6	chr1:8611510	shneg	shCHD1	OK	5,88593	8,37795	0,509328	2,65248	5,00E-05	0,0004193	yes
WRAP53	WRAP53	WRAP53	chr17:757171	shneg	shCHD1	OK	8,83272	12,5675	0,508764	1,98707	0,00025	0,00176854	yes
ZNF704	ZNF704	ZNF704	chr8:8154068	shneg	shCHD1	OK	6,58796	9,36999	0,508215	2,8272	5,00E-05	0,0004193	yes
CD74	CD74	CD74	chr5:1497811	shneg	shCHD1	OK	72,234	102,73	0,508105	2,83506	5,00E-05	0,0004193	yes
PSMD10	PSMD10	PSMD10	chrX:1073274	shneg	shCHD1	OK	20,0043	28,4284	0,507022	2,55086	5,00E-05	0,0004193	yes
POLA1	POLA1	POLA1	chrX:2471206	shneg	shCHD1	OK	8,20082	11,6473	0,506156	2,67532	5,00E-05	0,0004193	yes
CWC27	CWC27	CWC27	chr5:6406475	shneg	shCHD1	OK	13,6687	19,413	0,506139	2,5482	5,00E-05	0,0004193	yes
CHAF1A	CHAF1A	CHAF1A	chr19:440265	shneg	shCHD1	OK	13,5446	19,233	0,505866	2,66913	5,00E-05	0,0004193	yes
OIP5	OIP5	OIP5	chr15:416014	shneg	shCHD1	OK	10,8929	15,4667	0,505771	2,37457	5,00E-05	0,0004193	yes
MASTL	MASTL	MASTL	chr10:274437	shneg	shCHD1	OK	8,07091	11,4593	0,505713	2,57027	5,00E-05	0,0004193	yes
ZNF618	ZNF618	ZNF618	chr9:1166385	shneg	shCHD1	OK	4,92597	6,99357	0,505621	2,67955	0,00015	0,00112331	yes
GNG4	GNG4	GNG4	chr1:2357109	shneg	shCHD1	OK	12,6421	17,9468	0,505485	2,74922	5,00E-05	0,0004193	yes
DUSP10	DUSP10	DUSP10	chr1:2218747	shneg	shCHD1	OK	2,59465	3,68322	0,505429	2,01103	0,00045	0,00293585	yes
DUSP3	DUSP3	DUSP3	chr17:418434	shneg	shCHD1	OK	32,1523	45,6403	0,505389	2,86251	5,00E-05	0,0004193	yes
ITPRIPL2	ITPRIPL2	ITPRIPL2	chr16:191252	shneg	shCHD1	OK	9,4543	13,4173	0,505051	2,75141	5,00E-05	0,0004193	yes
DBF4	DBF4	DBF4	chr7:8746381	shneg	shCHD1	OK	9,01289	12,7904	0,505003	1,80884	0,0017	0,00909693	yes
RFWD3	RFWD3	RFWD3	chr16:746552	shneg	shCHD1	OK	15,8231	22,4549	0,504995	2,77584	5,00E-05	0,0004193	yes
RAB5C	RAB5C	RAB5C	chr17:402769	shneg	shCHD1	OK	71,0444	100,805	0,504778	2,84492	5,00E-05	0,0004193	yes
FANCA	FANCA	FANCA	chr16:897735	shneg	shCHD1	OK	15,2966	21,6997	0,504459	1,92752	0,0008	0,0048114	yes
COPZ2	COPZ2	COPZ2	chr17:461035	shneg	shCHD1	OK	11,9763	16,9772	0,503414	2,23928	0,0001	0,00078473	yes
BUB1	BUB1	BUB1	chr2:1113954	shneg	shCHD1	OK	18,6506	26,4207	0,502445	2,72583	5,00E-05	0,0004193	yes
PAIP2	PAIP2	PAIP2	chr5:1386775	shneg	shCHD1	OK	57,375	81,2775	0,502434	2,73673	5,00E-05	0,0004193	yes
CHRM4	CHRM4	CHRM4	chr11:464066	shneg	shCHD1	OK	3,91313	5,5425	0,502213	1,86259	0,0013	0,00726051	yes
RASSF5	RASSF5	RASSF5	chr1:2066808	shneg	shCHD1	OK	2,3553	3,33592	0,502172	2,18317	0,0002	0,00144867	yes
GSTP1	GSTP1	GSTP1	chr11:673510	shneg	shCHD1	OK	292,932	414,685	0,50145	2,9087	5,00E-05	0,0004193	yes
UHRF1	UHRF1	UHRF1	chr19:490950	shneg	shCHD1	OK	32,8623	46,5019	0,500857	2,8373	5,00E-05	0,0004193	yes
CYP27A1	CYP27A1	CYP27A1	chr2:2196464	shneg	shCHD1	OK	112,703	159,478	0,500832	2,91295	5,00E-05	0,0004193	yes
MIEN1	MIEN1	MIEN1	chr17:378854	shneg	shCHD1	OK	62,4908	88,3982	0,500372	2,59568	5,00E-05	0,0004193	yes
DTX4	DTX4	DTX4	chr11:589398	shneg	shCHD1	OK	0,79377	1,12243	0,499833	1,76002	0,00255	0,0127904	yes
FOXC1	FOXC1	FOXC1	chr6:1610680	shneg	shCHD1	OK	6,76587	9,56506	0,4995	2,46674	5,00E-05	0,0004193	yes
ZMIZ2	ZMIZ2	ZMIZ2	chr7:4478852	shneg	shCHD1	OK	18,2566	25,7997	0,498934	2,75819	5,00E-05	0,0004193	yes
B3GALT1	B3GALT1	B3GALT1	chr2:1686751	shneg	shCHD1	OK	1,73384	2,44968	0,49862	1,6073	0,00635	0,0272991	yes
RASGRP2	RASGRP2	RASGRP2	chr11:644943	shneg	shCHD1	OK	3,81402	5,38858	0,498591	2,15783	0,0003	0,0020738	yes
MSH2	MSH2	MSH2	chr2:4763026	shneg	shCHD1	OK	23,3159	32,9365	0,49837	2,7224	5,00E-05	0,0004193	yes
HMGN2	HMGN2	HMGN2	chr1:2679890	shneg	shCHD1	OK	238,071	336,188	0,497876	2,95464	5,00E-05	0,0004193	yes
PIK3C3	PIK3C3	PIK3C3	chr18:395351	shneg	shCHD1	OK	8,30689	11,727	0,497452	2,47919	5,00E-05	0,0004193	yes

GDAP1	GDAP1	GDAP1	chr8:7526261	shneg	shCHD1	OK	8,71188	12,2967	0,497211	2,5604	5,00E-05	0,0004193	yes
DSEL	DSEL	DSEL	chr18:651738	shneg	shCHD1	OK	8,63843	12,1927	0,497175	2,70944	0,0001	0,00078473	yes
TNPO3	TNPO3	TNPO3	chr7:1285942	shneg	shCHD1	OK	32,7054	46,1531	0,496897	2,8312	5,00E-05	0,0004193	yes
HAT1	HAT1	HAT1	chr2:1727789	shneg	shCHD1	OK	28,0939	39,63	0,496332	2,62267	5,00E-05	0,0004193	yes
NPEPPS	NPEPPS	NPEPPS	chr17:456084	shneg	shCHD1	OK	28,7394	40,5379	0,49624	2,80057	5,00E-05	0,0004193	yes
FCHSD2	FCHSD2	FCHSD2	chr11:725477	shneg	shCHD1	OK	8,65359	12,2032	0,495887	2,58296	5,00E-05	0,0004193	yes
RPL39	RPL39	RPL39	chrX:1189204	shneg	shCHD1	OK	1965,74	2771,91	0,495812	2,93344	5,00E-05	0,0004193	yes
PBK	PBK	PBK	chr8:2766713	shneg	shCHD1	OK	8,12392	11,4508	0,495207	2,38353	5,00E-05	0,0004193	yes
BZW2	BZW2	BZW2	chr7:1668575	shneg	shCHD1	OK	27,8889	39,3097	0,495194	2,6378	5,00E-05	0,0004193	yes
HAUS1	HAUS1	HAUS1	chr18:436842	shneg	shCHD1	OK	11,9057	16,7784	0,494954	2,30701	0,0001	0,00078473	yes
STRA6	STRA6	STRA6	chr15:744718	shneg	shCHD1	OK	14,0731	19,8316	0,494861	2,53585	5,00E-05	0,0004193	yes
PTPN14	PTPN14	PTPN14	chr1:2145220	shneg	shCHD1	OK	10,6192	14,9564	0,494093	2,81066	5,00E-05	0,0004193	yes
CDC20	CDC20	CDC20	chr1:4382462	shneg	shCHD1	OK	65,5787	92,352	0,493916	2,75527	5,00E-05	0,0004193	yes
TSR1	TSR1	TSR1	chr17:220724	shneg	shCHD1	OK	13,0397	18,3498	0,492857	2,63175	5,00E-05	0,0004193	yes
IGIP	IGIP	IGIP	chr5:1395055	shneg	shCHD1	OK	3,40049	4,78278	0,492107	2,24569	0,0001	0,00078473	yes
IQGAP1	IQGAP1	IQGAP1	chr15:909314	shneg	shCHD1	OK	49,6463	69,8193	0,491939	2,90206	5,00E-05	0,0004193	yes
AMMECR1	AMMECR1	AMMECR1	chrX:1094374	shneg	shCHD1	OK	3,87732	5,45265	0,491897	2,39534	5,00E-05	0,0004193	yes
NOC3L	NOC3L	NOC3L	chr10:960929	shneg	shCHD1	OK	7,11902	10,0091	0,491561	2,43765	5,00E-05	0,0004193	yes
KIF2C	KIF2C	KIF2C	chr1:4520548	shneg	shCHD1	OK	31,2146	43,8514	0,490402	2,71406	5,00E-05	0,0004193	yes
POLR2J4	POLR2J4	POLR2J4	chr7:4396603	shneg	shCHD1	OK	1,65314	2,32216	0,490257	1,63147	0,00405	0,0187979	yes
CDC42EP3	CDC42EP3	CDC42EP3	chr2:3787074	shneg	shCHD1	OK	2,83063	3,97459	0,489682	2,34972	5,00E-05	0,0004193	yes
CHDH	CHDH	CHDH	chr3:5385032	shneg	shCHD1	OK	3,56773	5,00898	0,489509	2,31722	5,00E-05	0,0004193	yes
FJX1	FJX1	FJX1	chr11:356397	shneg	shCHD1	OK	10,3686	14,5512	0,48892	2,45545	5,00E-05	0,0004193	yes
LRRC16A	LRRC16A	LRRC16A	chr6:2527965	shneg	shCHD1	OK	11,0131	15,4548	0,488836	2,64892	5,00E-05	0,0004193	yes
PCGF6	PCGF6	PCGF6	chr10:105062	shneg	shCHD1	OK	5,4348	7,62639	0,488773	2,28464	5,00E-05	0,0004193	yes
LIMD2	LIMD2	LIMD2	chr17:616998	shneg	shCHD1	OK	8,05311	11,2998	0,488674	1,88955	0,00105	0,00607369	yes
TUBA4A	TUBA4A	TUBA4A	chr2:2201101	shneg	shCHD1	OK	70,9424	99,5337	0,488536	2,44315	5,00E-05	0,0004193	yes
PINX1	PINX1	PINX1	chr8:1062288	shneg	shCHD1	OK	4,04075	5,66708	0,487981	1,56468	0,00775	0,03233	yes
HLA-B	HLA-B	HLA-B	chr6_ssto_ha	shneg	shCHD1	OK	187,579	263,06	0,487894	2,84747	5,00E-05	0,0004193	yes
CDC25C	CDC25C	CDC25C	chr5:1376209	shneg	shCHD1	OK	7,71529	10,8189	0,487766	2,37291	5,00E-05	0,0004193	yes
CD24	CD24	CD24	chrY:2109458	shneg	shCHD1	OK	1,66863	2,33961	0,487604	1,45094	0,01275	0,0486028	yes
SMC1A	SMC1A	SMC1A	chrX:5340106	shneg	shCHD1	OK	25,5743	35,8556	0,487503	2,83932	5,00E-05	0,0004193	yes
RFXAP	RFXAP	RFXAP	chr13:373933	shneg	shCHD1	OK	2,5093	3,51728	0,487172	1,9945	0,0006	0,00377139	yes
SH3PXD2B	SH3PXD2B	SH3PXD2B	chr5:1717605	shneg	shCHD1	OK	6,06791	8,50317	0,486802	2,58554	5,00E-05	0,0004193	yes
SLC5A6	SLC5A6	SLC5A6	chr2:2742245	shneg	shCHD1	OK	12,5557	17,5904	0,486444	1,62041	0,0058	0,0254211	yes
TEX30	TEX30	TEX30	chr13:103418	shneg	shCHD1	OK	21,9939	30,8071	0,486159	2,38213	0,0001	0,00078473	yes
CNGB1	CNGB1	CNGB1	chr16:579162	shneg	shCHD1	OK	2,72667	3,81902	0,486061	2,17548	0,0001	0,00078473	yes
IL6ST	IL6ST	IL6ST	chr5:5523092	shneg	shCHD1	OK	15,0286	21,0456	0,485802	2,7371	5,00E-05	0,0004193	yes
TTLL4	TTLL4	TTLL4	chr2:2195755	shneg	shCHD1	OK	11,4075	15,9693	0,485313	2,61272	5,00E-05	0,0004193	yes
C8orf55	C8orf55	C8orf55	chr8:1438086	shneg	shCHD1	OK	12,1567	17,017	0,485225	2,42565	5,00E-05	0,0004193	yes
ANKHD1	ANKHD1	ANKHD1	chr5:1397813	shneg	shCHD1	OK	17,2114	24,0885	0,484982	1,53847	0,0087	0,0355373	yes
RNF44	RNF44	RNF44	chr5:1759536	shneg	shCHD1	OK	20,4881	28,6731	0,484912	2,67285	5,00E-05	0,0004193	yes
TMEM57	TMEM57	TMEM57	chr1:2575738	shneg	shCHD1	OK	3,43255	4,80347	0,484794	2,3097	0,0001	0,00078473	yes
ARRB2	ARRB2	ARRB2	chr17:461378	shneg	shCHD1	OK	20,4974	28,6831	0,48476	2,52761	5,00E-05	0,0004193	yes
DPYSL5	DPYSL5	DPYSL5	chr2:2707096	shneg	shCHD1	OK	3,74035	5,23313	0,484499	2,30839	5,00E-05	0,0004193	yes
MPRIP	MPRIP	MPRIP	chr17:169460	shneg	shCHD1	OK	8,8206	12,3395	0,484339	2,69833	5,00E-05	0,0004193	yes
CYCS	CYCS	CYCS	chr7:2515826	shneg	shCHD1	OK	18,7523	26,2316	0,484242	2,70714	5,00E-05	0,0004193	yes
SLC38A5	SLC38A5	SLC38A5	chrX:4831692	shneg	shCHD1	OK	46,6221	65,2131	0,484147	2,67127	5,00E-05	0,0004193	yes
TCEA1	TCEA1	TCEA1	chr8:5487911	shneg	shCHD1	OK	42,1825	58,9856	0,483718	2,72235	5,00E-05	0,0004193	yes
CLDN16	CLDN16	CLDN16	chr3:1901056	shneg	shCHD1	OK	1,88723	2,63893	0,483684	1,88936	0,00095	0,00559279	yes
CHEK2	CHEK2	CHEK2	chr22:290837	shneg	shCHD1	OK	10,8533	15,1746	0,483527	2,31638	5,00E-05	0,0004193	yes
EEF1A2	EEF1A2	EEF1A2	chr20:621193	shneg	shCHD1	OK	119,802	167,483	0,483366	2,78684	5,00E-05	0,0004193	yes
BCL2L1	BCL2L1	BCL2L1	chr20:302522	shneg	shCHD1	OK	66,8651	93,4662	0,483192	2,75736	5,00E-05	0,0004193	yes
NUP210	NUP210	NUP210	chr3:1335773	shneg	shCHD1	OK	12,2295	17,094	0,483128	2,67438	5,00E-05	0,0004193	yes
TNIP1	TNIP1	TNIP1	chr5:1504095	shneg	shCHD1	OK	28,0068	39,1254	0,482327	2,62284	5,00E-05	0,0004193	yes
NKD2	NKD2	NKD2	chr5:1009167	shneg	shCHD1	OK	3,27194	4,57084	0,482315	1,82394	0,0024	0,0121606	yes
ZRSR2	ZRSR2	ZRSR2	chrX:1580857	shneg	shCHD1	OK	11,9035	16,6264	0,482095	2,34411	0,0001	0,00078473	yes
DHX8	DHX8	DHX8	chr17:415613	shneg	shCHD1	OK	16,8867	23,5856	0,482013	2,62939	5,00E-05	0,0004193	yes
BOP1	BOP1	BOP1	chr8:1454860	shneg	shCHD1	OK	30,7878	42,9957	0,481836	2,61484	5,00E-05	0,0004193	yes
AGPAT5	AGPAT5	AGPAT5	chr8:6565877	shneg	shCHD1	OK	5,27724	7,36878	0,481642	2,44806	5,00E-05	0,0004193	yes
PHLPP1	PHLPP1	PHLPP1	chr18:603826	shneg	shCHD1	OK	1,8397	2,56811	0,481231	2,26362	0,00015	0,00112331	yes
GINS2	GINS2	GINS2	chr16:857112	shneg	shCHD1	OK	17,5531	24,4892	0,480418	2,29199	5,00E-05	0,0004193	yes
ZNF395	ZNF395	ZNF395	chr8:2820310	shneg	shCHD1	OK	8,07114	11,2569	0,479965	2,50597	5,00E-05	0,0004193	yes
TUBB2B	TUBB2B	TUBB2B	chr6:3224494	shneg	shCHD1	OK	30,5405	42,5941	0,47993	2,57616	5,00E-05	0,0004193	yes
EXOSC8	EXOSC8	EXOSC8	chr13:375746	shneg	shCHD1	OK	18,2853	25,5004	0,479834	1,45214	0,0124	0,0475026	yes
DNAJC6	DNAJC6	DNAJC6	chr1:6573042	shneg	shCHD1	OK	1,68066	2,34356	0,479675	2,20745	0,0002	0,00144867	yes
IL18	IL18	IL18	chr11:112013	shneg	shCHD1	OK	4,81113	6,70433	0,478717	1,71445	0,00335	0,0160296	yes
LRRC8D	LRRC8D	LRRC8D	chr1:9028657	shneg	shCHD1	OK	5,4933	7,65466	0,478666	2,31819	5,00E-05	0,0004193	yes
C1orf112	C1orf112	C1orf112	chr1:1697645	shneg	shCHD1	OK	3,3032	4,60166	0,478292	1,56965	0,0059	0,0257621	yes
GFPT1	GFPT1	GFPT1	chr2:6954690	shneg	shCHD1	OK	8,88498	12,3769	0,478204	2,63073	5,00E-05	0,0004193	yes
ANKRD33B	ANKRD33B	ANKRD33B	chr5:1056443	shneg	shCHD1	OK	1,93526	2,69562	0,478092	2,29209	5,00E-05	0,0004193	yes
FTSJ2	FTSJ2	FTSJ2	chr7:2273925	shneg	shCHD1	OK	19,0684	26,5539	0,477736	2,41423	5,00E-05	0,0004193	yes
RGS1	RGS1	RGS1	chr1:1925448	shneg	shCHD1	OK	83,9962	116,962	0,477639	2,66883	5,00E-05	0,0004193	yes
EFCAB11	EFCAB11	EFCAB11	chr14:902634	shneg	shCHD1	OK	8,0765	11,2418	0,477067	1,62925	0,00435	0,019951	yes
SERPINE2	SERPINE2	SERPINE2	chr2:2248397	shneg	shCHD1	OK	126,215	175,617	0,476546	2,77972	5,00E-05	0,0004193	yes
MAK16	MAK16	MAK16	chr8:3334268	shneg	shCHD1	OK	5,51107	7,6659	0,476123	1,70818	0,00275	0,0136364	yes
PKIG	PKIG	PKIG	chr20:431604	shneg	shCHD1	OK	12,9276	17,9811	0,476027	2,25127	0,0001	0,00078473	yes
PPM1H	PPM1H	PPM1H	chr12:630377	shneg	shCHD1	OK	25,241	35,1015	0,475768	2,71743	5,00E-05	0,0004193	yes
NUSAP1	NUSAP1	NUSAP1	chr15:416248	shneg	shCHD1	OK	56,3586	78,33	0,47493	2,67933	5,00E-05	0,0004193	yes
LYRM4	LYRM4	LYRM4	chr6:5108652	shneg	shCHD1	OK	8,98331	12,4843	0,474794	1,87183	0,001	0,0058183	yes
Sep 03	Sep 03	Sep 03	chr22:423729	shneg	shCHD1	OK	4,15407	5,77075	0,474235	2,02497	0,00035	0,00236888	yes
SMARCC1	SMARCC1	SMARCC1	chr3:4762737	shneg	shCHD1	OK	31,5643	43,8347	0,47378	2,72197	5,00E-05	0,0004193	yes
CDC45	CDC45	CDC45	chr22:194674	shneg	shCHD1	OK	7,28635	10,1174	0,473567	2,24452	0,0001	0,00078473	yes
KLHDC7A	KLHDC7A	KLHDC7A	chr1:1880742	shneg	shCHD1	OK	7,14836	9,91882	0,472557	2,4492	5,00E-05	0,0004193	yes
SLC25A45	SLC25A45	SLC25A45	chr11:651426	shneg	shCHD1	OK	3,34141	4,63533	0,472214	1,9666	0,0007	0,00428773	yes
SLC30A3	SLC30A3	SLC30A3	chr2:2747743	shneg	shCHD1	OK	3,05765	4,23933	0,471415	1,84395	0,00105	0,00607369	yes
MPDU1	MPDU1	MPDU1	chr17:748696	shneg	shCHD1	OK	17,5057	24,2685	0,471262	2,33034	0,0001	0,00078473	yes
TRAF3	TRAF3	TRAF3	chr14:103243	shneg	shCHD1	OK	8,71604	12,0832	0,471256	2,57174	0,0001	0,00078473	yes
G3BP1	G3BP1	G3BP1	chr5:1511514	shneg	shCHD1	OK	57,4141	79,5939	0,471252	2,68552	5,00E-05	0,0004193	yes
HAUS8	HAUS8	HAUS8	chr19:171605	shneg	shCHD1	OK	9,8009	13,5856	0,47109	2,22477	5,00E-05	0,0004193	yes
DBN1	DBN1	DBN1	chr5:1768836	shneg	shCHD1	OK	66,3431	91,9478	0,470869	2,71683	5,00E-05	0,0004193	yes
CFDP1	CFDP1	CFDP1	chr16:753276	shneg	shCHD1	OK	58,9368	81,6595	0,470452	2,55405	5,00E-05	0,0004193	yes
YWHAE	YWHAE	YWHAE	chr17:124783	shneg	shCHD1	OK	238,105	329,871	0,470303	2,79891	5,00E-05	0,0004193	yes

KPNB1	KPNB1	KPNB1	chr17:457272	shneg	shCHD1	OK	92,2898	127,857	0,470283	2,77985	5,00E-05	0,0004193	yes
TAF4B	TAF4B	TAF4B	chr18:238064	shneg	shCHD1	OK	1,55669	2,1564	0,470141	1,97891	0,0005	0,00321686	yes
CORO7	CORO7	CORO7	chr16:439025	shneg	shCHD1	OK	5,98629	8,29246	0,470138	1,96384	0,0007	0,00428773	yes
RAB11FIP5	RAB11FIP5	RAB11FIP5	chr2:7330050	shneg	shCHD1	OK	17,3201	23,9909	0,470042	2,57374	5,00E-05	0,0004193	yes
PNP	PNP	PNP	chr14:209375	shneg	shCHD1	OK	29,649	41,0543	0,469546	2,55482	5,00E-05	0,0004193	yes
NOL7	NOL7	NOL7	chr6:1361555	shneg	shCHD1	OK	30,4867	42,2062	0,469277	2,29638	0,0001	0,00078473	yes
SOX4	SOX4	SOX4	chr6:2159397	shneg	shCHD1	OK	32,2608	44,6616	0,469255	2,67757	5,00E-05	0,0004193	yes
TMEM14C	TMEM14C	TMEM14C	chr6:1072314	shneg	shCHD1	OK	44,6525	61,7992	0,468849	2,47679	5,00E-05	0,0004193	yes
PSMG4	PSMG4	PSMG4	chr6:3259161	shneg	shCHD1	OK	16,9634	23,4754	0,468724	1,45728	0,01075	0,0422389	yes
MCTS1	MCTS1	MCTS1	chrX:1197377	shneg	shCHD1	OK	3,77835	5,22775	0,468434	2,44313	5,00E-05	0,0004193	yes
FKBP10	FKBP10	FKBP10	chr17:399689	shneg	shCHD1	OK	144,844	200,356	0,468071	2,7704	5,00E-05	0,0004193	yes
FKBP5	FKBP5	FKBP5	chr6:3554136	shneg	shCHD1	OK	21,665	29,9574	0,46755	2,5508	5,00E-05	0,0004193	yes
CHTF18	CHTF18	CHTF18	chr16:838621	shneg	shCHD1	OK	12,0932	16,7218	0,467543	2,38881	5,00E-05	0,0004193	yes
CDC42EP2	CDC42EP2	CDC42EP2	chr11:650822	shneg	shCHD1	OK	4,09288	5,65892	0,467411	1,99874	0,0006	0,00377139	yes
AZIN1	AZIN1	AZIN1	chr8:1038385	shneg	shCHD1	OK	25,7115	35,5491	0,4674	2,61085	5,00E-05	0,0004193	yes
OTUD6B	OTUD6B	OTUD6B	chr8:9208242	shneg	shCHD1	OK	3,07601	4,25188	0,467042	2,1575	0,00025	0,00176854	yes
AIFM2	AIFM2	AIFM2	chr10:718123	shneg	shCHD1	OK	6,42865	8,88548	0,466934	1,7096	0,0029	0,0142283	yes
DENND5A	DENND5A	DENND5A	chr11:916037	shneg	shCHD1	OK	37,4887	51,7929	0,466298	2,70072	5,00E-05	0,0004193	yes
NIPAL1	NIPAL1	NIPAL1	chr4:4801879	shneg	shCHD1	OK	4,82036	6,65899	0,466162	2,15636	0,00015	0,00112331	yes
ULK4	ULK4	ULK4	chr3:4128808	shneg	shCHD1	OK	0,929472	1,28395	0,466108	1,57291	0,0059	0,0257621	yes
KIF15	KIF15	KIF15	chr3:4480320	shneg	shCHD1	OK	5,95199	8,22188	0,466097	2,35446	0,00015	0,00112331	yes
NUP62CL	NUP62CL	NUP62CL	chrX:1063666	shneg	shCHD1	OK	6,917	9,53976	0,463807	2,10966	0,00035	0,00236888	yes
SH2D4A	SH2D4A	SH2D4A	chr8:1917108	shneg	shCHD1	OK	8,7271	12,036	0,463785	2,34526	5,00E-05	0,0004193	yes
TANC1	TANC1	TANC1	chr2:1598251	shneg	shCHD1	OK	2,78218	3,83658	0,463602	2,27584	0,0001	0,00078473	yes
SMYD2	SMYD2	SMYD2	chr1:2144545	shneg	shCHD1	OK	13,7062	18,8912	0,462881	2,26021	0,0001	0,00078473	yes
SLC35B3	SLC35B3	SLC35B3	chr6:8413300	shneg	shCHD1	OK	4,7731	6,57769	0,462654	2,04177	0,0004	0,00266202	yes
ATAD2	ATAD2	ATAD2	chr8:1243320	shneg	shCHD1	OK	12,2352	16,8606	0,46261	2,51222	5,00E-05	0,0004193	yes
UBE2C	UBE2C	UBE2C	chr20:444412	shneg	shCHD1	OK	126,519	174,322	0,462401	2,45323	5,00E-05	0,0004193	yes
CBX5	CBX5	CBX5	chr12:546247	shneg	shCHD1	OK	31,2095	42,9757	0,461537	2,71666	5,00E-05	0,0004193	yes
SPATS2	SPATS2	SPATS2	chr12:497606	shneg	shCHD1	OK	18,7815	25,8588	0,461348	2,48621	5,00E-05	0,0004193	yes
UBE2D2	UBE2D2	UBE2D2	chr5:1389407	shneg	shCHD1	OK	26,3526	36,2727	0,460937	2,50701	5,00E-05	0,0004193	yes
NEDD4L	NEDD4L	NEDD4L	chr18:557116	shneg	shCHD1	OK	1,83025	2,51856	0,460558	2,19697	0,00015	0,00112331	yes
HMMR	HMMR	HMMR	chr5:1628875	shneg	shCHD1	OK	9,41353	12,9536	0,460547	2,32202	0,0001	0,00078473	yes
GPC2	GPC2	GPC2	chr7:9976722	shneg	shCHD1	OK	2,16975	2,98461	0,460018	1,71214	0,00345	0,0164347	yes
FAM64A	FAM64A	FAM64A	chr17:634773	shneg	shCHD1	OK	20,4689	28,1546	0,459939	2,32375	0,0001	0,00078473	yes
FBRSL1	FBRSL1	FBRSL1	chr12:133067	shneg	shCHD1	OK	9,88551	13,5936	0,459541	2,40828	5,00E-05	0,0004193	yes
RLTPR	RLTPR	RLTPR	chr16:676790	shneg	shCHD1	OK	7,94717	10,928	0,459515	1,80577	0,00185	0,00975716	yes
RPL22L1	RPL22L1	RPL22L1	chr3:1705826	shneg	shCHD1	OK	6,748	9,27352	0,458657	2,17218	5,00E-05	0,0004193	yes
MCPH1	MCPH1	MCPH1	chr8:6264112	shneg	shCHD1	OK	2,7083	3,72171	0,458578	1,71352	0,00365	0,0172576	yes
TMEM70	TMEM70	TMEM70	chr8:7488837	shneg	shCHD1	OK	9,14355	12,5555	0,457494	2,17916	0,00015	0,00112331	yes
HIRIP3	HIRIP3	HIRIP3	chr16:300036	shneg	shCHD1	OK	4,45346	6,11424	0,457247	2,1478	0,00025	0,00176854	yes
BLVRA	BLVRA	BLVRA	chr7:4379827	shneg	shCHD1	OK	27,601	37,8897	0,457087	2,28181	5,00E-05	0,0004193	yes
CKS1B	CKS1B	CKS1B	chr1:1549471	shneg	shCHD1	OK	130,064	178,516	0,456833	2,53173	5,00E-05	0,0004193	yes
NOP16	NOP16	NOP16	chr5:1758109	shneg	shCHD1	OK	26,1557	35,8722	0,455739	2,22794	0,00015	0,00112331	yes
ORC6	ORC6	ORC6	chr16:467235	shneg	shCHD1	OK	15,0414	20,6289	0,45573	2,24408	0,00015	0,00112331	yes
PFN1	PFN1	PFN1	chr17:484894	shneg	shCHD1	OK	818,718	1122,37	0,455114	2,70288	5,00E-05	0,0004193	yes
EZH1	EZH1	EZH1	chr17:408522	shneg	shCHD1	OK	14,2959	19,5943	0,454832	2,46845	5,00E-05	0,0004193	yes
TRIP13	TRIP13	TRIP13	chr5:892968-!	shneg	shCHD1	OK	23,1333	31,7033	0,454664	2,37213	5,00E-05	0,0004193	yes
HSPA9	HSPA9	HSPA9	chr5:1378905	shneg	shCHD1	OK	122,929	168,454	0,45453	2,69365	5,00E-05	0,0004193	yes
C2orf18	C2orf18	C2orf18	chr2:2698714	shneg	shCHD1	OK	11,9526	16,376	0,454255	2,40252	5,00E-05	0,0004193	yes
CDC7	CDC7	CDC7	chr1:9196640	shneg	shCHD1	OK	4,87654	6,67941	0,453861	2,16457	0,0002	0,00144867	yes
EFHD2	EFHD2	EFHD2	chr1:1573639	shneg	shCHD1	OK	14,6123	20,0104	0,45357	2,33375	5,00E-05	0,0004193	yes
LINC00319	LINC00319	LINC00319	chr21:448699	shneg	shCHD1	OK	11,2567	15,4143	0,453483	2,31341	5,00E-05	0,0004193	yes
LSM5	LSM5	LSM5	chr7:3252494	shneg	shCHD1	OK	15,7638	21,5855	0,453454	2,31108	5,00E-05	0,0004193	yes
GINS4	GINS4	GINS4	chr8:4138672	shneg	shCHD1	OK	7,5551	10,3451	0,453419	2,29815	5,00E-05	0,0004193	yes
DBF4B	DBF4B	DBF4B	chr17:427859	shneg	shCHD1	OK	7,58017	10,3784	0,453279	2,1551	0,00035	0,00236888	yes
CACNG8	CACNG8	CACNG8	chr19:544662	shneg	shCHD1	OK	3,68394	5,04352	0,453181	2,32238	0,0001	0,00078473	yes
HAR1A	HAR1A	HAR1A	chr20:617268	shneg	shCHD1	OK	1,61307	2,2079	0,452869	1,52779	0,00805	0,0332923	yes
FAM83H	FAM83H	FAM83H	chr8:1448061	shneg	shCHD1	OK	4,4363	6,07188	0,452787	2,24791	0,0001	0,00078473	yes
KCNC3	KCNC3	KCNC3	chr19:508187	shneg	shCHD1	OK	2,65235	3,62999	0,452691	1,96314	0,0009	0,00533451	yes
FKBPL	FKBPL	FKBPL	chr6_mcf_ha|	shneg	shCHD1	OK	4,28794	5,86677	0,452282	1,65981	0,0051	0,0228051	yes
PELP1	PELP1	PELP1	chr17:457467	shneg	shCHD1	OK	16,5245	22,6073	0,452185	2,4387	5,00E-05	0,0004193	yes
DHRS7B	DHRS7B	DHRS7B	chr17:210302	shneg	shCHD1	OK	5,73793	7,84907	0,451992	2,07786	0,00045	0,00293585	yes
KCTD15	KCTD15	KCTD15	chr19:342877	shneg	shCHD1	OK	5,42188	7,4167	0,451985	2,2052	5,00E-05	0,0004193	yes
IL13RA2	IL13RA2	IL13RA2	chrX:1142385	shneg	shCHD1	OK	426,223	582,839	0,451488	2,68709	5,00E-05	0,0004193	yes
DCP2	DCP2	DCP2	chr5:1123124	shneg	shCHD1	OK	4,17557	5,70965	0,451428	2,13553	0,0002	0,00144867	yes
HELLS	HELLS	HELLS	chr10:963055	shneg	shCHD1	OK	8,55421	11,6957	0,451276	2,25556	0,0001	0,00078473	yes
LOC10050671	LOC10050671	LOC10050671	chr22:455296	shneg	shCHD1	OK	3,93304	5,3773	0,451239	1,6017	0,00605	0,0262198	yes
C15orf42	C15orf42	C15orf42	chr15:901188	shneg	shCHD1	OK	4,37101	5,97545	0,451078	1,67286	0,0035	0,0166331	yes
TCF19	TCF19	TCF19	chr6_ssto_ha	shneg	shCHD1	OK	15,7747	21,5645	0,451047	2,38689	5,00E-05	0,0004193	yes
CCNE1	CCNE1	CCNE1	chr19:303029	shneg	shCHD1	OK	8,66426	11,8438	0,450982	2,17526	0,0002	0,00144867	yes
GNL3	GNL3	GNL3	chr3:5271993	shneg	shCHD1	OK	35,2941	48,2444	0,450931	1,57824	0,00615	0,0265788	yes
DTYMK	DTYMK	DTYMK	chr2:2426151	shneg	shCHD1	OK	34,6154	47,3072	0,450649	2,33831	5,00E-05	0,0004193	yes
CNOT7	CNOT7	CNOT7	chr8:1708673	shneg	shCHD1	OK	17,2398	23,5581	0,450483	2,31366	0,0002	0,00144867	yes
EIF3B	EIF3B	EIF3B	chr7:2394473	shneg	shCHD1	OK	85,1195	116,293	0,450202	2,61017	5,00E-05	0,0004193	yes
C3orf14	C3orf14	C3orf14	chr3:6230539	shneg	shCHD1	OK	15,3572	20,9768	0,449882	2,01619	0,0005	0,00321686	yes
NAPB	NAPB	NAPB	chr20:233551	shneg	shCHD1	OK	3,73523	5,10094	0,449565	2,16736	0,00035	0,00236888	yes
DPH2	DPH2	DPH2	chr1:4443565	shneg	shCHD1	OK	13,2232	18,0553	0,44935	2,26633	0,00015	0,00112331	yes
CSE1L	CSE1L	CSE1L	chr20:476627	shneg	shCHD1	OK	37,1601	50,7263	0,448981	2,5539	5,00E-05	0,0004193	yes
NBR1	NBR1	NBR1	chr17:413224	shneg	shCHD1	OK	38,6152	52,7117	0,448954	2,58776	5,00E-05	0,0004193	yes
EIF4EBP2	EIF4EBP2	EIF4EBP2	chr10:721638	shneg	shCHD1	OK	14,637	19,9665	0,447965	2,51357	5,00E-05	0,0004193	yes
DIAPH1	DIAPH1	DIAPH1	chr5:1408945	shneg	shCHD1	OK	28,2643	38,5503	0,44776	2,55987	5,00E-05	0,0004193	yes
GRPEL2	GRPEL2	GRPEL2	chr5:1487249	shneg	shCHD1	OK	5,21935	7,11859	0,44772	2,18295	0,0002	0,00144867	yes
FOXO1	FOXO1	FOXO1	chr13:411298	shneg	shCHD1	OK	1,37214	1,87135	0,447654	1,91758	0,0009	0,00533451	yes
FHOD1	FHOD1	FHOD1	chr16:672632	shneg	shCHD1	OK	17,1974	23,453	0,447585	2,42778	5,00E-05	0,0004193	yes
MICB	MICB	MICB	chr6_ssto_ha	shneg	shCHD1	OK	7,11405	9,70153	0,44754	2,11444	0,00025	0,00176854	yes
HNRNPA0	HNRNPA0	HNRNPA0	chr5:1370870	shneg	shCHD1	OK	72,2026	98,4435	0,447245	2,5815	5,00E-05	0,0004193	yes
FAM35A	FAM35A	FAM35A	chr10:888549	shneg	shCHD1	OK	4,0661	5,54351	0,447151	2,13282	0,0003	0,0020738	yes
GARS	GARS	GARS	chr7:3063418	shneg	shCHD1	OK	50,6735	69,076	0,446955	2,52857	5,00E-05	0,0004193	yes
POLA2	POLA2	POLA2	chr11:650294	shneg	shCHD1	OK	11,2285	15,301	0,446459	2,24297	5,00E-05	0,0004193	yes
CXorf56	CXorf56	CXorf56	chrX:1186721	shneg	shCHD1	OK	8,69163	11,8416	0,446166	2,16002	0,0001	0,00078473	yes
CAB39L	CAB39L	CAB39L	chr13:498827	shneg	shCHD1	OK	2,75765	3,75661	0,445994	2,03399	0,00045	0,00293585	yes
IDH2	IDH2	IDH2	chr15:906272	shneg	shCHD1	OK	21,0858	28,7129	0,445432	2,29379	5,00E-05	0,0004193	yes

SKP1	SKP1	SKP1	chr5:1334920 shneg	shCHD1	OK	91,3963	124,452	0,445387	2,54415	0,0001	0,00078473	yes
KIAA1797	KIAA1797	KIAA1797	chr9:2065830 shneg	shCHD1	OK	7,79357	10,6122	0,445367	2,36281	0,0001	0,00078473	yes
KLHL11	KLHL11	KLHL11	chr17:400097 shneg	shCHD1	OK	2,80334	3,81719	0,445367	1,75512	0,00215	0,0110834	yes
ENTPD4	ENTPD4	ENTPD4	chr8:2328666 shneg	shCHD1	OK	8,90651	12,1219	0,444678	2,38126	5,00E-05	0,0004193	yes
RNF19B	RNF19B	RNF19B	chr1:3340204 shneg	shCHD1	OK	6,92164	9,41775	0,444269	2,09483	0,00015	0,00112331	yes
LARGE	LARGE	LARGE	chr22:336690 shneg	shCHD1	OK	2,98831	4,06524	0,444012	2,12031	0,0002	0,00144867	yes
LRRN4	LRRN4	LRRN4	chr20:602142 shneg	shCHD1	OK	3,00841	4,09106	0,443474	1,89354	0,0012	0,00680293	yes
TMEM109	TMEM109	TMEM109	chr11:606813 shneg	shCHD1	OK	33,244	45,2045	0,443372	2,42771	5,00E-05	0,0004193	yes
CCDC14	CCDC14	CCDC14	chr3:1236322 shneg	shCHD1	OK	5,66953	7,70758	0,44305	2,18749	0,00015	0,00112331	yes
ZWINT	ZWINT	ZWINT	chr10:581171 shneg	shCHD1	OK	39,6051	53,8312	0,442755	2,35776	5,00E-05	0,0004193	yes
PRR5	PRR5	PRR5	chr22:450644 shneg	shCHD1	OK	5,64851	7,67521	0,442335	1,65755	0,0043	0,0197347	yes
CPLX1	CPLX1	CPLX1	chr4:778744-8 shneg	shCHD1	OK	12,4178	16,8683	0,44191	2,19341	0,0001	0,00078473	yes
LRR1	LRR1	LRR1	chr14:500654 shneg	shCHD1	OK	9,46997	12,8633	0,441832	1,6853	0,005	0,0224661	yes
TYMP	TYMP	TYMP	chr22:509466 shneg	shCHD1	OK	36,7665	49,9281	0,441463	1,72395	0,00285	0,0140225	yes
PRELID2	PRELID2	PRELID2	chr5:1451385 shneg	shCHD1	OK	2,33327	3,16801	0,441221	1,58893	0,00595	0,0259073	yes
TAP1	TAP1	TAP1	chr6_ssto_ha shneg	shCHD1	OK	24,4431	33,1831	0,441018	1,8485	0,00135	0,00748275	yes
UPF3B	UPF3B	UPF3B	chrX:1189679 shneg	shCHD1	OK	15,7897	21,4239	0,44024	2,27847	0,0001	0,00078473	yes
ANO7	ANO7	ANO7	chr2:2421279 shneg	shCHD1	OK	1,32967	1,80408	0,440194	1,51793	0,0058	0,0254211	yes
NHSL1	NHSL1	NHSL1	chr6:1387431 shneg	shCHD1	OK	15,7455	21,3629	0,440164	2,47049	5,00E-05	0,0004193	yes
H2AFX	H2AFX	H2AFX	chr11:118964 shneg	shCHD1	OK	61,575	83,5362	0,440056	2,42963	5,00E-05	0,0004193	yes
GSR	GSR	GSR	chr8:3053557 shneg	shCHD1	OK	14,6719	19,8989	0,439633	2,31745	5,00E-05	0,0004193	yes
C5orf25	C5orf25	C5orf25	chr5:1756653 shneg	shCHD1	OK	6,45631	8,75405	0,439242	2,0544	0,0003	0,0020738	yes
CENPM	CENPM	CENPM	chr22:423347 shneg	shCHD1	OK	16,8979	22,9035	0,438722	2,05063	0,00035	0,00236888	yes
EIF4EBP1	EIF4EBP1	EIF4EBP1	chr8:3788801 shneg	shCHD1	OK	58,4701	79,2504	0,438719	2,29046	0,00025	0,00176854	yes
RFC3	RFC3	RFC3	chr13:343922 shneg	shCHD1	OK	14,6492	19,8554	0,438709	2,20761	5,00E-05	0,0004193	yes
RNF168	RNF168	RNF168	chr3:1961956 shneg	shCHD1	OK	3,99717	5,41615	0,438288	2,142	0,00015	0,00112331	yes
RPA1	RPA1	RPA1	chr17:173327 shneg	shCHD1	OK	25,7531	34,8916	0,438133	2,45685	5,00E-05	0,0004193	yes
PTPRN2	PTPRN2	PTPRN2	chr7:1573317 shneg	shCHD1	OK	9,27275	12,5632	0,438129	2,2847	5,00E-05	0,0004193	yes
AP3M2	AP3M2	AP3M2	chr8:4201046 shneg	shCHD1	OK	9,76139	13,2225	0,437842	2,2603	0,00015	0,00112331	yes
MND1	MND1	MND1	chr4:1542658 shneg	shCHD1	OK	8,09427	10,9587	0,437104	1,74032	0,00225	0,0115391	yes
MTHFD2	MTHFD2	MTHFD2	chr2:7442568 shneg	shCHD1	OK	28,955	39,1979	0,436962	2,36144	0,0001	0,00078473	yes
POLE	POLE	POLE	chr12:133200 shneg	shCHD1	OK	18,6755	25,2787	0,43677	2,48041	5,00E-05	0,0004193	yes
FZD5	FZD5	FZD5	chr2:2086273 shneg	shCHD1	OK	4,67832	6,32926	0,436047	2,22428	5,00E-05	0,0004193	yes
LOC642846	LOC642846	LOC642846	chr12:943625 shneg	shCHD1	OK	1,66313	2,2497	0,435836	1,66586	0,00455	0,0207047	yes
PDGFB	PDGFB	PDGFB	chr22:396196 shneg	shCHD1	OK	8,59535	11,6255	0,435667	2,06633	0,00025	0,00176854	yes
STIP1	STIP1	STIP1	chr11:639535 shneg	shCHD1	OK	104,319	141,07	0,435403	2,51635	5,00E-05	0,0004193	yes
C17orf85	C17orf85	C17orf85	chr17:371004 shneg	shCHD1	OK	8,22267	11,1144	0,434747	2,35979	5,00E-05	0,0004193	yes
PTTG1	PTTG1	PTTG1	chr5:1598488 shneg	shCHD1	OK	108,129	146,114	0,434339	2,33487	5,00E-05	0,0004193	yes
PDGFRL	PDGFRL	PDGFRL	chr8:1743394 shneg	shCHD1	OK	3,08959	4,17492	0,434332	1,63646	0,0059	0,0257621	yes
MAP2K1	MAP2K1	MAP2K1	chr15:666792 shneg	shCHD1	OK	17,1595	23,1839	0,434111	2,074	0,0002	0,00144867	yes
GRK6	GRK6	GRK6	chr5:1768536 shneg	shCHD1	OK	22,6682	30,6245	0,43402	1,77398	0,00275	0,0136364	yes
SH2D5	SH2D5	SH2D5	chr1:2104622 shneg	shCHD1	OK	1,1863	1,60266	0,434001	1,50766	0,01	0,0398199	yes
ALG13	ALG13	ALG13	chrX:1109243 shneg	shCHD1	OK	9,92396	13,4054	0,433832	2,20875	0,0001	0,00078473	yes
DHX33	DHX33	DHX33	chr17:534423 shneg	shCHD1	OK	7,34842	9,92612	0,433796	2,27476	0,0001	0,00078473	yes
SHCBP1	SHCBP1	SHCBP1	chr16:466144 shneg	shCHD1	OK	10,8277	14,6255	0,433763	2,23423	5,00E-05	0,0004193	yes
ANKRD10	ANKRD10	ANKRD10	chr13:111530 shneg	shCHD1	OK	25,7026	34,7064	0,433288	2,33901	0,0001	0,00078473	yes
WEE1	WEE1	WEE1	chr11:959522 shneg	shCHD1	OK	20,841	28,1388	0,433137	2,36523	5,00E-05	0,0004193	yes
NDC80	NDC80	NDC80	chr18:257150 shneg	shCHD1	OK	11,4153	15,4093	0,432827	2,13611	0,00025	0,00176854	yes
FAM89A	FAM89A	FAM89A	chr1:2311547 shneg	shCHD1	OK	5,43764	7,33934	0,43267	1,80219	0,00195	0,0102111	yes
CCNF	CCNF	CCNF	chr16:247939 shneg	shCHD1	OK	10,4749	14,1348	0,432317	2,28131	0,0001	0,00078473	yes
MCM7	MCM7	MCM7	chr7:9969040 shneg	shCHD1	OK	134,381	181,329	0,432282	2,14421	0,00025	0,00176854	yes
MRPS18B	MRPS18B	MRPS18B	chr6_ssto_ha shneg	shCHD1	OK	38,3193	51,6988	0,432058	2,31006	5,00E-05	0,0004193	yes
MAPK8IP2	MAPK8IP2	MAPK8IP2	chr22:510391 shneg	shCHD1	OK	23,3657	31,5195	0,431852	2,358	0,0001	0,00078473	yes
NTNG2	NTNG2	NTNG2	chr9:1350373 shneg	shCHD1	OK	21,1639	28,546	0,431684	2,33303	0,00015	0,00112331	yes
ICAM1	ICAM1	ICAM1	chr19:103815 shneg	shCHD1	OK	24,1745	32,5918	0,431019	2,36225	5,00E-05	0,0004193	yes
LOC10028863	LOC10028863	LOC10028863	chr15:309383 shneg	shCHD1	OK	3,7432	5,04611	0,430902	2,08828	0,00045	0,00293585	yes
MCM3	MCM3	MCM3	chr6:5212881 shneg	shCHD1	OK	61,3043	82,6404	0,430859	2,47703	5,00E-05	0,0004193	yes
ANKRD46	ANKRD46	ANKRD46	chr8:1015329 shneg	shCHD1	OK	2,9069	3,91853	0,430834	1,83924	0,0014	0,00768951	yes
PRPF4B	PRPF4B	PRPF4B	chr6:4021568 shneg	shCHD1	OK	9,04361	12,1866	0,430325	2,33869	5,00E-05	0,0004193	yes
ZRANB3	ZRANB3	ZRANB3	chr2:1359575 shneg	shCHD1	OK	1,72736	2,32739	0,430146	1,70137	0,0038	0,0178336	yes
CENPF	CENPF	CENPF	chr1:2147765 shneg	shCHD1	OK	32,2299	43,4215	0,43001	2,50242	5,00E-05	0,0004193	yes
ECI2	ECI2	ECI2	chr6:4068592 shneg	shCHD1	OK	13,2367	17,8318	0,429905	2,03559	0,0003	0,0020738	yes
GCNT1	GCNT1	GCNT1	chr9:7905658 shneg	shCHD1	OK	0,956198	1,28805	0,429808	1,58044	0,0063	0,0271428	yes
WRNIP1	WRNIP1	WRNIP1	chr6:2765665 shneg	shCHD1	OK	17,4275	23,4753	0,429776	2,26648	5,00E-05	0,0004193	yes
PRNP	PRNP	PRNP	chr20:466679 shneg	shCHD1	OK	20,2863	27,3233	0,429628	2,26978	0,00015	0,00112331	yes
PPDPF	PPDPF	PPDPF	chr20:621521 shneg	shCHD1	OK	141,672	190,703	0,428774	2,35836	5,00E-05	0,0004193	yes
FOXM1	FOXM1	FOXM1	chr12:294598 shneg	shCHD1	OK	36,8962	49,6573	0,428535	2,39243	5,00E-05	0,0004193	yes
PPP2R3C	PPP2R3C	PPP2R3C	chr14:355546 shneg	shCHD1	OK	5,1026	6,8667	0,428386	1,88753	0,00115	0,00656221	yes
FAM188B	FAM188B	FAM188B	chr7:3079175 shneg	shCHD1	OK	4,34741	5,85027	0,428349	1,92049	0,0006	0,00377139	yes
ARHGEF10	ARHGEF10	ARHGEF10	chr8:1772148 shneg	shCHD1	OK	11,5966	15,6031	0,428137	2,31909	0,0001	0,00078473	yes
HDAC3	HDAC3	HDAC3	chr5:1410004 shneg	shCHD1	OK	47,3449	63,6934	0,427934	2,36372	5,00E-05	0,0004193	yes
PSMD13	PSMD13	PSMD13	chr11:236807 shneg	shCHD1	OK	46,7408	62,8746	0,427796	2,34586	5,00E-05	0,0004193	yes
TRIM16	TRIM16	TRIM16	chr17:155312 shneg	shCHD1	OK	2,14264	2,88195	0,427655	1,66679	0,0047	0,0212899	yes
PEMT	PEMT	PEMT	chr17:174088 shneg	shCHD1	OK	15,0798	20,2804	0,427472	2,02329	0,0003	0,0020738	yes
PDE8A	PDE8A	PDE8A	chr15:855237 shneg	shCHD1	OK	19,8753	26,7288	0,427414	2,31805	5,00E-05	0,0004193	yes
RPA3	RPA3	RPA3	chr7:7676574 shneg	shCHD1	OK	15,423	20,7361	0,427067	1,69094	0,00285	0,0140225	yes
SLC38A1	SLC38A1	SLC38A1	chr12:465768 shneg	shCHD1	OK	47,6585	64,0456	0,426366	2,53707	5,00E-05	0,0004193	yes
C1orf106	C1orf106	C1orf106	chr1:2008606 shneg	shCHD1	OK	1,64952	2,21633	0,426123	1,72769	0,0033	0,0158557	yes
LBR	LBR	LBR	chr1:2255892 shneg	shCHD1	OK	14,5636	19,5659	0,425977	2,28547	5,00E-05	0,0004193	yes
SSR1	SSR1	SSR1	chr6:7281287 shneg	shCHD1	OK	7,09127	9,52627	0,425867	2,3191	5,00E-05	0,0004193	yes
POU5F1	POU5F1	POU5F1	chr6_ssto_ha shneg	shCHD1	OK	4,01053	5,38609	0,425445	1,61662	0,0052	0,0231261	yes
HDHD1	HDHD1	HDHD1	chrX:6966960 shneg	shCHD1	OK	7,17442	9,63498	0,425419	1,86436	0,00135	0,00748275	yes
FAM57A	FAM57A	FAM57A	chr17:635846 shneg	shCHD1	OK	11,3523	15,2387	0,424756	2,08444	0,00025	0,00176854	yes
MNS1	MNS1	MNS1	chr15:566576 shneg	shCHD1	OK	3,61736	4,85535	0,424637	1,49529	0,0108	0,0423994	yes
ST8SIA4	ST8SIA4	ST8SIA4	chr5:1001426 shneg	shCHD1	OK	1,42731	1,91513	0,424143	1,85655	0,0013	0,00726051	yes
ITGA1	ITGA1	ITGA1	chr5:5208377 shneg	shCHD1	OK	185,564	248,898	0,42364	2,35009	5,00E-05	0,0004193	yes
PVRL3	PVRL3	PVRL3	chr3:1107906 shneg	shCHD1	OK	13,9827	18,7481	0,423097	2,0384	0,0002	0,00144867	yes
BPHL	BPHL	BPHL	chr6:3118925 shneg	shCHD1	OK	8,37754	11,229	0,422628	1,94174	0,00055	0,00350455	yes
BUB1B	BUB1B	BUB1B	chr15:404532 shneg	shCHD1	OK	18,4131	24,6802	0,422624	1,5396	0,0063	0,0271428	yes
GCSH	GCSH	GCSH	chr16:811155 shneg	shCHD1	OK	14,693	19,6811	0,421682	2,04705	0,0005	0,00321686	yes
MSX1	MSX1	MSX1	chr4:4861391 shneg	shCHD1	OK	3,72465	4,98853	0,421508	1,70671	0,0037	0,0174466	yes
CCDC90A	CCDC90A	CCDC90A	chr6:1378678 shneg	shCHD1	OK	10,9542	14,6706	0,421449	2,27404	5,00E-05	0,0004193	yes
CDYL	CDYL	CDYL	chr6:4706392 shneg	shCHD1	OK	4,47938	5,99627	0,420765	2,02567	0,0004	0,00266202	yes

ADD3	ADD3	ADD3	chr10:111705 shneg	shCHD1	OK	6,20064	8,29992	0,42068	2,11822	0,0003	0,0020738	yes
RPA2	RPA2	RPA2	chr1:2821804 shneg	shCHD1	OK	27,4998	36,8054	0,420499	2,21483	0,00015	0,00112331	yes
LASP1	LASP1	LASP1	chr17:370261 shneg	shCHD1	OK	105,612	141,283	0,419812	2,48849	5,00E-05	0,0004193	yes
UNG	UNG	UNG	chr12:109535 shneg	shCHD1	OK	45,0964	60,274	0,418526	2,30802	0,0001	0,00078473	yes
FLNB	FLNB	FLNB	chr3:5799412 shneg	shCHD1	OK	27,3779	36,5888	0,418394	2,4161	5,00E-05	0,0004193	yes
VGF	VGF	VGF	chr7:1008057 shneg	shCHD1	OK	50,2999	67,2071	0,418059	2,34446	5,00E-05	0,0004193	yes
AGAP1	AGAP1	AGAP1	chr2:2364027 shneg	shCHD1	OK	9,57509	12,7934	0,41804	2,13737	0,0003	0,0020738	yes
PABPC4	PABPC4	PABPC4	chr1:4002648 shneg	shCHD1	OK	30,3851	40,597	0,418006	2,30646	0,0001	0,00078473	yes
RTN2	RTN2	RTN2	chr19:459885 shneg	shCHD1	OK	36,2135	48,3777	0,417814	2,25522	0,00015	0,00112331	yes
XRN2	XRN2	XRN2	chr20:212839 shneg	shCHD1	OK	23,8715	31,8782	0,417285	2,2939	5,00E-05	0,0004193	yes
ITGAV	ITGAV	ITGAV	chr2:1874547 shneg	shCHD1	OK	31,5091	42,0445	0,416149	2,37207	0,0001	0,00078473	yes
KIF11	KIF11	KIF11	chr10:943528 shneg	shCHD1	OK	17,6381	23,5335	0,416019	2,29945	0,0001	0,00078473	yes
TPM1	TPM1	TPM1	chr15:633348 shneg	shCHD1	OK	285,128	380,349	0,415716	2,44519	0,0001	0,00078473	yes
DCTPP1	DCTPP1	DCTPP1	chr16:304350 shneg	shCHD1	OK	21,5067	28,686	0,415558	2,01835	0,0008	0,0048114	yes
CLPB	CLPB	CLPB	chr11:720034 shneg	shCHD1	OK	4,03722	5,38329	0,415126	1,95745	0,00095	0,00559279	yes
C17orf51	C17orf51	C17orf51	chr17:214315 shneg	shCHD1	OK	2,97861	3,9717	0,415114	2,05485	0,00035	0,00236888	yes
EFCAB4B	EFCAB4B	EFCAB4B	chr12:372449 shneg	shCHD1	OK	1,85125	2,46822	0,414969	1,47211	0,0114	0,0443301	yes
ANKRD32	ANKRD32	ANKRD32	chr5:9395439 shneg	shCHD1	OK	3,64864	4,86241	0,414311	1,9749	0,0009	0,00533451	yes
RANBP1	RANBP1	RANBP1	chr22:201050 shneg	shCHD1	OK	63,1115	84,1009	0,414217	2,18397	5,00E-05	0,0004193	yes
NEK2	NEK2	NEK2	chr1:2118315 shneg	shCHD1	OK	26,5168	35,3348	0,41418	2,19712	0,00015	0,00112331	yes
TNFAIP8	TNFAIP8	TNFAIP8	chr5:1186044 shneg	shCHD1	OK	11,8114	15,7352	0,413807	2,0286	0,0003	0,0020738	yes
LEMD2	LEMD2	LEMD2	chr6:3373898 shneg	shCHD1	OK	21,2953	28,3667	0,413664	2,12578	0,00025	0,00176854	yes
CYB5B	CYB5B	CYB5B	chr16:694584 shneg	shCHD1	OK	90,5149	120,539	0,413279	2,44079	5,00E-05	0,0004193	yes
GRB7	GRB7	GRB7	chr17:378941 shneg	shCHD1	OK	34,79	46,3236	0,413072	2,26975	5,00E-05	0,0004193	yes
STMN1	STMN1	STMN1	chr1:2621067 shneg	shCHD1	OK	482,809	642,848	0,413023	2,48401	5,00E-05	0,0004193	yes
SNRPE	SNRPE	SNRPE	chr1:2038307 shneg	shCHD1	OK	45,6024	60,7178	0,413011	2,23468	5,00E-05	0,0004193	yes
COX17	COX17	COX17	chr3:1193883 shneg	shCHD1	OK	51,1876	68,1543	0,41301	1,92236	0,00065	0,0040329	yes
PSMB6	PSMB6	PSMB6	chr17:469945 shneg	shCHD1	OK	95,3899	126,989	0,412791	2,22888	5,00E-05	0,0004193	yes
GLUD1	GLUD1	GLUD1	chr10:888099 shneg	shCHD1	OK	38,0932	50,7097	0,412726	2,3323	0,00015	0,00112331	yes
PGK1	PGK1	PGK1	chrX:7735966 shneg	shCHD1	OK	147,612	196,498	0,412706	2,42763	5,00E-05	0,0004193	yes
OSBP2	OSBP2	OSBP2	chr22:310907 shneg	shCHD1	OK	2,92717	3,89577	0,4124	1,95651	0,0004	0,00266202	yes
SBK1	SBK1	SBK1	chr16:283038 shneg	shCHD1	OK	4,09774	5,45203	0,411966	1,9933	0,0005	0,00321686	yes
MAD1L1	MAD1L1	MAD1L1	chr7:1855427 shneg	shCHD1	OK	12,2079	16,239	0,411651	2,09185	0,00035	0,00236888	yes
SNRPD1	SNRPD1	SNRPD1	chr18:191922 shneg	shCHD1	OK	27,1094	36,0524	0,411303	2,14435	0,0002	0,00144867	yes
CLIC4	CLIC4	CLIC4	chr1:2507175 shneg	shCHD1	OK	72,5924	96,5377	0,411275	2,40997	5,00E-05	0,0004193	yes
VAT1	VAT1	VAT1	chr17:411666 shneg	shCHD1	OK	118,763	157,927	0,41118	2,41158	5,00E-05	0,0004193	yes
PEA15	PEA15	PEA15	chr1:1601751 shneg	shCHD1	OK	20,6479	27,4557	0,411114	2,18292	5,00E-05	0,0004193	yes
ZNF22	ZNF22	ZNF22	chr10:454931 shneg	shCHD1	OK	3,31347	4,4056	0,410997	1,67329	0,00305	0,0148336	yes
MSN	MSN	MSN	chrX:6488751 shneg	shCHD1	OK	59,7219	79,3949	0,410787	2,38168	5,00E-05	0,0004193	yes
HSPD1	HSPD1	HSPD1	chr2:1983513 shneg	shCHD1	OK	223,618	297,275	0,410764	2,1671	0,0001	0,00078473	yes
MAP4K4	MAP4K4	MAP4K4	chr2:1023141 shneg	shCHD1	OK	15,4858	20,5843	0,4106	2,30332	5,00E-05	0,0004193	yes
BAG3	BAG3	BAG3	chr10:121410 shneg	shCHD1	OK	22,3922	29,7632	0,410531	2,19902	0,0002	0,00144867	yes
MB21D1	MB21D1	MB21D1	chr6:7413485 shneg	shCHD1	OK	7,02792	9,34113	0,410498	1,9477	0,00045	0,00293585	yes
IPO11	IPO11	IPO11	chr5:6170857 shneg	shCHD1	OK	8,33319	11,0739	0,410225	2,11501	0,00035	0,00236888	yes
UFD1L	UFD1L	UFD1L	chr22:194374 shneg	shCHD1	OK	11,3592	15,0851	0,409258	1,96538	0,0008	0,0048114	yes
TIPIN	TIPIN	TIPIN	chr15:666290 shneg	shCHD1	OK	7,00533	9,29968	0,408728	1,74157	0,0029	0,0142283	yes
PAM	PAM	PAM	chr5:1022015 shneg	shCHD1	OK	142,08	188,605	0,408668	2,43848	5,00E-05	0,0004193	yes
NHP2	NHP2	NHP2	chr5:1775764 shneg	shCHD1	OK	113,078	150,068	0,408298	2,23978	0,0001	0,00078473	yes
MXD3	MXD3	MXD3	chr5:1767308 shneg	shCHD1	OK	34,6264	45,953	0,408286	1,51616	0,0081	0,0334891	yes
LARS	LARS	LARS	chr5:1454925 shneg	shCHD1	OK	36,1184	47,9085	0,407547	2,33622	5,00E-05	0,0004193	yes
RIPK1	RIPK1	RIPK1	chr6:3077057 shneg	shCHD1	OK	9,19945	12,2023	0,40754	2,10535	0,00015	0,00112331	yes
SLC7A1	SLC7A1	SLC7A1	chr13:300835 shneg	shCHD1	OK	18,1764	24,1085	0,40748	2,30698	5,00E-05	0,0004193	yes
NDEL1	NDEL1	NDEL1	chr17:833917 shneg	shCHD1	OK	10,1978	13,5233	0,407194	2,00396	0,00045	0,00293585	yes
WDR89	WDR89	WDR89	chr14:640637 shneg	shCHD1	OK	5,4315	7,20054	0,406755	1,93829	0,0007	0,00428773	yes
CALM1	CALM1	CALM1	chr14:908633 shneg	shCHD1	OK	44,1801	58,5659	0,406663	2,33707	5,00E-05	0,0004193	yes
HOXB3	HOXB3	HOXB3	chr17:466262 shneg	shCHD1	OK	21,8388	28,9454	0,406439	2,22624	0,00015	0,00112331	yes
ARHGAP26	ARHGAP26	ARHGAP26	chr5:1421502 shneg	shCHD1	OK	36,9482	48,9655	0,40626	2,38521	5,00E-05	0,0004193	yes
CHCHD2	CHCHD2	CHCHD2	chr7:5616926 shneg	shCHD1	OK	315,888	418,583	0,406099	2,34075	5,00E-05	0,0004193	yes
RNF216P1	RNF216P1	RNF216P1	chr7:5013615 shneg	shCHD1	OK	9,66951	12,8032	0,404992	1,9588	0,00055	0,00350455	yes
KRT18	KRT18	KRT18	chr12:532909 shneg	shCHD1	OK	190,823	252,545	0,404307	1,78617	0,0023	0,0117566	yes
PSAT1	PSAT1	PSAT1	chr9:8091205 shneg	shCHD1	OK	41,2343	54,5678	0,404206	2,23164	5,00E-05	0,0004193	yes
TTYH3	TTYH3	TTYH3	chr7:2671602 shneg	shCHD1	OK	75,2858	99,6267	0,404154	2,37451	5,00E-05	0,0004193	yes
XPO7	XPO7	XPO7	chr8:2177717 shneg	shCHD1	OK	13,6434	18,0505	0,403838	2,18795	0,00025	0,00176854	yes
IDE	IDE	IDE	chr10:942114 shneg	shCHD1	OK	8,91378	11,7926	0,403771	2,15317	0,00015	0,00112331	yes
KIF18A	KIF18A	KIF18A	chr11:280421 shneg	shCHD1	OK	7,07485	9,35754	0,40343	1,99436	0,0006	0,00377139	yes
HSPA1B	HSPA1B	HSPA1B	chr6_qbl_hap shneg	shCHD1	OK	13,7809	18,2259	0,403318	2,05782	0,00055	0,00350455	yes
RRP7A	RRP7A	RRP7A	chr22:428965 shneg	shCHD1	OK	14,3001	18,9097	0,403103	2,20826	5,00E-05	0,0004193	yes
HSPA1A	HSPA1A	HSPA1A	chr6_qbl_hap shneg	shCHD1	OK	11,6411	15,3905	0,402815	2,00812	0,00035	0,00236888	yes
NKAP	NKAP	NKAP	chrX:1190590 shneg	shCHD1	OK	13,4721	17,8052	0,402328	1,92758	0,001	0,0058183	yes
PPP2CB	PPP2CB	PPP2CB	chr8:3064312 shneg	shCHD1	OK	15,1677	20,0374	0,401701	2,02344	0,0007	0,00428773	yes
CDCA8	CDCA8	CDCA8	chr1:3815815 shneg	shCHD1	OK	23,2277	30,6785	0,401384	2,13413	0,00015	0,00112331	yes
SLC35E4	SLC35E4	SLC35E4	chr22:310317 shneg	shCHD1	OK	6,56594	8,6691	0,40088	1,93299	0,0011	0,0063157	yes
EIF2B3	EIF2B3	EIF2B3	chr1:4531619 shneg	shCHD1	OK	7,0854	9,35472	0,400846	1,83142	0,00085	0,00508358	yes
BAIAP2L2	BAIAP2L2	BAIAP2L2	chr22:384808 shneg	shCHD1	OK	7,96775	10,5193	0,400797	1,92672	0,00105	0,00607369	yes
LYRM7	LYRM7	LYRM7	chr5:1305066 shneg	shCHD1	OK	4,73982	6,25748	0,400749	2,03555	0,0003	0,0020738	yes
HES6	HES6	HES6	chr2:2391469 shneg	shCHD1	OK	6,30095	8,31363	0,39991	1,69045	0,00375	0,0176465	yes
WDR4	WDR4	WDR4	chr21:442632 shneg	shCHD1	OK	8,13801	10,7349	0,399565	1,81452	0,0015	0,00814879	yes
CD68	CD68	CD68	chr17:748280 shneg	shCHD1	OK	9,5486	12,5938	0,399353	1,89133	0,00095	0,00559279	yes
NUDCD1	NUDCD1	NUDCD1	chr8:1102531 shneg	shCHD1	OK	6,34388	8,35548	0,397358	1,97501	0,0004	0,00266202	yes
SAE1	SAE1	SAE1	chr19:476340 shneg	shCHD1	OK	52,1193	68,6408	0,39725	2,23798	5,00E-05	0,0004193	yes
CCNH	CCNH	CCNH	chr5:8669007 shneg	shCHD1	OK	14,5376	19,1458	0,397239	1,89017	0,00175	0,00931439	yes
DOT1L	DOT1L	DOT1L	chr19:216414 shneg	shCHD1	OK	7,06029	9,29512	0,396746	2,11254	0,00015	0,00112331	yes
RNF8	RNF8	RNF8	chr6:3732174 shneg	shCHD1	OK	5,444	7,16298	0,395893	2,01333	0,0004	0,00266202	yes
NASP	NASP	NASP	chr1:4604965 shneg	shCHD1	OK	64,7326	85,1518	0,395545	2,20992	0,0002	0,00144867	yes
TMEM164	TMEM164	TMEM164	chrX:1092458 shneg	shCHD1	OK	3,74871	4,9312	0,395542	1,90075	0,0011	0,0063157	yes
TRAIP	TRAIP	TRAIP	chr3:4986602 shneg	shCHD1	OK	6,28362	8,2656	0,395523	1,88292	0,00135	0,00748275	yes
PSMA7	PSMA7	PSMA7	chr20:607117 shneg	shCHD1	OK	183,451	241,223	0,394974	2,24543	0,0001	0,00078473	yes
KIF4A	KIF4A	KIF4A	chrX:6950987 shneg	shCHD1	OK	17,4153	22,8985	0,394901	2,16348	0,0004	0,00266202	yes
CKAP2L	CKAP2L	CKAP2L	chr2:1134954 shneg	shCHD1	OK	22,4222	29,4811	0,394858	2,14926	0,0001	0,00078473	yes
PPM1F	PPM1F	PPM1F	chr22:222737 shneg	shCHD1	OK	9,15306	12,0338	0,394768	2,09186	0,0004	0,00266202	yes
NT5M	NT5M	NT5M	chr17:172066 shneg	shCHD1	OK	3,80508	5,00238	0,394686	1,48078	0,01165	0,0451385	yes
HEATR2	HEATR2	HEATR2	chr7:588833-ł shneg	shCHD1	OK	18,2106	23,9403	0,394663	1,87686	0,0011	0,0063157	yes
FBXL15	FBXL15	FBXL15	chr10:104179 shneg	shCHD1	OK	8,99773	11,828	0,39458	1,9087	0,0007	0,00428773	yes

DARS	DARS	DARS	chr2:1366642 shneg	shCHD1	OK	40,7936	53,6256	0,394579	2,18313	0,0002	0,00144867	yes
GCLM	GCLM	GCLM	chr1:9435258 shneg	shCHD1	OK	8,05667	10,5898	0,394423	1,95235	0,00065	0,0040329	yes
WWP1	WWP1	WWP1	chr8:8735499 shneg	shCHD1	OK	3,00847	3,95437	0,394421	1,84137	0,0014	0,00768951	yes
PTDSS1	PTDSS1	PTDSS1	chr8:9727416 shneg	shCHD1	OK	12,4939	16,4205	0,394279	2,00446	0,00065	0,0040329	yes
TOMM5	TOMM5	TOMM5	chr9:3758841 shneg	shCHD1	OK	68,1734	89,5882	0,394101	2,05987	0,0004	0,00266202	yes
SUGT1	SUGT1	SUGT1	chr13:532268 shneg	shCHD1	OK	22,6739	29,795	0,394038	2,03951	0,0004	0,00266202	yes
BARD1	BARD1	BARD1	chr2:2155932 shneg	shCHD1	OK	4,17553	5,48584	0,393753	1,83647	0,0014	0,00768951	yes
FH	FH	FH	chr1:2416608 shneg	shCHD1	OK	19,4997	25,6185	0,393732	2,02707	0,00045	0,00293585	yes
VDAC3	VDAC3	VDAC3	chr8:4224927 shneg	shCHD1	OK	59,4329	78,0818	0,393723	2,17611	0,0001	0,00078473	yes
MITF	MITF	MITF	chr3:6978858 shneg	shCHD1	OK	11,9734	15,7278	0,393477	2,04429	5,00E-05	0,0004193	yes
KIAA1908	KIAA1908	KIAA1908	chr7:1609708 shneg	shCHD1	OK	2,87571	3,77705	0,393341	1,80094	0,0019	0,00996052	yes
TMEM180	TMEM180	TMEM180	chr10:104221 shneg	shCHD1	OK	5,49569	7,21617	0,392931	1,90931	0,0013	0,00726051	yes
CLTB	CLTB	CLTB	chr5:1758194 shneg	shCHD1	OK	44,7143	58,7118	0,392915	2,06662	0,00045	0,00293585	yes
TPTEP1	TPTEP1	TPTEP1	chr22:170828 shneg	shCHD1	OK	5,12246	6,72601	0,392912	1,57258	0,00715	0,0300974	yes
TFPI	TFPI	TFPI	chr2:1883289 shneg	shCHD1	OK	102,723	134,847	0,392571	1,80515	0,0013	0,00726051	yes
NFE2L1	NFE2L1	NFE2L1	chr17:461256 shneg	shCHD1	OK	36,8933	48,4304	0,392555	2,25183	5,00E-05	0,0004193	yes
MYBL1	MYBL1	MYBL1	chr8:6747440 shneg	shCHD1	OK	6,58624	8,64503	0,392415	2,0016	0,0005	0,00321686	yes
MRTO4	MRTO4	MRTO4	chr1:1957807 shneg	shCHD1	OK	19,4215	25,4921	0,392397	2,05947	0,00045	0,00293585	yes
ANKRD11	ANKRD11	ANKRD11	chr16:893340 shneg	shCHD1	OK	33,9378	44,5453	0,392381	1,79105	0,0022	0,011316	yes
MRPS28	MRPS28	MRPS28	chr8:8083109 shneg	shCHD1	OK	18,7062	24,534	0,39127	1,79382	0,0019	0,00996052	yes
RBM24	RBM24	RBM24	chr6:1728180 shneg	shCHD1	OK	3,26092	4,27537	0,39077	1,63618	0,005	0,0224661	yes
TCERG1	TCERG1	TCERG1	chr5:1458268 shneg	shCHD1	OK	23,5649	30,8919	0,39059	2,16652	0,0002	0,00144867	yes
CLEC2D	CLEC2D	CLEC2D	chr12:982230 shneg	shCHD1	OK	3,79751	4,97797	0,390505	1,86536	0,0012	0,00680293	yes
CDK8	CDK8	CDK8	chr13:268287 shneg	shCHD1	OK	9,24971	12,1171	0,38957	1,89551	0,0006	0,00377139	yes
SCAMP5	SCAMP5	SCAMP5	chr15:752878 shneg	shCHD1	OK	20,7178	27,1372	0,389404	2,09782	0,00015	0,00112331	yes
PITHD1	PITHD1	PITHD1	chr1:2410487 shneg	shCHD1	OK	21,6764	28,3878	0,389148	1,99153	0,0006	0,00377139	yes
ENY2	ENY2	ENY2	chr8:1103465 shneg	shCHD1	OK	15,5717	20,3879	0,388787	2,04773	0,00045	0,00293585	yes
SLC25A37	SLC25A37	SLC25A37	chr8:2338636 shneg	shCHD1	OK	29,2385	38,2755	0,388553	2,06657	0,0006	0,00377139	yes
GALE	GALE	GALE	chr1:2412208 shneg	shCHD1	OK	4,08226	5,34309	0,388307	1,47482	0,0104	0,0410955	yes
EDIL3	EDIL3	EDIL3	chr5:8323812 shneg	shCHD1	OK	8,68369	11,3653	0,388252	1,92208	0,001	0,0058183	yes
KIF23	KIF23	KIF23	chr15:697066 shneg	shCHD1	OK	21,4052	28,0058	0,387761	2,1078	0,00035	0,00236888	yes
NPR2	NPR2	NPR2	chr9:3579240 shneg	shCHD1	OK	4,77018	6,24059	0,38764	1,68155	0,00465	0,0210977	yes
FAM8A1	FAM8A1	FAM8A1	chr6:1760051 shneg	shCHD1	OK	3,41832	4,47183	0,387577	1,82861	0,00125	0,00702633	yes
C12orf75	C12orf75	C12orf75	chr12:105724 shneg	shCHD1	OK	124,56	162,918	0,387301	2,2028	0,0001	0,00078473	yes
CWC25	CWC25	CWC25	chr17:369574 shneg	shCHD1	OK	19,0754	24,9446	0,387014	2,03266	0,0005	0,00321686	yes
POLD1	POLD1	POLD1	chr19:508875 shneg	shCHD1	OK	18,3492	23,9911	0,386786	2,07553	0,00035	0,00236888	yes
AMOTL2	AMOTL2	AMOTL2	chr3:1340741 shneg	shCHD1	OK	8,52856	11,1493	0,386582	2,02497	0,0003	0,0020738	yes
GPCPD1	GPCPD1	GPCPD1	chr20:552507 shneg	shCHD1	OK	1,7546	2,29361	0,386474	1,75799	0,0026	0,0129897	yes
NCAPG2	NCAPG2	NCAPG2	chr7:1584240 shneg	shCHD1	OK	31,1896	40,7581	0,386021	2,17216	0,0001	0,00078473	yes
BAG2	BAG2	BAG2	chr6:5703710 shneg	shCHD1	OK	15,3585	20,0654	0,385677	1,95688	0,0007	0,00428773	yes
CBFA2T2	CBFA2T2	CBFA2T2	chr20:320779 shneg	shCHD1	OK	13,7272	17,931	0,385416	2,15889	0,0001	0,00078473	yes
IFRD2	IFRD2	IFRD2	chr3:5031651 shneg	shCHD1	OK	25,0905	32,7703	0,385247	2,0445	0,0003	0,0020738	yes
TPD52	TPD52	TPD52	chr8:8094710 shneg	shCHD1	OK	9,64115	12,5905	0,385056	1,99712	0,00045	0,00293585	yes
CCDC18	CCDC18	CCDC18	chr1:9364628 shneg	shCHD1	OK	4,05076	5,28848	0,384663	1,83465	0,00145	0,00793286	yes
RARS	RARS	RARS	chr5:1679134 shneg	shCHD1	OK	25,5092	33,2929	0,384196	2,04318	0,0005	0,00321686	yes
PARP2	PARP2	PARP2	chr14:208117 shneg	shCHD1	OK	12,3562	16,1237	0,383944	1,86429	0,0014	0,00768951	yes
MTHFD1	MTHFD1	MTHFD1	chr14:648547 shneg	shCHD1	OK	41,0104	53,5108	0,38384	2,17014	5,00E-05	0,0004193	yes
IVNS1ABP	IVNS1ABP	IVNS1ABP	chr1:1852655 shneg	shCHD1	OK	16,6424	21,7119	0,383624	2,08563	0,00025	0,00176854	yes
ZNF703	ZNF703	ZNF703	chr8:3755330 shneg	shCHD1	OK	48,7519	63,596	0,383477	2,12108	0,00015	0,00112331	yes
MTF2	MTF2	MTF2	chr1:9354479 shneg	shCHD1	OK	6,92159	9,028	0,383303	1,9264	0,001	0,0058183	yes
RDH10	RDH10	RDH10	chr8:7420683 shneg	shCHD1	OK	56,652	73,8517	0,382504	2,21304	5,00E-05	0,0004193	yes
LOC493754	LOC493754	LOC493754	chr7:6601855 shneg	shCHD1	OK	4,93648	6,43476	0,382403	1,83368	0,00135	0,00748275	yes
NT5DC2	NT5DC2	NT5DC2	chr3:5252935 shneg	shCHD1	OK	44,2037	57,6148	0,382272	2,08809	0,0003	0,0020738	yes
ALKBH8	ALKBH8	ALKBH8	chr11:107373 shneg	shCHD1	OK	5,04189	6,57118	0,382186	1,85008	0,00135	0,00748275	yes
IFI35	IFI35	IFI35	chr17:411587 shneg	shCHD1	OK	40,5474	52,8388	0,38199	2,00359	0,00055	0,00350455	yes
MACROD1	MACROD1	MACROD1	chr11:637660 shneg	shCHD1	OK	25,9178	33,769	0,381755	1,88366	0,00085	0,00508358	yes
TMEM41A	TMEM41A	TMEM41A	chr3:1852073 shneg	shCHD1	OK	19,6191	25,5603	0,381645	2,03888	0,0002	0,00144867	yes
CMTM7	CMTM7	CMTM7	chr3:3243316 shneg	shCHD1	OK	15,9549	20,781	0,381265	1,83385	0,0015	0,00814879	yes
KIF22	KIF22	KIF22	chr16:298020 shneg	shCHD1	OK	28,3004	36,8547	0,381028	2,05605	0,0006	0,00377139	yes
KIAA1967	KIAA1967	KIAA1967	chr8:2246214 shneg	shCHD1	OK	24,7758	32,2542	0,380558	1,98372	0,00065	0,0040329	yes
PCM1	PCM1	PCM1	chr8:1778036 shneg	shCHD1	OK	8,0017	10,4134	0,380061	2,06898	0,00055	0,00350455	yes
TUBB4A	TUBB4A	TUBB4A	chr19:649432 shneg	shCHD1	OK	6,83252	8,89036	0,379825	1,79196	0,00255	0,0127904	yes
PPP2R2A	PPP2R2A	PPP2R2A	chr8:2614900 shneg	shCHD1	OK	8,6857	11,2978	0,379325	1,93064	0,00095	0,00559279	yes
SH3GL1	SH3GL1	SH3GL1	chr19:436036 shneg	shCHD1	OK	27,3712	35,5773	0,378301	2,0156	0,00035	0,00236888	yes
TOP3A	TOP3A	TOP3A	chr17:181772 shneg	shCHD1	OK	8,02425	10,4279	0,378011	1,93832	0,00065	0,0040329	yes
CISH	CISH	CISH	chr3:5064388 shneg	shCHD1	OK	4,25134	5,52239	0,377378	1,64652	0,0039	0,018217	yes
PPP1R3F	PPP1R3F	PPP1R3F	chrX:4912630 shneg	shCHD1	OK	5,67508	7,37161	0,37734	1,81639	0,00155	0,00838119	yes
CDCA4	CDCA4	CDCA4	chr14:105475 shneg	shCHD1	OK	23,0565	29,9445	0,377115	1,98789	0,0005	0,00321686	yes
MTMR9LP	MTMR9LP	MTMR9LP	chr1:3269726 shneg	shCHD1	OK	6,99151	9,07985	0,377063	1,79464	0,00175	0,00931439	yes
RGNEF	RGNEF	RGNEF	chr5:7292198 shneg	shCHD1	OK	31,5155	40,9276	0,377014	2,13825	0,00015	0,00112331	yes
UBR7	UBR7	UBR7	chr14:936692 shneg	shCHD1	OK	10,1128	13,1271	0,376366	1,77407	0,00265	0,0132017	yes
ALDH3A1	ALDH3A1	ALDH3A1	chr17:196412 shneg	shCHD1	OK	70,7844	91,873	0,376209	2,08888	0,00025	0,00176854	yes
SLC1A5	SLC1A5	SLC1A5	chr19:472781 shneg	shCHD1	OK	45,7031	59,3119	0,37603	2,01014	0,0003	0,0020738	yes
TPCN1	TPCN1	TPCN1	chr12:113659 shneg	shCHD1	OK	8,27404	10,7344	0,375576	1,97674	0,00075	0,00455593	yes
PRIM2	PRIM2	PRIM2	chr6:5718242 shneg	shCHD1	OK	9,86481	12,7898	0,374632	1,82496	0,00175	0,00931439	yes
NDFIP1	NDFIP1	NDFIP1	chr5:1414883 shneg	shCHD1	OK	56,4254	73,1481	0,374476	2,15651	0,0002	0,00144867	yes
CCDC112	CCDC112	CCDC112	chr5:1146028 shneg	shCHD1	OK	2,47655	3,21037	0,374407	1,43889	0,0124	0,0475026	yes
FBXL6	FBXL6	FBXL6	chr8:1455790 shneg	shCHD1	OK	15,978	20,7037	0,373799	1,88072	0,00075	0,00455593	yes
GEMIN2	GEMIN2	GEMIN2	chr14:395834 shneg	shCHD1	OK	5,10543	6,6125	0,373164	1,44903	0,0111	0,0433694	yes
SLC25A11	SLC25A11	SLC25A11	chr17:484042 shneg	shCHD1	OK	22,6136	29,2886	0,373149	1,96062	0,00085	0,00508358	yes
WARS	WARS	WARS	chr14:100800 shneg	shCHD1	OK	26,5595	34,3892	0,372729	2,00124	0,00075	0,00455593	yes
HN1L	HN1L	HN1L	chr16:172827 shneg	shCHD1	OK	35,0993	45,4334	0,372309	2,10095	0,00025	0,00176854	yes
TXNDC5	TXNDC5	TXNDC5	chr6:7727010 shneg	shCHD1	OK	57,0973	73,9052	0,372254	1,95486	0,00025	0,00176854	yes
C5orf54	C5orf54	C5orf54	chr5:1598201 shneg	shCHD1	OK	2,60523	3,37198	0,372182	1,52437	0,00965	0,0388146	yes
C20orf72	C20orf72	C20orf72	chr20:179497 shneg	shCHD1	OK	5,13907	6,65053	0,371962	1,7263	0,0025	0,012594	yes
GNL2	GNL2	GNL2	chr1:3802251 shneg	shCHD1	OK	22,7571	29,4496	0,371932	1,9827	0,0004	0,00266202	yes
TP53I11	TP53I11	TP53I11	chr11:447859 shneg	shCHD1	OK	9,72581	12,5833	0,37162	1,90413	0,00105	0,00607369	yes
SEC61G	SEC61G	SEC61G	chr7:5481993 shneg	shCHD1	OK	129,189	167,123	0,371428	1,91503	0,001	0,0058183	yes
TONSL	TONSL	TONSL	chr8:1456541 shneg	shCHD1	OK	14,5945	18,8771	0,371213	2,00154	0,0005	0,00321686	yes
SSBP3	SSBP3	SSBP3	chr1:5469219 shneg	shCHD1	OK	9,40174	12,1594	0,371073	1,77253	0,0016	0,00862477	yes
FSCN1	FSCN1	FSCN1	chr7:5632435 shneg	shCHD1	OK	199,491	257,996	0,371021	2,20741	0,0003	0,0020738	yes
BECN1	BECN1	BECN1	chr17:409508 shneg	shCHD1	OK	30,4232	39,327	0,370348	1,98329	0,0005	0,00321686	yes
ANK2	ANK2	ANK2	chr4:1137392 shneg	shCHD1	OK	4,61521	5,96576	0,37031	1,73601	0,0028	0,0138449	yes

HNRNPA1L2	HNRNPA1L2	HNRNPA1L2	chr13:531916	shneg	shCHD1	OK	3,63289	4,69454	0,369863	1,56703	0,0073	0,030664	yes
CHRAC1	CHRAC1	CHRAC1	chr8:1415213	shneg	shCHD1	OK	14,045	18,1481	0,369764	1,91724	0,00095	0,00559279	yes
CSF3	CSF3	CSF3	chr17:381716	shneg	shCHD1	OK	32,2407	41,6577	0,369702	1,91488	0,00065	0,0040329	yes
KIF13A	KIF13A	KIF13A	chr6:1775941	shneg	shCHD1	OK	4,58766	5,92735	0,369629	1,51417	0,0082	0,0338022	yes
SUV39H1	SUV39H1	SUV39H1	chrX:4855513	shneg	shCHD1	OK	9,94373	12,8435	0,36918	1,8457	0,0012	0,00680293	yes
PRSS16	PRSS16	PRSS16	chr6:2721550	shneg	shCHD1	OK	3,9898	5,15299	0,369094	1,74997	0,0024	0,0121606	yes
RBBP4	RBBP4	RBBP4	chr1:3311674	shneg	shCHD1	OK	21,2107	27,3931	0,369021	1,88306	0,0009	0,00533451	yes
ETF1	ETF1	ETF1	chr5:1378417	shneg	shCHD1	OK	37,5142	48,4305	0,368477	2,0938	0,0005	0,00321686	yes
VDAC1	VDAC1	VDAC1	chr5:1333075	shneg	shCHD1	OK	135,542	174,919	0,367943	2,12865	5,00E-05	0,0004193	yes
ZNF184	ZNF184	ZNF184	chr6:2741852	shneg	shCHD1	OK	3,53229	4,55793	0,367776	1,7245	0,00275	0,0136364	yes
TRIM27	TRIM27	TRIM27	chr6_ssto_ha	shneg	shCHD1	OK	29,9701	38,6712	0,367736	2,02729	0,0004	0,00266202	yes
ZNF706	ZNF706	ZNF706	chr8:1022092	shneg	shCHD1	OK	13,8246	17,8372	0,367651	1,87622	0,0011	0,0063157	yes
E2F1	E2F1	E2F1	chr20:322632	shneg	shCHD1	OK	14,1019	18,1925	0,367451	1,90127	0,00095	0,00559279	yes
SLC43A2	SLC43A2	SLC43A2	chr17:147769	shneg	shCHD1	OK	3,08109	3,97454	0,36735	1,63257	0,00405	0,0187979	yes
CYC1	CYC1	CYC1	chr8:1451499	shneg	shCHD1	OK	135,343	174,533	0,366873	2,07356	0,0003	0,0020738	yes
SQRDL	SQRDL	SQRDL	chr15:459272	shneg	shCHD1	OK	199,596	257,36	0,366705	2,14746	0,0003	0,0020738	yes
FLJ42709	FLJ42709	FLJ42709	chr5:9274506	shneg	shCHD1	OK	21,7513	28,0417	0,366471	1,60909	0,00565	0,0248732	yes
MAFF	MAFF	MAFF	chr22:385979	shneg	shCHD1	OK	4,49342	5,79257	0,366389	1,66984	0,0039	0,018217	yes
INTS9	INTS9	INTS9	chr8:2862517	shneg	shCHD1	OK	4,21396	5,43202	0,366311	1,68364	0,00325	0,0156858	yes
PDLIM1	PDLIM1	PDLIM1	chr10:969973	shneg	shCHD1	OK	7,45179	9,60507	0,366209	1,66659	0,00395	0,0184012	yes
NXT2	NXT2	NXT2	chrX:1087790	shneg	shCHD1	OK	2,64592	3,4093	0,365707	1,46454	0,0104	0,0410955	yes
CD274	CD274	CD274	chr9:5450502	shneg	shCHD1	OK	9,53233	12,2734	0,364638	1,87676	0,0011	0,0063157	yes
ZNF362	ZNF362	ZNF362	chr1:3372217	shneg	shCHD1	OK	7,47845	9,62752	0,364425	1,78698	0,00185	0,00975716	yes
DCUN1D2	DCUN1D2	DCUN1D2	chr13:114110	shneg	shCHD1	OK	5,29767	6,81825	0,364043	1,74595	0,0032	0,015482	yes
ADAM15	ADAM15	ADAM15	chr1:1550062	shneg	shCHD1	OK	26,5967	34,2286	0,363953	1,83403	0,0011	0,0063157	yes
NUDC	NUDC	NUDC	chr1:2724822	shneg	shCHD1	OK	79,6322	102,482	0,363947	2,01631	0,0002	0,00144867	yes
STK17A	STK17A	STK17A	chr7:4362269	shneg	shCHD1	OK	5,43131	6,98936	0,363859	1,76502	0,00215	0,0110834	yes
HSPA14	HSPA14	HSPA14	chr10:148802	shneg	shCHD1	OK	8,23356	10,5924	0,363434	1,74936	0,00245	0,0123735	yes
NRM	NRM	NRM	chr6_qbl_hap	shneg	shCHD1	OK	24,7643	31,8564	0,36332	1,85127	0,00155	0,00838119	yes
PARP1	PARP1	PARP1	chr1:2265483	shneg	shCHD1	OK	51,4019	66,1203	0,363271	2,09376	0,00025	0,00176854	yes
ACSL4	ACSL4	ACSL4	chrX:1088845	shneg	shCHD1	OK	10,677	13,7304	0,362869	1,91912	0,0008	0,0048114	yes
YARS	YARS	YARS	chr1:3324083	shneg	shCHD1	OK	34,3041	44,1125	0,362805	1,77821	0,00165	0,00886	yes
TUFT1	TUFT1	TUFT1	chr1:1515127	shneg	shCHD1	OK	3,88987	5,00027	0,362283	1,73479	0,00235	0,0119595	yes
ZIC5	ZIC5	ZIC5	chr13:100615	shneg	shCHD1	OK	5,6882	7,31181	0,362255	1,80759	0,0015	0,00814879	yes
GPAT2	GPAT2	GPAT2	chr2:9667629	shneg	shCHD1	OK	18,2141	23,4046	0,361736	1,87199	0,001	0,0058183	yes
POLD3	POLD3	POLD3	chr11:743036	shneg	shCHD1	OK	8,88258	11,4138	0,361733	1,83647	0,00175	0,00931439	yes
C5orf62	C5orf62	C5orf62	chr5:1501575	shneg	shCHD1	OK	3,51794	4,51899	0,361271	1,52858	0,0077	0,0321599	yes
PLSCR1	PLSCR1	PLSCR1	chr3:1462329	shneg	shCHD1	OK	16,3535	21,0068	0,36126	1,85977	0,00135	0,00748275	yes
C6orf62	C6orf62	C6orf62	chr6:2466726	shneg	shCHD1	OK	22,7543	29,223	0,360964	1,71976	0,003	0,0146313	yes
SNRPC	SNRPC	SNRPC	chr6:3472487	shneg	shCHD1	OK	65,6296	84,2822	0,360882	1,96515	0,00045	0,00293585	yes
ATAD3A	ATAD3A	ATAD3A	chr1:1447522	shneg	shCHD1	OK	10,1311	13,01	0,360827	1,76606	0,0023	0,0117566	yes
HMGB1	HMGB1	HMGB1	chr13:310328	shneg	shCHD1	OK	185,122	237,663	0,360444	2,15632	0,00015	0,00112331	yes
CCDC15	CCDC15	CCDC15	chr11:124824	shneg	shCHD1	OK	2,06494	2,65091	0,36039	1,53839	0,00775	0,03233	yes
ECM1	ECM1	ECM1	chr1:1504804	shneg	shCHD1	OK	48,7145	62,5377	0,360375	2,01144	0,0002	0,00144867	yes
LAPTM4B	LAPTM4B	LAPTM4B	chr8:9878780	shneg	shCHD1	OK	39,8996	51,2178	0,360271	1,98467	0,00055	0,00350455	yes
MANF	MANF	MANF	chr3:5142269	shneg	shCHD1	OK	65,2131	83,7085	0,360211	1,91596	0,001	0,0058183	yes
TFE3	TFE3	TFE3	chrX:4888624	shneg	shCHD1	OK	24,0414	30,8497	0,359737	1,97374	0,0005	0,00321686	yes
PRPF8	PRPF8	PRPF8	chr17:155392	shneg	shCHD1	OK	52,5859	67,4596	0,359349	2,12286	0,00035	0,00236888	yes
AHDC1	AHDC1	AHDC1	chr1:2786075	shneg	shCHD1	OK	2,07296	2,65917	0,359285	1,72612	0,0028	0,0138449	yes
GTF2IRD1	GTF2IRD1	GTF2IRD1	chr7:7386811	shneg	shCHD1	OK	12,8587	16,4949	0,359277	1,8832	0,0008	0,0048114	yes
PIP4K2A	PIP4K2A	PIP4K2A	chr10:228237	shneg	shCHD1	OK	21,4719	27,5435	0,359263	1,97397	0,00085	0,00508358	yes
CDKAL1	CDKAL1	CDKAL1	chr6:2053468	shneg	shCHD1	OK	6,29298	8,07184	0,359154	1,7349	0,0029	0,0142283	yes
TDP1	TDP1	TDP1	chr14:904222	shneg	shCHD1	OK	7,24773	9,29644	0,35915	1,79169	0,0012	0,00680293	yes
FLII	FLII	FLII	chr17:181289	shneg	shCHD1	OK	38,4514	49,3123	0,358911	1,78232	0,00155	0,00838119	yes
SCRN2	SCRN2	SCRN2	chr17:459089	shneg	shCHD1	OK	10,932	14,0186	0,358785	1,59712	0,00505	0,0226106	yes
PWWP2A	PWWP2A	PWWP2A	chr5:1595028	shneg	shCHD1	OK	6,56441	8,41726	0,358685	1,76699	0,0017	0,00909693	yes
CCBL1	CCBL1	CCBL1	chr9:1315953	shneg	shCHD1	OK	5,12833	6,57563	0,358639	1,5988	0,00605	0,0262198	yes
TIMELESS	TIMELESS	TIMELESS	chr12:568101	shneg	shCHD1	OK	17,667	22,6515	0,358553	1,9843	0,00035	0,00236888	yes
GBE1	GBE1	GBE1	chr3:8153884	shneg	shCHD1	OK	8,3998	10,769	0,35845	1,78236	0,0021	0,0108578	yes
DCAF13	DCAF13	DCAF13	chr8:1044108	shneg	shCHD1	OK	19,2462	24,6734	0,35838	1,59909	0,00525	0,0232593	yes
CNN3	CNN3	CNN3	chr1:9536250	shneg	shCHD1	OK	25,1172	32,1936	0,358097	1,89884	0,0012	0,00680293	yes
TRABD	TRABD	TRABD	chr22:506243	shneg	shCHD1	OK	16,144	20,6915	0,358044	1,84408	0,0013	0,00726051	yes
LRRFIP2	LRRFIP2	LRRFIP2	chr3:3709411	shneg	shCHD1	OK	10,3911	13,3178	0,358011	1,80985	0,0019	0,00996052	yes
GAMT	GAMT	GAMT	chr19:139702	shneg	shCHD1	OK	41,0088	52,5506	0,357772	1,84722	0,0011	0,0063157	yes
YEATS4	YEATS4	YEATS4	chr12:697535	shneg	shCHD1	OK	13,3044	17,0482	0,357718	1,72854	0,0029	0,0142283	yes
POC1A	POC1A	POC1A	chr3:5210924	shneg	shCHD1	OK	9,95913	12,7614	0,3577	1,71889	0,0031	0,0150558	yes
PSMA3	PSMA3	PSMA3	chr14:587115	shneg	shCHD1	OK	96,4289	123,525	0,357265	1,60603	0,00575	0,0252576	yes
PSMA2	PSMA2	PSMA2	chr7:4295646	shneg	shCHD1	OK	72,9475	93,4437	0,357239	1,98207	0,00045	0,00293585	yes
AGPS	AGPS	AGPS	chr2:1782574	shneg	shCHD1	OK	6,23418	7,98456	0,357011	1,88467	0,00125	0,00702633	yes
RNF219	RNF219	RNF219	chr13:791884	shneg	shCHD1	OK	6,01565	7,70377	0,356843	1,72838	0,0027	0,0134267	yes
SLC45A3	SLC45A3	SLC45A3	chr1:2056269	shneg	shCHD1	OK	4,13347	5,29335	0,356828	1,69572	0,00335	0,0160296	yes
XRCC2	XRCC2	XRCC2	chr7:1523435	shneg	shCHD1	OK	13,2584	16,9772	0,356699	1,86159	0,0014	0,00768951	yes
RPUSD3	RPUSD3	RPUSD3	chr3:9879532	shneg	shCHD1	OK	17,7608	22,7407	0,356576	1,70508	0,00305	0,0148336	yes
SNRNP25	SNRNP25	SNRNP25	chr16:103828	shneg	shCHD1	OK	22,49	28,7912	0,356342	1,72687	0,00305	0,0148336	yes
POLD2	POLD2	POLD2	chr7:4415428	shneg	shCHD1	OK	82,6659	105,805	0,356045	2,00642	0,00065	0,0040329	yes
TEX10	TEX10	TEX10	chr9:1030643	shneg	shCHD1	OK	22,8556	29,2515	0,355962	1,92977	0,00065	0,0040329	yes
PHF14	PHF14	PHF14	chr7:1101349	shneg	shCHD1	OK	14,7054	18,8195	0,355886	1,65728	0,0042	0,0193522	yes
MED1	MED1	MED1	chr17:375605	shneg	shCHD1	OK	23,2924	29,8029	0,355595	2,04391	0,0003	0,0020738	yes
TCF12	TCF12	TCF12	chr15:572108	shneg	shCHD1	OK	14,8781	19,0301	0,355096	1,91733	0,0008	0,0048114	yes
DAZAP1	DAZAP1	DAZAP1	chr19:140758	shneg	shCHD1	OK	58,7195	75,0805	0,354599	2,00226	0,0005	0,00321686	yes
HNF1B	HNF1B	HNF1B	chr17:360464	shneg	shCHD1	OK	69,275	88,5755	0,354575	2,03325	0,00045	0,00293585	yes
CDKN3	CDKN3	CDKN3	chr14:548636	shneg	shCHD1	OK	25,9654	33,1984	0,354524	1,64395	0,0048	0,0216934	yes
HIC1	HIC1	HIC1	chr17:195839	shneg	shCHD1	OK	2,35442	3,01024	0,354504	1,45991	0,01115	0,0435525	yes
SFXN1	SFXN1	SFXN1	chr5:1749055	shneg	shCHD1	OK	29,481	37,6877	0,354308	1,95301	0,0007	0,00428773	yes
HCG11	HCG11	HCG11	chr6:2652193	shneg	shCHD1	OK	4,56441	5,833	0,353809	1,76151	0,0019	0,00996052	yes
CDK5R2	CDK5R2	CDK5R2	chr2:2198243	shneg	shCHD1	OK	6,49829	8,30347	0,353655	1,64316	0,00505	0,0226106	yes
BEAN1	BEAN1	BEAN1	chr16:664611	shneg	shCHD1	OK	32,1618	41,0946	0,353601	1,92953	0,00095	0,00559279	yes
TTK	TTK	TTK	chr6:8071432	shneg	shCHD1	OK	14,9896	19,1505	0,35342	1,85766	0,0015	0,00814879	yes
FGFR4	FGFR4	FGFR4	chr5:1765139	shneg	shCHD1	OK	17,8572	22,8123	0,353307	1,87202	0,001	0,0058183	yes
PKD1P1	PKD1P1	PKD1P1	chr16:164114	shneg	shCHD1	OK	29,9162	38,2083	0,35296	2,0207	0,0005	0,00321686	yes
RP9	RP9	RP9	chr7:3313440	shneg	shCHD1	OK	7,30935	9,33491	0,352893	1,44123	0,01295	0,0491768	yes
RNASEH2A	RNASEH2A	RNASEH2A	chr19:129174	shneg	shCHD1	OK	41,9801	53,6119	0,352846	1,84355	0,00165	0,00886	yes
SRSF8	SRSF8	SRSF8	chr11:948000	shneg	shCHD1	OK	28,5353	36,4303	0,352392	1,98055	0,00055	0,00350455	yes

THSD1P1	THSD1P1	THSD1P1	chr13:527418 shneg	shCHD1	OK	2,56762	3,27787	0,352324	1,55084	0,00705	0,0297484	yes
USP1	USP1	USP1	chr1:6290197 shneg	shCHD1	OK	15,5028	19,7886	0,352148	1,85473	0,0018	0,00951868	yes
SPAG7	SPAG7	SPAG7	chr17:486252 shneg	shCHD1	OK	36,8995	47,1005	0,352143	1,79819	0,0017	0,00909693	yes
C11orf84	C11orf84	C11orf84	chr11:635809 shneg	shCHD1	OK	17,2669	22,0398	0,352099	1,79709	0,0018	0,00951868	yes
FAM40B	FAM40B	FAM40B	chr7:1290742 shneg	shCHD1	OK	4,33505	5,53299	0,352009	1,56156	0,00645	0,0276097	yes
MRPL22	MRPL22	MRPL22	chr5:1543206 shneg	shCHD1	OK	7,74061	9,87712	0,351643	1,74018	0,0021	0,0108578	yes
NCL	NCL	NCL	chr2:2323194 shneg	shCHD1	OK	237,113	302,493	0,351325	2,09631	0,00015	0,00112331	yes
LANCL2	LANCL2	LANCL2	chr7:5543314 shneg	shCHD1	OK	4,22117	5,38149	0,350364	1,66612	0,00375	0,0176465	yes
KIF13B	KIF13B	KIF13B	chr8:2892479 shneg	shCHD1	OK	1,216	1,55013	0,350238	1,64512	0,00425	0,0195438	yes
GTSE1	GTSE1	GTSE1	chr22:466926 shneg	shCHD1	OK	14,5057	18,4901	0,350132	1,8424	0,0018	0,00951868	yes
CBX3	CBX3	CBX3	chr7:2624083 shneg	shCHD1	OK	89,3178	113,846	0,35007	2,01209	0,00055	0,00350455	yes
TPX2	TPX2	TPX2	chr20:303269 shneg	shCHD1	OK	37,7471	48,0996	0,34966	1,97795	0,0003	0,0020738	yes
SMYD4	SMYD4	SMYD4	chr17:168282 shneg	shCHD1	OK	5,31895	6,77648	0,349396	1,71612	0,0027	0,0134267	yes
GLRX3	GLRX3	GLRX3	chr10:131934 shneg	shCHD1	OK	43,7933	55,7924	0,349359	1,85464	0,00115	0,00656221	yes
PSMB2	PSMB2	PSMB2	chr1:3606514 shneg	shCHD1	OK	18,7181	23,845	0,349254	1,93597	0,00045	0,00293585	yes
POMT2	POMT2	POMT2	chr14:777412 shneg	shCHD1	OK	8,53932	10,8774	0,349136	1,8293	0,0015	0,00814879	yes
PGP	PGP	PGP	chr16:226160 shneg	shCHD1	OK	9,20384	11,7201	0,348676	1,75166	0,00245	0,0123735	yes
ARMC9	ARMC9	ARMC9	chr2:2320633 shneg	shCHD1	OK	39,8372	50,7216	0,348486	1,91807	0,00085	0,00508358	yes
TMED9	TMED9	TMED9	chr5:1770192 shneg	shCHD1	OK	148,255	188,704	0,348048	1,99558	0,00055	0,00350455	yes
SLC20A1	SLC20A1	SLC20A1	chr2:1134034 shneg	shCHD1	OK	20,476	26,0625	0,348042	1,88684	0,0007	0,00428773	yes
C7orf46	C7orf46	C7orf46	chr7:2371974 shneg	shCHD1	OK	6,30955	8,03031	0,34792	1,44728	0,0107	0,0420661	yes
PRKDC	PRKDC	PRKDC	chr8:4868566 shneg	shCHD1	OK	41,4457	52,7419	0,347727	2,07996	0,00025	0,00176854	yes
KCMF1	KCMF1	KCMF1	chr2:8519823 shneg	shCHD1	OK	6,49551	8,26522	0,34761	1,84609	0,00085	0,00508358	yes
AMH	AMH	AMH	chr19:224911 shneg	shCHD1	OK	15,633	19,8906	0,347493	1,72894	0,0021	0,0108578	yes
PNO1	PNO1	PNO1	chr2:6838500 shneg	shCHD1	OK	14,0183	17,8326	0,347203	1,73218	0,00305	0,0148336	yes
MRPS26	MRPS26	MRPS26	chr20:302667 shneg	shCHD1	OK	18,9977	24,1663	0,347168	1,67162	0,0032	0,015482	yes
DOCK11	DOCK11	DOCK11	chrX:1176298 shneg	shCHD1	OK	21,145	26,8946	0,346997	1,9571	0,0007	0,00428773	yes
KHSRP	KHSRP	KHSRP	chr19:641311 shneg	shCHD1	OK	55,1667	70,1523	0,346695	1,98539	0,0006	0,00377139	yes
ELP3	ELP3	ELP3	chr8:2795058 shneg	shCHD1	OK	4,5432	5,77633	0,346444	1,66004	0,00365	0,0172576	yes
EXTL3	EXTL3	EXTL3	chr8:2855915 shneg	shCHD1	OK	10,5139	13,3648	0,346144	1,87314	0,00075	0,00455593	yes
TEAD3	TEAD3	TEAD3	chr6:3544137 shneg	shCHD1	OK	5,58209	7,09538	0,346075	1,66192	0,0033	0,0158557	yes
CCDC88C	CCDC88C	CCDC88C	chr14:917376 shneg	shCHD1	OK	2,74698	3,49152	0,346008	1,67934	0,00455	0,0207047	yes
IFI6	IFI6	IFI6	chr1:2799257 shneg	shCHD1	OK	168,844	214,585	0,345858	1,94319	0,0009	0,00533451	yes
CCT5	CCT5	CCT5	chr5:1025028 shneg	shCHD1	OK	99,6594	126,651	0,345779	2,03097	0,0002	0,00144867	yes
DCLRE1A	DCLRE1A	DCLRE1A	chr10:115594 shneg	shCHD1	OK	2,94326	3,73952	0,345438	1,63923	0,00415	0,0191853	yes
DNMT1	DNMT1	DNMT1	chr19:102440 shneg	shCHD1	OK	37,2148	47,2822	0,345423	1,98649	0,00075	0,00455593	yes
FAM83D	FAM83D	FAM83D	chr20:375549 shneg	shCHD1	OK	16,3301	20,7379	0,344736	1,78985	0,003	0,0146313	yes
TOMM40	TOMM40	TOMM40	chr19:453944 shneg	shCHD1	OK	37,0764	47,0786	0,344571	1,84555	0,0015	0,00814879	yes
NAA16	NAA16	NAA16	chr13:418853 shneg	shCHD1	OK	4,73076	6,00677	0,344518	1,54843	0,0064	0,0274548	yes
MPST	MPST	MPST	chr22:374156 shneg	shCHD1	OK	40,1598	50,9814	0,344219	1,8155	0,00125	0,00702633	yes
FBXO5	FBXO5	FBXO5	chr6:1532916 shneg	shCHD1	OK	22,9955	29,1864	0,343944	1,80485	0,0017	0,00909693	yes
PNN	PNN	PNN	chr14:396443 shneg	shCHD1	OK	43,6765	55,4211	0,343578	1,95619	0,00065	0,0040329	yes
SUV39H2	SUV39H2	SUV39H2	chr10:149207 shneg	shCHD1	OK	6,88117	8,72943	0,343235	1,62687	0,00455	0,0207047	yes
EPB41L4A	EPB41L4A	EPB41L4A	chr5:1114983 shneg	shCHD1	OK	1,70742	2,16557	0,342935	1,46404	0,0115	0,0446442	yes
PTPN12	PTPN12	PTPN12	chr7:7716677 shneg	shCHD1	OK	32,2852	40,93	0,342285	1,8943	0,0008	0,0048114	yes
NOP58	NOP58	NOP58	chr2:2031305 shneg	shCHD1	OK	33,2996	42,2143	0,342229	1,84154	0,0016	0,00862477	yes
PWWP2B	PWWP2B	PWWP2B	chr10:134210 shneg	shCHD1	OK	7,444	9,43449	0,341865	1,60515	0,005	0,0224661	yes
ZNF664	ZNF664	ZNF664	chr12:124457 shneg	shCHD1	OK	48,9976	62,0971	0,341814	1,97557	0,00035	0,00236888	yes
RMND5B	RMND5B	RMND5B	chr5:1775580 shneg	shCHD1	OK	23,0474	29,2079	0,341755	1,77477	0,0024	0,0121606	yes
C17orf53	C17orf53	C17orf53	chr17:422192 shneg	shCHD1	OK	4,0176	5,09135	0,341712	1,5855	0,0064	0,0274548	yes
MRPL13	MRPL13	MRPL13	chr8:1214080 shneg	shCHD1	OK	24,6485	31,2229	0,341103	1,67674	0,00385	0,0180379	yes
SNRPB	SNRPB	SNRPB	chr20:244228 shneg	shCHD1	OK	107,549	136,222	0,340965	1,87479	0,0007	0,00428773	yes
MYO9B	MYO9B	MYO9B	chr19:171865 shneg	shCHD1	OK	19,6767	24,9182	0,340715	1,93633	0,0008	0,0048114	yes
MRPL39	MRPL39	MRPL39	chr21:269579 shneg	shCHD1	OK	21,3704	27,06	0,340546	1,64051	0,0046	0,0208912	yes
PLK1	PLK1	PLK1	chr16:236902 shneg	shCHD1	OK	27,495	34,8138	0,340489	1,78538	0,00175	0,00931439	yes
SPR	SPR	SPR	chr2:7311451 shneg	shCHD1	OK	49,3821	62,5145	0,340204	1,83962	0,00125	0,00702633	yes
LOC389831	LOC389831	LOC389831	chr7_gl00019 shneg	shCHD1	OK	26,7873	33,9095	0,340144	1,83382	0,00205	0,0106507	yes
WDR91	WDR91	WDR91	chr7:1348685 shneg	shCHD1	OK	6,1556	7,79219	0,34013	1,71577	0,0033	0,0158557	yes
MMP10	MMP10	MMP10	chr11:102641 shneg	shCHD1	OK	36,5384	46,2407	0,33975	1,82604	0,00205	0,0106507	yes
CSNK1G3	CSNK1G3	CSNK1G3	chr5:1228477 shneg	shCHD1	OK	10,7031	13,5446	0,339698	1,78159	0,00175	0,00931439	yes
AHSA1	AHSA1	AHSA1	chr14:779243 shneg	shCHD1	OK	64,4335	81,5383	0,339666	1,86042	0,00145	0,00793286	yes
ITGA5	ITGA5	ITGA5	chr12:547890 shneg	shCHD1	OK	14,7006	18,5995	0,339388	1,82937	0,0016	0,00862477	yes
OAS3	OAS3	OAS3	chr12:113376 shneg	shCHD1	OK	75,2742	95,2268	0,339212	2,01628	0,00065	0,0040329	yes
NUP93	NUP93	NUP93	chr16:567640 shneg	shCHD1	OK	63,5806	80,4313	0,339172	1,93739	0,00075	0,00455593	yes
NPIP	NPIP	NPIP	chr16:150312 shneg	shCHD1	OK	35,2252	44,5581	0,33908	1,73607	0,00175	0,00931439	yes
ITSN1	ITSN1	ITSN1	chr21:350147 shneg	shCHD1	OK	6,17686	7,81261	0,338932	1,73872	0,00245	0,0123735	yes
MAP3K1	MAP3K1	MAP3K1	chr5:5611089 shneg	shCHD1	OK	7,63976	9,66171	0,338751	1,81815	0,00135	0,00748275	yes
CCDC117	CCDC117	CCDC117	chr22:291686 shneg	shCHD1	OK	8,07259	10,2069	0,338444	1,72657	0,00275	0,0136364	yes
EIF2AK1	EIF2AK1	EIF2AK1	chr7:6048881 shneg	shCHD1	OK	42,7897	54,0976	0,338302	1,83142	0,0015	0,00814879	yes
ATAD3B	ATAD3B	ATAD3B	chr1:1407163 shneg	shCHD1	OK	8,55255	10,809	0,337802	1,62034	0,00505	0,0226106	yes
TNKS	TNKS	TNKS	chr8:9413444 shneg	shCHD1	OK	4,68572	5,91853	0,336967	1,77699	0,0017	0,00909693	yes
PIR	PIR	PIR	chrX:1536371 shneg	shCHD1	OK	20,3257	25,67	0,336777	1,66858	0,00365	0,0172576	yes
CASP2	CASP2	CASP2	chr7:1429853 shneg	shCHD1	OK	17,2566	21,7921	0,33666	1,82388	0,0014	0,00768951	yes
DCTN4	DCTN4	DCTN4	chr5:1500883 shneg	shCHD1	OK	19,2749	24,3372	0,336444	1,84367	0,00085	0,00508358	yes
NLN	NLN	NLN	chr5:6501802 shneg	shCHD1	OK	9,83832	12,4221	0,336423	1,8553	0,00125	0,00702633	yes
C17orf96	C17orf96	C17orf96	chr17:368279 shneg	shCHD1	OK	30,9841	39,0954	0,335468	1,85944	0,0014	0,00768951	yes
ARL4A	ARL4A	ARL4A	chr7:1272645 shneg	shCHD1	OK	12,9444	16,3314	0,335321	1,72285	0,0027	0,0134267	yes
CCT6A	CCT6A	CCT6A	chr7:5611937 shneg	shCHD1	OK	110,695	139,659	0,33532	1,96301	0,0004	0,00266202	yes
PRTFDC1	PRTFDC1	PRTFDC1	chr10:251375 shneg	shCHD1	OK	7,80748	9,84997	0,335262	1,59803	0,005	0,0224661	yes
NUP35	NUP35	NUP35	chr2:1839890 shneg	shCHD1	OK	8,0206	10,1184	0,335204	1,61868	0,0046	0,0208912	yes
LRRC20	LRRC20	LRRC20	chr10:720587 shneg	shCHD1	OK	6,33677	7,99253	0,334904	1,57648	0,00615	0,0265788	yes
DAXX	DAXX	DAXX	chr6_qbl_hap shneg	shCHD1	OK	33,2644	41,9539	0,334826	1,82332	0,00155	0,00838119	yes
MAPK8IP1	MAPK8IP1	MAPK8IP1	chr11:459070 shneg	shCHD1	OK	16,3973	20,6703	0,334102	1,77333	0,0023	0,0117566	yes
FANCE	FANCE	FANCE	chr6:3542013 shneg	shCHD1	OK	4,82779	6,08564	0,334046	1,60904	0,0047	0,0212899	yes
DDX41	DDX41	DDX41	chr5:1769385 shneg	shCHD1	OK	36,9979	46,6184	0,333454	1,81869	0,0016	0,00862477	yes
CSNK2B	CSNK2B	CSNK2B	chr6_ssto_ha shneg	shCHD1	OK	87,8908	110,716	0,333081	1,6767	0,00315	0,015272	yes
RERE	RERE	RERE	chr1:8412463 shneg	shCHD1	OK	17,0444	21,4697	0,333008	1,86579	0,0014	0,00768951	yes
RBPMS2	RBPMS2	RBPMS2	chr15:650320 shneg	shCHD1	OK	6,05064	7,6211	0,33291	1,58237	0,00615	0,0265788	yes
ZNHIT2	ZNHIT2	ZNHIT2	chr11:648838 shneg	shCHD1	OK	10,7262	13,5096	0,332846	1,58001	0,00645	0,0276097	yes
C1orf198	C1orf198	C1orf198	chr1:2309728 shneg	shCHD1	OK	7,1073	8,94906	0,332435	1,67121	0,00325	0,0156858	yes
HIGD1A	HIGD1A	HIGD1A	chr3:4282439 shneg	shCHD1	OK	17,4597	21,9827	0,332339	1,75754	0,0019	0,00996052	yes
DNAJC18	DNAJC18	DNAJC18	chr5:1387458 shneg	shCHD1	OK	4,07399	5,12904	0,332247	1,6102	0,0063	0,0271428	yes
CCDC51	CCDC51	CCDC51	chr3:4847357 shneg	shCHD1	OK	8,50253	10,7002	0,331668	1,58647	0,00475	0,0214814	yes

NTAN1	NTAN1	NTAN1	chr16:151317 shneg	shCHD1	OK	24,5087	30,8333	0,331198	1,62862	0,0047	0,0212899	yes
C9orf25	C9orf25	C9orf25	chr9:3439818 shneg	shCHD1	OK	3,55912	4,47693	0,330991	1,55606	0,00775	0,03233	yes
HDGF	HDGF	HDGF	chr1:1567118 shneg	shCHD1	OK	221,217	278,257	0,330952	1,94553	0,001	0,0058183	yes
RBL1	RBL1	RBL1	chr20:356261 shneg	shCHD1	OK	2,84091	3,57266	0,330642	1,5513	0,00695	0,0293888	yes
RNF182	RNF182	RNF182	chr6:1392467 shneg	shCHD1	OK	33,3698	41,9587	0,330424	1,83859	0,0014	0,00768951	yes
NANP	NANP	NANP	chr20:255935 shneg	shCHD1	OK	2,51702	3,16481	0,330401	1,48371	0,0107	0,0420661	yes
FAM126A	FAM126A	FAM126A	chr7:2298087 shneg	shCHD1	OK	4,8686	6,12146	0,330369	1,665	0,0042	0,0193522	yes
IPO9	IPO9	IPO9	chr1:2017982 shneg	shCHD1	OK	15,5046	19,483	0,329521	1,8897	0,0007	0,00428773	yes
EIF2C2	EIF2C2	EIF2C2	chr8:1415412 shneg	shCHD1	OK	11,4083	14,3268	0,328628	1,71608	0,0027	0,0134267	yes
BTBD3	BTBD3	BTBD3	chr20:118714 shneg	shCHD1	OK	5,32527	6,68553	0,328186	1,62329	0,0052	0,0231261	yes
HSPA8	HSPA8	HSPA8	chr11:122928 shneg	shCHD1	OK	699,15	877,665	0,328069	1,97273	0,00065	0,0040329	yes
INCENP	INCENP	INCENP	chr11:618914 shneg	shCHD1	OK	18,5108	23,2364	0,328021	1,7897	0,002	0,0104181	yes
TCEB3	TCEB3	TCEB3	chr1:2406985 shneg	shCHD1	OK	10,2185	12,8261	0,327903	1,61579	0,00515	0,0229918	yes
CABLES2	CABLES2	CABLES2	chr20:609636 shneg	shCHD1	OK	5,56608	6,98525	0,327649	1,58802	0,00585	0,0255919	yes
DDX1	DDX1	DDX1	chr2:1573176 shneg	shCHD1	OK	38,1731	47,8998	0,327464	1,81938	0,0018	0,00951868	yes
TSPAN17	TSPAN17	TSPAN17	chr5:1760743 shneg	shCHD1	OK	32,477	40,7483	0,32732	1,79125	0,00225	0,0115391	yes
NEDD9	NEDD9	NEDD9	chr6:1118353 shneg	shCHD1	OK	9,8452	12,3508	0,327109	1,63807	0,0044	0,0201471	yes
NPIPL3	NPIPL3	NPIPL3	chr16:214134 shneg	shCHD1	OK	5,16341	6,4768	0,326958	1,53135	0,0087	0,0355373	yes
SNRPG	SNRPG	SNRPG	chr2:7050850 shneg	shCHD1	OK	119,976	150,45	0,326544	1,72349	0,00255	0,0127904	yes
PRKCD	PRKCD	PRKCD	chr3:5319522 shneg	shCHD1	OK	7,66441	9,60922	0,326245	1,57181	0,00585	0,0255919	yes
C5orf34	C5orf34	C5orf34	chr5:4348680 shneg	shCHD1	OK	4,0067	5,02192	0,325823	1,47858	0,00995	0,0396775	yes
PCSK6	PCSK6	PCSK6	chr15:101844 shneg	shCHD1	OK	3,85799	4,83484	0,32562	1,43567	0,013	0,0492861	yes
ITGB3BP	ITGB3BP	ITGB3BP	chr1:6390644 shneg	shCHD1	OK	14,3789	18,0144	0,325197	1,53402	0,0075	0,0313999	yes
MRVI1	MRVI1	MRVI1	chr11:105627 shneg	shCHD1	OK	32,3102	40,4788	0,325179	1,86566	0,0011	0,0063157	yes
LSM6	LSM6	LSM6	chr4:1470968 shneg	shCHD1	OK	17,6686	22,1308	0,324868	1,52	0,00805	0,0332923	yes
RPL23	RPL23	RPL23	chr17:370063 shneg	shCHD1	OK	2410,71	3018,58	0,324411	1,95554	0,00075	0,00455593	yes
UBE2G1	UBE2G1	UBE2G1	chr17:417251 shneg	shCHD1	OK	13,6015	17,0306	0,324366	1,73846	0,00285	0,0140225	yes
SERPINH1	SERPINH1	SERPINH1	chr11:752731 shneg	shCHD1	OK	112,998	141,432	0,323811	1,8831	0,00075	0,00455593	yes
ATP11A	ATP11A	ATP11A	chr13:113344 shneg	shCHD1	OK	8,14643	10,1956	0,323709	1,76138	0,00285	0,0140225	yes
GPAA1	GPAA1	GPAA1	chr8:1451375 shneg	shCHD1	OK	53,4116	66,8419	0,3236	1,79993	0,0018	0,00951868	yes
RBM22	RBM22	RBM22	chr5:1500703 shneg	shCHD1	OK	23,4634	29,361	0,323489	1,72231	0,00335	0,0160296	yes
TSIX	TSIX	TSIX	chrX:7301203 shneg	shCHD1	OK	2,53177	3,16775	0,323311	1,79007	0,002	0,0104181	yes
AEN	AEN	AEN	chr15:891645 shneg	shCHD1	OK	9,83016	12,2994	0,323301	1,63917	0,00455	0,0207047	yes
CKAP2	CKAP2	CKAP2	chr13:530294 shneg	shCHD1	OK	28,3125	35,4144	0,322899	1,78996	0,00215	0,0110834	yes
U2AF1	U2AF1	U2AF1	chr21:445130 shneg	shCHD1	OK	76,0162	95,0754	0,322765	1,73048	0,00275	0,0136364	yes
SPATA5L1	SPATA5L1	SPATA5L1	chr15:456945 shneg	shCHD1	OK	4,54495	5,68318	0,322433	1,52677	0,0079	0,0327888	yes
WHSC1L1	WHSC1L1	WHSC1L1	chr8:3813256 shneg	shCHD1	OK	14,909	18,6408	0,322281	1,70412	0,00315	0,015272	yes
EHD4	EHD4	EHD4	chr15:421916 shneg	shCHD1	OK	11,7435	14,6824	0,322223	1,64969	0,00495	0,022299	yes
LRRC49	LRRC49	LRRC49	chr15:711849 shneg	shCHD1	OK	4,78348	5,97989	0,322057	1,52784	0,00725	0,0304815	yes
RINT1	RINT1	RINT1	chr7:1051725 shneg	shCHD1	OK	11,8618	14,818	0,321032	1,61343	0,0052	0,0231261	yes
PLEKHJ1	PLEKHJ1	PLEKHJ1	chr19:223315 shneg	shCHD1	OK	28,1544	35,1654	0,320795	1,61966	0,0055	0,0242742	yes
NAV3	NAV3	NAV3	chr12:782250 shneg	shCHD1	OK	3,03398	3,78908	0,320635	1,62716	0,00455	0,0207047	yes
C2orf72	C2orf72	C2orf72	chr2:2319022 shneg	shCHD1	OK	6,57794	8,21428	0,320498	1,57616	0,00605	0,0262198	yes
MAN2C1	MAN2C1	MAN2C1	chr15:756481 shneg	shCHD1	OK	15,6544	19,5472	0,320392	1,68051	0,0034	0,0162243	yes
TFDP1	TFDP1	TFDP1	chr13:114239 shneg	shCHD1	OK	60,3079	75,277	0,319863	1,8094	0,00145	0,00793286	yes
METTL2B	METTL2B	METTL2B	chr7:1281167 shneg	shCHD1	OK	6,73203	8,4009	0,319502	1,54182	0,00665	0,0283439	yes
TIMM22	TIMM22	TIMM22	chr17:900356 shneg	shCHD1	OK	9,03867	11,2788	0,319429	1,51873	0,00785	0,0326299	yes
POLR3D	POLR3D	POLR3D	chr8:2210261 shneg	shCHD1	OK	5,91108	7,37462	0,319146	1,48825	0,00975	0,0391039	yes
PALM	PALM	PALM	chr19:708952 shneg	shCHD1	OK	14,4776	18,0594	0,318928	1,6646	0,00375	0,0176465	yes
PDCD7	PDCD7	PDCD7	chr15:654097 shneg	shCHD1	OK	11,2527	14,0363	0,318883	1,62138	0,0053	0,0234659	yes
TTC19	TTC19	TTC19	chr17:158798 shneg	shCHD1	OK	14,8641	18,5343	0,318372	1,59209	0,0062	0,0267699	yes
PROSER1	PROSER1	PROSER1	chr13:395840 shneg	shCHD1	OK	9,77738	12,1896	0,318127	1,69354	0,0032	0,015482	yes
XBP1	XBP1	XBP1	chr22:291905 shneg	shCHD1	OK	35,0787	43,7067	0,317257	1,70916	0,0029	0,0142283	yes
BAI3	BAI3	BAI3	chr6:6934563 shneg	shCHD1	OK	5,81096	7,23764	0,316744	1,61913	0,00525	0,0232593	yes
KIAA0020	KIAA0020	KIAA0020	chr9:2804154 shneg	shCHD1	OK	10,7032	13,3278	0,3164	1,54449	0,00935	0,0377496	yes
SOCS7	SOCS7	SOCS7	chr17:365080 shneg	shCHD1	OK	7,92424	9,86619	0,31622	1,70868	0,00335	0,0160296	yes
RHOU	RHOU	RHOU	chr1:2287803 shneg	shCHD1	OK	4,59102	5,71573	0,316122	1,45496	0,0113	0,044015	yes
PFDN1	PFDN1	PFDN1	chr5:1396246 shneg	shCHD1	OK	37,3541	46,5043	0,316099	1,66139	0,0039	0,018217	yes
DEGS1	DEGS1	DEGS1	chr1:2243709 shneg	shCHD1	OK	34,598	43,0661	0,315864	1,7099	0,0033	0,0158557	yes
LMNB1	LMNB1	LMNB1	chr5:1261123 shneg	shCHD1	OK	185,308	230,661	0,31585	1,69611	0,0029	0,0142283	yes
SLC9A3R1	SLC9A3R1	SLC9A3R1	chr17:727447 shneg	shCHD1	OK	20,0918	25,0073	0,315741	1,63694	0,0045	0,0205241	yes
BCL3	BCL3	BCL3	chr19:452519 shneg	shCHD1	OK	5,82486	7,24772	0,315304	1,44339	0,01205	0,0464557	yes
ZFHX3	ZFHX3	ZFHX3	chr16:728167 shneg	shCHD1	OK	3,38063	4,20539	0,314946	1,6536	0,0043	0,0197347	yes
RAC1	RAC1	RAC1	chr7:6414125 shneg	shCHD1	OK	159,253	198,098	0,314897	1,85435	0,00135	0,00748275	yes
SORBS1	SORBS1	SORBS1	chr10:970715 shneg	shCHD1	OK	23,8093	29,6129	0,314702	1,77779	0,0016	0,00862477	yes
MCMBP	MCMBP	MCMBP	chr10:121588 shneg	shCHD1	OK	14,8929	18,5228	0,314679	1,6955	0,00295	0,0144329	yes
TRIP10	TRIP10	TRIP10	chr19:673970 shneg	shCHD1	OK	24,5391	30,5172	0,31454	1,65811	0,005	0,0224661	yes
BRD8	BRD8	BRD8	chr5:1374754 shneg	shCHD1	OK	38,5236	47,9029	0,314372	1,768	0,00235	0,0119595	yes
SRXN1	SRXN1	SRXN1	chr20:627267 shneg	shCHD1	OK	6,57477	8,17536	0,314341	1,48692	0,0108	0,0423994	yes
GNG11	GNG11	GNG11	chr7:9355101 shneg	shCHD1	OK	554,263	689,184	0,314319	1,85835	0,00105	0,00607369	yes
UBE2E2	UBE2E2	UBE2E2	chr3:2324478 shneg	shCHD1	OK	17,7107	22,0177	0,314043	1,58424	0,0056	0,0246843	yes
MYH9	MYH9	MYH9	chr22:366773 shneg	shCHD1	OK	127,476	158,435	0,313666	1,89013	0,001	0,0058183	yes
GTF3C2	GTF3C2	GTF3C2	chr2:2754872 shneg	shCHD1	OK	18,2866	22,7256	0,31353	1,64327	0,0038	0,0178336	yes
ZNRF1	ZNRF1	ZNRF1	chr16:750329 shneg	shCHD1	OK	3,65895	4,54673	0,313397	1,48803	0,0097	0,0389594	yes
NHP2L1	NHP2L1	NHP2L1	chr22:420699 shneg	shCHD1	OK	45,9901	57,1468	0,313348	1,69063	0,0032	0,015482	yes
ERH	ERH	ERH	chr14:698468 shneg	shCHD1	OK	139,877	173,8	0,313271	1,72691	0,002	0,0104181	yes
AURKA	AURKA	AURKA	chr20:549444 shneg	shCHD1	OK	24,0232	29,8473	0,313172	1,6437	0,0039	0,018217	yes
CHMP7	CHMP7	CHMP7	chr8:2310114 shneg	shCHD1	OK	9,54186	11,8533	0,312944	1,59568	0,00565	0,0248732	yes
KIF2A	KIF2A	KIF2A	chr5:6160198 shneg	shCHD1	OK	12,4083	15,4139	0,312928	1,64645	0,0034	0,0162243	yes
DIMT1	DIMT1	DIMT1	chr5:6168435 shneg	shCHD1	OK	17,6672	21,9436	0,312727	1,54974	0,00605	0,0262198	yes
PDRG1	PDRG1	PDRG1	chr20:305327 shneg	shCHD1	OK	16,2329	20,1609	0,312634	1,50446	0,00935	0,0377496	yes
C6orf174	C6orf174	C6orf174	chr6:1277595 shneg	shCHD1	OK	9,24333	11,4799	0,312621	1,74063	0,0023	0,0117566	yes
PAPD7	PAPD7	PAPD7	chr5:6714717 shneg	shCHD1	OK	16,3462	20,3002	0,312535	1,66129	0,0041	0,0189794	yes
CFL1	CFL1	CFL1	chr11:656222 shneg	shCHD1	OK	511,613	635,179	0,312112	1,86176	0,00125	0,00702633	yes
ACVR2B	ACVR2B	ACVR2B	chr3:3849251 shneg	shCHD1	OK	2,8147	3,49432	0,312029	1,5666	0,0058	0,0254211	yes
GPX8	GPX8	GPX8	chr5:5440879 shneg	shCHD1	OK	12,8049	15,8953	0,311908	1,63872	0,00345	0,0164347	yes
CTSL1	CTSL1	CTSL1	chr9:9034097 shneg	shCHD1	OK	89,3065	110,842	0,311663	1,75581	0,0024	0,0121606	yes
NUFIP1	NUFIP1	NUFIP1	chr13:455133 shneg	shCHD1	OK	3,36207	4,1719	0,311358	1,48431	0,00855	0,0350893	yes
TUB	TUB	TUB	chr11:806017 shneg	shCHD1	OK	3,69692	4,58704	0,311239	1,52146	0,00775	0,03233	yes
SRP9	SRP9	SRP9	chr1:2259655 shneg	shCHD1	OK	117,958	146,303	0,310684	1,76368	0,0023	0,0117566	yes
HMGB2	HMGB2	HMGB2	chr4:1742525 shneg	shCHD1	OK	163,145	202,341	0,310636	1,79179	0,0021	0,0108578	yes
C20orf112	C20orf112	C20orf112	chr20:310308 shneg	shCHD1	OK	1,94845	2,41653	0,310611	1,50425	0,00905	0,0367622	yes
SLC37A4	SLC37A4	SLC37A4	chr11:118895 shneg	shCHD1	OK	19,7453	24,4829	0,310268	1,59472	0,0053	0,0234659	yes

NRGN	NRGN	NRGN	chr11:124609	shneg	shCHD1	OK	11,5782	14,3546	0,310098	1,47388	0,00985	0,0393915	yes
CCDC86	CCDC86	CCDC86	chr11:606094	shneg	shCHD1	OK	15,6392	19,3887	0,310049	1,54149	0,00785	0,0326299	yes
FAM20C	FAM20C	FAM20C	chr7:192968-:	shneg	shCHD1	OK	11,8642	14,7067	0,309854	1,57705	0,00575	0,0252576	yes
YES1	YES1	YES1	chr18:721591	shneg	shCHD1	OK	11,5845	14,3591	0,309766	1,64903	0,004	0,018603	yes
VARS	VARS	VARS	chr6_ssto_ha	shneg	shCHD1	OK	20,2824	25,135	0,30947	1,70315	0,00375	0,0176465	yes
ABCF1	ABCF1	ABCF1	chr6_ssto_ha	shneg	shCHD1	OK	34,7649	43,0822	0,309459	1,73006	0,00215	0,0110834	yes
PTGES3	PTGES3	PTGES3	chr12:570571	shneg	shCHD1	OK	125,206	155,13	0,309171	1,78584	0,00215	0,0110834	yes
LOC647979	LOC647979	LOC647979	chr20:346335	shneg	shCHD1	OK	46,0008	56,9938	0,309148	1,79147	0,00205	0,0106507	yes
ASF1B	ASF1B	ASF1B	chr19:142303	shneg	shCHD1	OK	33,8154	41,8857	0,308776	1,64311	0,00445	0,020336	yes
ZBTB9	ZBTB9	ZBTB9	chr6_ssto_ha	shneg	shCHD1	OK	7,55778	9,36118	0,308728	1,48334	0,00975	0,0391039	yes
CKS2	CKS2	CKS2	chr9:9192611	shneg	shCHD1	OK	192,769	238,764	0,308715	1,69226	0,00255	0,0127904	yes
NECAB3	NECAB3	NECAB3	chr20:322448	shneg	shCHD1	OK	20,717	25,6514	0,308221	1,50985	0,0087	0,0355373	yes
SNX11	SNX11	SNX11	chr17:461849	shneg	shCHD1	OK	7,43843	9,20764	0,307834	1,45145	0,0115	0,0446442	yes
MCAT	MCAT	MCAT	chr22:435282	shneg	shCHD1	OK	9,66037	11,9534	0,307273	1,46143	0,01145	0,0444873	yes
ZNF143	ZNF143	ZNF143	chr11:948251	shneg	shCHD1	OK	5,03222	6,22484	0,306843	1,48632	0,00855	0,0350893	yes
SEPHS1	SEPHS1	SEPHS1	chr10:133594	shneg	shCHD1	OK	28,6406	35,4266	0,306774	1,70418	0,0033	0,0158557	yes
FAM104B	FAM104B	FAM104B	chrX:5516953	shneg	shCHD1	OK	12,4883	15,4373	0,305837	1,42349	0,01135	0,0441727	yes
PROSC	PROSC	PROSC	chr8:3762010	shneg	shCHD1	OK	19,5712	24,1912	0,30575	1,61839	0,00555	0,0244872	yes
COL6A1	COL6A1	COL6A1	chr21:474016	shneg	shCHD1	OK	82,1841	101,545	0,305184	1,79114	0,0024	0,0121606	yes
KDELR2	KDELR2	KDELR2	chr7:6500711	shneg	shCHD1	OK	76,9392	95,0535	0,305021	1,76024	0,00235	0,0119595	yes
CYS1	CYS1	CYS1	chr2:1019692	shneg	shCHD1	OK	9,97613	12,3244	0,304968	1,51732	0,00785	0,0326299	yes
SQSTM1	SQSTM1	SQSTM1	chr5:1792245	shneg	shCHD1	OK	231,959	286,546	0,304901	1,46477	0,0103	0,0407813	yes
ARHGAP18	ARHGAP18	ARHGAP18	chr6:1298982	shneg	shCHD1	OK	41,2159	50,9142	0,304865	1,72626	0,00235	0,0119595	yes
POMP	POMP	POMP	chr13:292331	shneg	shCHD1	OK	47,5676	58,7584	0,304816	1,64423	0,0049	0,0220881	yes
HPCAL1	HPCAL1	HPCAL1	chr2:1044303	shneg	shCHD1	OK	10,5572	13,0387	0,304569	1,44807	0,0104	0,0410955	yes
MNF1	MNF1	MNF1	chr6:3366534	shneg	shCHD1	OK	84,8847	104,813	0,304241	1,52334	0,00815	0,033636	yes
MYO19	MYO19	MYO19	chr17:348424	shneg	shCHD1	OK	40,2662	49,7031	0,303768	1,44462	0,01205	0,0464557	yes
NRCAM	NRCAM	NRCAM	chr7:1077880	shneg	shCHD1	OK	50,8762	62,7931	0,303614	1,76392	0,00235	0,0119595	yes
SSH2	SSH2	SSH2	chr17:279529	shneg	shCHD1	OK	5,58613	6,8932	0,303326	1,59045	0,00505	0,0226106	yes
MIDN	MIDN	MIDN	chr19:124855	shneg	shCHD1	OK	9,61462	11,8615	0,302985	1,56061	0,0068	0,028886	yes
CDK17	CDK17	CDK17	chr12:966720	shneg	shCHD1	OK	6,0981	7,52229	0,302812	1,50007	0,0089	0,036248	yes
NUMA1	NUMA1	NUMA1	chr11:717099	shneg	shCHD1	OK	43,5672	53,7388	0,302723	1,54464	0,00795	0,0329472	yes
TDP2	TDP2	TDP2	chr6:2465020	shneg	shCHD1	OK	10,5906	13,062	0,302593	1,44254	0,0123	0,0472104	yes
LRP3	LRP3	LRP3	chr19:336855	shneg	shCHD1	OK	22,2497	27,4408	0,302535	1,64325	0,0038	0,0178336	yes
SLC35A4	SLC35A4	SLC35A4	chr5:1399444	shneg	shCHD1	OK	35,1185	43,3114	0,302516	1,66997	0,00285	0,0140225	yes
MRPL48	MRPL48	MRPL48	chr11:734989	shneg	shCHD1	OK	15,2729	18,8344	0,302398	1,47104	0,01005	0,0399619	yes
PKM2	PKM2	PKM2	chr15:724913	shneg	shCHD1	OK	663,444	818,086	0,302278	1,81089	0,0015	0,00814879	yes
RRS1	RRS1	RRS1	chr8:6734126	shneg	shCHD1	OK	39,1414	48,2626	0,302213	1,62802	0,0046	0,0208912	yes
ACSL1	ACSL1	ACSL1	chr4:1856767	shneg	shCHD1	OK	8,60976	10,6158	0,30217	1,54249	0,0067	0,0285134	yes
KNTC1	KNTC1	KNTC1	chr12:123011	shneg	shCHD1	OK	11,3752	14,0238	0,301986	1,65359	0,0042	0,0193522	yes
ARHGEF16	ARHGEF16	ARHGEF16	chr1:3371146	shneg	shCHD1	OK	7,85852	9,6854	0,301553	1,47149	0,0108	0,0423994	yes
KIAA1429	KIAA1429	KIAA1429	chr8:9550000	shneg	shCHD1	OK	6,53084	8,04902	0,301546	1,44502	0,01175	0,0454375	yes
ANXA6	ANXA6	ANXA6	chr5:1504802	shneg	shCHD1	OK	45,0943	55,5755	0,301504	1,70608	0,004	0,018603	yes
GEMIN5	GEMIN5	GEMIN5	chr5:1542669	shneg	shCHD1	OK	8,24584	10,162	0,301441	1,58636	0,00485	0,0218981	yes
MARS	MARS	MARS	chr12:578817	shneg	shCHD1	OK	51,8185	63,859	0,301422	1,59108	0,00525	0,0232593	yes
ODF2	ODF2	ODF2	chr9:1312174	shneg	shCHD1	OK	33,3864	41,1387	0,301237	1,61183	0,00465	0,0210977	yes
NOLC1	NOLC1	NOLC1	chr10:103911	shneg	shCHD1	OK	32,8029	40,419	0,301211	1,69708	0,0033	0,0158557	yes
CTPS2	CTPS2	CTPS2	chrX:1660612	shneg	shCHD1	OK	9,68264	11,9297	0,301088	1,55384	0,00595	0,0259073	yes
LOC10050703	LOC10050703	LOC10050703	chr3:1296127	shneg	shCHD1	OK	3,48741	4,2966	0,301038	1,44907	0,0112	0,0437111	yes
MARS2	MARS2	MARS2	chr2:1985700	shneg	shCHD1	OK	3,91545	4,82341	0,300875	1,44293	0,0114	0,0443301	yes
RRP12	RRP12	RRP12	chr10:991164	shneg	shCHD1	OK	10,7261	13,2131	0,300839	1,58904	0,0052	0,0231261	yes
SIVA1	SIVA1	SIVA1	chr14:105219	shneg	shCHD1	OK	77,2712	95,1852	0,300806	1,58315	0,00425	0,0195438	yes
IL27RA	IL27RA	IL27RA	chr19:141422	shneg	shCHD1	OK	22,5911	27,825	0,300628	1,63377	0,00445	0,020336	yes
PIP4K2B	PIP4K2B	PIP4K2B	chr17:369219	shneg	shCHD1	OK	39,0312	48,0717	0,300561	1,73792	0,00255	0,0127904	yes
LAMC1	LAMC1	LAMC1	chr1:1829925	shneg	shCHD1	OK	104,649	128,886	0,300539	1,80807	0,0021	0,0108578	yes
SH3BP1	SH3BP1	SH3BP1	chr22:380356	shneg	shCHD1	OK	10,1649	12,5153	0,300102	1,48823	0,0099	0,0395347	yes
RUNDC3A	RUNDC3A	RUNDC3A	chr17:423859	shneg	shCHD1	OK	35,3392	43,4952	0,299585	1,62208	0,0043	0,0197347	yes
TSTA3	TSTA3	TSTA3	chr8:1446947	shneg	shCHD1	OK	44,1487	54,3288	0,299346	1,59198	0,00585	0,0255919	yes
MRPS17	MRPS17	MRPS17	chr7:5601961	shneg	shCHD1	OK	40,1551	49,4117	0,299271	1,43539	0,01255	0,0479717	yes
NUCKS1	NUCKS1	NUCKS1	chr1:2056819	shneg	shCHD1	OK	71,8549	88,4077	0,299086	1,77314	0,00125	0,00702633	yes
RPP25	RPP25	RPP25	chr15:752474	shneg	shCHD1	OK	16,7329	20,5847	0,298891	1,54777	0,0067	0,0285134	yes
RBBP7	RBBP7	RBBP7	chrX:1686277	shneg	shCHD1	OK	81,1079	99,7756	0,298843	1,70681	0,0029	0,0142283	yes
PGAM1	PGAM1	PGAM1	chr10:991860	shneg	shCHD1	OK	125,461	154,31	0,2986	1,71643	0,0023	0,0117566	yes
ARHGEF3	ARHGEF3	ARHGEF3	chr3:5676144	shneg	shCHD1	OK	3,58974	4,41445	0,298354	1,43362	0,0107	0,0420661	yes
MRPL32	MRPL32	MRPL32	chr7:4297193	shneg	shCHD1	OK	38,3386	47,1226	0,29762	1,49318	0,00955	0,0384457	yes
PRMT1	PRMT1	PRMT1	chr19:501804	shneg	shCHD1	OK	106,594	130,974	0,297144	1,6661	0,00395	0,0184012	yes
C16orf59	C16orf59	C16orf59	chr16:251011	shneg	shCHD1	OK	8,45771	10,3903	0,296894	1,41445	0,01215	0,0467765	yes
DNAJC9	DNAJC9	DNAJC9	chr10:750025	shneg	shCHD1	OK	33,8711	41,6024	0,296611	1,55737	0,007	0,0295643	yes
DCLRE1B	DCLRE1B	DCLRE1B	chr1:1144479	shneg	shCHD1	OK	3,52138	4,32457	0,296416	1,41015	0,0127	0,0484255	yes
RNF216	RNF216	RNF216	chr7:5659671	shneg	shCHD1	OK	12,482	15,3279	0,29631	1,61354	0,0051	0,0228051	yes
ZC3H7B	ZC3H7B	ZC3H7B	chr22:416975	shneg	shCHD1	OK	29,4365	36,1464	0,296246	1,69367	0,0034	0,0162243	yes
PSMD7	PSMD7	PSMD7	chr16:743306	shneg	shCHD1	OK	56,1269	68,9033	0,295879	1,62868	0,0054	0,0238631	yes
CRK	CRK	CRK	chr17:132464	shneg	shCHD1	OK	22,3827	27,4755	0,295767	1,59989	0,00505	0,0226106	yes
RAD21	RAD21	RAD21	chr8:1178581	shneg	shCHD1	OK	68,4507	84,0203	0,295673	1,70982	0,0033	0,0158557	yes
SF3A1	SF3A1	SF3A1	chr22:307279	shneg	shCHD1	OK	21,3795	26,2423	0,295664	1,56496	0,00605	0,0262198	yes
PPP2CA	PPP2CA	PPP2CA	chr5:1335321	shneg	shCHD1	OK	55,9706	68,6986	0,295611	1,64959	0,00365	0,0172576	yes
CALM3	CALM3	CALM3	chr19:471045	shneg	shCHD1	OK	189,626	232,745	0,295595	1,74674	0,00235	0,0119595	yes
SNHG1	SNHG1	SNHG1	chr11:626194	shneg	shCHD1	OK	49,0607	60,2134	0,295515	1,52263	0,008	0,0331249	yes
SWAP70	SWAP70	SWAP70	chr11:968562	shneg	shCHD1	OK	26,2181	32,1729	0,295285	1,66293	0,00385	0,0180379	yes
FADD	FADD	FADD	chr11:700492	shneg	shCHD1	OK	14,4809	17,7689	0,295207	1,46304	0,0111	0,0433694	yes
GHDC	GHDC	GHDC	chr17:403411	shneg	shCHD1	OK	14,8071	18,1663	0,294972	1,50813	0,00855	0,0350893	yes
CSDA	CSDA	CSDA	chr12:108516	shneg	shCHD1	OK	177,481	217,741	0,294951	1,72295	0,00255	0,0127904	yes
EHMT2	EHMT2	EHMT2	chr6_ssto_ha	shneg	shCHD1	OK	25,4429	31,2099	0,294741	1,61745	0,00425	0,0195438	yes
PARVB	PARVB	PARVB	chr22:443950	shneg	shCHD1	OK	23,9282	29,3516	0,294728	1,51299	0,00895	0,036409	yes
NUP50	NUP50	NUP50	chr22:455597	shneg	shCHD1	OK	15,3485	18,8246	0,294521	1,60974	0,00475	0,0214814	yes
DENND1C	DENND1C	DENND1C	chr19:646425	shneg	shCHD1	OK	9,14765	11,2156	0,294036	1,44563	0,0111	0,0433694	yes
CCT2	CCT2	CCT2	chr12:699792	shneg	shCHD1	OK	123,159	151,001	0,29403	1,69783	0,0035	0,0166331	yes
C7orf50	C7orf50	C7orf50	chr7:1036622	shneg	shCHD1	OK	74,5166	91,3614	0,294023	1,56381	0,0072	0,0302987	yes
TUBGCP3	TUBGCP3	TUBGCP3	chr13:113139	shneg	shCHD1	OK	11,897	14,5842	0,293803	1,54752	0,0069	0,0292395	yes
ALG8	ALG8	ALG8	chr11:778119	shneg	shCHD1	OK	19,5593	23,9739	0,293611	1,47415	0,0121	0,0466356	yes
SH3RF1	SH3RF1	SH3RF1	chr4:1700154	shneg	shCHD1	OK	4,72335	5,78919	0,293551	1,45358	0,0116	0,0449948	yes
MAP2K4	MAP2K4	MAP2K4	chr17:119241	shneg	shCHD1	OK	5,6674	6,94522	0,293333	1,41982	0,013	0,0492861	yes
Sep 07	Sep 07	Sep 07	chr7:3584059	shneg	shCHD1	OK	36,1944	44,3512	0,293208	1,67153	0,0026	0,0129897	yes

FARSB	FARSB	FARSB	chr2:2234361	shneg	shCHD1	OK	15,0254	18,4101	0,293096	1,49201	0,00995	0,0396775	yes
ARGLU1	ARGLU1	ARGLU1	chr13:107195	shneg	shCHD1	OK	73,8865	90,5271	0,29304	1,64504	0,0043	0,0197347	yes
PDLIM7	PDLIM7	PDLIM7	chr5:1769103	shneg	shCHD1	OK	48,6173	59,5614	0,292909	1,46858	0,0105	0,0414554	yes
EBNA1BP2	EBNA1BP2	EBNA1BP2	chr1:4362984	shneg	shCHD1	OK	37,3441	45,746	0,292764	1,51331	0,00905	0,0367622	yes
GTPBP4	GTPBP4	GTPBP4	chr10:103434	shneg	shCHD1	OK	28,5086	34,9197	0,292644	1,58429	0,00665	0,0283439	yes
CHAMP1	CHAMP1	CHAMP1	chr13:115079	shneg	shCHD1	OK	11,4676	14,0451	0,292507	1,52252	0,00815	0,033636	yes
MTSS1L	MTSS1L	MTSS1L	chr16:706951	shneg	shCHD1	OK	5,78365	7,08347	0,292476	1,46984	0,0102	0,0404429	yes
TM9SF3	TM9SF3	TM9SF3	chr10:982778	shneg	shCHD1	OK	35,7042	43,7181	0,292137	1,68524	0,00295	0,0144329	yes
C14orf43	C14orf43	C14orf43	chr14:741818	shneg	shCHD1	OK	4,53317	5,54948	0,291832	1,49954	0,00945	0,0380871	yes
CHEK1	CHEK1	CHEK1	chr11:125495	shneg	shCHD1	OK	10,9526	13,4045	0,291447	1,43883	0,01295	0,0491768	yes
ARHGAP31	ARHGAP31	ARHGAP31	chr3:1190132	shneg	shCHD1	OK	4,82543	5,90558	0,29142	1,51796	0,0078	0,0324802	yes
KCTD3	KCTD3	KCTD3	chr1:2157407	shneg	shCHD1	OK	10,8018	13,2189	0,291334	1,52158	0,0084	0,0345449	yes
METTL17	METTL17	METTL17	chr14:214579	shneg	shCHD1	OK	21,6214	26,449	0,290755	1,4932	0,0099	0,0395347	yes
PSMC1	PSMC1	PSMC1	chr14:907228	shneg	shCHD1	OK	56,5278	69,1415	0,290593	1,59505	0,0058	0,0254211	yes
BCHE	BCHE	BCHE	chr3:1654906	shneg	shCHD1	OK	72,8909	89,1516	0,290523	1,65683	0,0037	0,0174466	yes
ASH2L	ASH2L	ASH2L	chr8:3796301	shneg	shCHD1	OK	14,1652	17,3228	0,290325	1,49163	0,0112	0,0437111	yes
MAML1	MAML1	MAML1	chr5:1791598	shneg	shCHD1	OK	19,4815	23,8187	0,289986	1,6222	0,00455	0,0207047	yes
IDH3B	IDH3B	IDH3B	chr20:263904	shneg	shCHD1	OK	22,1002	27,02	0,289971	1,43125	0,01285	0,0488904	yes
PIK3C2B	PIK3C2B	PIK3C2B	chr1:2043917	shneg	shCHD1	OK	10,7335	13,1222	0,289895	1,59268	0,0052	0,0231261	yes
CIRH1A	CIRH1A	CIRH1A	chr16:691664	shneg	shCHD1	OK	26,4016	32,2636	0,289281	1,54793	0,0083	0,0341639	yes
PRC1	PRC1	PRC1	chr15:915092	shneg	shCHD1	OK	45,4107	55,4813	0,288971	1,62368	0,00505	0,0226106	yes
PPRC1	PPRC1	PPRC1	chr10:103892	shneg	shCHD1	OK	14,7505	18,0175	0,288639	1,57962	0,0065	0,0277896	yes
SRSF7	SRSF7	SRSF7	chr2:3897074	shneg	shCHD1	OK	46,8438	57,2018	0,288202	1,617	0,00515	0,0229918	yes
ERRFI1	ERRFI1	ERRFI1	chr1:8071778	shneg	shCHD1	OK	93,1713	113,718	0,287505	1,67619	0,00335	0,0160296	yes
PRRC1	PRRC1	PRRC1	chr5:1268533	shneg	shCHD1	OK	24,1485	29,4724	0,287433	1,6062	0,0044	0,0201471	yes
ANP32A	ANP32A	ANP32A	chr15:690708	shneg	shCHD1	OK	40,9177	49,9344	0,28731	1,56269	0,00725	0,0304815	yes
CEBPG	CEBPG	CEBPG	chr19:338645	shneg	shCHD1	OK	10,6199	12,9595	0,287238	1,49434	0,0087	0,0355373	yes
GPR172A	GPR172A	GPR172A	chr8:1455822	shneg	shCHD1	OK	41,0255	50,063	0,287224	1,55746	0,00695	0,0293888	yes
DDX52	DDX52	DDX52	chr17:359723	shneg	shCHD1	OK	20,5468	25,0687	0,286976	1,56794	0,00605	0,0262198	yes
ATP1A3	ATP1A3	ATP1A3	chr19:424707	shneg	shCHD1	OK	10,7822	13,1542	0,286879	1,48081	0,00915	0,0370927	yes
WDR46	WDR46	WDR46	chr6_qbl_hap	shneg	shCHD1	OK	22,2758	27,1738	0,28674	1,52233	0,0075	0,0313999	yes
SMAD5	SMAD5	SMAD5	chr5:1354652	shneg	shCHD1	OK	11,4138	13,9225	0,286639	1,55456	0,0063	0,0271428	yes
TMEM106C	TMEM106C	TMEM106C	chr12:483573	shneg	shCHD1	OK	74,6836	91,0912	0,286519	1,58879	0,00625	0,0269607	yes
PABPC1	PABPC1	PABPC1	chr8:1017151	shneg	shCHD1	OK	191,889	233,972	0,286068	1,70458	0,0026	0,0129897	yes
TERF1	TERF1	TERF1	chr8:7392109	shneg	shCHD1	OK	11,9991	14,6276	0,285771	1,46385	0,0126	0,0481232	yes
WDR54	WDR54	WDR54	chr2:7464888	shneg	shCHD1	OK	40,1562	48,9487	0,285648	1,48276	0,01015	0,040279	yes
RPS6KA1	RPS6KA1	RPS6KA1	chr1:2685624	shneg	shCHD1	OK	10,7541	13,1071	0,285465	1,4611	0,01085	0,0425717	yes
AP1B1	AP1B1	AP1B1	chr22:297236	shneg	shCHD1	OK	19,1065	23,274	0,284658	1,55685	0,0078	0,0324802	yes
MEX3D	MEX3D	MEX3D	chr19:155466	shneg	shCHD1	OK	16,9252	20,6164	0,284613	1,46905	0,01	0,0398199	yes
PPFIA3	PPFIA3	PPFIA3	chr19:496226	shneg	shCHD1	OK	7,3895	8,99639	0,28387	1,45646	0,0123	0,0472104	yes
OPTN	OPTN	OPTN	chr10:131420	shneg	shCHD1	OK	39,9358	48,6157	0,28374	1,6072	0,00525	0,0232593	yes
STK24	STK24	STK24	chr13:991024	shneg	shCHD1	OK	17,6403	21,4645	0,28308	1,55249	0,0058	0,0254211	yes
MED4	MED4	MED4	chr13:486500	shneg	shCHD1	OK	21,0333	25,5852	0,282637	1,47009	0,01095	0,0428555	yes
PSPC1	PSPC1	PSPC1	chr13:202488	shneg	shCHD1	OK	31,8166	38,6857	0,28202	1,48799	0,0094	0,0379295	yes
MLEC	MLEC	MLEC	chr12:121124	shneg	shCHD1	OK	54,7368	66,5392	0,281692	1,65686	0,00445	0,020336	yes
ZIC2	ZIC2	ZIC2	chr13:100634	shneg	shCHD1	OK	34,8033	42,2971	0,281333	1,56085	0,0065	0,0277896	yes
LMO4	LMO4	LMO4	chr1:8779415	shneg	shCHD1	OK	18,6918	22,7072	0,280744	1,56154	0,00625	0,0269607	yes
FTSJ1	FTSJ1	FTSJ1	chrX:4833454	shneg	shCHD1	OK	38,4762	46,7273	0,280299	1,50705	0,00795	0,0329472	yes
TRAF3IP1	TRAF3IP1	TRAF3IP1	chr2:2392291	shneg	shCHD1	OK	9,05965	10,9997	0,279931	1,44677	0,0107	0,0420661	yes
PPM1G	PPM1G	PPM1G	chr2:2760406	shneg	shCHD1	OK	69,9286	84,8677	0,279333	1,5846	0,0064	0,0274548	yes
UBE2S	UBE2S	UBE2S	chr19:559126	shneg	shCHD1	OK	124,227	150,742	0,279106	1,56659	0,0065	0,0277896	yes
MAP1LC3B	MAP1LC3B	MAP1LC3B	chr16:874258	shneg	shCHD1	OK	24,0178	29,1341	0,278606	1,48186	0,01015	0,040279	yes
SPIRE1	SPIRE1	SPIRE1	chr18:124465	shneg	shCHD1	OK	8,11348	9,84116	0,278506	1,44312	0,01245	0,0476418	yes
GNA13	GNA13	GNA13	chr17:630054	shneg	shCHD1	OK	12,0575	14,624	0,278412	1,51965	0,0082	0,0338022	yes
STK39	STK39	STK39	chr2:1688105	shneg	shCHD1	OK	63,8008	77,3751	0,278296	1,6021	0,0045	0,0205241	yes
SRSF4	SRSF4	SRSF4	chr1:2947424	shneg	shCHD1	OK	34,4955	41,8332	0,278239	1,52751	0,00915	0,0370927	yes
MANEAL	MANEAL	MANEAL	chr1:3825977	shneg	shCHD1	OK	25,7461	31,2179	0,278021	1,49196	0,00945	0,0380871	yes
LYPLA2	LYPLA2	LYPLA2	chr1:2411764	shneg	shCHD1	OK	28,9107	35,0544	0,277994	1,45277	0,01145	0,0444873	yes
USP22	USP22	USP22	chr17:209029	shneg	shCHD1	OK	45,3447	54,9654	0,277588	1,60728	0,00595	0,0259073	yes
TRAP1	TRAP1	TRAP1	chr16:370293	shneg	shCHD1	OK	41,5655	50,3796	0,277452	1,45717	0,01095	0,0428555	yes
STIL	STIL	STIL	chr1:4771581	shneg	shCHD1	OK	8,55156	10,3626	0,277122	1,45121	0,01225	0,0470834	yes
TALDO1	TALDO1	TALDO1	chr11:747431	shneg	shCHD1	OK	120,916	146,511	0,277011	1,56266	0,0082	0,0338022	yes
TPP2	TPP2	TPP2	chr13:103249	shneg	shCHD1	OK	17,8798	21,6567	0,276483	1,51809	0,0078	0,0324802	yes
LMNB2	LMNB2	LMNB2	chr19:242816	shneg	shCHD1	OK	64,0335	77,5119	0,275591	1,60719	0,0047	0,0212899	yes
AGPAT6	AGPAT6	AGPAT6	chr8:4143570	shneg	shCHD1	OK	17,6882	21,4039	0,275091	1,53921	0,00715	0,0300974	yes
GNAI1	GNAI1	GNAI1	chr7:7976413	shneg	shCHD1	OK	30,381	36,7564	0,274824	1,52403	0,0071	0,0299412	yes
CSPP1	CSPP1	CSPP1	chr8:6797660	shneg	shCHD1	OK	9,30639	11,2528	0,273985	1,43801	0,0127	0,0484255	yes
NUP43	NUP43	NUP43	chr6:1500454	shneg	shCHD1	OK	15,4798	18,7083	0,273287	1,46567	0,0119	0,045941	yes
YWHAZ	YWHAZ	YWHAZ	chr8:1019308	shneg	shCHD1	OK	157,672	190,544	0,273198	1,61378	0,0052	0,0231261	yes
CLINT1	CLINT1	CLINT1	chr5:1572127	shneg	shCHD1	OK	25,501	30,8154	0,273096	1,52554	0,00665	0,0283439	yes
TNFRSF10D	TNFRSF10D	TNFRSF10D	chr8:2299310	shneg	shCHD1	OK	17,0601	20,611	0,272785	1,46108	0,01175	0,0454375	yes
COX6C	COX6C	COX6C	chr8:1008902	shneg	shCHD1	OK	73,8619	89,2224	0,272574	1,4561	0,0118	0,0456055	yes
DDX39A	DDX39A	DDX39A	chr19:145196	shneg	shCHD1	OK	51,1132	61,6786	0,271072	1,47188	0,0089	0,036248	yes
SLMAP	SLMAP	SLMAP	chr3:5774317	shneg	shCHD1	OK	14,3028	17,2577	0,27094	1,45961	0,0103	0,0407813	yes
SSRP1	SSRP1	SSRP1	chr11:570934	shneg	shCHD1	OK	91,6351	110,537	0,270559	1,57028	0,00695	0,0293888	yes
MCM2	MCM2	MCM2	chr3:1273172	shneg	shCHD1	OK	33,5873	40,4966	0,269886	1,50927	0,0083	0,0341639	yes
GET4	GET4	GET4	chr7:916190-	shneg	shCHD1	OK	25,6721	30,9514	0,269802	1,42805	0,0127	0,0484255	yes
CACYBP	CACYBP	CACYBP	chr1:1749685	shneg	shCHD1	OK	25,7091	30,9824	0,269172	1,47173	0,00995	0,0396775	yes
SCN3A	SCN3A	SCN3A	chr2:1659440	shneg	shCHD1	OK	18,8418	22,7053	0,269086	1,52554	0,00895	0,036409	yes
ESPL1	ESPL1	ESPL1	chr12:536620	shneg	shCHD1	OK	12,674	15,2727	0,269076	1,47949	0,00975	0,0391039	yes
KBTBD2	KBTBD2	KBTBD2	chr7:3290777	shneg	shCHD1	OK	17,8311	21,484	0,268867	1,45202	0,01175	0,0454375	yes
UPF1	UPF1	UPF1	chr19:189427	shneg	shCHD1	OK	22,6834	27,3204	0,268344	1,50506	0,0086	0,0352322	yes
NCALD	NCALD	NCALD	chr8:1026987	shneg	shCHD1	OK	38,0598	45,8196	0,267697	1,51257	0,0093	0,0375913	yes
COL3A1	COL3A1	COL3A1	chr2:1898390	shneg	shCHD1	OK	95,3126	114,725	0,267437	1,58397	0,00635	0,0272991	yes
XRCC6	XRCC6	XRCC6	chr22:420172	shneg	shCHD1	OK	163,918	197,295	0,267379	1,56836	0,00605	0,0262198	yes
NCAPD2	NCAPD2	NCAPD2	chr12:660329	shneg	shCHD1	OK	46,9221	56,4568	0,266879	1,54112	0,007	0,0295643	yes
PSMC3	PSMC3	PSMC3	chr11:474403	shneg	shCHD1	OK	52,4233	63,0482	0,266248	1,4514	0,0113	0,044015	yes
RAP1B	RAP1B	RAP1B	chr12:690046	shneg	shCHD1	OK	88,3278	106,21	0,265979	1,52705	0,0073	0,030664	yes
LRRK1	LRRK1	LRRK1	chr15:101459	shneg	shCHD1	OK	8,51992	10,2411	0,265465	1,43494	0,0119	0,045941	yes
SUPT16H	SUPT16H	SUPT16H	chr14:218196	shneg	shCHD1	OK	48,3007	58,0242	0,264611	1,52848	0,0079	0,0327888	yes
SLC25A13	SLC25A13	SLC25A13	chr7:9574953	shneg	shCHD1	OK	24,0557	28,8954	0,264459	1,4393	0,01175	0,0454375	yes
WIPI2	WIPI2	WIPI2	chr7:5229834	shneg	shCHD1	OK	22,1191	26,5662	0,264302	1,46205	0,01125	0,0438693	yes
NAV1	NAV1	NAV1	chr1:2016174	shneg	shCHD1	OK	12,2068	14,6444	0,262657	1,48465	0,00935	0,0377496	yes

ELAVL1	ELAVL1	ELAVL1	chr19:802345 shneg	shCHD1	OK	15,2817	18,332	0,262562	1,45206	0,01085	0,0425717	yes
STRN	STRN	STRN	chr2:3706484 shneg	shCHD1	OK	4,68682	5,62171	0,262401	1,41892	0,0132	0,0499627	yes
CAD	CAD	CAD	chr2:2744025 shneg	shCHD1	OK	23,7503	28,4676	0,261374	1,49263	0,0089	0,036248	yes
NDST1	NDST1	NDST1	chr5:1498876 shneg	shCHD1	OK	13,3539	16,0061	0,261355	1,45813	0,01155	0,0448258	yes
NCAPD3	NCAPD3	NCAPD3	chr11:134022 shneg	shCHD1	OK	18,6759	22,3751	0,260713	1,4527	0,012	0,0463014	yes
PRDX2	PRDX2	PRDX2	chr19:129076 shneg	shCHD1	OK	147,164	176,29	0,260527	1,45683	0,0107	0,0420661	yes
YWHAH	YWHAH	YWHAH	chr22:323295 shneg	shCHD1	OK	44,8039	53,6676	0,260426	1,41786	0,0128	0,0487667	yes
ENO1	ENO1	ENO1	chr1:8921058 shneg	shCHD1	OK	506,347	606,196	0,259658	1,56413	0,0076	0,0317804	yes
CYBA	CYBA	CYBA	chr16:887096 shneg	shCHD1	OK	327,207	391,646	0,259344	1,47735	0,00975	0,0391039	yes
ATP2A2	ATP2A2	ATP2A2	chr12:110719 shneg	shCHD1	OK	49,8334	59,6384	0,259129	1,42183	0,01285	0,0488904	yes
KDM3B	KDM3B	KDM3B	chr5:1376882 shneg	shCHD1	OK	32,3152	38,6556	0,258464	1,4931	0,0098	0,0392479	yes
MGAT1	MGAT1	MGAT1	chr5:1802175 shneg	shCHD1	OK	36,7775	43,9829	0,25812	1,4187	0,0132	0,0499627	yes
NSD1	NSD1	NSD1	chr5:1765600 shneg	shCHD1	OK	13,2241	15,8107	0,257732	1,47054	0,01015	0,040279	yes
ENDOD1	ENDOD1	ENDOD1	chr11:948229 shneg	shCHD1	OK	21,9878	26,2858	0,257582	1,42941	0,01215	0,0467765	yes
HJURP	HJURP	HJURP	chr2:2347454 shneg	shCHD1	OK	36,4184	43,5327	0,257432	1,43614	0,01135	0,0441727	yes
S100A11	S100A11	S100A11	chr1:1520049 shneg	shCHD1	OK	1071,66	1280,47	0,256823	1,52145	0,00865	0,0353954	yes
CYFIP2	CYFIP2	CYFIP2	chr5:1566930 shneg	shCHD1	OK	16,807	20,0809	0,256761	1,43461	0,01235	0,0473762	yes
PSMD2	PSMD2	PSMD2	chr3:1840170 shneg	shCHD1	OK	85,1982	101,714	0,255626	1,48076	0,00905	0,0367622	yes
MT2A	MT2A	MT2A	chr16:566424 shneg	shCHD1	OK	1212,3	1446,88	0,255201	1,47707	0,00895	0,036409	yes
CDK12	CDK12	CDK12	chr17:376177 shneg	shCHD1	OK	15,4948	18,4834	0,254444	1,43498	0,01305	0,0494622	yes
ANXA11	ANXA11	ANXA11	chr10:819148 shneg	shCHD1	OK	62,9976	75,1326	0,254143	1,43322	0,0128	0,0487667	yes
CANX	CANX	CANX	chr5:1791259 shneg	shCHD1	OK	168,757	201,255	0,254073	1,52442	0,00815	0,033636	yes
LSM4	LSM4	LSM4	chr19:184170 shneg	shCHD1	OK	71,2998	85,0148	0,253815	1,42386	0,01315	0,0498141	yes
USP10	USP10	USP10	chr16:847335 shneg	shCHD1	OK	28,629	34,1104	0,252732	1,39913	0,01285	0,0488904	yes
PLS1	PLS1	PLS1	chr3:1423152 shneg	shCHD1	OK	41,0981	48,9625	0,252606	1,43799	0,012	0,0463014	yes
NPM1	NPM1	NPM1	chr5:1708147 shneg	shCHD1	OK	525,903	626,073	0,251534	1,50446	0,00755	0,0315808	yes
CALM2	CALM2	CALM2	chr2:4738722 shneg	shCHD1	OK	268,469	319,267	0,250007	1,46116	0,00925	0,0374435	yes
YWHAB	YWHAB	YWHAB	chr20:435143 shneg	shCHD1	OK	97,8853	116,208	0,247547	1,45228	0,01135	0,0441727	yes
PHLDB2	PHLDB2	PHLDB2	chr3:1113935 shneg	shCHD1	OK	52,2621	61,9307	0,244891	1,42423	0,01225	0,0470834	yes
LARP1	LARP1	LARP1	chr5:1540924 shneg	shCHD1	OK	57,7667	68,3723	0,243172	1,43424	0,0126	0,0481232	yes
HNRNPA2B1	HNRNPA2B1	HNRNPA2B1	chr7:2622955 shneg	shCHD1	OK	296,685	350,539	0,240644	1,45167	0,01205	0,0464557	yes
RPS15	RPS15	RPS15	chr19:143836 shneg	shCHD1	OK	1740,13	1467,71	-0,24563	-1,45149	0,0116	0,0449948	yes
NEAT1	NEAT1	NEAT1	chr11:651902 shneg	shCHD1	OK	276,421	232,687	-0,248475	-1,48731	0,0097	0,0389594	yes
RPL31	RPL31	RPL31	chr2:1016186 shneg	shCHD1	OK	2342,59	1970,42	-0,2496	-1,46597	0,00875	0,0357102	yes
HIP1R	HIP1R	HIP1R	chr12:123320 shneg	shCHD1	OK	47,6753	40,0805	-0,25034	-1,43043	0,0126	0,0481232	yes
H1F0	H1F0	H1F0	chr22:382011 shneg	shCHD1	OK	135,023	113,506	-0,250432	-1,44975	0,01095	0,0428555	yes
TKT	TKT	TKT	chr3:5325872 shneg	shCHD1	OK	418,754	351,55	-0,252374	-1,51041	0,00885	0,0360865	yes
CSRP1	CSRP1	CSRP1	chr1:2014526 shneg	shCHD1	OK	89,0379	74,7292	-0,252746	-1,4351	0,01255	0,0479717	yes
UBE2D3	UBE2D3	UBE2D3	chr4:1037171 shneg	shCHD1	OK	93,1702	78,0753	-0,255002	-1,44598	0,01175	0,0454375	yes
SLC4A7	SLC4A7	SLC4A7	chr3:2741421 shneg	shCHD1	OK	41,1475	34,477	-0,255171	-1,47187	0,0105	0,0414554	yes
HERPUD1	HERPUD1	HERPUD1	chr16:569657 shneg	shCHD1	OK	65,058	54,5095	-0,255217	-1,43093	0,01095	0,0428555	yes
NT5E	NT5E	NT5E	chr6:8615930 shneg	shCHD1	OK	135,485	113,435	-0,256271	-1,52408	0,00815	0,033636	yes
F2R	F2R	F2R	chr5:7601186 shneg	shCHD1	OK	88,8474	74,3709	-0,256591	-1,49484	0,00785	0,0326299	yes
TWF1	TWF1	TWF1	chr12:441875 shneg	shCHD1	OK	46,8875	39,2336	-0,257114	-1,43697	0,01185	0,0457733	yes
NACC2	NACC2	NACC2	chr9:1388983 shneg	shCHD1	OK	18,102	15,1418	-0,257605	-1,43416	0,01175	0,0454375	yes
RPL12	RPL12	RPL12	chr9:1302099 shneg	shCHD1	OK	2041,96	1707,09	-0,258414	-1,55362	0,0074	0,0310185	yes
PLCB3	PLCB3	PLCB3	chr11:640189 shneg	shCHD1	OK	16,8972	14,1182	-0,259229	-1,41856	0,0129	0,049027	yes
LOC220906	LOC220906	LOC220906	chr10:288088 shneg	shCHD1	OK	27,8867	23,2896	-0,259893	-1,46156	0,00975	0,0391039	yes
ARNT2	ARNT2	ARNT2	chr15:806966 shneg	shCHD1	OK	20,8094	17,3726	-0,260423	-1,45825	0,01005	0,0399619	yes
SIPA1L3	SIPA1L3	SIPA1L3	chr19:383978 shneg	shCHD1	OK	16,7619	13,992	-0,260587	-1,45371	0,01035	0,040956	yes
ITGB5	ITGB5	ITGB5	chr3:1244817 shneg	shCHD1	OK	177,782	148,383	-0,260781	-1,54921	0,0064	0,0274548	yes
GRB2	GRB2	GRB2	chr17:733141 shneg	shCHD1	OK	34,5755	28,8479	-0,261285	-1,4476	0,01125	0,0438693	yes
EEF1A1	EEF1A1	EEF1A1	chr6:7422547 shneg	shCHD1	OK	2685,81	2240,74	-0,261378	-1,46222	0,01075	0,0422389	yes
FBXW5	FBXW5	FBXW5	chr9:1398348 shneg	shCHD1	OK	65,4797	54,5997	-0,262154	-1,47103	0,00955	0,0384457	yes
PLEC	PLEC	PLEC	chr8:1449893 shneg	shCHD1	OK	46,5087	38,7176	-0,264513	-1,57144	0,00615	0,0265788	yes
MEGF8	MEGF8	MEGF8	chr19:428297 shneg	shCHD1	OK	16,6135	13,8258	-0,264992	-1,50526	0,00805	0,0332923	yes
ECH1	ECH1	ECH1	chr19:393060 shneg	shCHD1	OK	122,176	101,604	-0,266005	-1,48629	0,0086	0,0352322	yes
U2AF2	U2AF2	U2AF2	chr19:561654 shneg	shCHD1	OK	110,73	92,0817	-0,266056	-1,5566	0,006	0,0260761	yes
ATG2B	ATG2B	ATG2B	chr14:967475 shneg	shCHD1	OK	11,5613	9,61409	-0,266087	-1,48472	0,0099	0,0395347	yes
BTBD9	BTBD9	BTBD9	chr6:3813622 shneg	shCHD1	OK	45,4026	37,755	-0,26611	-1,54846	0,0074	0,0310185	yes
CLSTN3	CLSTN3	CLSTN3	chr12:728296 shneg	shCHD1	OK	20,6394	17,1551	-0,266764	-1,445	0,01245	0,0476418	yes
LZTS2	LZTS2	LZTS2	chr10:102756 shneg	shCHD1	OK	62,3394	51,8052	-0,267049	-1,48291	0,00955	0,0384457	yes
GALNT11	GALNT11	GALNT11	chr7:1517227 shneg	shCHD1	OK	49,2995	40,961	-0,267324	-1,4919	0,00815	0,033636	yes
SAT1	SAT1	SAT1	chrX:2380127 shneg	shCHD1	OK	651,761	541,325	-0,267849	-1,58841	0,0054	0,0238631	yes
HTT	HTT	HTT	chr4:3076407 shneg	shCHD1	OK	5,6137	4,65795	-0,269255	-1,45373	0,0122	0,0469301	yes
C3orf64	C3orf64	C3orf64	chr3:6902436 shneg	shCHD1	OK	16,9342	14,0481	-0,269566	-1,4445	0,01225	0,0470834	yes
MCAM	MCAM	MCAM	chr11:119179 shneg	shCHD1	OK	89,7858	74,4703	-0,269823	-1,56376	0,00635	0,0272991	yes
F2RL1	F2RL1	F2RL1	chr5:7611483 shneg	shCHD1	OK	339,032	280,726	-0,27226	-1,63485	0,0049	0,0220881	yes
SKI	SKI	SKI	chr1:2160133 shneg	shCHD1	OK	13,2448	10,9669	-0,272282	-1,46753	0,01175	0,0454375	yes
PRCP	PRCP	PRCP	chr11:825354 shneg	shCHD1	OK	31,3031	25,9175	-0,27238	-1,45108	0,01095	0,0428555	yes
NEBL	NEBL	NEBL	chr10:210689 shneg	shCHD1	OK	15,2638	12,635	-0,272693	-1,48307	0,01025	0,040618	yes
ADH5	ADH5	ADH5	chr4:9999212 shneg	shCHD1	OK	37,763	31,2585	-0,272724	-1,49241	0,00955	0,0384457	yes
PDP2	PDP2	PDP2	chr16:669143 shneg	shCHD1	OK	8,19477	6,78061	-0,273289	-1,44553	0,0124	0,0475026	yes
PRKAA1	PRKAA1	PRKAA1	chr5:4075948 shneg	shCHD1	OK	25,2938	20,9212	-0,273822	-1,52587	0,0089	0,036248	yes
MAP3K7	MAP3K7	MAP3K7	chr6:9122535 shneg	shCHD1	OK	26,0205	21,5197	-0,273991	-1,46262	0,0112	0,0437111	yes
NUB1	NUB1	NUB1	chr7:1510388 shneg	shCHD1	OK	24,2728	20,0729	-0,274091	-1,47468	0,01065	0,0419524	yes
STXBP5	STXBP5	STXBP5	chr6:1471625 shneg	shCHD1	OK	37,5093	31,0103	-0,274503	-1,59773	0,0046	0,0208912	yes
C14orf135	C14orf135	C14orf135	chr14:605586 shneg	shCHD1	OK	12,0967	9,9999	-0,274625	-1,42473	0,013	0,0492861	yes
FAM149A	FAM149A	FAM149A	chr4:1870659 shneg	shCHD1	OK	38,0542	31,4566	-0,274694	-1,49003	0,00915	0,0370927	yes
VMP1	VMP1	VMP1	chr17:577848 shneg	shCHD1	OK	115,271	95,2839	-0,274731	-1,57841	0,0055	0,0242742	yes
VEGFA	VEGFA	VEGFA	chr6:4373794 shneg	shCHD1	OK	117,453	97,075	-0,274914	-1,60985	0,00475	0,0214814	yes
EEF2	EEF2	EEF2	chr19:397605 shneg	shCHD1	OK	1118,77	924,28	-0,275508	-1,65548	0,0035	0,0166331	yes
USP11	USP11	USP11	chrX:4709231 shneg	shCHD1	OK	32,7434	27,0471	-0,275728	-1,51814	0,00795	0,0329472	yes
SPOPL	SPOPL	SPOPL	chr2:1392593 shneg	shCHD1	OK	10,1461	8,38096	-0,275741	-1,45646	0,01065	0,0419524	yes
DDR1	DDR1	DDR1	chr6_qbl_hap shneg	shCHD1	OK	35,415	29,2511	-0,275873	-1,52575	0,00895	0,036409	yes
CD63	CD63	CD63	chr12:561192 shneg	shCHD1	OK	484,231	399,902	-0,276049	-1,63321	0,00475	0,0214814	yes
ANKIB1	ANKIB1	ANKIB1	chr7:9187554 shneg	shCHD1	OK	18,9307	15,631	-0,276318	-1,52898	0,0078	0,0324802	yes
PIAS3	PIAS3	PIAS3	chr1:1455759 shneg	shCHD1	OK	24,0123	19,8205	-0,276784	-1,44061	0,0124	0,0475026	yes
SLC25A23	SLC25A23	SLC25A23	chr19:644007 shneg	shCHD1	OK	42,2753	34,8915	-0,276939	-1,55171	0,00665	0,0283439	yes
SOS2	SOS2	SOS2	chr14:505838 shneg	shCHD1	OK	12,3731	10,2119	-0,276955	-1,47367	0,01035	0,040956	yes
ATRX	ATRX	ATRX	chrX:7676035 shneg	shCHD1	OK	16,5679	13,6701	-0,277369	-1,55306	0,0075	0,0313999	yes
GTF3C6	GTF3C6	GTF3C6	chr6:1112797 shneg	shCHD1	OK	123,352	101,773	-0,277425	-1,51885	0,0083	0,0341639	yes
GPR107	GPR107	GPR107	chr9:1328159 shneg	shCHD1	OK	27,4347	22,6312	-0,27769	-1,58689	0,00595	0,0259073	yes

GOLGB1	GOLGB1	GOLGB1	chr3:1213820	shneg	shCHD1	OK	17,1565	14,1465	-0,278309	-1,57267	0,0059	0,0257621	yes
RNF213	RNF213	RNF213	chr17:782346	shneg	shCHD1	OK	18,9004	15,578	-0,27891	-1,42764	0,0122	0,0469301	yes
PHF12	PHF12	PHF12	chr17:272322	shneg	shCHD1	OK	16,084	13,2446	-0,280217	-1,49581	0,0093	0,0375913	yes
TSC22D3	TSC22D3	TSC22D3	chrX:1069564	shneg	shCHD1	OK	187,373	154,27	-0,280454	-1,62798	0,0048	0,0216934	yes
SUZ12	SUZ12	SUZ12	chr17:302640	shneg	shCHD1	OK	17,9065	14,7413	-0,280617	-1,51693	0,0091	0,036933	yes
ABCC4	ABCC4	ABCC4	chr13:956720	shneg	shCHD1	OK	45,1337	37,15	-0,280843	-1,59832	0,00525	0,0232593	yes
SLC35F5	SLC35F5	SLC35F5	chr2:1144719	shneg	shCHD1	OK	25,9477	21,3484	-0,281484	-1,49875	0,00845	0,0347403	yes
CELSR1	CELSR1	CELSR1	chr22:467567	shneg	shCHD1	OK	22,6937	18,6687	-0,281669	-1,62322	0,0042	0,0193522	yes
ATP2C1	ATP2C1	ATP2C1	chr3:1305693	shneg	shCHD1	OK	28,0443	23,0678	-0,281831	-1,46853	0,01005	0,0399619	yes
TYSND1	TYSND1	TYSND1	chr10:718977	shneg	shCHD1	OK	9,76963	8,0348	-0,282042	-1,42729	0,0107	0,0420661	yes
SDHA	SDHA	SDHA	chr5:218355-	shneg	shCHD1	OK	219,57	180,523	-0,282498	-1,66986	0,00395	0,0184012	yes
ATP11B	ATP11B	ATP11B	chr3:1825112	shneg	shCHD1	OK	10,566	8,68196	-0,283335	-1,52141	0,00875	0,0357102	yes
CHMP1B	CHMP1B	CHMP1B	chr18:116891	shneg	shCHD1	OK	27,0082	22,1864	-0,283721	-1,4984	0,00865	0,0353954	yes
MYSM1	MYSM1	MYSM1	chr1:5912041	shneg	shCHD1	OK	12,8957	10,5896	-0,284239	-1,54462	0,00685	0,0290629	yes
C10orf26	C10orf26	C10orf26	chr10:104503	shneg	shCHD1	OK	8,93984	7,3387	-0,284726	-1,43923	0,0123	0,0472104	yes
PRRC2B	PRRC2B	PRRC2B	chr9:1343054	shneg	shCHD1	OK	59,2846	48,6315	-0,285766	-1,70317	0,0021	0,0108578	yes
CTBP1	CTBP1	CTBP1	chr4:1205227	shneg	shCHD1	OK	41,9138	34,3794	-0,285881	-1,55506	0,00645	0,0276097	yes
UBXN1	UBXN1	UBXN1	chr11:624439	shneg	shCHD1	OK	87,6351	71,8775	-0,285967	-1,56842	0,006	0,0260761	yes
LOC642852	LOC642852	LOC642852	chr21:467079	shneg	shCHD1	OK	8,83007	7,24146	-0,286144	-1,52428	0,007	0,0295643	yes
HLCS	HLCS	HLCS	chr21:381231	shneg	shCHD1	OK	5,52078	4,52642	-0,286501	-1,43062	0,01285	0,0488904	yes
GIPC1	GIPC1	GIPC1	chr19:145885	shneg	shCHD1	OK	57,2578	46,9435	-0,286549	-1,5381	0,00695	0,0293888	yes
EXT2	EXT2	EXT2	chr11:441170	shneg	shCHD1	OK	21,716	17,8031	-0,286624	-1,54081	0,00795	0,0329472	yes
PAQR7	PAQR7	PAQR7	chr1:2618797	shneg	shCHD1	OK	11,2772	9,24459	-0,286727	-1,436	0,01185	0,0457733	yes
SUPT6H	SUPT6H	SUPT6H	chr17:269893	shneg	shCHD1	OK	27,1634	22,2551	-0,287527	-1,62308	0,0036	0,0170733	yes
RPL7L1	RPL7L1	RPL7L1	chr6:4284767	shneg	shCHD1	OK	86,785	71,0874	-0,287851	-1,57718	0,0061	0,0264201	yes
UNC13B	UNC13B	UNC13B	chr9:3516198	shneg	shCHD1	OK	7,03401	5,75995	-0,288291	-1,49264	0,01015	0,040279	yes
BRCC3	BRCC3	BRCC3	chrX:1542996	shneg	shCHD1	OK	18,6237	15,2395	-0,289321	-1,50743	0,00935	0,0377496	yes
HS1BP3	HS1BP3	HS1BP3	chr2:2081756	shneg	shCHD1	OK	23,3904	19,1398	-0,28934	-1,51908	0,00785	0,0326299	yes
APP	APP	APP	chr21:272528	shneg	shCHD1	OK	191,197	156,439	-0,28946	-1,72612	0,00295	0,0144329	yes
TJP2	TJP2	TJP2	chr9:7173617	shneg	shCHD1	OK	38,8241	31,7597	-0,289756	-1,62548	0,00525	0,0232593	yes
FKBP15	FKBP15	FKBP15	chr9:1159277	shneg	shCHD1	OK	12,3276	10,0836	-0,289883	-1,52187	0,0086	0,0352322	yes
IPO13	IPO13	IPO13	chr1:4441247	shneg	shCHD1	OK	13,3864	10,9488	-0,289995	-1,51777	0,00675	0,0286999	yes
ZNF605	ZNF605	ZNF605	chr12:133498	shneg	shCHD1	OK	5,61096	4,58882	-0,290126	-1,45951	0,0107	0,0420661	yes
CBR1	CBR1	CBR1	chr21:374419	shneg	shCHD1	OK	54,2845	44,3911	-0,29027	-1,53445	0,0077	0,0321599	yes
CCDC88A	CCDC88A	CCDC88A	chr2:5551497	shneg	shCHD1	OK	3,78393	3,09361	-0,290591	-1,45428	0,0111	0,0433694	yes
LSS	LSS	LSS	chr21:476083	shneg	shCHD1	OK	10,4063	8,50698	-0,290743	-1,478	0,009	0,0365911	yes
SCOC	SCOC	SCOC	chr4:1411784	shneg	shCHD1	OK	21,9006	17,9024	-0,29082	-1,48679	0,0091	0,036933	yes
ROBO3	ROBO3	ROBO3	chr11:124735	shneg	shCHD1	OK	7,7123	6,30312	-0,291095	-1,46514	0,00965	0,0388146	yes
TMEM33	TMEM33	TMEM33	chr4:4193713	shneg	shCHD1	OK	8,62737	7,05061	-0,291173	-1,55433	0,0067	0,0285134	yes
THUMPD1	THUMPD1	THUMPD1	chr16:207449	shneg	shCHD1	OK	13,6651	11,1626	-0,291823	-1,54065	0,0069	0,0292395	yes
TAF8	TAF8	TAF8	chr6:4201825	shneg	shCHD1	OK	9,62854	7,86362	-0,292123	-1,49871	0,00865	0,0353954	yes
HPS5	HPS5	HPS5	chr11:183002	shneg	shCHD1	OK	5,67346	4,63288	-0,292319	-1,41383	0,013	0,0492861	yes
PHC3	PHC3	PHC3	chr3:1698053	shneg	shCHD1	OK	5,42657	4,4293	-0,292961	-1,5665	0,00795	0,0329472	yes
GOPC	GOPC	GOPC	chr6:1178038	shneg	shCHD1	OK	13,5114	11,0266	-0,293196	-1,48537	0,0098	0,0392479	yes
MICAL1	MICAL1	MICAL1	chr6:1097652	shneg	shCHD1	OK	16,7747	13,689	-0,29327	-1,54228	0,00805	0,0332923	yes
MYL10	MYL10	MYL10	chr7:1012566	shneg	shCHD1	OK	837,518	683,291	-0,29362	-1,75074	0,00285	0,0140225	yes
SERINC1	SERINC1	SERINC1	chr6:1227644	shneg	shCHD1	OK	73,8019	60,1941	-0,294038	-1,68467	0,003	0,0146313	yes
NEK7	NEK7	NEK7	chr1:1981261	shneg	shCHD1	OK	9,30992	7,59052	-0,294569	-1,49578	0,0097	0,0389594	yes
NCOA7	NCOA7	NCOA7	chr6:1261023	shneg	shCHD1	OK	4,19214	3,41754	-0,294729	-1,41927	0,01265	0,0482745	yes
TMEM167B	TMEM167B	TMEM167B	chr1:1096334	shneg	shCHD1	OK	14,8904	12,1357	-0,295122	-1,50156	0,00995	0,0396775	yes
ZMIZ1	ZMIZ1	ZMIZ1	chr10:808287	shneg	shCHD1	OK	8,92705	7,27422	-0,295392	-1,57728	0,0069	0,0292395	yes
SHISA9	SHISA9	SHISA9	chr16:129954	shneg	shCHD1	OK	5,2035	4,24003	-0,295407	-1,47678	0,00855	0,0350893	yes
EFHC1	EFHC1	EFHC1	chr6:5228499	shneg	shCHD1	OK	12,6107	10,268	-0,296488	-1,56964	0,0065	0,0277896	yes
PCGF3	PCGF3	PCGF3	chr4:699572-	shneg	shCHD1	OK	9,56065	7,78258	-0,29686	-1,56072	0,00635	0,0272991	yes
FAXC	FAXC	FAXC	chr6:9972079	shneg	shCHD1	OK	1,4791	1,204	-0,296879	-1,42765	0,01295	0,0491768	yes
PTEN	PTEN	PTEN	chr10:896231	shneg	shCHD1	OK	6,44516	5,24486	-0,297312	-1,50193	0,00915	0,0370927	yes
ZNF318	ZNF318	ZNF318	chr6:4330380	shneg	shCHD1	OK	10,081	8,203	-0,297409	-1,60949	0,00535	0,0236722	yes
HERC5	HERC5	HERC5	chr4:8937826	shneg	shCHD1	OK	11,6963	9,51713	-0,29745	-1,51933	0,0086	0,0352322	yes
KIFC2	KIFC2	KIFC2	chr8:1456917	shneg	shCHD1	OK	27,4286	22,306	-0,298247	-1,49852	0,00925	0,0374435	yes
GPS1	GPS1	GPS1	chr17:800097	shneg	shCHD1	OK	41,2446	33,5407	-0,298292	-1,59213	0,0049	0,0220881	yes
YPEL5	YPEL5	YPEL5	chr2:3036974	shneg	shCHD1	OK	38,8938	31,6268	-0,298392	-1,61122	0,00595	0,0259073	yes
PLA2G12A	PLA2G12A	PLA2G12A	chr4:1106311	shneg	shCHD1	OK	4,33813	3,52747	-0,29844	-1,43411	0,0129	0,049027	yes
CRAT	CRAT	CRAT	chr9:1318570	shneg	shCHD1	OK	34,9355	28,4041	-0,298597	-1,62611	0,00415	0,0191853	yes
PPP2R5A	PPP2R5A	PPP2R5A	chr1:2124588	shneg	shCHD1	OK	16,6996	13,5758	-0,298779	-1,55056	0,00695	0,0293888	yes
PNRC1	PNRC1	PNRC1	chr6:8979042	shneg	shCHD1	OK	38,5457	31,3323	-0,29892	-1,61154	0,00545	0,0240688	yes
FAM100B	FAM100B	FAM100B	chr17:742612	shneg	shCHD1	OK	21,4979	17,4727	-0,299093	-1,48357	0,0093	0,0375913	yes
IFT57	IFT57	IFT57	chr3:1078796	shneg	shCHD1	OK	18,3139	14,882	-0,29937	-1,57669	0,00645	0,0276097	yes
NINJ1	NINJ1	NINJ1	chr9:9588377	shneg	shCHD1	OK	37,0852	30,1339	-0,299452	-1,53458	0,00765	0,0319703	yes
RTN4	RTN4	RTN4	chr2:5519932	shneg	shCHD1	OK	238,253	193,591	-0,299481	-1,70621	0,00285	0,0140225	yes
SUOX	SUOX	SUOX	chr12:563910	shneg	shCHD1	OK	16,0709	13,0553	-0,299812	-1,50056	0,00925	0,0374435	yes
MGAT4C	MGAT4C	MGAT4C	chr12:863730	shneg	shCHD1	OK	26,5104	21,5354	-0,299852	-1,55214	0,0054	0,0238631	yes
SCP2	SCP2	SCP2	chr1:5339290	shneg	shCHD1	OK	40,1343	32,5932	-0,300265	-1,47168	0,01	0,0398199	yes
USP46	USP46	USP46	chr4:5345712	shneg	shCHD1	OK	3,02995	2,46005	-0,300607	-1,44307	0,0124	0,0475026	yes
CRIPT	CRIPT	CRIPT	chr2:4684432	shneg	shCHD1	OK	8,10495	6,58018	-0,300677	-1,44152	0,01245	0,0476418	yes
LRSAM1	LRSAM1	LRSAM1	chr9:1302137	shneg	shCHD1	OK	14,4111	11,6991	-0,300792	-1,51794	0,0086	0,0352322	yes
EDEM3	EDEM3	EDEM3	chr1:1846596	shneg	shCHD1	OK	7,35532	5,9699	-0,301082	-1,56417	0,0067	0,0285134	yes
SCAI	SCAI	SCAI	chr9:1277048	shneg	shCHD1	OK	2,7023	2,1931	-0,301221	-1,50646	0,00985	0,0393915	yes
SOS1	SOS1	SOS1	chr2:3920868	shneg	shCHD1	OK	6,07181	4,92731	-0,301326	-1,56613	0,00605	0,0262198	yes
P4HTM	P4HTM	P4HTM	chr3:4902734	shneg	shCHD1	OK	28,6259	23,2284	-0,301437	-1,57973	0,00645	0,0276097	yes
FBXO10	FBXO10	FBXO10	chr9:3751088	shneg	shCHD1	OK	4,06765	3,3005	-0,301509	-1,45745	0,0105	0,0414554	yes
SRF	SRF	SRF	chr6:4313891	shneg	shCHD1	OK	23,6706	19,201	-0,301912	-1,65654	0,00365	0,0172576	yes
KIDINS220	KIDINS220	KIDINS220	chr2:8868986	shneg	shCHD1	OK	12,1122	9,82309	-0,302208	-1,64758	0,0039	0,018217	yes
PET112	PET112	PET112	chr4:1525918	shneg	shCHD1	OK	7,45227	6,04271	-0,302484	-1,45659	0,01165	0,0451385	yes
C8orf4	C8orf4	C8orf4	chr8:4001098	shneg	shCHD1	OK	103,864	84,2145	-0,302554	-1,7155	0,00265	0,0132017	yes
SLC50A1	SLC50A1	SLC50A1	chr1:1551082	shneg	shCHD1	OK	26,78	21,7119	-0,302669	-1,4788	0,00995	0,0396775	yes
RCOR2	RCOR2	RCOR2	chr11:636786	shneg	shCHD1	OK	9,93504	8,05428	-0,302771	-1,45822	0,01125	0,0438693	yes
VPS11	VPS11	VPS11	chr11:118938	shneg	shCHD1	OK	10,9491	8,87634	-0,302776	-1,52209	0,0097	0,0389594	yes
GNPTAB	GNPTAB	GNPTAB	chr12:102139	shneg	shCHD1	OK	6,90876	5,59999	-0,303003	-1,54157	0,00715	0,0300974	yes
RNFT1	RNFT1	RNFT1	chr17:580297	shneg	shCHD1	OK	10,7872	8,7433	-0,303069	-1,43453	0,01285	0,0488904	yes
QPCTL	QPCTL	QPCTL	chr19:461957	shneg	shCHD1	OK	9,56024	7,74433	-0,303907	-1,4667	0,0109	0,0427438	yes
CERS2	CERS2	CERS2	chr1:1509376	shneg	shCHD1	OK	111,049	89,9534	-0,303947	-1,76747	0,00175	0,00931439	yes
ABHD8	ABHD8	ABHD8	chr19:174029	shneg	shCHD1	OK	14,2556	11,547	-0,304009	-1,48587	0,0101	0,0401378	yes
OS9	OS9	OS9	chr12:580877	shneg	shCHD1	OK	93,9313	76,0483	-0,30469	-1,74912	0,00175	0,00931439	yes

HSP90AB2P	HSP90AB2P	HSP90AB2P	chr4:1333503	shneg	shCHD1	OK	42,302	34,2372	-0,305158	-1,74162	0,0028	0,0138449	yes
LOC10013156	LOC10013156	LOC10013156	chr1:9377566	shneg	shCHD1	OK	7,0454	5,70146	-0,305349	-1,49138	0,01	0,0398199	yes
CUX1	CUX1	CUX1	chr7:1014591	shneg	shCHD1	OK	79,814	64,5863	-0,305415	-1,44479	0,01265	0,0482745	yes
RSPRY1	RSPRY1	RSPRY1	chr16:572202	shneg	shCHD1	OK	12,7491	10,316	-0,305518	-1,46938	0,0118	0,0456055	yes
TMBIM4	TMBIM4	TMBIM4	chr12:665307	shneg	shCHD1	OK	20,2964	16,4219	-0,305604	-1,534	0,0079	0,0327888	yes
MFSD1	MFSD1	MFSD1	chr3:1585197	shneg	shCHD1	OK	11,6377	9,41546	-0,305704	-1,48263	0,00985	0,0393915	yes
FGFRL1	FGFRL1	FGFRL1	chr4:1005609	shneg	shCHD1	OK	12,4655	10,0827	-0,306055	-1,55681	0,0061	0,0264201	yes
SLC35D1	SLC35D1	SLC35D1	chr1:6746501	shneg	shCHD1	OK	2,25972	1,82769	-0,306124	-1,47189	0,0104	0,0410955	yes
PXN	PXN	PXN	chr12:120639	shneg	shCHD1	OK	112,35	90,8433	-0,30655	-1,79702	0,00145	0,00793286	yes
PLP2	PLP2	PLP2	chrX:4902818	shneg	shCHD1	OK	174,365	140,945	-0,306982	-1,72754	0,00235	0,0119595	yes
SP1	SP1	SP1	chr12:537739	shneg	shCHD1	OK	25,8058	20,8556	-0,307257	-1,748	0,0026	0,0129897	yes
PRMT6	PRMT6	PRMT6	chr1:1075992	shneg	shCHD1	OK	8,18779	6,6153	-0,307668	-1,44406	0,01315	0,0498141	yes
CTSL2	CTSL2	CTSL2	chr9:9979195	shneg	shCHD1	OK	6,86468	5,54606	-0,30773	-1,51625	0,00855	0,0350893	yes
TOR1AIP1	TOR1AIP1	TOR1AIP1	chr1:1798514	shneg	shCHD1	OK	17,2438	13,9281	-0,308085	-1,64033	0,0045	0,0205241	yes
PPM1M	PPM1M	PPM1M	chr3:5227980	shneg	shCHD1	OK	13,4141	10,8284	-0,308924	-1,45636	0,0114	0,0443302	yes
STX1B	STX1B	STX1B	chr16:310005	shneg	shCHD1	OK	4,14462	3,34554	-0,309002	-1,4645	0,01215	0,0467765	yes
ZDHHC14	ZDHHC14	ZDHHC14	chr6:1578025	shneg	shCHD1	OK	4,86505	3,92702	-0,309019	-1,43249	0,013	0,0492861	yes
TBC1D24	TBC1D24	TBC1D24	chr16:252514	shneg	shCHD1	OK	4,96233	4,00489	-0,309255	-1,54555	0,00825	0,0339983	yes
IDH1	IDH1	IDH1	chr2:2091009	shneg	shCHD1	OK	31,9635	25,7928	-0,309454	-1,65889	0,0043	0,0197347	yes
LRBA	LRBA	LRBA	chr4:1511858	shneg	shCHD1	OK	12,1158	9,7765	-0,309502	-1,72874	0,00205	0,0106507	yes
RABGAP1	RABGAP1	RABGAP1	chr9:1257032	shneg	shCHD1	OK	15,7378	12,6973	-0,309718	-1,67155	0,00355	0,0168592	yes
RICTOR	RICTOR	RICTOR	chr5:3893802	shneg	shCHD1	OK	7,44316	6,00305	-0,310221	-1,63855	0,0049	0,0220881	yes
AKIRIN2	AKIRIN2	AKIRIN2	chr6:8838457	shneg	shCHD1	OK	29,4725	23,7674	-0,310381	-1,59667	0,00525	0,0232593	yes
PLEKHM1	PLEKHM1	PLEKHM1	chr17_ctg5_h	shneg	shCHD1	OK	9,76485	7,87345	-0,310602	-1,62971	0,00445	0,020336	yes
CCNG2	CCNG2	CCNG2	chr4:7807835	shneg	shCHD1	OK	7,65824	6,17457	-0,310675	-1,59374	0,00595	0,0259073	yes
ZNF274	ZNF274	ZNF274	chr19:586943	shneg	shCHD1	OK	10,8002	8,70716	-0,310786	-1,51755	0,00745	0,0312187	yes
C14orf129	C14orf129	C14orf129	chr14:968460	shneg	shCHD1	OK	28,0692	22,6263	-0,310985	-1,634	0,0045	0,0205241	yes
DYNLL2	DYNLL2	DYNLL2	chr17:561607	shneg	shCHD1	OK	39,9783	32,2176	-0,311368	-1,63706	0,0045	0,0205241	yes
CTSD	CTSD	CTSD	chr11:149068	shneg	shCHD1	OK	117,621	94,7792	-0,311508	-1,6858	0,00355	0,0168592	yes
UFC1	UFC1	UFC1	chr1:1611235	shneg	shCHD1	OK	65,8422	53,053	-0,311578	-1,65631	0,0036	0,0170733	yes
WWC3	WWC3	WWC3	chrX:9983794	shneg	shCHD1	OK	6,58229	5,30347	-0,311654	-1,60319	0,0058	0,0254211	yes
ACOT2	ACOT2	ACOT2	chr14:740343	shneg	shCHD1	OK	14,824	11,9435	-0,311707	-1,49804	0,0093	0,0375913	yes
HEBP1	HEBP1	HEBP1	chr12:131277	shneg	shCHD1	OK	26,7585	21,5576	-0,311798	-1,51576	0,00715	0,0300974	yes
FAM89B	FAM89B	FAM89B	chr11:653398	shneg	shCHD1	OK	38,292	30,849	-0,31182	-1,61146	0,0056	0,0246843	yes
ASH1L	ASH1L	ASH1L	chr1:1553050	shneg	shCHD1	OK	10,3198	8,31281	-0,312007	-1,70399	0,0024	0,0121606	yes
CLN3	CLN3	CLN3	chr16:284885	shneg	shCHD1	OK	13,0268	10,4926	-0,312111	-1,48754	0,0099	0,0395347	yes
KIAA1530	KIAA1530	KIAA1530	chr4:1341103	shneg	shCHD1	OK	3,39514	2,73434	-0,312281	-1,47068	0,0098	0,0392479	yes
PPP1R9B	PPP1R9B	PPP1R9B	chr17:482111	shneg	shCHD1	OK	33,9535	27,3427	-0,312407	-1,74148	0,002	0,0104181	yes
CCNI	CCNI	CCNI	chr4:7796917	shneg	shCHD1	OK	75,1684	60,5316	-0,312438	-1,74256	0,00215	0,0110834	yes
IFT80	IFT80	IFT80	chr3:1599747	shneg	shCHD1	OK	16,0194	12,899	-0,312561	-1,63979	0,00395	0,0184012	yes
CD59	CD59	CD59	chr11:337245	shneg	shCHD1	OK	98,5389	79,3426	-0,312597	-1,85922	0,0014	0,00768951	yes
ARL13B	ARL13B	ARL13B	chr3:9369898	shneg	shCHD1	OK	8,89623	7,16297	-0,312636	-1,56745	0,00615	0,0265788	yes
MAPKBP1	MAPKBP1	MAPKBP1	chr15:420666	shneg	shCHD1	OK	6,76314	5,44522	-0,312703	-1,63005	0,0038	0,0178336	yes
AMPD2	AMPD2	AMPD2	chr1:1101624	shneg	shCHD1	OK	23,6774	19,0631	-0,312724	-1,67412	0,0042	0,0193522	yes
MAP3K10	MAP3K10	MAP3K10	chr19:406976	shneg	shCHD1	OK	7,1548	5,75987	-0,312876	-1,49549	0,0087	0,0355373	yes
CEP350	CEP350	CEP350	chr1:1799239	shneg	shCHD1	OK	7,12854	5,73865	-0,312896	-1,6938	0,0031	0,0150558	yes
REV3L	REV3L	REV3L	chr6:1116202	shneg	shCHD1	OK	5,90579	4,7536	-0,31311	-1,65434	0,00395	0,0184012	yes
PON3	PON3	PON3	chr7:9498918	shneg	shCHD1	OK	38,063	30,635	-0,313207	-1,59134	0,0046	0,0208912	yes
CFL2	CFL2	CFL2	chr14:351795	shneg	shCHD1	OK	23,5734	18,973	-0,313218	-1,67142	0,0037	0,0174466	yes
TACC2	TACC2	TACC2	chr10:123748	shneg	shCHD1	OK	9,39392	7,56023	-0,313297	-1,55372	0,00605	0,0262198	yes
ATM	ATM	ATM	chr11:108093	shneg	shCHD1	OK	11,045	8,88847	-0,313382	-1,74508	0,00215	0,0110834	yes
C6orf108	C6orf108	C6orf108	chr6:4319336	shneg	shCHD1	OK	93,6708	75,3772	-0,313472	-1,61399	0,00515	0,0229918	yes
ORC4	ORC4	ORC4	chr2:1486025	shneg	shCHD1	OK	32,028	25,7683	-0,313738	-1,68481	0,0034	0,0162243	yes
TRMT2B	TRMT2B	TRMT2B	chrX:1002643	shneg	shCHD1	OK	9,72852	7,82681	-0,313796	-1,5438	0,007	0,0295643	yes
OTUD4	OTUD4	OTUD4	chr4:1460548	shneg	shCHD1	OK	11,613	9,34223	-0,313905	-1,52955	0,00425	0,0195438	yes
FBXL3	FBXL3	FBXL3	chr13:775793	shneg	shCHD1	OK	13,2097	10,6246	-0,314191	-1,62476	0,005	0,0224661	yes
RGS17	RGS17	RGS17	chr6:1533320	shneg	shCHD1	OK	23,5306	18,9197	-0,314649	-1,55074	0,0079	0,0327888	yes
RPS27	RPS27	RPS27	chr1:1539632	shneg	shCHD1	OK	3713,15	2985,5	-0,314672	-1,86877	0,0009	0,00533451	yes
PARP3	PARP3	PARP3	chr3:5197636	shneg	shCHD1	OK	9,92953	7,98367	-0,314673	-1,49437	0,01055	0,0416174	yes
KCNN4	KCNN4	KCNN4	chr19:442706	shneg	shCHD1	OK	74,535	59,925	-0,31476	-1,77314	0,00185	0,00975716	yes
CORO1B	CORO1B	CORO1B	chr11:672055	shneg	shCHD1	OK	54,8565	44,0933	-0,315103	-1,72666	0,0022	0,011316	yes
PRPSAP1	PRPSAP1	PRPSAP1	chr17:743068	shneg	shCHD1	OK	18,8723	15,1695	-0,315104	-1,61894	0,0052	0,0231261	yes
NPAS2	NPAS2	NPAS2	chr2:1014366	shneg	shCHD1	OK	21,9301	17,6268	-0,315143	-1,71149	0,00325	0,0156858	yes
YIPF3	YIPF3	YIPF3	chr6:4347956	shneg	shCHD1	OK	119,566	96,0862	-0,315406	-1,78439	0,0017	0,00909693	yes
PI4K2B	PI4K2B	PI4K2B	chr4:2523565	shneg	shCHD1	OK	26,2344	21,0795	-0,315622	-1,72131	0,00295	0,0144329	yes
TAF13	TAF13	TAF13	chr1:1096069	shneg	shCHD1	OK	38,7132	31,0963	-0,316081	-1,5308	0,00755	0,0315808	yes
ATAD2B	ATAD2B	ATAD2B	chr2:2397153	shneg	shCHD1	OK	2,25656	1,81251	-0,31613	-1,50107	0,0086	0,0352322	yes
USF1	USF1	USF1	chr1:1610090	shneg	shCHD1	OK	20,219	16,2398	-0,316174	-1,5806	0,00565	0,0248732	yes
NUCB1	NUCB1	NUCB1	chr19:494033	shneg	shCHD1	OK	94,7223	76,0662	-0,316449	-1,81651	0,001	0,0058183	yes
TM9SF1	TM9SF1	TM9SF1	chr14:246583	shneg	shCHD1	OK	44,3738	35,6324	-0,316519	-1,7287	0,00315	0,015272	yes
C4orf48	C4orf48	C4orf48	chr4:2043719	shneg	shCHD1	OK	76,2003	61,1731	-0,3169	-1,46508	0,0103	0,0407813	yes
CDC42BPB	CDC42BPB	CDC42BPB	chr14:103398	shneg	shCHD1	OK	41,5216	33,3313	-0,316982	-1,83333	0,00155	0,00838119	yes
IKBKAP	IKBKAP	IKBKAP	chr9:1116297	shneg	shCHD1	OK	13,6203	10,9317	-0,317246	-1,7168	0,0035	0,0166331	yes
RAPGEF2	RAPGEF2	RAPGEF2	chr4:1601889	shneg	shCHD1	OK	5,12559	4,11241	-0,317735	-1,59773	0,0056	0,0246843	yes
PSME1	PSME1	PSME1	chr14:246053	shneg	shCHD1	OK	268,671	215,543	-0,317869	-1,73274	0,00285	0,0140225	yes
JRKL	JRKL	JRKL	chr11:961231	shneg	shCHD1	OK	4,94111	3,96347	-0,31807	-1,5311	0,00735	0,0308368	yes
IFNGR1	IFNGR1	IFNGR1	chr6:1375186	shneg	shCHD1	OK	7,60792	6,10254	-0,318093	-1,50447	0,0082	0,0338022	yes
C1D	C1D	C1D	chr2:6826933	shneg	shCHD1	OK	16,8939	13,5509	-0,318113	-1,51555	0,0078	0,0324802	yes
BLVRB	BLVRB	BLVRB	chr19:409536	shneg	shCHD1	OK	57,9491	46,4778	-0,318244	-1,62131	0,0047	0,0212899	yes
SSR4	SSR4	SSR4	chrX:1530512	shneg	shCHD1	OK	288,275	231,208	-0,318255	-1,4763	0,01005	0,0399619	yes
PRKAR1A	PRKAR1A	PRKAR1A	chr17:665081	shneg	shCHD1	OK	88,7024	71,1411	-0,318289	-1,84438	0,0012	0,00680293	yes
FBL	FBL	FBL	chr19:403250	shneg	shCHD1	OK	459,12	368,213	-0,318331	-1,87558	0,0007	0,00428773	yes
DIAPH2	DIAPH2	DIAPH2	chrX:9593966	shneg	shCHD1	OK	5,68793	4,56046	-0,318726	-1,47228	0,01065	0,0419524	yes
CRELD1	CRELD1	CRELD1	chr3:9975523	shneg	shCHD1	OK	15,1541	12,1501	-0,318736	-1,57334	0,00705	0,0297484	yes
CASP7	CASP7	CASP7	chr10:115438	shneg	shCHD1	OK	12,3647	9,91323	-0,318801	-1,55739	0,0074	0,0310185	yes
WSB2	WSB2	WSB2	chr12:118470	shneg	shCHD1	OK	38,9606	31,2266	-0,31924	-1,75803	0,0028	0,0138449	yes
SRCIN1	SRCIN1	SRCIN1	chr17:366862	shneg	shCHD1	OK	19,2852	15,455	-0,31942	-1,78023	0,00145	0,00793286	yes
KAT2B	KAT2B	KAT2B	chr3:2008152	shneg	shCHD1	OK	3,20506	2,56747	-0,320001	-1,55505	0,00715	0,0300974	yes
KDM4C	KDM4C	KDM4C	chr9:6720862	shneg	shCHD1	OK	4,00985	3,21205	-0,320053	-1,48853	0,01095	0,0428555	yes
FURIN	FURIN	FURIN	chr15:914118	shneg	shCHD1	OK	20,071	16,0752	-0,320272	-1,73133	0,0033	0,0158557	yes
CLEC11A	CLEC11A	CLEC11A	chr19:512266	shneg	shCHD1	OK	215,247	172,388	-0,320336	-1,84394	0,00125	0,00702633	yes
DNAJB12	DNAJB12	DNAJB12	chr10:740925	shneg	shCHD1	OK	14,3498	11,4843	-0,321362	-1,61259	0,0043	0,0197347	yes
FAM193A	FAM193A	FAM193A	chr4:2627158	shneg	shCHD1	OK	7,2359	5,79058	-0,321465	-1,61064	0,0052	0,0231261	yes

CALCOCO1	CALCOCO1	CALCOCO1	chr12:541049 shneg	shCHD1	OK	22,5059	18,0074	-0,321709	-1,7044	0,003	0,0146313	yes
ZNF217	ZNF217	ZNF217	chr20:521836 shneg	shCHD1	OK	41,5696	33,2534	-0,322027	-1,84839	0,00125	0,00702633	yes
USP21	USP21	USP21	chr1:1611292 shneg	shCHD1	OK	13,9184	11,1339	-0,322042	-1,58794	0,0064	0,0274548	yes
TBCB	TBCB	TBCB	chr19:366046 shneg	shCHD1	OK	46,6037	37,2791	-0,322077	-1,46995	0,0115	0,0446442	yes
SLC44A2	SLC44A2	SLC44A2	chr19:107131 shneg	shCHD1	OK	22,2583	17,8026	-0,322255	-1,72401	0,0033	0,0158557	yes
PDSS2	PDSS2	PDSS2	chr6:1074737 shneg	shCHD1	OK	5,28286	4,22454	-0,322524	-1,52093	0,00875	0,0357102	yes
APOLD1	APOLD1	APOLD1	chr12:128788 shneg	shCHD1	OK	2,8446	2,27388	-0,323073	-1,50718	0,0088	0,0358932	yes
KIAA1244	KIAA1244	KIAA1244	chr6:1384830 shneg	shCHD1	OK	3,10394	2,48107	-0,323138	-1,63805	0,00345	0,0164347	yes
C1orf9	C1orf9	C1orf9	chr1:1725022 shneg	shCHD1	OK	5,6929	4,54901	-0,323612	-1,60207	0,006	0,0260761	yes
INTS2	INTS2	INTS2	chr17:599427 shneg	shCHD1	OK	3,723	2,97477	-0,323686	-1,55557	0,00755	0,0315808	yes
POMGNT1	POMGNT1	POMGNT1	chr1:4665435 shneg	shCHD1	OK	29,5561	23,6122	-0,323923	-1,69518	0,003	0,0146313	yes
FBXW4	FBXW4	FBXW4	chr10:103370 shneg	shCHD1	OK	14,6551	11,7062	-0,324131	-1,60545	0,0052	0,0231261	yes
PANX1	PANX1	PANX1	chr11:938620 shneg	shCHD1	OK	7,90346	6,31254	-0,324264	-1,54002	0,00735	0,0308368	yes
AMPD3	AMPD3	AMPD3	chr11:104718 shneg	shCHD1	OK	7,70596	6,15262	-0,324774	-1,60842	0,0052	0,0231261	yes
TSPYL1	TSPYL1	TSPYL1	chr6:1165960 shneg	shCHD1	OK	19,7189	15,7404	-0,325107	-1,78704	0,00185	0,00975716	yes
CRTC1	CRTC1	CRTC1	chr19:187944 shneg	shCHD1	OK	9,2846	7,4097	-0,325424	-1,71452	0,0032	0,015482	yes
FOSL1	FOSL1	FOSL1	chr11:656596 shneg	shCHD1	OK	91,1433	72,7312	-0,325562	-1,81853	0,0018	0,00951868	yes
ADI1	ADI1	ADI1	chr2:3501689 shneg	shCHD1	OK	33,0964	26,4089	-0,325651	-1,69924	0,00375	0,0176465	yes
MAP3K6	MAP3K6	MAP3K6	chr1:2768166 shneg	shCHD1	OK	6,21663	4,95895	-0,326098	-1,58509	0,006	0,0260761	yes
PARP11	PARP11	PARP11	chr12:391802 shneg	shCHD1	OK	2,91973	2,32856	-0,326394	-1,51176	0,00725	0,0304815	yes
ADPRHL1	ADPRHL1	ADPRHL1	chr13:114076 shneg	shCHD1	OK	7,48579	5,96961	-0,326518	-1,5063	0,00815	0,033636	yes
SYNGR3	SYNGR3	SYNGR3	chr16:203994 shneg	shCHD1	OK	6,88384	5,48873	-0,326741	-1,52198	0,0085	0,0349355	yes
C11orf68	C11orf68	C11orf68	chr11:656842 shneg	shCHD1	OK	38,3246	30,5519	-0,327008	-1,69865	0,0031	0,0150558	yes
MT1E	MT1E	MT1E	chr16:566595 shneg	shCHD1	OK	310,617	247,513	-0,327632	-1,79004	0,0021	0,0108578	yes
LIMA1	LIMA1	LIMA1	chr12:505695 shneg	shCHD1	OK	22,8749	18,227	-0,327683	-1,7597	0,00265	0,0132017	yes
ZDHHC1	ZDHHC1	ZDHHC1	chr16:674283 shneg	shCHD1	OK	7,45917	5,9428	-0,327872	-1,55056	0,00685	0,0290629	yes
RGS14	RGS14	RGS14	chr5:1767848 shneg	shCHD1	OK	17,6073	14,0255	-0,328123	-1,67217	0,0044	0,0201471	yes
C12orf5	C12orf5	C12orf5	chr12:443035 shneg	shCHD1	OK	2,84137	2,2633	-0,328157	-1,56718	0,00585	0,0255919	yes
CLIP2	CLIP2	CLIP2	chr7:7370380 shneg	shCHD1	OK	18,8345	15,0013	-0,32829	-1,80562	0,00135	0,00748275	yes
RPL23A	RPL23A	RPL23A	chr17:270469 shneg	shCHD1	OK	972,67	774,706	-0,328301	-1,95304	0,0006	0,00377139	yes
CYP2S1	CYP2S1	CYP2S1	chr19:416991 shneg	shCHD1	OK	15,16	12,0735	-0,32843	-1,66563	0,0041	0,0189794	yes
FAM214A	FAM214A	FAM214A	chr15:528735 shneg	shCHD1	OK	3,93585	3,1343	-0,328533	-1,56161	0,00615	0,0265788	yes
TMEM212	TMEM212	TMEM212	chr3:1715611 shneg	shCHD1	OK	36,719	29,2382	-0,328674	-1,7467	0,00255	0,0127904	yes
SLC9A7	SLC9A7	SLC9A7	chrX:4646637 shneg	shCHD1	OK	6,81645	5,42683	-0,328911	-1,5634	0,0078	0,0324802	yes
WDR1	WDR1	WDR1	chr4:1007596 shneg	shCHD1	OK	69,3811	55,2356	-0,328944	-1,88336	0,0015	0,00814879	yes
TIMP1	TIMP1	TIMP1	chrX:4742049 shneg	shCHD1	OK	376,072	299,385	-0,329005	-1,69124	0,00285	0,0140225	yes
LINC00410	LINC00410	LINC00410	chr13:915432 shneg	shCHD1	OK	6,28642	5,00423	-0,329092	-1,47768	0,00925	0,0374435	yes
BAK1	BAK1	BAK1	chr6:3354032 shneg	shCHD1	OK	10,1033	8,04246	-0,329121	-1,54937	0,0066	0,0281825	yes
H6PD	H6PD	H6PD	chr1:9294862 shneg	shCHD1	OK	15,3341	12,2047	-0,329313	-1,8448	0,00105	0,00607369	yes
RBM47	RBM47	RBM47	chr4:4042527 shneg	shCHD1	OK	27,7751	22,1042	-0,329471	-1,83109	0,00115	0,00656221	yes
KLHL4	KLHL4	KLHL4	chrX:8677271 shneg	shCHD1	OK	19,8266	15,7779	-0,329532	-1,54076	0,00765	0,0319703	yes
FLJ14186	FLJ14186	FLJ14186	chr4:1203266 shneg	shCHD1	OK	4,40047	3,50181	-0,329557	-1,56279	0,0068	0,028886	yes
XPR1	XPR1	XPR1	chr1:1806011 shneg	shCHD1	OK	16,2659	12,9434	-0,329631	-1,84278	0,00125	0,00702633	yes
MYO6	MYO6	MYO6	chr6:7645890 shneg	shCHD1	OK	8,80407	7,00472	-0,329843	-1,76394	0,00225	0,0115391	yes
SIX5	SIX5	SIX5	chr19:462680 shneg	shCHD1	OK	15,5472	12,3673	-0,330122	-1,71583	0,0033	0,0158557	yes
HEXB	HEXB	HEXB	chr5:7398096 shneg	shCHD1	OK	118,645	94,3578	-0,330437	-1,69957	0,0039	0,018217	yes
MCOLN3	MCOLN3	MCOLN3	chr1:8548376 shneg	shCHD1	OK	8,25154	6,56148	-0,330642	-1,57599	0,0067	0,0285134	yes
PRPS1	PRPS1	PRPS1	chrX:1068716 shneg	shCHD1	OK	52,5707	41,8029	-0,330658	-1,82226	0,00155	0,00838119	yes
SRD5A3	SRD5A3	SRD5A3	chr4:5621238 shneg	shCHD1	OK	5,35146	4,25336	-0,33133	-1,55419	0,00655	0,0279776	yes
ADAM10	ADAM10	ADAM10	chr15:588885 shneg	shCHD1	OK	58,0694	46,1488	-0,331484	-1,89102	0,0008	0,0048114	yes
CCDC6	CCDC6	CCDC6	chr10:615485 shneg	shCHD1	OK	9,62364	7,64717	-0,331658	-1,74716	0,0023	0,0117566	yes
PLXNB3	PLXNB3	PLXNB3	chrX:1530296 shneg	shCHD1	OK	4,25373	3,38005	-0,331683	-1,6101	0,00525	0,0232593	yes
RABGGTB	RABGGTB	RABGGTB	chr1:7625188 shneg	shCHD1	OK	23,1342	18,3826	-0,33169	-1,64168	0,0035	0,0166331	yes
BTC	BTC	BTC	chr4:7567144 shneg	shCHD1	OK	11,8186	9,39068	-0,331756	-1,56352	0,00675	0,0286999	yes
FN3K	FN3K	FN3K	chr17:806934 shneg	shCHD1	OK	18,5686	14,7534	-0,331814	-1,58161	0,0059	0,0257621	yes
SUMO2	SUMO2	SUMO2	chr17:731638 shneg	shCHD1	OK	264,735	210,339	-0,331832	-1,90599	0,00115	0,00656221	yes
SP5	SP5	SP5	chr2:1715718 shneg	shCHD1	OK	9,90504	7,86791	-0,332183	-1,60752	0,00535	0,0236722	yes
SH3RF2	SH3RF2	SH3RF2	chr5:1453161 shneg	shCHD1	OK	10,1995	8,10171	-0,332206	-1,63885	0,00405	0,0187979	yes
SLC35B2	SLC35B2	SLC35B2	chr6:4422183 shneg	shCHD1	OK	96,6997	76,7881	-0,332628	-1,88909	0,00075	0,00455593	yes
ZMYND8	ZMYND8	ZMYND8	chr20:458383 shneg	shCHD1	OK	64,3523	51,0831	-0,333145	-1,92282	0,0006	0,00377139	yes
MALAT1	MALAT1	MALAT1	chr11:652652 shneg	shCHD1	OK	154,725	122,817	-0,333201	-2,00229	0,00045	0,00293585	yes
LCLAT1	LCLAT1	LCLAT1	chr2:3067012 shneg	shCHD1	OK	4,33933	3,44417	-0,333316	-1,58607	0,0057	0,0250775	yes
AP2A2	AP2A2	AP2A2	chr11:925808 shneg	shCHD1	OK	42,3122	33,5813	-0,333414	-1,89159	0,00115	0,00656221	yes
UBL3	UBL3	UBL3	chr13:303385 shneg	shCHD1	OK	2,78496	2,21	-0,333609	-1,50432	0,0098	0,0392479	yes
TAOK1	TAOK1	TAOK1	chr17:277179 shneg	shCHD1	OK	15,8163	12,5464	-0,334141	-1,88849	0,0007	0,00428773	yes
C5orf51	C5orf51	C5orf51	chr5:4190446 shneg	shCHD1	OK	9,21169	7,30688	-0,334211	-1,73207	0,0026	0,0129897	yes
FAM210B	FAM210B	FAM210B	chr20:549339 shneg	shCHD1	OK	11,6911	9,2724	-0,334391	-1,67689	0,00315	0,015272	yes
TFEB	TFEB	TFEB	chr6:4165171 shneg	shCHD1	OK	6,25612	4,96007	-0,334907	-1,54957	0,00735	0,0308368	yes
XRN1	XRN1	XRN1	chr3:1420254 shneg	shCHD1	OK	4,32607	3,42822	-0,335597	-1,69951	0,00365	0,0172576	yes
DPYD	DPYD	DPYD	chr1:9754329 shneg	shCHD1	OK	53,433	42,3398	-0,335719	-1,85673	0,0011	0,0063157	yes
PGRMC2	PGRMC2	PGRMC2	chr4:1291903 shneg	shCHD1	OK	14,2484	11,2884	-0,33596	-1,76051	0,0019	0,00996052	yes
MAB21L3	MAB21L3	MAB21L3	chr1:1166543 shneg	shCHD1	OK	16,0551	12,7196	-0,335974	-1,74034	0,00295	0,0144329	yes
DIRAS2	DIRAS2	DIRAS2	chr9:9337211 shneg	shCHD1	OK	4,84158	3,83457	-0,336415	-1,64133	0,00365	0,0172576	yes
CSNK1D	CSNK1D	CSNK1D	chr17:802005 shneg	shCHD1	OK	53,4922	42,3606	-0,336605	-1,92	0,0009	0,00533451	yes
ZNF621	ZNF621	ZNF621	chr3:4056637 shneg	shCHD1	OK	6,70008	5,30495	-0,336839	-1,76258	0,0025	0,012594	yes
LOC729987	LOC729987	LOC729987	chr1:9867626 shneg	shCHD1	OK	4,57123	3,61884	-0,337058	-1,42088	0,0129	0,049027	yes
C20orf3	C20orf3	C20orf3	chr20:249435 shneg	shCHD1	OK	46,6787	36,9528	-0,33708	-1,84598	0,0015	0,00814879	yes
IFIH1	IFIH1	IFIH1	chr2:1631235 shneg	shCHD1	OK	8,39271	6,64371	-0,337146	-1,64486	0,00385	0,0180379	yes
ALS2CR8	ALS2CR8	ALS2CR8	chr2:2037769 shneg	shCHD1	OK	3,01689	2,38775	-0,337407	-1,6167	0,00505	0,0226106	yes
COL5A2	COL5A2	COL5A2	chr2:1898966 shneg	shCHD1	OK	99,9704	79,1216	-0,33743	-2,01365	0,0005	0,00321686	yes
AKAP13	AKAP13	AKAP13	chr15:859238 shneg	shCHD1	OK	12,8062	10,1352	-0,33747	-1,79996	0,00175	0,00931439	yes
R3HDM2	R3HDM2	R3HDM2	chr12:576475 shneg	shCHD1	OK	29,1549	23,0571	-0,338529	-1,8712	0,00105	0,00607369	yes
B7H6	B7H6	B7H6	chr11:173733 shneg	shCHD1	OK	3,52049	2,78347	-0,33889	-1,60814	0,00505	0,0226106	yes
CCDC165	CCDC165	CCDC165	chr18:871736 shneg	shCHD1	OK	18,1577	14,354	-0,339132	-1,87242	0,0009	0,00533451	yes
TTC17	TTC17	TTC17	chr11:433804 shneg	shCHD1	OK	20,0283	15,8309	-0,339299	-1,8469	0,00115	0,00656221	yes
RBPJ	RBPJ	RBPJ	chr4:2632133 shneg	shCHD1	OK	11,2074	8,85846	-0,339325	-1,80378	0,0018	0,00951868	yes
PNPLA6	PNPLA6	PNPLA6	chr19:759903 shneg	shCHD1	OK	39,5054	31,2232	-0,339429	-1,91485	0,0011	0,0063157	yes
ELF1	ELF1	ELF1	chr13:415060 shneg	shCHD1	OK	10,7665	8,50883	-0,339523	-1,7099	0,00285	0,0140225	yes
SLC6A8	SLC6A8	SLC6A8	chrX:1529537 shneg	shCHD1	OK	24,1231	19,0626	-0,339666	-1,78532	0,0018	0,00951868	yes
LOC10050562	LOC10050562	LOC10050562	chr19:426367 shneg	shCHD1	OK	19,8594	15,6928	-0,339723	-1,44366	0,0123	0,0472104	yes
MT1L	MT1L	MT1L	chr16:566513 shneg	shCHD1	OK	131,271	103,725	-0,339783	-1,68173	0,0039	0,018217	yes
DCTD	DCTD	DCTD	chr4:1838112 shneg	shCHD1	OK	41,1035	32,4776	-0,339817	-1,83202	0,0019	0,00996052	yes
NFAT5	NFAT5	NFAT5	chr16:695998 shneg	shCHD1	OK	44,6281	35,2429	-0,34062	-1,83195	0,00135	0,00748275	yes

SLC10A3	SLC10A3	SLC10A3	chrX:1537156	shneg	shCHD1	OK	22,7523	17,9613	-0,341124	-1,69115	0,00275	0,0136364	yes
CHM	CHM	CHM	chrX:8511618	shneg	shCHD1	OK	12,1933	9,62516	-0,341204	-1,73954	0,0021	0,0108578	yes
SDF4	SDF4	SDF4	chr1:1152287	shneg	shCHD1	OK	81,0151	63,9496	-0,341256	-1,92588	0,00095	0,00559279	yes
PYCARD	PYCARD	PYCARD	chr16:312128	shneg	shCHD1	OK	83,1134	65,6047	-0,341282	-1,76044	0,00255	0,0127904	yes
SDSL	SDSL	SDSL	chr12:113860	shneg	shCHD1	OK	14,2134	11,2185	-0,34137	-1,58647	0,005	0,0224661	yes
CBR3	CBR3	CBR3	chr21:375040	shneg	shCHD1	OK	12,5546	9,9089	-0,341424	-1,45961	0,01255	0,0479717	yes
SYNJ2	SYNJ2	SYNJ2	chr6:1584028	shneg	shCHD1	OK	64,86	51,1823	-0,341684	-2,02802	0,00025	0,00176854	yes
MOB3C	MOB3C	MOB3C	chr1:4707338	shneg	shCHD1	OK	5,21612	4,11364	-0,342564	-1,63069	0,0042	0,0193522	yes
MRPS30	MRPS30	MRPS30	chr5:4480902	shneg	shCHD1	OK	28,2928	22,3096	-0,342774	-1,76089	0,0027	0,0134267	yes
TMEM199	TMEM199	TMEM199	chr17:266846	shneg	shCHD1	OK	14,3681	11,329	-0,342842	-1,65546	0,00495	0,022299	yes
RAB3D	RAB3D	RAB3D	chr19:114068	shneg	shCHD1	OK	9,96058	7,85208	-0,343156	-1,75839	0,00225	0,0115391	yes
TOR2A	TOR2A	TOR2A	chr9:1304783	shneg	shCHD1	OK	13,3432	10,5176	-0,343307	-1,49419	0,00865	0,0353954	yes
AMZ2	AMZ2	AMZ2	chr17:662441	shneg	shCHD1	OK	28,606	22,5473	-0,343368	-1,60616	0,00505	0,0226106	yes
GIT1	GIT1	GIT1	chr17:279004	shneg	shCHD1	OK	39,512	31,1319	-0,343895	-1,92066	0,00115	0,00656221	yes
ZNF780B	ZNF780B	ZNF780B	chr19:405341	shneg	shCHD1	OK	4,08801	3,22089	-0,343938	-1,73379	0,0023	0,0117566	yes
F12	F12	F12	chr5:1768291	shneg	shCHD1	OK	16,7885	13,2265	-0,344043	-1,70779	0,0038	0,0178336	yes
TMUB2	TMUB2	TMUB2	chr17:422643	shneg	shCHD1	OK	16,6327	13,0961	-0,344885	-1,69017	0,00335	0,0160296	yes
MORN4	MORN4	MORN4	chr10:993743	shneg	shCHD1	OK	7,0007	5,51188	-0,344955	-1,66688	0,0042	0,0193522	yes
VPS45	VPS45	VPS45	chr1:1500393	shneg	shCHD1	OK	9,88725	7,78404	-0,345049	-1,67337	0,00465	0,0210977	yes
PLD3	PLD3	PLD3	chr19:408269	shneg	shCHD1	OK	49,1563	38,6965	-0,345172	-1,78306	0,0016	0,00862477	yes
LPCAT4	LPCAT4	LPCAT4	chr15:346510	shneg	shCHD1	OK	25,3375	19,9451	-0,345244	-1,78226	0,0021	0,0108578	yes
RHBDF1	RHBDF1	RHBDF1	chr16:108057	shneg	shCHD1	OK	8,71658	6,86124	-0,345293	-1,66079	0,00475	0,0214814	yes
PDK2	PDK2	PDK2	chr17:481721	shneg	shCHD1	OK	17,4358	13,7219	-0,345575	-1,47737	0,0101	0,0401378	yes
SENP7	SENP7	SENP7	chr3:1010431	shneg	shCHD1	OK	4,27917	3,36765	-0,345586	-1,61995	0,00575	0,0252576	yes
FAM208B	FAM208B	FAM208B	chr10:572680	shneg	shCHD1	OK	14,5139	11,4181	-0,346105	-1,91414	0,0007	0,00428773	yes
ZDHHC17	ZDHHC17	ZDHHC17	chr12:771578	shneg	shCHD1	OK	11,9559	9,40182	-0,346715	-1,8201	0,00165	0,00886	yes
RPL9	RPL9	RPL9	chr4:3945574	shneg	shCHD1	OK	1425,45	1120,89	-0,346775	-2,07634	0,0002	0,00144867	yes
SERGEF	SERGEF	SERGEF	chr11:178095	shneg	shCHD1	OK	13,7328	10,7984	-0,346812	-1,62265	0,0045	0,0205241	yes
TNFRSF12A	TNFRSF12A	TNFRSF12A	chr16:307031	shneg	shCHD1	OK	61,3631	48,2406	-0,347122	-1,81686	0,0008	0,0048114	yes
XAF1	XAF1	XAF1	chr17:665915	shneg	shCHD1	OK	3,91176	3,07501	-0,347226	-1,62444	0,0051	0,0228051	yes
SCNN1A	SCNN1A	SCNN1A	chr12:645600	shneg	shCHD1	OK	136,093	106,923	-0,348021	-2,02808	0,00045	0,00293585	yes
LOC10028901	LOC10028901	LOC10028901	chr9:1308734	shneg	shCHD1	OK	15,9296	12,5138	-0,348194	-1,84058	0,00175	0,00931439	yes
SOCS2	SOCS2	SOCS2	chr12:939594	shneg	shCHD1	OK	23,3324	18,3264	-0,348417	-1,7569	0,0022	0,011316	yes
FADS3	FADS3	FADS3	chr11:616409	shneg	shCHD1	OK	18,9239	14,8596	-0,348815	-1,72181	0,0024	0,0121606	yes
HES2	HES2	HES2	chr1:6475293	shneg	shCHD1	OK	2,61831	2,05591	-0,348858	-1,52898	0,0083	0,0341639	yes
PHLDB1	PHLDB1	PHLDB1	chr11:118477	shneg	shCHD1	OK	56,5738	44,4142	-0,349113	-2,00775	0,0004	0,00266202	yes
PYCR1	PYCR1	PYCR1	chr17:798902	shneg	shCHD1	OK	42,8042	33,6031	-0,349157	-1,8871	0,0012	0,00680293	yes
CREBL2	CREBL2	CREBL2	chr12:127648	shneg	shCHD1	OK	12,8383	10,0737	-0,349867	-1,81332	0,00125	0,00702633	yes
KIAA0195	KIAA0195	KIAA0195	chr17:734526	shneg	shCHD1	OK	11,7978	9,25586	-0,350081	-1,52229	0,0071	0,0299412	yes
JUNB	JUNB	JUNB	chr19:129023	shneg	shCHD1	OK	98,0253	76,8799	-0,350548	-1,95678	0,0006	0,00377139	yes
ZNF385A	ZNF385A	ZNF385A	chr12:547629	shneg	shCHD1	OK	11,5306	9,04086	-0,350938	-1,66865	0,00395	0,0184012	yes
KIAA0182	KIAA0182	KIAA0182	chr16:856450	shneg	shCHD1	OK	26,3204	20,6365	-0,35098	-1,99339	0,0004	0,00266202	yes
DCAF11	DCAF11	DCAF11	chr14:245839	shneg	shCHD1	OK	17,8572	13,9995	-0,35113	-1,85334	0,00105	0,00607369	yes
TPPP	TPPP	TPPP	chr5:659976-(	shneg	shCHD1	OK	8,66391	6,79147	-0,351295	-1,83843	0,00125	0,00702633	yes
LOC146880	LOC146880	LOC146880	chr17:627457	shneg	shCHD1	OK	25,5451	20,0239	-0,351321	-1,88288	0,0013	0,00726051	yes
POLR3GL	POLR3GL	POLR3GL	chr1:1454562	shneg	shCHD1	OK	16,0089	12,5482	-0,351393	-1,6929	0,00305	0,0148336	yes
XYLT2	XYLT2	XYLT2	chr17:484233	shneg	shCHD1	OK	9,71449	7,61416	-0,351454	-1,75728	0,002	0,0104181	yes
ZNF841	ZNF841	ZNF841	chr19:525677	shneg	shCHD1	OK	4,96099	3,88796	-0,351613	-1,66303	0,00405	0,0187979	yes
CLTC	CLTC	CLTC	chr17:576970	shneg	shCHD1	OK	109,72	85,9859	-0,351652	-2,11066	0,00025	0,00176854	yes
LPPR2	LPPR2	LPPR2	chr19:114660	shneg	shCHD1	OK	11,7464	9,20353	-0,351954	-1,7222	0,00325	0,0156858	yes
PRSS23	PRSS23	PRSS23	chr11:865114	shneg	shCHD1	OK	141,9	111,173	-0,352069	-2,08468	0,00025	0,00176854	yes
SPAG9	SPAG9	SPAG9	chr17:490395	shneg	shCHD1	OK	28,8652	22,612	-0,352243	-2,02377	0,00035	0,00236888	yes
WNT5A	WNT5A	WNT5A	chr3:5549974	shneg	shCHD1	OK	19,7417	15,4629	-0,352437	-1,92933	0,0007	0,00428773	yes
TJAP1	TJAP1	TJAP1	chr6:4344526	shneg	shCHD1	OK	23,3136	18,2527	-0,353063	-1,86345	0,0018	0,00951868	yes
UFL1	UFL1	UFL1	chr6:9696970	shneg	shCHD1	OK	7,89461	6,17998	-0,353266	-1,76073	0,0025	0,012594	yes
PXMP4	PXMP4	PXMP4	chr20:322905	shneg	shCHD1	OK	3,10236	2,42818	-0,353489	-1,68571	0,0039	0,018217	yes
PEX12	PEX12	PEX12	chr17:339018	shneg	shCHD1	OK	6,45104	5,04842	-0,353699	-1,68871	0,0037	0,0174466	yes
MT1M	MT1M	MT1M	chr16:566665	shneg	shCHD1	OK	83,9618	65,7016	-0,353805	-1,68121	0,0044	0,0201471	yes
PNPO	PNPO	PNPO	chr17:460188	shneg	shCHD1	OK	9,09707	7,11796	-0,353937	-1,75164	0,003	0,0146313	yes
CHMP3	CHMP3	CHMP3	chr2:8673055	shneg	shCHD1	OK	36,0282	28,183	-0,354299	-1,48515	0,0104	0,0410955	yes
SSH3	SSH3	SSH3	chr11:670709	shneg	shCHD1	OK	19,2661	15,064	-0,354958	-1,85811	0,0014	0,00768951	yes
PHF3	PHF3	PHF3	chr6:6435643	shneg	shCHD1	OK	16,852	13,1747	-0,355151	-1,95754	0,0007	0,00428773	yes
ANKRD49	ANKRD49	ANKRD49	chr11:942271	shneg	shCHD1	OK	6,53792	5,11124	-0,355157	-1,57879	0,00635	0,0272991	yes
LANCL1	LANCL1	LANCL1	chr2:2112959	shneg	shCHD1	OK	14,1438	11,0561	-0,355329	-1,8777	0,00055	0,00350455	yes
TRMT1L	TRMT1L	TRMT1L	chr1:1850872	shneg	shCHD1	OK	5,63298	4,40316	-0,35536	-1,56698	0,0068	0,028886	yes
TRIM21	TRIM21	TRIM21	chr11:440612	shneg	shCHD1	OK	15,619	12,2087	-0,3554	-1,73697	0,00325	0,0156858	yes
LOC645638	LOC645638	LOC645638	chr17:581609	shneg	shCHD1	OK	392,448	306,72	-0,35558	-2,00754	0,0006	0,00377139	yes
MYH15	MYH15	MYH15	chr3:1080992	shneg	shCHD1	OK	65,9442	51,5361	-0,355663	-2,08558	0,00025	0,00176854	yes
COQ2	COQ2	COQ2	chr4:8418497	shneg	shCHD1	OK	7,56211	5,90959	-0,35573	-1,57027	0,00695	0,0293888	yes
IGBP1	IGBP1	IGBP1	chrX:6935331	shneg	shCHD1	OK	59,983	46,8585	-0,356242	-1,93674	0,00085	0,00508358	yes
PIK3CA	PIK3CA	PIK3CA	chr3:1788663	shneg	shCHD1	OK	5,60962	4,3817	-0,35641	-1,69198	0,00305	0,0148336	yes
PRKAG2	PRKAG2	PRKAG2	chr7:1512532	shneg	shCHD1	OK	110,506	86,3097	-0,356527	-2,02922	0,00025	0,00176854	yes
NFXL1	NFXL1	NFXL1	chr4:4784925	shneg	shCHD1	OK	6,08157	4,74923	-0,35675	-1,70715	0,0025	0,012594	yes
IRF1	IRF1	IRF1	chr5:1318173	shneg	shCHD1	OK	23,7661	18,5576	-0,356895	-1,93227	0,00105	0,00607369	yes
PDCD6	PDCD6	PDCD6	chr5:271735-4	shneg	shCHD1	OK	219,512	171,375	-0,357144	-1,86369	0,00085	0,00508358	yes
PLCH1	PLCH1	PLCH1	chr3:1551976	shneg	shCHD1	OK	2,69482	2,10385	-0,357161	-1,67357	0,00385	0,0180379	yes
LRPAP1	LRPAP1	LRPAP1	chr4:3505323	shneg	shCHD1	OK	6,09672	4,75936	-0,357267	-1,90218	0,0014	0,00768951	yes
LMAN2L	LMAN2L	LMAN2L	chr2:9737166	shneg	shCHD1	OK	11,9902	9,35994	-0,357285	-1,72673	0,00225	0,0115391	yes
BOD1L	BOD1L	BOD1L	chr4:1357036	shneg	shCHD1	OK	8,60177	6,71336	-0,357599	-1,94244	0,0007	0,00428773	yes
MUT	MUT	MUT	chr6:4939807	shneg	shCHD1	OK	14,4967	11,3138	-0,357638	-1,87869	0,00055	0,00350455	yes
EEF1D	EEF1D	EEF1D	chr8:1446618	shneg	shCHD1	OK	140,276	109,475	-0,357661	-1,91464	0,00115	0,00656221	yes
SOCS5	SOCS5	SOCS5	chr2:4692609	shneg	shCHD1	OK	10,285	8,02627	-0,35774	-1,86237	0,00125	0,00702633	yes
GAS5	GAS5	GAS5	chr1:1738323	shneg	shCHD1	OK	557,355	434,936	-0,357793	-2,03545	0,00065	0,0040329	yes
FRYL	FRYL	FRYL	chr4:4849937	shneg	shCHD1	OK	11,477	8,95588	-0,357837	-1,98259	0,0003	0,0020738	yes
TMEM150A	TMEM150A	TMEM150A	chr2:8582566	shneg	shCHD1	OK	11,4596	8,93876	-0,358415	-1,70215	0,0026	0,0129897	yes
RAB22A	RAB22A	RAB22A	chr20:568847	shneg	shCHD1	OK	4,30474	3,35747	-0,358551	-1,81294	0,0019	0,00996052	yes
PDXK	PDXK	PDXK	chr21:451389	shneg	shCHD1	OK	19,3785	15,1134	-0,358623	-2,00186	0,0005	0,00321686	yes
ABTB2	ABTB2	ABTB2	chr11:341725	shneg	shCHD1	OK	8,60049	6,70629	-0,358904	-1,83775	0,0015	0,00814879	yes
IBTK	IBTK	IBTK	chr6:8287995	shneg	shCHD1	OK	23,746	18,5135	-0,359112	-1,99982	0,0006	0,00377139	yes
FNIP2	FNIP2	FNIP2	chr4:1596901	shneg	shCHD1	OK	1,83057	1,42686	-0,359451	-1,5531	0,00715	0,0300974	yes
UBA6	UBA6	UBA6	chr4:6848147	shneg	shCHD1	OK	9,59723	7,4805	-0,359484	-1,90301	0,00095	0,00559279	yes
ZNF160	ZNF160	ZNF160	chr19:535698	shneg	shCHD1	OK	8,09578	6,30814	-0,359956	-1,82283	0,0014	0,00768951	yes
DNAJC22	DNAJC22	DNAJC22	chr12:497410	shneg	shCHD1	OK	12,7017	9,89617	-0,360083	-1,74301	0,00255	0,0127904	yes

ERO1LB	ERO1LB	ERO1LB	chr1:2363784	shneg	shCHD1	OK	19,3115	15,045	-0,360178	-1,96879	0,00075	0,00455593	yes
IRAK1	IRAK1	IRAK1	chrX:1532759	shneg	shCHD1	OK	100,096	77,9567	-0,360634	-2,08194	0,00025	0,00176854	yes
C10orf118	C10orf118	C10orf118	chr10:115881	shneg	shCHD1	OK	4,12141	3,20977	-0,360667	-1,71689	0,0026	0,0129897	yes
C11orf67	C11orf67	C11orf67	chr11:775322	shneg	shCHD1	OK	28,6884	22,3406	-0,3608	-1,47688	0,0109	0,0427438	yes
GUCY1B2	GUCY1B2	GUCY1B2	chr13:515686	shneg	shCHD1	OK	5,1498	4,00986	-0,360966	-1,73703	0,00295	0,0144329	yes
PHKB	PHKB	PHKB	chr16:474952	shneg	shCHD1	OK	10,7643	8,37874	-0,361445	-1,90773	0,0007	0,00428773	yes
AP2B1	AP2B1	AP2B1	chr17:339142	shneg	shCHD1	OK	127,392	99,1353	-0,361808	-2,1679	0,00015	0,00112331	yes
TRPM4	TRPM4	TRPM4	chr19:496610	shneg	shCHD1	OK	9,20737	7,16489	-0,361844	-1,82399	0,00155	0,00838119	yes
LOC652276	LOC652276	LOC652276	chr16:265338	shneg	shCHD1	OK	4,95299	3,85418	-0,361874	-1,66423	0,00335	0,0160296	yes
ATRN	ATRN	ATRN	chr20:345166	shneg	shCHD1	OK	7,00261	5,44897	-0,36191	-1,64475	0,004	0,018603	yes
TMTC3	TMTC3	TMTC3	chr12:885360	shneg	shCHD1	OK	5,40138	4,20292	-0,361937	-1,81842	0,0018	0,00951868	yes
GPRC5B	GPRC5B	GPRC5B	chr16:198702	shneg	shCHD1	OK	21,0301	16,3587	-0,362394	-1,90736	0,001	0,0058183	yes
GABBR1	GABBR1	GABBR1	chr6_qbl_hap	shneg	shCHD1	OK	5,81479	4,52298	-0,362453	-1,73117	0,0026	0,0129897	yes
CASP6	CASP6	CASP6	chr4:1106097	shneg	shCHD1	OK	11,073	8,61124	-0,36276	-1,71546	0,002	0,0104181	yes
OCIAD1	OCIAD1	OCIAD1	chr4:4883305	shneg	shCHD1	OK	54,3161	42,2315	-0,363063	-1,99287	0,0007	0,00428773	yes
ZNF446	ZNF446	ZNF446	chr19:589877	shneg	shCHD1	OK	10,1139	7,8635	-0,363089	-1,74637	0,00255	0,0127904	yes
IMPACT	IMPACT	IMPACT	chr18:220066	shneg	shCHD1	OK	6,19673	4,81791	-0,363099	-1,73547	0,00215	0,0110834	yes
LIN37	LIN37	LIN37	chr19:362392	shneg	shCHD1	OK	8,59249	6,67901	-0,363441	-1,45177	0,01235	0,0473762	yes
MON2	MON2	MON2	chr12:628605	shneg	shCHD1	OK	8,63136	6,70746	-0,363821	-1,97129	0,00055	0,00350455	yes
TMEM184C	TMEM184C	TMEM184C	chr4:1485385	shneg	shCHD1	OK	9,89885	7,69109	-0,364074	-1,78136	0,002	0,0104181	yes
PHLDA3	PHLDA3	PHLDA3	chr1:2014346	shneg	shCHD1	OK	428,593	332,937	-0,364362	-2,15585	0,0002	0,00144867	yes
CAPG	CAPG	CAPG	chr2:8562187	shneg	shCHD1	OK	130,399	101,276	-0,364639	-2,04415	0,00035	0,00236888	yes
THSD4	THSD4	THSD4	chr15:714337	shneg	shCHD1	OK	51,9986	40,3853	-0,364643	-2,15056	0,00015	0,00112331	yes
ST3GAL5	ST3GAL5	ST3GAL5	chr2:8606627	shneg	shCHD1	OK	20,492	15,909	-0,365218	-1,86797	0,0009	0,00533451	yes
LRRC37A4	LRRC37A4	LRRC37A4	chr17_ctg5_h	shneg	shCHD1	OK	5,5596	4,3158	-0,365352	-1,86536	0,001	0,0058183	yes
FAM21C	FAM21C	FAM21C	chr10:462226	shneg	shCHD1	OK	4,73185	3,67235	-0,3657	-1,72838	0,0033	0,0158557	yes
C6orf1	C6orf1	C6orf1	chr6:3421415	shneg	shCHD1	OK	49,6687	38,5428	-0,365878	-1,79824	0,00225	0,0115391	yes
AGA	AGA	AGA	chr4:1783519	shneg	shCHD1	OK	12,2676	9,51879	-0,366	-1,75637	0,00205	0,0106507	yes
ZNF430	ZNF430	ZNF430	chr19:212034	shneg	shCHD1	OK	2,71795	2,10885	-0,366063	-1,57544	0,0054	0,0238631	yes
GDI1	GDI1	GDI1	chrX:1536652	shneg	shCHD1	OK	101,379	78,6469	-0,366294	-2,10218	0,00025	0,00176854	yes
UBE2H	UBE2H	UBE2H	chr7:1294705	shneg	shCHD1	OK	42,2822	32,7935	-0,366641	-2,0791	0,00035	0,00236888	yes
CHD9	CHD9	CHD9	chr16:530889	shneg	shCHD1	OK	7,43942	5,7681	-0,367094	-1,96533	0,0006	0,00377139	yes
ZNF558	ZNF558	ZNF558	chr19:892038	shneg	shCHD1	OK	6,75044	5,23256	-0,367463	-1,78881	0,0022	0,011316	yes
PTPLB	PTPLB	PTPLB	chr3:1232133	shneg	shCHD1	OK	13,7577	10,6642	-0,367465	-1,73496	0,00245	0,0123735	yes
RPS3A	RPS3A	RPS3A	chr4:1520207	shneg	shCHD1	OK	1728,77	1339,47	-0,368082	-2,21811	0,0001	0,00078473	yes
GLIS2	GLIS2	GLIS2	chr16:438222	shneg	shCHD1	OK	11,3277	8,77575	-0,36826	-1,88255	0,0012	0,00680293	yes
RBM20	RBM20	RBM20	chr10:112404	shneg	shCHD1	OK	29,4695	22,8293	-0,368334	-2,10641	0,0003	0,0020738	yes
DOCK6	DOCK6	DOCK6	chr19:113099	shneg	shCHD1	OK	5,89249	4,56452	-0,368413	-1,86201	0,0009	0,00533451	yes
CYP3A5	CYP3A5	CYP3A5	chr7:9924581	shneg	shCHD1	OK	250,286	193,844	-0,368683	-1,95034	0,0006	0,00377139	yes
DSTNP2	DSTNP2	DSTNP2	chr12:699384	shneg	shCHD1	OK	15,007	11,6178	-0,369299	-1,73629	0,00395	0,0184012	yes
ASCC3	ASCC3	ASCC3	chr6:1009566	shneg	shCHD1	OK	8,71254	6,74217	-0,36988	-1,87368	0,0014	0,00768951	yes
REEP3	REEP3	REEP3	chr10:652811	shneg	shCHD1	OK	13,0573	10,1041	-0,369908	-1,97092	0,00045	0,00293585	yes
CRYL1	CRYL1	CRYL1	chr13:209778	shneg	shCHD1	OK	22,3036	17,2552	-0,370247	-1,82448	0,00165	0,00886	yes
ZNF524	ZNF524	ZNF524	chr19:561117	shneg	shCHD1	OK	19,0426	14,7317	-0,370314	-1,78724	0,00155	0,00838119	yes
TTC33	TTC33	TTC33	chr5:4071167	shneg	shCHD1	OK	5,69252	4,40287	-0,370623	-1,82769	0,002	0,0104181	yes
C6orf89	C6orf89	C6orf89	chr6:3685363	shneg	shCHD1	OK	18,943	14,6498	-0,370777	-2,0633	0,0003	0,0020738	yes
ABCA2	ABCA2	ABCA2	chr9:1399016	shneg	shCHD1	OK	28,2777	21,86	-0,371367	-2,11326	0,00035	0,00236888	yes
SNAPC2	SNAPC2	SNAPC2	chr19:798519	shneg	shCHD1	OK	15,7027	12,1376	-0,371525	-1,76622	0,0024	0,0121606	yes
DDX26B	DDX26B	DDX26B	chrX:1346545	shneg	shCHD1	OK	9,09392	7,02882	-0,37162	-1,86221	0,0012	0,00680293	yes
COQ10A	COQ10A	COQ10A	chr12:566606	shneg	shCHD1	OK	15,1083	11,6773	-0,371641	-1,76486	0,00245	0,0123735	yes
SDF2	SDF2	SDF2	chr17:269753	shneg	shCHD1	OK	26,4909	20,4688	-0,372071	-1,82875	0,00135	0,00748275	yes
SLC2A1	SLC2A1	SLC2A1	chr1:4339104	shneg	shCHD1	OK	11,9491	9,22787	-0,372826	-1,86857	0,001	0,0058183	yes
MFSD10	MFSD10	MFSD10	chr4:2932287	shneg	shCHD1	OK	37,7669	29,1632	-0,372972	-1,9692	0,0007	0,00428773	yes
MYOF	MYOF	MYOF	chr10:950661	shneg	shCHD1	OK	107,915	83,331	-0,372974	-2,23583	0,0001	0,00078473	yes
ZNF277	ZNF277	ZNF277	chr7:1118466	shneg	shCHD1	OK	11,5602	8,92622	-0,373048	-1,82756	0,00135	0,00748275	yes
GLO1	GLO1	GLO1	chr6:3864370	shneg	shCHD1	OK	154,051	118,943	-0,373145	-2,16017	0,0001	0,00078473	yes
NSDHL	NSDHL	NSDHL	chrX:1519995	shneg	shCHD1	OK	25,269	19,5096	-0,373182	-1,88063	0,0009	0,00533451	yes
SPATS2L	SPATS2L	SPATS2L	chr2:2011706	shneg	shCHD1	OK	40,6102	31,3516	-0,373303	-2,16396	0,00015	0,00112331	yes
TOP1	TOP1	TOP1	chr20:396574	shneg	shCHD1	OK	49,688	38,3554	-0,373469	-2,11698	0,0003	0,0020738	yes
NEU1	NEU1	NEU1	chr6_ssto_ha	shneg	shCHD1	OK	42,9036	33,1128	-0,373708	-2,01941	0,0004	0,00266202	yes
TRIM68	TRIM68	TRIM68	chr11:461990	shneg	shCHD1	OK	4,43856	3,4254	-0,373818	-1,78104	0,0021	0,0108578	yes
ASPH	ASPH	ASPH	chr8:6220052	shneg	shCHD1	OK	130,691	100,831	-0,374221	-2,15752	0,0001	0,00078473	yes
BAIAP2	BAIAP2	BAIAP2	chr17:790089	shneg	shCHD1	OK	11,1263	8,58352	-0,374332	-1,57097	0,00595	0,0259073	yes
NUFIP2	NUFIP2	NUFIP2	chr17:275828	shneg	shCHD1	OK	16,8863	13,0264	-0,374415	-2,11914	0,0002	0,00144867	yes
FDXR	FDXR	FDXR	chr17:728586	shneg	shCHD1	OK	19,6879	15,186	-0,374566	-1,87952	0,0011	0,0063157	yes
SUMF1	SUMF1	SUMF1	chr3:4402828	shneg	shCHD1	OK	13,2493	10,2196	-0,374579	-1,80891	0,0015	0,00814879	yes
SLK	SLK	SLK	chr10:105727	shneg	shCHD1	OK	57,9326	44,6831	-0,374647	-2,1541	0,00015	0,00112331	yes
EPN1	EPN1	EPN1	chr19:561865	shneg	shCHD1	OK	56,5027	43,5649	-0,375154	-2,09771	0,00025	0,00176854	yes
PPP2R5D	PPP2R5D	PPP2R5D	chr6:4295232	shneg	shCHD1	OK	38,6742	29,8148	-0,37534	-1,56762	0,00655	0,0279776	yes
SHC1	SHC1	SHC1	chr1:1549347	shneg	shCHD1	OK	300,024	231,268	-0,375511	-2,24526	5,00E-05	0,0004193	yes
MYO18A	MYO18A	MYO18A	chr17:274005	shneg	shCHD1	OK	6,5833	5,07462	-0,375513	-1,94319	0,0008	0,0048114	yes
PHLDB3	PHLDB3	PHLDB3	chr19:439792	shneg	shCHD1	OK	5,47226	4,21742	-0,375777	-1,72785	0,003	0,0146313	yes
TMEM62	TMEM62	TMEM62	chr15:434257	shneg	shCHD1	OK	4,01753	3,09549	-0,376143	-1,62954	0,00575	0,0252576	yes
ABCC9	ABCC9	ABCC9	chr12:219503	shneg	shCHD1	OK	1,56045	1,20213	-0,376375	-1,72463	0,00265	0,0132017	yes
ALKBH6	ALKBH6	ALKBH6	chr19:365000	shneg	shCHD1	OK	13,84	10,6601	-0,376617	-1,64002	0,0042	0,0193522	yes
NUMBL	NUMBL	NUMBL	chr19:411718	shneg	shCHD1	OK	13,5047	10,4018	-0,376635	-1,94518	0,0007	0,00428773	yes
TOM1L2	TOM1L2	TOM1L2	chr17:177468	shneg	shCHD1	OK	10,086	7,7684	-0,376668	-1,98867	0,00065	0,0040329	yes
PEPD	PEPD	PEPD	chr19:338778	shneg	shCHD1	OK	40,3435	31,073	-0,376674	-2,02468	0,0006	0,00377139	yes
PIGV	PIGV	PIGV	chr1:2711445	shneg	shCHD1	OK	6,35969	4,89761	-0,376878	-1,80669	0,0013	0,00726051	yes
HIAT1	HIAT1	HIAT1	chr1:1005037	shneg	shCHD1	OK	29,0134	22,3392	-0,377138	-2,02316	0,00045	0,00293585	yes
MAP7D3	MAP7D3	MAP7D3	chrX:1352953	shneg	shCHD1	OK	8,26978	6,36643	-0,377364	-1,89858	0,00095	0,00559279	yes
INTS3	INTS3	INTS3	chr1:1537005	shneg	shCHD1	OK	25,4756	19,6066	-0,377776	-2,08302	0,00045	0,00293585	yes
LOC10013125	LOC10013125	LOC10013125	chr7:7115400	shneg	shCHD1	OK	9,44588	7,26977	-0,377776	-2,14449	0,0001	0,00078473	yes
HNF1A	HNF1A	HNF1A	chr12:121416	shneg	shCHD1	OK	4,58699	3,52888	-0,378337	-1,7592	0,0025	0,012594	yes
COMMD8	COMMD8	COMMD8	chr4:4745281	shneg	shCHD1	OK	11,5951	8,91894	-0,378575	-1,79997	0,0015	0,00814879	yes
RNF215	RNF215	RNF215	chr22:307748	shneg	shCHD1	OK	16,562	12,7389	-0,378641	-1,85863	0,0018	0,00951868	yes
C9orf95	C9orf95	C9orf95	chr9:7767611	shneg	shCHD1	OK	18,2557	14,0413	-0,378668	-1,77582	0,00265	0,0132017	yes
SPECC1L	SPECC1L	SPECC1L	chr22:246667	shneg	shCHD1	OK	11,1628	8,58568	-0,378697	-2,0301	0,0004	0,00266202	yes
NRARP	NRARP	NRARP	chr9:1401940	shneg	shCHD1	OK	20,6126	15,8518	-0,378879	-1,9776	0,001	0,0058183	yes
LAGE3	LAGE3	LAGE3	chrX:1537052	shneg	shCHD1	OK	24,0596	18,5017	-0,378954	-1,92738	0,0011	0,0063157	yes
NADSYN1	NADSYN1	NADSYN1	chr11:711642	shneg	shCHD1	OK	15,4335	11,8664	-0,379184	-1,90891	0,00105	0,00607369	yes
SLC9B2	SLC9B2	SLC9B2	chr4:1039466	shneg	shCHD1	OK	4,20642	3,23384	-0,379346	-1,7788	0,0021	0,0108578	yes
MMP17	MMP17	MMP17	chr12:132312	shneg	shCHD1	OK	13,3581	10,2677	-0,379605	-1,86945	0,00185	0,00975716	yes

TRANK1	TRANK1	TRANK1	chr3:3686830 shneg	shCHD1	OK	8,46837	6,50817	-0,379832	-2,06294	0,0005	0,00321686	yes
ASTN2	ASTN2	ASTN2	chr9:1191875 shneg	shCHD1	OK	74,0369	56,8862	-0,380166	-1,87245	0,00095	0,00559279	yes
PLAT	PLAT	PLAT	chr8:4203223 shneg	shCHD1	OK	2,47527	1,90141	-0,380512	-1,46161	0,00985	0,0393915	yes
KLHL28	KLHL28	KLHL28	chr14:453935 shneg	shCHD1	OK	2,51928	1,93509	-0,380612	-1,80079	0,0012	0,00680293	yes
ALPK2	ALPK2	ALPK2	chr18:561484 shneg	shCHD1	OK	75,7257	58,1642	-0,380652	-2,25629	0,00025	0,00176854	yes
GCHFR	GCHFR	GCHFR	chr15:410562 shneg	shCHD1	OK	21,323	16,3724	-0,381145	-1,68013	0,00425	0,0195438	yes
CLOCK	CLOCK	CLOCK	chr4:5629865 shneg	shCHD1	OK	4,78541	3,67428	-0,381182	-1,8219	0,0017	0,00909693	yes
TEFM	TEFM	TEFM	chr17:292260 shneg	shCHD1	OK	8,05237	6,18268	-0,381182	-1,57945	0,00615	0,0265788	yes
MYO9A	MYO9A	MYO9A	chr15:721183 shneg	shCHD1	OK	4,16458	3,19691	-0,38149	-1,86899	0,00115	0,00656221	yes
KIFAP3	KIFAP3	KIFAP3	chr1:1698904 shneg	shCHD1	OK	8,08048	6,20212	-0,381679	-1,84437	0,0018	0,00951868	yes
PIP4K2C	PIP4K2C	PIP4K2C	chr12:579849 shneg	shCHD1	OK	25,5881	19,6382	-0,381805	-2,04982	0,00035	0,00236888	yes
TSPAN11	TSPAN11	TSPAN11	chr12:310798 shneg	shCHD1	OK	5,25716	4,03446	-0,381908	-1,87393	0,00135	0,00748275	yes
EPOR	EPOR	EPOR	chr19:114878 shneg	shCHD1	OK	16,0009	12,2783	-0,382041	-1,9176	0,0009	0,00533451	yes
ZNF227	ZNF227	ZNF227	chr19:447166 shneg	shCHD1	OK	6,03756	4,63113	-0,382604	-1,81747	0,0014	0,00768951	yes
PCYOX1	PCYOX1	PCYOX1	chr2:7048523 shneg	shCHD1	OK	20,0162	15,351	-0,382833	-2,10523	0,0002	0,00144867	yes
LAMTOR3	LAMTOR3	LAMTOR3	chr4:1007994 shneg	shCHD1	OK	6,89152	5,28525	-0,382852	-1,87216	0,0014	0,00768951	yes
CHP	CHP	CHP	chr15:415234 shneg	shCHD1	OK	27,2485	20,8957	-0,382972	-2,07584	0,00015	0,00112331	yes
CTSZ	CTSZ	CTSZ	chr20:575702 shneg	shCHD1	OK	64,3339	49,3264	-0,383218	-2,07901	0,0002	0,00144867	yes
ABCC2	ABCC2	ABCC2	chr10:101542 shneg	shCHD1	OK	54,2711	41,6092	-0,383282	-2,2127	0,0001	0,00078473	yes
NCEH1	NCEH1	NCEH1	chr3:1723484 shneg	shCHD1	OK	34,9724	26,8114	-0,38337	-2,15124	0,0003	0,0020738	yes
CPNE7	CPNE7	CPNE7	chr16:896421 shneg	shCHD1	OK	52,823	40,4662	-0,384451	-2,10917	0,0001	0,00078473	yes
SLC16A2	SLC16A2	SLC16A2	chrX:7364108 shneg	shCHD1	OK	12,9881	9,94809	-0,384696	-2,02397	0,00055	0,00350455	yes
C12orf35	C12orf35	C12orf35	chr12:321123 shneg	shCHD1	OK	12,0091	9,1955	-0,385127	-2,05218	0,00035	0,00236888	yes
BAZ2B	BAZ2B	BAZ2B	chr2:1601754 shneg	shCHD1	OK	7,28037	5,57367	-0,385383	-2,01674	0,00035	0,00236888	yes
AIG1	AIG1	AIG1	chr6:1433820 shneg	shCHD1	OK	27,1446	20,7809	-0,38541	-1,92378	0,0012	0,00680293	yes
EMB	EMB	EMB	chr5:4969203 shneg	shCHD1	OK	3,64859	2,79273	-0,385665	-1,82714	0,00165	0,00886	yes
SPINT1	SPINT1	SPINT1	chr15:411362 shneg	shCHD1	OK	8,8233	6,75082	-0,386254	-1,83439	0,0013	0,00726051	yes
GYLTL1B	GYLTL1B	GYLTL1B	chr11:459431 shneg	shCHD1	OK	8,9703	6,86253	-0,386415	-1,83518	0,0013	0,00726051	yes
TMEM51	TMEM51	TMEM51	chr1:1547902 shneg	shCHD1	OK	32,9489	25,2036	-0,386602	-2,01149	0,00035	0,00236888	yes
FAM102A	FAM102A	FAM102A	chr9:1307028 shneg	shCHD1	OK	17,27	13,2102	-0,386618	-2,06338	0,00035	0,00236888	yes
LOC10049917	LOC10049917	LOC10049917	chr4:8381460 shneg	shCHD1	OK	25,1913	19,2681	-0,386711	-1,93324	0,00065	0,0040329	yes
TMEM192	TMEM192	TMEM192	chr4:1659972 shneg	shCHD1	OK	5,79154	4,42885	-0,387013	-1,86688	0,00115	0,00656221	yes
HGF	HGF	HGF	chr7:8133144 shneg	shCHD1	OK	24,5462	18,7686	-0,387175	-1,72741	0,00305	0,0148336	yes
ZNF580	ZNF580	ZNF580	chr19:561523 shneg	shCHD1	OK	47,548	36,3434	-0,387691	-1,93001	0,001	0,0058183	yes
MORC4	MORC4	MORC4	chrX:1061839 shneg	shCHD1	OK	102,495	78,3193	-0,388117	-2,26644	5,00E-05	0,0004193	yes
KLHL20	KLHL20	KLHL20	chr1:1736840 shneg	shCHD1	OK	5,57182	4,25755	-0,388126	-1,83878	0,00075	0,00455593	yes
UTP3	UTP3	UTP3	chr4:7155419 shneg	shCHD1	OK	16,5322	12,6312	-0,388279	-1,92967	0,00075	0,00455593	yes
OAS2	OAS2	OAS2	chr12:113416 shneg	shCHD1	OK	84,6145	64,6341	-0,38861	-2,19561	5,00E-05	0,0004193	yes
MOSPD2	MOSPD2	MOSPD2	chrX:1489152 shneg	shCHD1	OK	8,97283	6,85398	-0,388622	-1,89214	0,0008	0,0048114	yes
WBP2	WBP2	WBP2	chr17:738417 shneg	shCHD1	OK	36,8105	28,1172	-0,388663	-2,0675	0,00045	0,00293585	yes
CISD2	CISD2	CISD2	chr4:1037901 shneg	shCHD1	OK	8,6793	6,62953	-0,388671	-1,90923	0,00105	0,00607369	yes
SEZ6L2	SEZ6L2	SEZ6L2	chr16:298824 shneg	shCHD1	OK	34,6811	26,4899	-0,388706	-2,13567	0,00015	0,00112331	yes
ZFP1	ZFP1	ZFP1	chr16:751824 shneg	shCHD1	OK	5,24117	4,00238	-0,389031	-1,85345	0,0008	0,0048114	yes
SNX16	SNX16	SNX16	chr8:8271181 shneg	shCHD1	OK	2,86599	2,18853	-0,389068	-1,5812	0,00625	0,0269607	yes
MMP14	MMP14	MMP14	chr14:233057 shneg	shCHD1	OK	272,162	207,823	-0,38911	-2,33514	5,00E-05	0,0004193	yes
SGMS1	SGMS1	SGMS1	chr10:520653 shneg	shCHD1	OK	5,29876	4,04584	-0,389215	-1,89124	0,00095	0,00559279	yes
SLC38A10	SLC38A10	SLC38A10	chr17:792187 shneg	shCHD1	OK	28,1002	21,4534	-0,389372	-2,11865	5,00E-05	0,0004193	yes
LRRC23	LRRC23	LRRC23	chr12:701389 shneg	shCHD1	OK	8,7719	6,69692	-0,389392	-1,65851	0,00335	0,0160296	yes
L1CAM	L1CAM	L1CAM	chrX:1531269 shneg	shCHD1	OK	29,9062	22,8299	-0,389525	-2,17818	0,0002	0,00144867	yes
TMCO1	TMCO1	TMCO1	chr1:1656935 shneg	shCHD1	OK	3,98496	3,04149	-0,389788	-1,84689	0,0014	0,00768951	yes
CNNM4	CNNM4	CNNM4	chr2:9742663 shneg	shCHD1	OK	8,19897	6,25714	-0,389939	-1,98166	0,00055	0,00350455	yes
KAT6B	KAT6B	KAT6B	chr10:765861 shneg	shCHD1	OK	6,72863	5,13493	-0,389969	-2,02654	0,0003	0,0020738	yes
ARL5B	ARL5B	ARL5B	chr10:189483 shneg	shCHD1	OK	4,71782	3,59936	-0,39038	-1,73064	0,0026	0,0129897	yes
RARG	RARG	RARG	chr12:536043 shneg	shCHD1	OK	27,2111	20,7577	-0,390545	-2,06958	0,0004	0,00266202	yes
MC1R	MC1R	MC1R	chr16:899842 shneg	shCHD1	OK	17,3648	13,2447	-0,39075	-2,03484	0,0007	0,00428773	yes
TSC22D4	TSC22D4	TSC22D4	chr7:1000641 shneg	shCHD1	OK	47,9033	36,537	-0,390768	-2,14292	0,0003	0,0020738	yes
PIGT	PIGT	PIGT	chr20:440447 shneg	shCHD1	OK	45,9708	35,0539	-0,391139	-2,14037	0,0001	0,00078473	yes
SLC38A7	SLC38A7	SLC38A7	chr16:587002 shneg	shCHD1	OK	8,87413	6,76495	-0,391527	-1,88505	0,00115	0,00656221	yes
CKMT1B	CKMT1B	CKMT1B	chr15:438852 shneg	shCHD1	OK	4,57668	3,48868	-0,391621	-1,49692	0,009	0,0365911	yes
KIAA0317	KIAA0317	KIAA0317	chr14:751279 shneg	shCHD1	OK	15,5952	11,8851	-0,391945	-2,12263	0,0002	0,00144867	yes
KPNA5	KPNA5	KPNA5	chr6:1170023 shneg	shCHD1	OK	4,47743	3,41017	-0,392829	-1,58865	0,0057	0,0250775	yes
MAN2B1	MAN2B1	MAN2B1	chr19:127573 shneg	shCHD1	OK	25,5612	19,4601	-0,393432	-2,12391	0,0002	0,00144867	yes
DAZAP2	DAZAP2	DAZAP2	chr12:516325 shneg	shCHD1	OK	86,4518	65,7927	-0,393968	-2,01648	0,00045	0,00293585	yes
ZBTB26	ZBTB26	ZBTB26	chr9:1256803 shneg	shCHD1	OK	6,63288	5,04756	-0,39405	-1,75247	0,00225	0,0115391	yes
ADSSL1	ADSSL1	ADSSL1	chr14:105190 shneg	shCHD1	OK	8,17079	6,21786	-0,394059	-1,82516	0,0012	0,00680293	yes
ZNF792	ZNF792	ZNF792	chr19:354472 shneg	shCHD1	OK	1,95554	1,48809	-0,394111	-1,50556	0,01095	0,0428555	yes
SEMA3C	SEMA3C	SEMA3C	chr7:8037185 shneg	shCHD1	OK	205,098	156,046	-0,39434	-2,36116	5,00E-05	0,0004193	yes
ZNF292	ZNF292	ZNF292	chr6:8786526 shneg	shCHD1	OK	6,75665	5,14031	-0,394452	-2,08171	0,0005	0,00321686	yes
KLHL24	KLHL24	KLHL24	chr3:1833534 shneg	shCHD1	OK	7,7263	5,87782	-0,394498	-2,07115	0,00035	0,00236888	yes
RNF149	RNF149	RNF149	chr2:1018920 shneg	shCHD1	OK	10,8863	8,2797	-0,394865	-1,95328	0,0009	0,00533451	yes
NME3	NME3	NME3	chr16:182032 shneg	shCHD1	OK	52,4812	39,9124	-0,394963	-2,03026	0,00035	0,00236888	yes
RUFY3	RUFY3	RUFY3	chr4:7157065 shneg	shCHD1	OK	6,6077	5,02499	-0,395027	-1,63852	0,0041	0,0189794	yes
RPL37	RPL37	RPL37	chr5:4083142 shneg	shCHD1	OK	715,248	543,88	-0,395155	-2,37552	5,00E-05	0,0004193	yes
ZNF771	ZNF771	ZNF771	chr16:304187 shneg	shCHD1	OK	9,40926	7,15486	-0,395158	-1,78163	0,0026	0,0129897	yes
ADD1	ADD1	ADD1	chr4:2845583 shneg	shCHD1	OK	37,8764	28,7855	-0,395956	-2,21176	0,0002	0,00144867	yes
BCL9L	BCL9L	BCL9L	chr11:118754 shneg	shCHD1	OK	22,0707	16,7729	-0,396	-2,22773	0,0001	0,00078473	yes
PHKA2	PHKA2	PHKA2	chrX:1890841 shneg	shCHD1	OK	7,05313	5,35978	-0,396089	-1,99818	0,00095	0,00559279	yes
TAAR6	TAAR6	TAAR6	chr6:1328914 shneg	shCHD1	OK	11,9084	9,04905	-0,396138	-1,69262	0,0034	0,0162243	yes
BPTF	BPTF	BPTF	chr17:658217 shneg	shCHD1	OK	12,8568	9,76727	-0,396508	-2,20728	5,00E-05	0,0004193	yes
TMC6	TMC6	TMC6	chr17:761089 shneg	shCHD1	OK	8,06359	6,12576	-0,396533	-1,82776	0,0014	0,00768951	yes
STXBP1	STXBP1	STXBP1	chr9:1303744 shneg	shCHD1	OK	20,8473	15,8338	-0,396848	-2,13126	0,00035	0,00236888	yes
TRAM2	TRAM2	TRAM2	chr6:5236219 shneg	shCHD1	OK	10,4847	7,96326	-0,396851	-2,12727	0,00015	0,00112331	yes
QPRT	QPRT	QPRT	chr16:296904 shneg	shCHD1	OK	96,6792	73,4089	-0,397251	-2,2144	5,00E-05	0,0004193	yes
HMGXB4	HMGXB4	HMGXB4	chr22:356534 shneg	shCHD1	OK	8,58816	6,51948	-0,397591	-1,9941	0,0005	0,00321686	yes
AHI1	AHI1	AHI1	chr6:1356051 shneg	shCHD1	OK	9,75271	7,40164	-0,397958	-1,89796	0,0014	0,00768951	yes
COL18A1	COL18A1	COL18A1	chr21:468250 shneg	shCHD1	OK	175,422	133,126	-0,398042	-2,38344	5,00E-05	0,0004193	yes
FAM127A	FAM127A	FAM127A	chrX:1341663 shneg	shCHD1	OK	69,5811	52,8042	-0,398043	-2,13335	0,0004	0,00266202	yes
CCDC74B	CCDC74B	CCDC74B	chr2:1308871 shneg	shCHD1	OK	9,55809	7,25295	-0,398154	-1,74974	0,0026	0,0129897	yes
UPRT	UPRT	UPRT	chrX:7449389 shneg	shCHD1	OK	5,27899	4,00559	-0,398247	-1,81651	0,0019	0,00996052	yes
LETM1	LETM1	LETM1	chr4:1813205 shneg	shCHD1	OK	7,76025	5,88609	-0,398793	-2,04268	0,00035	0,00236888	yes
LMO7	LMO7	LMO7	chr13:761945 shneg	shCHD1	OK	9,0804	6,88701	-0,398879	-2,11349	0,0003	0,0020738	yes
ACP6	ACP6	ACP6	chr1:1471191 shneg	shCHD1	OK	9,07087	6,87868	-0,399109	-1,8751	0,00115	0,00656221	yes
PSMB10	PSMB10	PSMB10	chr16:679684 shneg	shCHD1	OK	36,9704	28,0344	-0,399168	-1,96382	0,0006	0,00377139	yes

C10orf32	C10orf32	C10orf32	chr10:104613	shneg	shCHD1	OK	7,91044	5,99721	-0,399466	-1,67105	0,0044	0,0201471	yes
STK38	STK38	STK38	chr6:3646166	shneg	shCHD1	OK	32,086	24,323	-0,399621	-2,2044	0,0001	0,00078473	yes
TTC14	TTC14	TTC14	chr3:1803199	shneg	shCHD1	OK	12,1253	9,18502	-0,400666	-1,95343	0,00045	0,00293585	yes
PQLC3	PQLC3	PQLC3	chr2:1129553	shneg	shCHD1	OK	17,15	12,9891	-0,400905	-1,9567	0,0006	0,00377139	yes
CASP9	CASP9	CASP9	chr1:1581876	shneg	shCHD1	OK	6,76759	5,12179	-0,401993	-1,8422	0,0013	0,00726051	yes
ANAPC4	ANAPC4	ANAPC4	chr4:2537884	shneg	shCHD1	OK	20,2242	15,3032	-0,402245	-2,09384	0,0002	0,00144867	yes
NCOA3	NCOA3	NCOA3	chr20:461306	shneg	shCHD1	OK	40,0003	30,2671	-0,402261	-2,32102	5,00E-05	0,0004193	yes
IL17RC	IL17RC	IL17RC	chr3:9958757	shneg	shCHD1	OK	17,079	12,923	-0,40228	-2,02831	0,00065	0,0040329	yes
SCRN3	SCRN3	SCRN3	chr2:1752604	shneg	shCHD1	OK	6,59061	4,98492	-0,40284	-1,96025	0,0006	0,00377139	yes
MTM1	MTM1	MTM1	chrX:1497370	shneg	shCHD1	OK	4,39206	3,32139	-0,403113	-1,92341	0,00085	0,00508358	yes
CRIM1	CRIM1	CRIM1	chr2:3658336	shneg	shCHD1	OK	41,4773	31,365	-0,403168	-2,31127	0,0002	0,00144867	yes
WDSUB1	WDSUB1	WDSUB1	chr2:1600923	shneg	shCHD1	OK	9,1715	6,93449	-0,403369	-1,95367	0,001	0,0058183	yes
GIP	GIP	GIP	chr17:470359	shneg	shCHD1	OK	40,0066	30,2377	-0,403889	-1,9153	0,00085	0,00508358	yes
CYB561D1	CYB561D1	CYB561D1	chr1:1100366	shneg	shCHD1	OK	2,75521	2,08195	-0,404225	-1,85665	0,00135	0,00748275	yes
NCOR2	NCOR2	NCOR2	chr12:124808	shneg	shCHD1	OK	79,0558	59,7213	-0,404625	-2,38124	5,00E-05	0,0004193	yes
MMGT1	MMGT1	MMGT1	chrX:1350442	shneg	shCHD1	OK	14,5167	10,9573	-0,405815	-2,1142	0,00015	0,00112331	yes
NOA1	NOA1	NOA1	chr4:5782951	shneg	shCHD1	OK	24,2803	18,3147	-0,406784	-2,12501	0,0003	0,0020738	yes
CD55	CD55	CD55	chr1:2074948	shneg	shCHD1	OK	4,93069	3,71704	-0,407635	-1,88362	0,0013	0,00726051	yes
SPINT2	SPINT2	SPINT2	chr19:387550	shneg	shCHD1	OK	153,344	115,534	-0,408458	-2,34937	5,00E-05	0,0004193	yes
ARL4C	ARL4C	ARL4C	chr2:2354016	shneg	shCHD1	OK	140,182	105,607	-0,408603	-2,41948	0,00015	0,00112331	yes
NEK11	NEK11	NEK11	chr3:1307456	shneg	shCHD1	OK	5,4297	4,09016	-0,408716	-1,8889	0,00065	0,0040329	yes
ARMCX5	ARMCX5	ARMCX5	chrX:1018540	shneg	shCHD1	OK	7,09896	5,34739	-0,408771	-1,45957	0,01145	0,0444873	yes
TMCO4	TMCO4	TMCO4	chr1:2000870	shneg	shCHD1	OK	3,06392	2,3077	-0,408928	-1,64379	0,005	0,0224661	yes
GNS	GNS	GNS	chr12:651072	shneg	shCHD1	OK	41,6698	31,3829	-0,409023	-2,33601	5,00E-05	0,0004193	yes
HOOK1	HOOK1	HOOK1	chr1:6028053	shneg	shCHD1	OK	5,15157	3,87895	-0,409345	-1,9977	0,00025	0,00176854	yes
LTBP4	LTBP4	LTBP4	chr19:410990	shneg	shCHD1	OK	44,6305	33,597	-0,409695	-2,32586	0,00015	0,00112331	yes
BCAS3	BCAS3	BCAS3	chr17:587551	shneg	shCHD1	OK	12,5913	9,47826	-0,409731	-2,11064	0,00015	0,00112331	yes
AMZ2P1	AMZ2P1	AMZ2P1	chr17:629626	shneg	shCHD1	OK	3,13958	2,3633	-0,409769	-1,75807	0,0024	0,0121606	yes
HSBP1L1	HSBP1L1	HSBP1L1	chr18:777245	shneg	shCHD1	OK	20,0222	15,0663	-0,410273	-1,8047	0,002	0,0104181	yes
TTLL1	TTLL1	TTLL1	chr22:434355	shneg	shCHD1	OK	4,04063	3,04018	-0,410426	-1,47808	0,00985	0,0393915	yes
OCRL	OCRL	OCRL	chrX:1286742	shneg	shCHD1	OK	17,4747	13,1459	-0,410657	-2,22651	0,0002	0,00144867	yes
TBC1D22B	TBC1D22B	TBC1D22B	chr6:3717995	shneg	shCHD1	OK	7,95304	5,98192	-0,410898	-1,94335	0,00075	0,00455593	yes
FBXL4	FBXL4	FBXL4	chr6:9932160	shneg	shCHD1	OK	8,51984	6,40767	-0,411026	-1,96755	0,001	0,0058183	yes
FIG4	FIG4	FIG4	chr6:1100124	shneg	shCHD1	OK	6,01371	4,5226	-0,411104	-1,9554	0,00065	0,0040329	yes
ITPKA	ITPKA	ITPKA	chr15:417860	shneg	shCHD1	OK	6,34843	4,77377	-0,41127	-1,77918	0,00235	0,0119595	yes
PLEKHA4	PLEKHA4	PLEKHA4	chr19:493403	shneg	shCHD1	OK	6,90676	5,19347	-0,41131	-1,9432	0,00075	0,00455593	yes
SYTL1	SYTL1	SYTL1	chr1:2766848	shneg	shCHD1	OK	28,6456	21,5395	-0,41133	-2,14403	0,0001	0,00078473	yes
SKIL	SKIL	SKIL	chr3:1700754	shneg	shCHD1	OK	10,3193	7,75866	-0,411467	-2,15667	0,00015	0,00112331	yes
EPB41L4B	EPB41L4B	EPB41L4B	chr9:1119342	shneg	shCHD1	OK	6,48353	4,87443	-0,411546	-1,98702	0,0005	0,00321686	yes
PRRG4	PRRG4	PRRG4	chr11:328514	shneg	shCHD1	OK	2,64248	1,98591	-0,412093	-1,95875	0,0008	0,0048114	yes
SCAMP3	SCAMP3	SCAMP3	chr1:1552257	shneg	shCHD1	OK	43,2573	32,5022	-0,412406	-2,17385	0,00015	0,00112331	yes
CNNM2	CNNM2	CNNM2	chr10:104678	shneg	shCHD1	OK	8,29916	6,23498	-0,412582	-1,76816	0,00315	0,015272	yes
NOTCH2NL	NOTCH2NL	NOTCH2NL	chr1:1452091	shneg	shCHD1	OK	3,44413	2,58738	-0,412648	-1,96074	0,0004	0,00266202	yes
ST3GAL1	ST3GAL1	ST3GAL1	chr8:1344670	shneg	shCHD1	OK	2,69453	2,02417	-0,412701	-1,98009	0,00065	0,0040329	yes
ABHD14B	ABHD14B	ABHD14B	chr3:5200252	shneg	shCHD1	OK	43,9485	33,0134	-0,41276	-2,22985	0,00015	0,00112331	yes
PDGFC	PDGFC	PDGFC	chr4:1576827	shneg	shCHD1	OK	31,5307	23,685	-0,41278	-2,23934	0,0001	0,00078473	yes
MRPL41	MRPL41	MRPL41	chr9:1404463	shneg	shCHD1	OK	154,986	116,33	-0,413915	-2,1939	0,00015	0,00112331	yes
FAM84B	FAM84B	FAM84B	chr8:1275646	shneg	shCHD1	OK	1,88893	1,41776	-0,413957	-1,76316	0,0022	0,011316	yes
POLL	POLL	POLL	chr10:103338	shneg	shCHD1	OK	13,324	9,99843	-0,414257	-1,97451	0,0004	0,00266202	yes
TUBGCP6	TUBGCP6	TUBGCP6	chr22:506561	shneg	shCHD1	OK	9,85056	7,38955	-0,414719	-2,17796	5,00E-05	0,0004193	yes
ZBTB38	ZBTB38	ZBTB38	chr3:1410430	shneg	shCHD1	OK	26,4252	19,8185	-0,415065	-2,3748	5,00E-05	0,0004193	yes
CLIP3	CLIP3	CLIP3	chr19:365055	shneg	shCHD1	OK	3,15089	2,36291	-0,415198	-1,77263	0,00195	0,0102111	yes
CES3	CES3	CES3	chr16:669951	shneg	shCHD1	OK	3,38518	2,53855	-0,415228	-1,80462	0,00165	0,00886	yes
MIR31HG	MIR31HG	MIR31HG	chr9:2145426	shneg	shCHD1	OK	7,742	5,80416	-0,415621	-1,60808	0,0059	0,0257621	yes
RPL34	RPL34	RPL34	chr4:1095417	shneg	shCHD1	OK	483,561	362,46	-0,415877	-2,43362	5,00E-05	0,0004193	yes
CUL9	CUL9	CUL9	chr6:4314992	shneg	shCHD1	OK	3,48284	2,61029	-0,41605	-2,0205	0,00045	0,00293585	yes
DENND3	DENND3	DENND3	chr8:1421387	shneg	shCHD1	OK	51,4278	38,536	-0,416341	-2,40456	5,00E-05	0,0004193	yes
BMPR2	BMPR2	BMPR2	chr2:2032410	shneg	shCHD1	OK	9,52753	7,13803	-0,416575	-2,28471	0,00015	0,00112331	yes
ANKRD29	ANKRD29	ANKRD29	chr18:211799	shneg	shCHD1	OK	14,9014	11,1638	-0,416618	-2,05701	0,0005	0,00321686	yes
DLK1	DLK1	DLK1	chr14:101193	shneg	shCHD1	OK	30,279	22,6764	-0,417126	-2,13649	0,00035	0,00236888	yes
RPL38	RPL38	RPL38	chr17:721997	shneg	shCHD1	OK	1537,14	1151,11	-0,417227	-2,39061	5,00E-05	0,0004193	yes
ZDHHC21	ZDHHC21	ZDHHC21	chr9:1461106	shneg	shCHD1	OK	2,22213	1,66392	-0,41736	-1,92861	0,00115	0,00656221	yes
CCDC85B	CCDC85B	CCDC85B	chr11:656578	shneg	shCHD1	OK	136,045	101,86	-0,417502	-2,32777	0,0001	0,00078473	yes
ZNF225	ZNF225	ZNF225	chr19:446175	shneg	shCHD1	OK	2,21128	1,65545	-0,417659	-1,44176	0,011	0,043039	yes
KIAA1161	KIAA1161	KIAA1161	chr9:3436890	shneg	shCHD1	OK	2,90459	2,17442	-0,417701	-1,87077	0,00145	0,00793286	yes
FAM199X	FAM199X	FAM199X	chrX:1034111	shneg	shCHD1	OK	21,3988	16,0165	-0,41797	-2,34276	5,00E-05	0,0004193	yes
PPP1R9A	PPP1R9A	PPP1R9A	chr7:9453694	shneg	shCHD1	OK	6,20935	4,64737	-0,418028	-2,18952	0,0002	0,00144867	yes
HSDL2	HSDL2	HSDL2	chr9:1151421	shneg	shCHD1	OK	17,3432	12,9803	-0,418052	-2,20778	0,0002	0,00144867	yes
NKD1	NKD1	NKD1	chr16:505822	shneg	shCHD1	OK	1,59408	1,19294	-0,4182	-1,96496	0,001	0,0058183	yes
STX18	STX18	STX18	chr4:4387982	shneg	shCHD1	OK	8,44212	6,31404	-0,419043	-1,99141	0,00045	0,00293585	yes
CD99L2	CD99L2	CD99L2	chrX:1499348	shneg	shCHD1	OK	37,2025	27,8234	-0,419101	-2,34293	0,00015	0,00112331	yes
SMARCA1	SMARCA1	SMARCA1	chrX:1285804	shneg	shCHD1	OK	78,5444	58,7383	-0,419206	-2,41438	5,00E-05	0,0004193	yes
KLF16	KLF16	KLF16	chr19:185239	shneg	shCHD1	OK	21,2567	15,8958	-0,419269	-2,20574	0,00015	0,00112331	yes
MUC1	MUC1	MUC1	chr1:1551582	shneg	shCHD1	OK	6,90871	5,16595	-0,419381	-1,56087	0,00175	0,00931439	yes
ALDH3A2	ALDH3A2	ALDH3A2	chr17:195520	shneg	shCHD1	OK	78,2056	58,4742	-0,419472	-2,41548	5,00E-05	0,0004193	yes
RPL36A	RPL36A	RPL36A	chrX:1006458	shneg	shCHD1	OK	1106,48	827,219	-0,419632	-2,14235	0,0003	0,0020738	yes
ADCK4	ADCK4	ADCK4	chr19:411974	shneg	shCHD1	OK	16,4353	12,2869	-0,419677	-2,08899	0,00045	0,00293585	yes
MFSD6	MFSD6	MFSD6	chr2:1912730	shneg	shCHD1	OK	11,2778	8,43117	-0,419685	-2,19684	0,0001	0,00078473	yes
JOSD2	JOSD2	JOSD2	chr19:510092	shneg	shCHD1	OK	34,6805	25,9221	-0,419942	-2,0504	0,00045	0,00293585	yes
TRAPPC11	TRAPPC11	TRAPPC11	chr4:1845804	shneg	shCHD1	OK	8,6384	6,45639	-0,420037	-2,11399	0,0002	0,00144867	yes
CLIP4	CLIP4	CLIP4	chr2:2933830	shneg	shCHD1	OK	3,08279	2,30406	-0,420059	-1,92092	0,00065	0,0040329	yes
SMARCD3	SMARCD3	SMARCD3	chr7:1509360	shneg	shCHD1	OK	52,9537	39,575	-0,420144	-2,26572	5,00E-05	0,0004193	yes
KIAA2018	KIAA2018	KIAA2018	chr3:1133672	shneg	shCHD1	OK	1,77815	1,32857	-0,420508	-2,01302	0,00055	0,00350455	yes
ADAM17	ADAM17	ADAM17	chr2:9629410	shneg	shCHD1	OK	20,3045	15,17	-0,420584	-2,24472	0,00015	0,00112331	yes
MEF2D	MEF2D	MEF2D	chr1:1564335	shneg	shCHD1	OK	10,5515	7,88272	-0,420684	-2,22628	0,0002	0,00144867	yes
PCSK4	PCSK4	PCSK4	chr19:148142	shneg	shCHD1	OK	3,97534	2,96983	-0,420697	-1,77357	0,00245	0,0123735	yes
TMEM128	TMEM128	TMEM128	chr4:4237268	shneg	shCHD1	OK	5,98474	4,4707	-0,420788	-1,73143	0,00275	0,0136364	yes
ZMAT3	ZMAT3	ZMAT3	chr3:1787350	shneg	shCHD1	OK	1,77384	1,32491	-0,42098	-2,01319	0,00045	0,00293585	yes
SH3BGRL	SH3BGRL	SH3BGRL	chrX:8036919	shneg	shCHD1	OK	65,5648	48,9649	-0,421173	-2,28757	5,00E-05	0,0004193	yes
UTY	UTY	UTY	chrY:1536026	shneg	shCHD1	OK	6,80073	5,07841	-0,421312	-2,03764	0,0005	0,00321686	yes
MSMO1	MSMO1	MSMO1	chr4:1662488	shneg	shCHD1	OK	14,1792	10,588	-0,421346	-2,06019	0,0006	0,00377139	yes
TXNRD3	TXNRD3	TXNRD3	chr3:1262906	shneg	shCHD1	OK	4,50034	3,35966	-0,42172	-1,88867	0,0013	0,00726051	yes
FAM149B1	FAM149B1	FAM149B1	chr10:749278	shneg	shCHD1	OK	11,4542	8,54648	-0,422473	-2,11566	0,0003	0,0020738	yes

MFSD8	MFSD8	MFSD8	chr4:1288389	shneg	shCHD1	OK	6,06615	4,52589	-0,42258	-1,86241	0,00135	0,00748275	yes
ADAMTS9	ADAMTS9	ADAMTS9	chr3:6450133	shneg	shCHD1	OK	12,5251	9,34364	-0,422764	-2,26881	5,00E-05	0,0004193	yes
SOX12	SOX12	SOX12	chr20:306238	shneg	shCHD1	OK	9,10408	6,79123	-0,42284	-2,15717	0,0002	0,00144867	yes
RPS6KA6	RPS6KA6	RPS6KA6	chrX:8331335	shneg	shCHD1	OK	3,95314	2,9476	-0,42346	-2,08576	0,00045	0,00293585	yes
LOC10050633	LOC10050633	LOC10050633	chr21:353035	shneg	shCHD1	OK	7,01774	5,23183	-0,42369	-1,99486	0,0004	0,00266202	yes
PPME1	PPME1	PPME1	chr11:738823	shneg	shCHD1	OK	130,023	96,9012	-0,424185	-2,45666	5,00E-05	0,0004193	yes
C7orf60	C7orf60	C7orf60	chr7:1124592	shneg	shCHD1	OK	3,59396	2,67795	-0,424445	-1,98553	0,00075	0,00455593	yes
LRP8	LRP8	LRP8	chr1:5369256	shneg	shCHD1	OK	62,1952	46,3335	-0,424746	-2,3987	0,0001	0,00078473	yes
ABCC3	ABCC3	ABCC3	chr17:487122	shneg	shCHD1	OK	73,3368	54,6291	-0,424867	-2,43259	5,00E-05	0,0004193	yes
SYNE1	SYNE1	SYNE1	chr6:1524428	shneg	shCHD1	OK	32,3525	24,0984	-0,424939	-2,5411	5,00E-05	0,0004193	yes
MUC6	MUC6	MUC6	chr11:101282	shneg	shCHD1	OK	1,59051	1,18445	-0,425279	-1,81058	0,0025	0,012594	yes
COL6A3	COL6A3	COL6A3	chr2:2382326	shneg	shCHD1	OK	344,677	256,656	-0,425408	-2,54516	5,00E-05	0,0004193	yes
ATP8A1	ATP8A1	ATP8A1	chr4:4241039	shneg	shCHD1	OK	32,4565	24,1642	-0,425633	-2,46883	5,00E-05	0,0004193	yes
TRIM37	TRIM37	TRIM37	chr17:568332	shneg	shCHD1	OK	12,2752	9,13847	-0,425721	-2,18998	0,0002	0,00144867	yes
GLB1L	GLB1L	GLB1L	chr2:2201015	shneg	shCHD1	OK	5,28501	3,93346	-0,426109	-1,92971	0,00125	0,00702633	yes
GLB1	GLB1	GLB1	chr3:3303809	shneg	shCHD1	OK	65,0914	48,4407	-0,426246	-2,34209	5,00E-05	0,0004193	yes
ZNF211	ZNF211	ZNF211	chr19:581445	shneg	shCHD1	OK	6,53587	4,86144	-0,426996	-2,0212	0,00045	0,00293585	yes
ZNF490	ZNF490	ZNF490	chr19:126869	shneg	shCHD1	OK	1,98094	1,47289	-0,427538	-1,91297	0,001	0,0058183	yes
FKTN	FKTN	FKTN	chr9:1083204	shneg	shCHD1	OK	6,56279	4,87863	-0,427833	-2,1193	0,0003	0,0020738	yes
ICAM5	ICAM5	ICAM5	chr19:104006	shneg	shCHD1	OK	16,0144	11,9036	-0,427969	-2,19553	0,0003	0,0020738	yes
PGM5P2	PGM5P2	PGM5P2	chr9:6908024	shneg	shCHD1	OK	25,6614	19,0741	-0,427986	-2,19653	0,0002	0,00144867	yes
PTMS	PTMS	PTMS	chr12:687554	shneg	shCHD1	OK	607,719	451,694	-0,42806	-2,52992	5,00E-05	0,0004193	yes
MAPK14	MAPK14	MAPK14	chr6:3599545	shneg	shCHD1	OK	20,2222	15,0275	-0,428339	-2,11446	0,00015	0,00112331	yes
TMEM106B	TMEM106B	TMEM106B	chr7:1225084	shneg	shCHD1	OK	7,44138	5,52982	-0,428339	-2,18514	0,00015	0,00112331	yes
DTX3	DTX3	DTX3	chr12:579986	shneg	shCHD1	OK	24,652	18,3164	-0,428564	-2,20893	0,00025	0,00176854	yes
SLC41A2	SLC41A2	SLC41A2	chr12:105197	shneg	shCHD1	OK	2,89354	2,14972	-0,42869	-1,80051	0,00155	0,00838119	yes
MARC1	MARC1	MARC1	chr1:2209600	shneg	shCHD1	OK	4,27259	3,17244	-0,429519	-1,74471	0,0029	0,0142283	yes
SNHG7	SNHG7	SNHG7	chr9:1396070	shneg	shCHD1	OK	74,7594	55,5033	-0,42968	-1,89781	0,0011	0,0063157	yes
TMEM30A	TMEM30A	TMEM30A	chr6:7596263	shneg	shCHD1	OK	43,8002	32,5153	-0,429819	-2,42932	5,00E-05	0,0004193	yes
ARSE	ARSE	ARSE	chrX:2852672	shneg	shCHD1	OK	44,3948	32,9547	-0,429905	-2,3403	5,00E-05	0,0004193	yes
SLC35D2	SLC35D2	SLC35D2	chr9:9908298	shneg	shCHD1	OK	33,1862	24,6281	-0,430278	-2,23188	5,00E-05	0,0004193	yes
RABGAP1L	RABGAP1L	RABGAP1L	chr1:1741285	shneg	shCHD1	OK	7,79962	5,78778	-0,430395	-1,8509	0,0014	0,00768951	yes
CNIH3	CNIH3	CNIH3	chr1:2248041	shneg	shCHD1	OK	5,87545	4,35831	-0,430932	-1,98337	0,0008	0,0048114	yes
MICAL2	MICAL2	MICAL2	chr11:121321	shneg	shCHD1	OK	55,3366	41,0393	-0,43123	-2,46198	5,00E-05	0,0004193	yes
NPC2	NPC2	NPC2	chr14:749466	shneg	shCHD1	OK	142,363	105,576	-0,431286	-2,36428	5,00E-05	0,0004193	yes
CXorf40A	CXorf40A	CXorf40A	chrX:1486225	shneg	shCHD1	OK	5,75939	4,27078	-0,431416	-1,82679	0,0018	0,00951868	yes
C11orf63	C11orf63	C11orf63	chr11:122753	shneg	shCHD1	OK	5,69929	4,22592	-0,431518	-1,71884	0,0036	0,0170733	yes
RAB33B	RAB33B	RAB33B	chr4:1403749	shneg	shCHD1	OK	3,41146	2,52952	-0,431525	-1,96945	0,0006	0,00377139	yes
GAK	GAK	GAK	chr4:843064-[illegible]	shneg	shCHD1	OK	11,2676	8,35447	-0,431557	-2,2444	0,00015	0,00112331	yes
DYRK4	DYRK4	DYRK4	chr12:469924	shneg	shCHD1	OK	6,02697	4,46759	-0,431938	-1,83014	0,00135	0,00748275	yes
SLCO4A1	SLCO4A1	SLCO4A1	chr20:612737	shneg	shCHD1	OK	103,199	76,4879	-0,432119	-2,3356	5,00E-05	0,0004193	yes
LINC00493	LINC00493	LINC00493	chr20:185480	shneg	shCHD1	OK	28,3618	21,0206	-0,432147	-1,71701	0,0035	0,0166331	yes
MCOLN2	MCOLN2	MCOLN2	chr1:8539126	shneg	shCHD1	OK	5,56261	4,12108	-0,43274	-2,03302	0,0004	0,00266202	yes
CEP70	CEP70	CEP70	chr3:1382131	shneg	shCHD1	OK	11,1411	8,25199	-0,433083	-2,11034	0,0003	0,0020738	yes
DYNLT3	DYNLT3	DYNLT3	chrX:3769808	shneg	shCHD1	OK	11,3742	8,42449	-0,433109	-2,05486	0,00025	0,00176854	yes
FTSJD1	FTSJD1	FTSJD1	chr16:713162	shneg	shCHD1	OK	9,32417	6,90498	-0,433339	-2,17223	0,0002	0,00144867	yes
TM7SF2	TM7SF2	TM7SF2	chr11:648793	shneg	shCHD1	OK	15,9449	11,7993	-0,43439	-2,05148	0,00025	0,00176854	yes
WDR47	WDR47	WDR47	chr1:1095128	shneg	shCHD1	OK	21,9706	16,2558	-0,434622	-2,36978	5,00E-05	0,0004193	yes
FAM3A	FAM3A	FAM3A	chrX:1537333	shneg	shCHD1	OK	17,985	13,2977	-0,435616	-2,25474	0,0001	0,00078473	yes
APOE	APOE	APOE	chr19:454090	shneg	shCHD1	OK	327,916	242,368	-0,436127	-2,52341	5,00E-05	0,0004193	yes
TRAF4	TRAF4	TRAF4	chr17:270710	shneg	shCHD1	OK	22,9881	16,988	-0,436367	-2,31205	5,00E-05	0,0004193	yes
KIAA1377	KIAA1377	KIAA1377	chr11:101761	shneg	shCHD1	OK	4,21861	3,11747	-0,436392	-2,14061	5,00E-05	0,0004193	yes
CHST7	CHST7	CHST7	chrX:4643319	shneg	shCHD1	OK	21,6938	16,0311	-0,436416	-2,24948	5,00E-05	0,0004193	yes
MPPE1	MPPE1	MPPE1	chr18:118834	shneg	shCHD1	OK	5,41435	4,0001	-0,436752	-2,01699	0,00045	0,00293585	yes
ANKDD1A	ANKDD1A	ANKDD1A	chr15:652041	shneg	shCHD1	OK	3,7353	2,7596	-0,436767	-1,91082	0,0015	0,00814879	yes
TAF1D	TAF1D	TAF1D	chr11:934690	shneg	shCHD1	OK	34,8747	25,7515	-0,437521	-2,21714	0,00015	0,00112331	yes
TCTN1	TCTN1	TCTN1	chr12:111051	shneg	shCHD1	OK	21,673	15,996	-0,43819	-1,95733	0,001	0,0058183	yes
PMP22	PMP22	PMP22	chr17:151330	shneg	shCHD1	OK	10,3276	7,62113	-0,438427	-2,03263	0,0006	0,00377139	yes
CCNL2	CCNL2	CCNL2	chr1:1321090	shneg	shCHD1	OK	104,116	76,8309	-0,438431	-2,16943	0,00015	0,00112331	yes
LRFN3	LRFN3	LRFN3	chr19:364280	shneg	shCHD1	OK	8,31865	6,1379	-0,438603	-2,12667	0,0001	0,00078473	yes
CBFA2T3	CBFA2T3	CBFA2T3	chr16:889412	shneg	shCHD1	OK	7,28697	5,37582	-0,438835	-2,14503	0,0002	0,00144867	yes
ADAP2	ADAP2	ADAP2	chr17:292487	shneg	shCHD1	OK	7,2019	5,31234	-0,43903	-2,10667	0,00045	0,00293585	yes
TBXA2R	TBXA2R	TBXA2R	chr19:359450	shneg	shCHD1	OK	2,84054	2,09442	-0,439618	-1,61101	0,0041	0,0189794	yes
GSN	GSN	GSN	chr9:1240303	shneg	shCHD1	OK	21,5803	15,9097	-0,439813	-2,29236	5,00E-05	0,0004193	yes
COCH	COCH	COCH	chr14:313437	shneg	shCHD1	OK	7,97384	5,87741	-0,440095	-1,49088	0,0106	0,0418028	yes
CROT	CROT	CROT	chr7:8697495	shneg	shCHD1	OK	5,37231	3,95911	-0,440366	-1,69234	0,00415	0,0191853	yes
ZSCAN30	ZSCAN30	ZSCAN30	chr18:328209	shneg	shCHD1	OK	4,05027	2,98293	-0,441289	-1,46949	0,01215	0,0467765	yes
C6orf72	C6orf72	C6orf72	chr6:1498875	shneg	shCHD1	OK	36,0019	26,5033	-0,4419	-2,20166	0,0001	0,00078473	yes
APPBP2	APPBP2	APPBP2	chr17:585205	shneg	shCHD1	OK	15,58	11,4687	-0,441992	-2,41387	5,00E-05	0,0004193	yes
FBXO3	FBXO3	FBXO3	chr11:337624	shneg	shCHD1	OK	21,864	16,0935	-0,442078	-2,17537	5,00E-05	0,0004193	yes
RBM43	RBM43	RBM43	chr2:1521047	shneg	shCHD1	OK	5,97612	4,39826	-0,442275	-2,11546	0,0001	0,00078473	yes
FAM171A1	FAM171A1	FAM171A1	chr10:152536	shneg	shCHD1	OK	57,8598	42,5614	-0,443016	-2,53308	5,00E-05	0,0004193	yes
DCUN1D4	DCUN1D4	DCUN1D4	chr4:5270927	shneg	shCHD1	OK	13,2318	9,73222	-0,443174	-2,32648	0,00015	0,00112331	yes
KIF21A	KIF21A	KIF21A	chr12:396870	shneg	shCHD1	OK	8,59351	6,32032	-0,443248	-2,32197	5,00E-05	0,0004193	yes
RETSAT	RETSAT	RETSAT	chr2:8556907	shneg	shCHD1	OK	19,6284	14,4354	-0,443338	-2,34618	0,0001	0,00078473	yes
SLC25A20	SLC25A20	SLC25A20	chr3:4889435	shneg	shCHD1	OK	9,06633	6,66584	-0,443732	-2,10411	0,0003	0,0020738	yes
C17orf108	C17orf108	C17orf108	chr17:262053	shneg	shCHD1	OK	3,87611	2,84981	-0,443745	-1,45615	0,0113	0,044015	yes
TTC21A	TTC21A	TTC21A	chr3:3914915	shneg	shCHD1	OK	2,63309	1,93584	-0,443795	-1,91362	0,00165	0,00886	yes
DHX40	DHX40	DHX40	chr17:576428	shneg	shCHD1	OK	35,5306	26,1098	-0,444471	-2,47063	5,00E-05	0,0004193	yes
DMXL2	DMXL2	DMXL2	chr15:517399	shneg	shCHD1	OK	7,40869	5,44342	-0,444706	-2,17346	0,0002	0,00144867	yes
TSNAXIP1	TSNAXIP1	TSNAXIP1	chr16:678410	shneg	shCHD1	OK	2,70785	1,98885	-0,445214	-1,58371	0,00525	0,0232593	yes
ZNF417	ZNF417	ZNF417	chr19:584171	shneg	shCHD1	OK	2,68967	1,97532	-0,445345	-2,01414	0,0003	0,0020738	yes
F11R	F11R	F11R	chr1:1609650	shneg	shCHD1	OK	5,41253	3,97462	-0,445486	-2,14385	0,0002	0,00144867	yes
SMPD1	SMPD1	SMPD1	chr11:641164	shneg	shCHD1	OK	9,09273	6,67389	-0,446186	-2,14688	0,0001	0,00078473	yes
SEMA6B	SEMA6B	SEMA6B	chr19:454259	shneg	shCHD1	OK	24,2963	17,8277	-0,446615	-2,42137	5,00E-05	0,0004193	yes
DCAKD	DCAKD	DCAKD	chr17:431007	shneg	shCHD1	OK	14,9045	10,9363	-0,446631	-2,16162	0,0004	0,00266202	yes
RAB11FIP3	RAB11FIP3	RAB11FIP3	chr16:475667	shneg	shCHD1	OK	11,951	8,76698	-0,446984	-2,22071	0,00015	0,00112331	yes
BTN3A3	BTN3A3	BTN3A3	chr6:2644069	shneg	shCHD1	OK	3,6524	2,67922	-0,447031	-1,86095	0,0015	0,00814879	yes
HTR2B	HTR2B	HTR2B	chr2:2319215	shneg	shCHD1	OK	51,3695	37,6821	-0,447033	-1,56973	0,0084	0,0345449	yes
ITPR1	ITPR1	ITPR1	chr3:4535031	shneg	shCHD1	OK	1,43282	1,05019	-0,448208	-1,98576	0,001	0,0058183	yes
OXNAD1	OXNAD1	OXNAD1	chr3:1630666	shneg	shCHD1	OK	2,51258	1,84107	-0,448624	-1,87098	0,00145	0,00793286	yes
VEGFB	VEGFB	VEGFB	chr11:640020	shneg	shCHD1	OK	36,5906	26,8112	-0,448637	-2,36393	5,00E-05	0,0004193	yes
MPP5	MPP5	MPP5	chr14:677080	shneg	shCHD1	OK	10,282	7,53242	-0,448941	-2,3448	0,0001	0,00078473	yes

OSTM1	OSTM1	OSTM1	chr6:1083626 shneg	shCHD1	OK	16,6763	12,2129	-0,449391	-2,40503	5,00E-05	0,0004193	yes
KLHL8	KLHL8	KLHL8	chr4:8808221 shneg	shCHD1	OK	3,725	2,72765	-0,449582	-2,12619	0,00045	0,00293585	yes
GLTPD1	GLTPD1	GLTPD1	chr1:1260142 shneg	shCHD1	OK	11,8451	8,6722	-0,449821	-2,14054	0,0004	0,00266202	yes
C9orf7	C9orf7	C9orf7	chr9:1363250 shneg	shCHD1	OK	5,61214	4,1081	-0,450078	-2,06881	0,0003	0,0020738	yes
RFNG	RFNG	RFNG	chr17:800057 shneg	shCHD1	OK	13,6433	9,98317	-0,450617	-2,15838	0,0002	0,00144867	yes
LOC648987	LOC648987	LOC648987	chr5:4301483 shneg	shCHD1	OK	6,10228	4,46453	-0,450838	-2,03243	0,0002	0,00144867	yes
EXOC7	EXOC7	EXOC7	chr17:740752 shneg	shCHD1	OK	36,5764	26,7571	-0,45099	-2,54075	5,00E-05	0,0004193	yes
RAP2C	RAP2C	RAP2C	chrX:1313370 shneg	shCHD1	OK	19,8401	14,512	-0,451174	-2,41857	5,00E-05	0,0004193	yes
FAM63B	FAM63B	FAM63B	chr15:590633 shneg	shCHD1	OK	8,22544	6,01645	-0,45118	-2,22086	0,0002	0,00144867	yes
MLXIPL	MLXIPL	MLXIPL	chr7:7300752 shneg	shCHD1	OK	6,74732	4,93486	-0,451307	-2,17184	0,0002	0,00144867	yes
STAG2	STAG2	STAG2	chrX:1230944 shneg	shCHD1	OK	68,6927	50,2339	-0,451497	-2,61271	5,00E-05	0,0004193	yes
KIF17	KIF17	KIF17	chr1:2099050 shneg	shCHD1	OK	1,90699	1,39451	-0,451535	-1,65958	0,0041	0,0189794	yes
ASB4	ASB4	ASB4	chr7:9511521 shneg	shCHD1	OK	80,4981	58,8623	-0,451611	-2,31777	5,00E-05	0,0004193	yes
TRERF1	TRERF1	TRERF1	chr6:4219266 shneg	shCHD1	OK	13,2811	9,7109	-0,451702	-2,47318	5,00E-05	0,0004193	yes
PIGK	PIGK	PIGK	chr1:7755466 shneg	shCHD1	OK	18,5779	13,5797	-0,452142	-2,44219	5,00E-05	0,0004193	yes
DUSP7	DUSP7	DUSP7	chr3:5208293 shneg	shCHD1	OK	32,2117	23,5415	-0,452381	-2,47242	0,0001	0,00078473	yes
ARMCX2	ARMCX2	ARMCX2	chrX:1009102 shneg	shCHD1	OK	30,7436	22,4561	-0,45318	-2,44843	5,00E-05	0,0004193	yes
PTAR1	PTAR1	PTAR1	chr9:7232443 shneg	shCHD1	OK	9,13252	6,66981	-0,453366	-2,42816	5,00E-05	0,0004193	yes
SLC17A5	SLC17A5	SLC17A5	chr6:7430310 shneg	shCHD1	OK	8,01813	5,85579	-0,453403	-2,20207	0,0001	0,00078473	yes
CACNB4	CACNB4	CACNB4	chr2:1526892 shneg	shCHD1	OK	1,6697	1,21938	-0,453441	-2,0666	0,00065	0,0040329	yes
GALNT12	GALNT12	GALNT12	chr9:1015699 shneg	shCHD1	OK	3,60751	2,63393	-0,453788	-1,86679	0,00135	0,00748275	yes
ZHX1	ZHX1	ZHX1	chr8:1242321 shneg	shCHD1	OK	4,29821	3,13746	-0,45414	-1,48719	0,01055	0,0416174	yes
KHDRBS3	KHDRBS3	KHDRBS3	chr8:1364697 shneg	shCHD1	OK	3,21046	2,34341	-0,454169	-1,56205	0,00735	0,0308368	yes
CCDC132	CCDC132	CCDC132	chr7:9286165 shneg	shCHD1	OK	7,00453	5,11266	-0,454215	-2,15287	5,00E-05	0,0004193	yes
ACOX1	ACOX1	ACOX1	chr17:739375 shneg	shCHD1	OK	4,8638	3,54823	-0,454984	-1,76845	0,002	0,0104181	yes
IL15	IL15	IL15	chr4:1425577 shneg	shCHD1	OK	2,29259	1,67191	-0,455479	-1,46069	0,0093	0,0375913	yes
ZNF28	ZNF28	ZNF28	chr19:533006 shneg	shCHD1	OK	3,57026	2,60354	-0,455557	-2,16403	0,00025	0,00176854	yes
GUF1	GUF1	GUF1	chr4:4468043 shneg	shCHD1	OK	6,69725	4,88259	-0,455924	-2,20883	0,0001	0,00078473	yes
MKNK2	MKNK2	MKNK2	chr19:203746 shneg	shCHD1	OK	78,3569	57,0918	-0,456776	-2,33972	5,00E-05	0,0004193	yes
ZNF441	ZNF441	ZNF441	chr19:118778 shneg	shCHD1	OK	4,87063	3,54741	-0,457344	-2,14701	0,00035	0,00236888	yes
TTC8	TTC8	TTC8	chr14:892909 shneg	shCHD1	OK	12,8885	9,38074	-0,458317	-2,18126	0,0002	0,00144867	yes
B3GNT1	B3GNT1	B3GNT1	chr11:661128 shneg	shCHD1	OK	39,2848	28,5926	-0,458326	-2,45828	0,0001	0,00078473	yes
SHISA4	SHISA4	SHISA4	chr1:2018577 shneg	shCHD1	OK	20,7283	15,086	-0,458387	-2,1332	0,0001	0,00078473	yes
PLA2G16	PLA2G16	PLA2G16	chr11:633419 shneg	shCHD1	OK	15,4769	11,2618	-0,458685	-2,12243	0,00045	0,00293585	yes
KLHL21	KLHL21	KLHL21	chr1:6650783 shneg	shCHD1	OK	16,0261	11,6594	-0,458921	-2,44337	5,00E-05	0,0004193	yes
ELMOD2	ELMOD2	ELMOD2	chr4:1414453 shneg	shCHD1	OK	6,22008	4,52466	-0,459122	-2,21382	0,00015	0,00112331	yes
OPHN1	OPHN1	OPHN1	chrX:6726218 shneg	shCHD1	OK	3,50274	2,54796	-0,459138	-2,21557	0,0001	0,00078473	yes
CACNA1G	CACNA1G	CACNA1G	chr17:486335 shneg	shCHD1	OK	1,7746	1,29076	-0,459273	-1,98817	0,0002	0,00144867	yes
CNPY3	CNPY3	CNPY3	chr6:4289685 shneg	shCHD1	OK	73,1214	53,1775	-0,459479	-2,53891	5,00E-05	0,0004193	yes
TINAG	TINAG	TINAG	chr6:5417320 shneg	shCHD1	OK	46,2504	33,6299	-0,459723	-2,46711	5,00E-05	0,0004193	yes
ING2	ING2	ING2	chr4:1844262 shneg	shCHD1	OK	5,86628	4,26475	-0,459983	-1,55727	0,00665	0,0283439	yes
LPIN3	LPIN3	LPIN3	chr20:399695 shneg	shCHD1	OK	3,87399	2,81614	-0,460099	-2,02315	0,0005	0,00321686	yes
ANKRD34A	ANKRD34A	ANKRD34A	chr1:1454705 shneg	shCHD1	OK	2,10087	1,52715	-0,460152	-1,73109	0,00345	0,0164347	yes
QSOX1	QSOX1	QSOX1	chr1:1801239 shneg	shCHD1	OK	41,2577	29,9893	-0,460214	-2,32082	0,0001	0,00078473	yes
NAT9	NAT9	NAT9	chr17:727666 shneg	shCHD1	OK	23,8916	17,3645	-0,460363	-2,34141	0,00015	0,00112331	yes
CUL7	CUL7	CUL7	chr6:4300535 shneg	shCHD1	OK	30,3595	22,0566	-0,460937	-2,59745	5,00E-05	0,0004193	yes
ARHGAP4	ARHGAP4	ARHGAP4	chrX:1531728 shneg	shCHD1	OK	105,635	76,7147	-0,461513	-2,70653	5,00E-05	0,0004193	yes
FAM135A	FAM135A	FAM135A	chr6:7112310 shneg	shCHD1	OK	18,5808	13,4915	-0,461763	-2,51123	5,00E-05	0,0004193	yes
KDM5D	KDM5D	KDM5D	chrY:2186730 shneg	shCHD1	OK	4,64739	3,37443	-0,461779	-2,2343	5,00E-05	0,0004193	yes
ESPN	ESPN	ESPN	chr1:6484847 shneg	shCHD1	OK	2,43716	1,76925	-0,462064	-1,8097	0,0016	0,00862477	yes
BCAR3	BCAR3	BCAR3	chr1:9402734 shneg	shCHD1	OK	46,3992	33,6748	-0,462431	-2,54328	5,00E-05	0,0004193	yes
LOC283174	LOC283174	LOC283174	chr11:133766 shneg	shCHD1	OK	2,63058	1,90916	-0,462442	-2,15166	0,0006	0,00377139	yes
TTC3P1	TTC3P1	TTC3P1	chrX:7496037 shneg	shCHD1	OK	2,03775	1,47887	-0,462488	-1,49341	0,01025	0,040618	yes
FBXO9	FBXO9	FBXO9	chr6:5292979 shneg	shCHD1	OK	14,8899	10,8045	-0,462709	-2,47224	5,00E-05	0,0004193	yes
LINC00346	LINC00346	LINC00346	chr13:111516 shneg	shCHD1	OK	2,76834	2,00858	-0,46285	-2,18027	0,00015	0,00112331	yes
HOGA1	HOGA1	HOGA1	chr10:993441 shneg	shCHD1	OK	3,07807	2,23299	-0,463052	-1,5143	0,00925	0,0374435	yes
KITLG	KITLG	KITLG	chr12:888865 shneg	shCHD1	OK	30,4757	22,0953	-0,46392	-2,57145	5,00E-05	0,0004193	yes
NAAA	NAAA	NAAA	chr4:7683180 shneg	shCHD1	OK	12,8307	9,30163	-0,464049	-2,23602	0,00015	0,00112331	yes
C3orf67	C3orf67	C3orf67	chr3:5872773 shneg	shCHD1	OK	12,4452	9,0211	-0,464209	-2,27581	5,00E-05	0,0004193	yes
TOM1L1	TOM1L1	TOM1L1	chr17:529780 shneg	shCHD1	OK	20,3183	14,7261	-0,464399	-1,7986	0,0014	0,00768951	yes
FAM207A	FAM207A	FAM207A	chr21:463599 shneg	shCHD1	OK	38,1154	27,6226	-0,464525	-2,24344	0,00015	0,00112331	yes
GSTT1	GSTT1	GSTT1	chr22:243761 shneg	shCHD1	OK	39,8313	28,8612	-0,46477	-2,32647	0,00025	0,00176854	yes
TPP1	TPP1	TPP1	chr11:663399 shneg	shCHD1	OK	54,8646	39,7523	-0,464837	-2,63762	5,00E-05	0,0004193	yes
SNX30	SNX30	SNX30	chr9:1155131 shneg	shCHD1	OK	6,90542	5,00321	-0,464873	-2,42553	5,00E-05	0,0004193	yes
PTPN4	PTPN4	PTPN4	chr2:1205172 shneg	shCHD1	OK	4,43207	3,21115	-0,464889	-2,195	5,00E-05	0,0004193	yes
TBC1D4	TBC1D4	TBC1D4	chr13:758588 shneg	shCHD1	OK	9,38915	6,80242	-0,464946	-2,44917	5,00E-05	0,0004193	yes
RAB13	RAB13	RAB13	chr1:1539541 shneg	shCHD1	OK	114,554	82,9862	-0,465089	-2,56267	5,00E-05	0,0004193	yes
ENTPD6	ENTPD6	ENTPD6	chr20:251763 shneg	shCHD1	OK	66,8783	48,4444	-0,46521	-2,62917	5,00E-05	0,0004193	yes
RGAG4	RGAG4	RGAG4	chrX:7113093 shneg	shCHD1	OK	5,54238	4,01398	-0,465471	-2,15709	0,0002	0,00144867	yes
TBC1D19	TBC1D19	TBC1D19	chr4:2658554 shneg	shCHD1	OK	2,95959	2,14297	-0,465785	-1,62607	0,0052	0,0231261	yes
MTCP1NB	MTCP1NB	MTCP1NB	chrX:1542898 shneg	shCHD1	OK	17,9567	13,0013	-0,465859	-1,843	0,0018	0,00951868	yes
CDC14A	CDC14A	CDC14A	chr1:1008180 shneg	shCHD1	OK	5,02863	3,64065	-0,465971	-2,02543	0,0005	0,00321686	yes
ALAD	ALAD	ALAD	chr9:1161485 shneg	shCHD1	OK	12,3145	8,91369	-0,466261	-2,35163	5,00E-05	0,0004193	yes
SLC30A7	SLC30A7	SLC30A7	chr1:1013616 shneg	shCHD1	OK	5,04575	3,65222	-0,466295	-2,35496	0,00015	0,00112331	yes
FAM214B	FAM214B	FAM214B	chr9:3510411 shneg	shCHD1	OK	7,14711	5,17232	-0,466547	-2,28281	0,00035	0,00236888	yes
TDRD3	TDRD3	TDRD3	chr13:609705 shneg	shCHD1	OK	7,1856	5,19982	-0,466647	-2,18595	5,00E-05	0,0004193	yes
LETMD1	LETMD1	LETMD1	chr12:514420 shneg	shCHD1	OK	49,2258	35,6079	-0,467217	-2,49074	5,00E-05	0,0004193	yes
PORCN	PORCN	PORCN	chrX:4836737 shneg	shCHD1	OK	33,5627	24,2732	-0,467492	-2,4309	5,00E-05	0,0004193	yes
IQCD	IQCD	IQCD	chr12:113633 shneg	shCHD1	OK	4,3016	3,11076	-0,467607	-1,53092	0,0079	0,0327888	yes
SP140L	SP140L	SP140L	chr2:2311918 shneg	shCHD1	OK	4,73307	3,42271	-0,467637	-2,08322	0,0004	0,00266202	yes
KCTD20	KCTD20	KCTD20	chr6:3635832 shneg	shCHD1	OK	23,8018	17,2012	-0,468564	-2,58756	5,00E-05	0,0004193	yes
TPD52L1	TPD52L1	TPD52L1	chr6:1254748 shneg	shCHD1	OK	8,4385	6,09769	-0,468723	-1,93387	0,0008	0,0048114	yes
ROGDI	ROGDI	ROGDI	chr16:484696 shneg	shCHD1	OK	15,526	11,2146	-0,469307	-2,27527	0,0001	0,00078473	yes
BICC1	BICC1	BICC1	chr10:602729 shneg	shCHD1	OK	16,9913	12,2667	-0,470041	-2,43499	5,00E-05	0,0004193	yes
SEL1L	SEL1L	SEL1L	chr14:819378 shneg	shCHD1	OK	15,5506	11,2265	-0,470065	-2,5161	5,00E-05	0,0004193	yes
OCEL1	OCEL1	OCEL1	chr19:173370 shneg	shCHD1	OK	13,121	9,46986	-0,47046	-2,09315	0,0008	0,0048114	yes
LFNG	LFNG	LFNG	chr7:2552162 shneg	shCHD1	OK	84,3648	60,8676	-0,470966	-2,60966	5,00E-05	0,0004193	yes
OLFML2B	OLFML2B	OLFML2B	chr1:1619529 shneg	shCHD1	OK	2,86635	2,06773	-0,471168	-1,88331	0,0011	0,0063157	yes
SLC2A13	SLC2A13	SLC2A13	chr12:401488 shneg	shCHD1	OK	2,48025	1,78873	-0,471549	-2,23679	0,00015	0,00112331	yes
USP32	USP32	USP32	chr17:582546 shneg	shCHD1	OK	9,12747	6,58195	-0,4717	-2,49739	5,00E-05	0,0004193	yes
LIAS	LIAS	LIAS	chr4:3946066 shneg	shCHD1	OK	6,86088	4,94703	-0,471831	-2,01553	0,0007	0,00428773	yes
PARP8	PARP8	PARP8	chr5:4996173 shneg	shCHD1	OK	1,01663	0,733006	-0,471895	-1,75454	0,00275	0,0136364	yes
C4orf52	C4orf52	C4orf52	chr4:2591581 shneg	shCHD1	OK	18,8908	13,6204	-0,471915	-2,19818	0,0001	0,00078473	yes

HEXA	HEXA	HEXA	chr15:726357	shneg	shCHD1	OK	36,4393	26,2713	-0,472009	-2,5367	5,00E-05	0,0004193	yes
SAP30BP	SAP30BP	SAP30BP	chr17:736633	shneg	shCHD1	OK	25,4795	18,3662	-0,47228	-2,4949	5,00E-05	0,0004193	yes
SLC26A11	SLC26A11	SLC26A11	chr17:781941	shneg	shCHD1	OK	7,49498	5,40074	-0,472767	-2,28063	5,00E-05	0,0004193	yes
MOSPD1	MOSPD1	MOSPD1	chrX:1340216	shneg	shCHD1	OK	70,0806	50,4776	-0,473372	-2,65575	5,00E-05	0,0004193	yes
SLC7A7	SLC7A7	SLC7A7	chr14:232424	shneg	shCHD1	OK	11,969	8,6188	-0,473745	-2,24572	5,00E-05	0,0004193	yes
THAP8	THAP8	THAP8	chr19:365258	shneg	shCHD1	OK	4,40694	3,17337	-0,473762	-1,77902	0,0025	0,012594	yes
SYNM	SYNM	SYNM	chr15:996452	shneg	shCHD1	OK	3,5641	2,56612	-0,47395	-2,26698	5,00E-05	0,0004193	yes
CDHR3	CDHR3	CDHR3	chr7:1056036	shneg	shCHD1	OK	0,981892	0,706929	-0,473999	-1,68603	0,00525	0,0232593	yes
KCTD18	KCTD18	KCTD18	chr2:2013536	shneg	shCHD1	OK	7,75434	5,58052	-0,474606	-2,31094	5,00E-05	0,0004193	yes
MAMDC4	MAMDC4	MAMDC4	chr9:1397468	shneg	shCHD1	OK	4,53897	3,26593	-0,474871	-2,1266	0,0003	0,0020738	yes
CCDC53	CCDC53	CCDC53	chr12:102406	shneg	shCHD1	OK	21,1526	15,2169	-0,475159	-2,23801	0,00015	0,00112331	yes
HNF1A-AS1	HNF1A-AS1	HNF1A-AS1	chr12:121407	shneg	shCHD1	OK	13,3216	9,58307	-0,475207	-2,33599	5,00E-05	0,0004193	yes
ABTB1	ABTB1	ABTB1	chr3:1273917	shneg	shCHD1	OK	19,0388	13,6909	-0,475727	-2,36726	0,0001	0,00078473	yes
ABLIM1	ABLIM1	ABLIM1	chr10:116190	shneg	shCHD1	OK	1,88899	1,35791	-0,476226	-2,15185	0,00025	0,00176854	yes
MRPL28	MRPL28	MRPL28	chr16:417383	shneg	shCHD1	OK	38,5678	27,7243	-0,476243	-2,40177	5,00E-05	0,0004193	yes
LOC10050619	LOC10050619	LOC10050619	chr9:1322509	shneg	shCHD1	OK	11,771	8,46142	-0,476271	-2,28659	5,00E-05	0,0004193	yes
C17orf101	C17orf101	C17orf101	chr17:803470	shneg	shCHD1	OK	9,21183	6,61909	-0,476854	-1,51972	0,008	0,0331249	yes
TRPC6	TRPC6	TRPC6	chr11:101322	shneg	shCHD1	OK	2,23962	1,60893	-0,477155	-2,00437	0,00065	0,0040329	yes
NHLRC3	NHLRC3	NHLRC3	chr13:396124	shneg	shCHD1	OK	7,74121	5,56049	-0,477348	-2,32774	0,0001	0,00078473	yes
TRIM45	TRIM45	TRIM45	chr1:1176536	shneg	shCHD1	OK	3,92386	2,81753	-0,477842	-2,20648	0,00025	0,00176854	yes
SYPL1	SYPL1	SYPL1	chr7:1057309	shneg	shCHD1	OK	35,6682	25,6034	-0,478302	-2,55703	5,00E-05	0,0004193	yes
SPON2	SPON2	SPON2	chr4:1160720	shneg	shCHD1	OK	4,8018	3,44678	-0,478325	-1,59287	0,00835	0,0343596	yes
HIPK3	HIPK3	HIPK3	chr11:332791	shneg	shCHD1	OK	11,35	8,14517	-0,478678	-2,42539	5,00E-05	0,0004193	yes
RFX5	RFX5	RFX5	chr1:1513131	shneg	shCHD1	OK	33,072	23,7333	-0,478698	-2,63244	5,00E-05	0,0004193	yes
SGK2	SGK2	SGK2	chr20:421876	shneg	shCHD1	OK	33,9649	24,3689	-0,479007	-2,50386	5,00E-05	0,0004193	yes
GBP4	GBP4	GBP4	chr1:8964683	shneg	shCHD1	OK	1,08615	0,779197	-0,47917	-1,73341	0,00335	0,0160296	yes
C5orf41	C5orf41	C5orf41	chr5:1724833	shneg	shCHD1	OK	4,23462	3,03779	-0,479211	-1,95888	0,0005	0,00321686	yes
PFKFB2	PFKFB2	PFKFB2	chr1:2072266	shneg	shCHD1	OK	4,83014	3,46491	-0,479249	-2,11573	0,00025	0,00176854	yes
C17orf56	C17orf56	C17orf56	chr17:792020	shneg	shCHD1	OK	9,17114	6,57632	-0,479821	-2,2576	0,0001	0,00078473	yes
TMEM221	TMEM221	TMEM221	chr19:175463	shneg	shCHD1	OK	3,39153	2,43131	-0,480204	-1,67792	0,0038	0,0178336	yes
DYRK1B	DYRK1B	DYRK1B	chr19:403159	shneg	shCHD1	OK	18,3559	13,1536	-0,480785	-2,43377	5,00E-05	0,0004193	yes
INO80B	INO80B	INO80B	chr2:7468214	shneg	shCHD1	OK	23,4069	16,7679	-0,481232	-1,43808	0,01165	0,0451385	yes
GABRB3	GABRB3	GABRB3	chr15:267886	shneg	shCHD1	OK	3,32655	2,38241	-0,481607	-2,27563	0,00015	0,00112331	yes
TBC1D1	TBC1D1	TBC1D1	chr4:3789270	shneg	shCHD1	OK	5,93582	4,25015	-0,481932	-2,31884	5,00E-05	0,0004193	yes
ITGA6	ITGA6	ITGA6	chr2:1732923	shneg	shCHD1	OK	72,5592	51,9425	-0,482241	-2,82294	5,00E-05	0,0004193	yes
AHNAK	AHNAK	AHNAK	chr11:622010	shneg	shCHD1	OK	109,847	78,6207	-0,482507	-2,65242	5,00E-05	0,0004193	yes
DNASE2	DNASE2	DNASE2	chr19:129860	shneg	shCHD1	OK	42,8163	30,6327	-0,483086	-2,60099	5,00E-05	0,0004193	yes
NLGN1	NLGN1	NLGN1	chr3:1731162	shneg	shCHD1	OK	14,7015	10,5168	-0,483267	-2,54537	5,00E-05	0,0004193	yes
ZNF791	ZNF791	ZNF791	chr19:127217	shneg	shCHD1	OK	7,62434	5,45102	-0,484088	-2,28799	5,00E-05	0,0004193	yes
BBS7	BBS7	BBS7	chr4:1227454	shneg	shCHD1	OK	8,00435	5,72263	-0,484107	-2,22843	0,0002	0,00144867	yes
RTCD1	RTCD1	RTCD1	chr1:1007317	shneg	shCHD1	OK	21,9034	15,6592	-0,484142	-2,52847	5,00E-05	0,0004193	yes
ACTG1	ACTG1	ACTG1	chr17:794769	shneg	shCHD1	OK	942,628	673,774	-0,484425	-2,93714	5,00E-05	0,0004193	yes
LYST	LYST	LYST	chr1:2358243	shneg	shCHD1	OK	10,2382	7,3152	-0,484992	-2,67111	5,00E-05	0,0004193	yes
RAB32	RAB32	RAB32	chr6:1468648	shneg	shCHD1	OK	34,9711	24,9799	-0,485394	-2,4375	5,00E-05	0,0004193	yes
N4BP2	N4BP2	N4BP2	chr4:4004453	shneg	shCHD1	OK	7,49473	5,35252	-0,485658	-2,48271	0,0001	0,00078473	yes
DENND5B	DENND5B	DENND5B	chr12:315351	shneg	shCHD1	OK	1,67714	1,19769	-0,485749	-2,30172	5,00E-05	0,0004193	yes
CYP2U1	CYP2U1	CYP2U1	chr4:1088527	shneg	shCHD1	OK	3,56617	2,5455	-0,486427	-2,29038	5,00E-05	0,0004193	yes
IGF2R	IGF2R	IGF2R	chr6:1603901	shneg	shCHD1	OK	36,5758	26,1055	-0,486534	-2,81	5,00E-05	0,0004193	yes
NLRC5	NLRC5	NLRC5	chr16:570509	shneg	shCHD1	OK	3,76049	2,68395	-0,486563	-2,37986	0,00015	0,00112331	yes
SOCS3	SOCS3	SOCS3	chr17:763528	shneg	shCHD1	OK	16,0348	11,4431	-0,486725	-2,47701	5,00E-05	0,0004193	yes
TBC1D10A	TBC1D10A	TBC1D10A	chr22:306879	shneg	shCHD1	OK	5,4746	3,90626	-0,486964	-2,03453	0,00035	0,00236888	yes
DOCK3	DOCK3	DOCK3	chr3:5071267	shneg	shCHD1	OK	5,73568	4,09078	-0,487587	-2,53471	5,00E-05	0,0004193	yes
CRIPAK	CRIPAK	CRIPAK	chr4:1385339	shneg	shCHD1	OK	2,57822	1,83775	-0,488438	-2,11983	0,00045	0,00293585	yes
CHIC1	CHIC1	CHIC1	chrX:7278298	shneg	shCHD1	OK	3,42512	2,44019	-0,489161	-2,31194	5,00E-05	0,0004193	yes
ZNF585B	ZNF585B	ZNF585B	chr19:376724	shneg	shCHD1	OK	2,29887	1,63767	-0,489281	-2,28775	0,0002	0,00144867	yes
USP47	USP47	USP47	chr11:118629	shneg	shCHD1	OK	14,8233	10,5588	-0,489426	-2,6923	5,00E-05	0,0004193	yes
ZNF383	ZNF383	ZNF383	chr19:377173	shneg	shCHD1	OK	2,87364	2,04652	-0,489703	-1,50469	0,0113	0,044015	yes
NGFRAP1	NGFRAP1	NGFRAP1	chrX:1026312	shneg	shCHD1	OK	179,858	128,058	-0,490063	-2,73356	5,00E-05	0,0004193	yes
BRPF3	BRPF3	BRPF3	chr6:3616454	shneg	shCHD1	OK	17,3605	12,3588	-0,490268	-2,68582	5,00E-05	0,0004193	yes
CHPT1	CHPT1	CHPT1	chr12:102091	shneg	shCHD1	OK	15,4344	10,9843	-0,490713	-2,29768	5,00E-05	0,0004193	yes
SNX7	SNX7	SNX7	chr1:9912723	shneg	shCHD1	OK	42,2955	30,0966	-0,490904	-2,61724	5,00E-05	0,0004193	yes
ACOX3	ACOX3	ACOX3	chr4:8368008	shneg	shCHD1	OK	3,18135	2,26378	-0,490905	-1,95897	0,00075	0,00455593	yes
LOC387646	LOC387646	LOC387646	chr10:275347	shneg	shCHD1	OK	1,20112	0,854506	-0,491218	-1,87251	0,002	0,0104181	yes
RNPC3	RNPC3	RNPC3	chr1:1040685	shneg	shCHD1	OK	14,4782	10,2978	-0,491547	-1,97211	0,0008	0,0048114	yes
GSDMD	GSDMD	GSDMD	chr8:1446355	shneg	shCHD1	OK	31,5183	22,4121	-0,49191	-2,55014	5,00E-05	0,0004193	yes
ZNF467	ZNF467	ZNF467	chr7:1494614	shneg	shCHD1	OK	27,8665	19,8067	-0,492545	-2,58689	5,00E-05	0,0004193	yes
EFNB3	EFNB3	EFNB3	chr17:760851	shneg	shCHD1	OK	7,09503	5,04255	-0,492656	-2,37174	5,00E-05	0,0004193	yes
VPS37D	VPS37D	VPS37D	chr7:7308217	shneg	shCHD1	OK	13,0461	9,26525	-0,493716	-2,38248	5,00E-05	0,0004193	yes
TRIB1	TRIB1	TRIB1	chr8:1264425	shneg	shCHD1	OK	71,8384	51,0171	-0,493772	-2,83759	5,00E-05	0,0004193	yes
TAPT1	TAPT1	TAPT1	chr4:1616212	shneg	shCHD1	OK	2,13746	1,51794	-0,493779	-2,03313	0,0005	0,00321686	yes
ARMCX3	ARMCX3	ARMCX3	chrX:1008781	shneg	shCHD1	OK	28,3571	20,1374	-0,493836	-2,68693	5,00E-05	0,0004193	yes
WDR19	WDR19	WDR19	chr4:3918402	shneg	shCHD1	OK	9,62841	6,8374	-0,493849	-2,52306	5,00E-05	0,0004193	yes
NF1	NF1	NF1	chr17:294219	shneg	shCHD1	OK	14,5607	10,3391	-0,49397	-2,74824	5,00E-05	0,0004193	yes
CA5B	CA5B	CA5B	chrX:1575641	shneg	shCHD1	OK	4,93835	3,50648	-0,494006	-2,23631	5,00E-05	0,0004193	yes
CAT	CAT	CAT	chr11:344604	shneg	shCHD1	OK	22,5787	16,0317	-0,494035	-2,55405	0,0001	0,00078473	yes
POU4F1	POU4F1	POU4F1	chr13:791732	shneg	shCHD1	OK	3,40762	2,41857	-0,494612	-2,23323	0,00015	0,00112331	yes
Sep 09	Sep 09	Sep 09	chr17:752774	shneg	shCHD1	OK	74,8954	53,1519	-0,494757	-2,84569	5,00E-05	0,0004193	yes
SLC25A42	SLC25A42	SLC25A42	chr19:191748	shneg	shCHD1	OK	3,96142	2,81111	-0,494879	-2,19193	0,0002	0,00144867	yes
STARD8	STARD8	STARD8	chrX:6786751	shneg	shCHD1	OK	2,01742	1,43138	-0,495106	-2,05348	0,00035	0,00236888	yes
AIFM3	AIFM3	AIFM3	chr22:213194	shneg	shCHD1	OK	3,50386	2,48548	-0,495418	-1,87322	0,00085	0,00508358	yes
C1orf115	C1orf115	C1orf115	chr1:2208636	shneg	shCHD1	OK	8,66475	6,14461	-0,495837	-2,36402	5,00E-05	0,0004193	yes
JAK2	JAK2	JAK2	chr9:4985244	shneg	shCHD1	OK	3,10135	2,19888	-0,496127	-2,33342	5,00E-05	0,0004193	yes
TMEM38B	TMEM38B	TMEM38B	chr9:1084568	shneg	shCHD1	OK	7,15699	5,07398	-0,496236	-2,30061	5,00E-05	0,0004193	yes
PPL	PPL	PPL	chr16:493250	shneg	shCHD1	OK	1,071	0,759155	-0,496489	-1,78958	0,00255	0,0127904	yes
MPG	MPG	MPG	chr16:127017	shneg	shCHD1	OK	38,5548	27,3216	-0,496868	-1,76663	0,0034	0,0162243	yes
SERTAD3	SERTAD3	SERTAD3	chr19:409467	shneg	shCHD1	OK	28,7116	20,3431	-0,497096	-2,49208	5,00E-05	0,0004193	yes
IER5L	IER5L	IER5L	chr9:1319378	shneg	shCHD1	OK	55,4168	39,2423	-0,497914	-2,76551	5,00E-05	0,0004193	yes
PCYT2	PCYT2	PCYT2	chr17:798600	shneg	shCHD1	OK	7,71837	5,46347	-0,498478	-2,20855	5,00E-05	0,0004193	yes
ARID3A	ARID3A	ARID3A	chr19:926036	shneg	shCHD1	OK	15,9732	11,3049	-0,498708	-2,54468	5,00E-05	0,0004193	yes
FAM184A	FAM184A	FAM184A	chr6:1192809	shneg	shCHD1	OK	1,32911	0,940188	-0,499434	-1,53835	0,008	0,0331249	yes
MANSC1	MANSC1	MANSC1	chr12:124822	shneg	shCHD1	OK	6,19265	4,37889	-0,499994	-2,29269	5,00E-05	0,0004193	yes
APLP1	APLP1	APLP1	chr19:363594	shneg	shCHD1	OK	47,7841	33,7852	-0,500139	-2,73072	5,00E-05	0,0004193	yes
SLC27A2	SLC27A2	SLC27A2	chr15:504743	shneg	shCHD1	OK	1,99386	1,40923	-0,500663	-1,53681	0,00595	0,0259073	yes

CXXC5	CXXC5	CXXC5	chr5:1390283	shneg	shCHD1	OK	72,2932	51,083	-0,501016	-2,71935	5,00E-05	0,0004193	yes
ACTG2	ACTG2	ACTG2	chr2:7412009	shneg	shCHD1	OK	4,83264	3,41468	-0,501059	-1,60332	0,00545	0,0240688	yes
DVL1	DVL1	DVL1	chr1:1270657	shneg	shCHD1	OK	26,112	18,447	-0,501326	-2,67621	5,00E-05	0,0004193	yes
PEX16	PEX16	PEX16	chr11:459312	shneg	shCHD1	OK	8,25148	5,82851	-0,501525	-2,35031	5,00E-05	0,0004193	yes
FXYD2	FXYD2	FXYD2	chr11:117690	shneg	shCHD1	OK	655,108	462,625	-0,501888	-2,31949	5,00E-05	0,0004193	yes
RHOH	RHOH	RHOH	chr4:4019852	shneg	shCHD1	OK	8,56112	6,04499	-0,502061	-2,36625	5,00E-05	0,0004193	yes
AFAP1	AFAP1	AFAP1	chr4:7755816	shneg	shCHD1	OK	8,3204	5,87469	-0,502139	-2,62945	5,00E-05	0,0004193	yes
ARMCX6	ARMCX6	ARMCX6	chrX:1008701	shneg	shCHD1	OK	43,9722	31,0306	-0,502899	-2,3589	0,0001	0,00078473	yes
CTBS	CTBS	CTBS	chr1:8501880	shneg	shCHD1	OK	6,24225	4,40417	-0,503196	-2,39519	5,00E-05	0,0004193	yes
KIAA1009	KIAA1009	KIAA1009	chr6:8483395	shneg	shCHD1	OK	3,67957	2,59558	-0,50348	-2,39381	5,00E-05	0,0004193	yes
ARMCX4	ARMCX4	ARMCX4	chrX:1006732	shneg	shCHD1	OK	7,71953	5,44281	-0,50416	-2,26001	0,00015	0,00112331	yes
MARCH2	MARCH2	MARCH2	chr19:847818	shneg	shCHD1	OK	13,4621	9,4876	-0,504791	-2,33895	5,00E-05	0,0004193	yes
PEX10	PEX10	PEX10	chr1:2323213	shneg	shCHD1	OK	35,3157	24,8677	-0,506038	-1,71692	0,0023	0,0117566	yes
TMEM61	TMEM61	TMEM61	chr1:5544646	shneg	shCHD1	OK	6,50772	4,58242	-0,506041	-1,75391	0,002	0,0104181	yes
IL12A	IL12A	IL12A	chr3:1597066	shneg	shCHD1	OK	6,35623	4,47491	-0,506313	-1,96204	0,0008	0,0048114	yes
MESP1	MESP1	MESP1	chr15:902930	shneg	shCHD1	OK	5,46236	3,84343	-0,50713	-1,68475	0,00365	0,0172576	yes
TANK	TANK	TANK	chr2:1619934	shneg	shCHD1	OK	21,4895	15,1105	-0,508083	-1,61664	0,00595	0,0259073	yes
C7orf63	C7orf63	C7orf63	chr7:8987448	shneg	shCHD1	OK	2,03691	1,43225	-0,508097	-1,92709	0,00075	0,00455593	yes
PERP	PERP	PERP	chr6:1384096	shneg	shCHD1	OK	17,5165	12,3154	-0,508257	-2,71718	5,00E-05	0,0004193	yes
PREPL	PREPL	PREPL	chr2:4450259	shneg	shCHD1	OK	37,0673	26,0584	-0,508396	-1,79633	0,00205	0,0106507	yes
ARSD	ARSD	ARSD	chrX:2822010	shneg	shCHD1	OK	13,9672	9,81652	-0,508763	-2,71295	5,00E-05	0,0004193	yes
CCDC71L	CCDC71L	CCDC71L	chr7:1062972	shneg	shCHD1	OK	5,20387	3,65738	-0,508775	-2,45491	5,00E-05	0,0004193	yes
GAS6	GAS6	GAS6	chr13:114523	shneg	shCHD1	OK	58,2301	40,9149	-0,50914	-2,71721	5,00E-05	0,0004193	yes
TMEM59	TMEM59	TMEM59	chr1:5449734	shneg	shCHD1	OK	148,587	104,385	-0,509389	-2,91274	5,00E-05	0,0004193	yes
PRX	PRX	PRX	chr19:408996	shneg	shCHD1	OK	3,59782	2,52745	-0,509442	-2,39785	5,00E-05	0,0004193	yes
IRS1	IRS1	IRS1	chr2:2275960	shneg	shCHD1	OK	14,0985	9,89804	-0,510331	-2,82547	5,00E-05	0,0004193	yes
CELSR2	CELSR2	CELSR2	chr1:1097926	shneg	shCHD1	OK	8,29615	5,82416	-0,510392	-2,7631	5,00E-05	0,0004193	yes
SLC9A6	SLC9A6	SLC9A6	chrX:1350675	shneg	shCHD1	OK	8,9755	6,30048	-0,51053	-2,58144	5,00E-05	0,0004193	yes
SGMS2	SGMS2	SGMS2	chr4:1087457	shneg	shCHD1	OK	3,53383	2,47995	-0,510925	-2,43997	5,00E-05	0,0004193	yes
SERPINE1	SERPINE1	SERPINE1	chr7:1007703	shneg	shCHD1	OK	52,2099	36,6347	-0,511113	-2,87811	5,00E-05	0,0004193	yes
WIPI1	WIPI1	WIPI1	chr17:664174	shneg	shCHD1	OK	11,3062	7,93066	-0,511601	-2,46639	5,00E-05	0,0004193	yes
KIF27	KIF27	KIF27	chr9:8645161	shneg	shCHD1	OK	3,50235	2,45538	-0,512374	-2,37635	5,00E-05	0,0004193	yes
MKS1	MKS1	MKS1	chr17:562827	shneg	shCHD1	OK	5,24213	3,6719	-0,513626	-2,26436	0,0001	0,00078473	yes
C19orf26	C19orf26	C19orf26	chr19:122994	shneg	shCHD1	OK	4,17905	2,92717	-0,513668	-2,15207	0,00025	0,00176854	yes
NR2F2	NR2F2	NR2F2	chr15:968691	shneg	shCHD1	OK	80,7098	56,5285	-0,513766	-2,92182	5,00E-05	0,0004193	yes
C17orf70	C17orf70	C17orf70	chr17:795069	shneg	shCHD1	OK	12,3624	8,65822	-0,513816	-2,62028	5,00E-05	0,0004193	yes
SP100	SP100	SP100	chr2:2312808	shneg	shCHD1	OK	18,2131	12,7539	-0,514032	-2,19778	0,00015	0,00112331	yes
TCP11L2	TCP11L2	TCP11L2	chr12:106696	shneg	shCHD1	OK	2,20855	1,54527	-0,515245	-1,60029	0,00685	0,0290629	yes
RAB17	RAB17	RAB17	chr2:2384829	shneg	shCHD1	OK	66,5909	46,5904	-0,515294	-2,86268	5,00E-05	0,0004193	yes
MATN3	MATN3	MATN3	chr2:2019181	shneg	shCHD1	OK	6,1599	4,30863	-0,515679	-2,44987	5,00E-05	0,0004193	yes
USP51	USP51	USP51	chrX:5551104	shneg	shCHD1	OK	1,86219	1,30236	-0,515873	-1,98808	0,00095	0,00559279	yes
MAN1B1	MAN1B1	MAN1B1	chr9:1399813	shneg	shCHD1	OK	44,7754	31,3135	-0,515923	-2,85903	5,00E-05	0,0004193	yes
CTAGE5	CTAGE5	CTAGE5	chr14:397344	shneg	shCHD1	OK	12,8204	8,96314	-0,516363	-2,61676	5,00E-05	0,0004193	yes
SFXN5	SFXN5	SFXN5	chr2:7316916	shneg	shCHD1	OK	15,9613	11,1585	-0,516429	-2,72936	5,00E-05	0,0004193	yes
FAM63A	FAM63A	FAM63A	chr1:1509693	shneg	shCHD1	OK	8,13335	5,68533	-0,516607	-2,35859	5,00E-05	0,0004193	yes
CD151	CD151	CD151	chr11:832951	shneg	shCHD1	OK	197,061	137,682	-0,517304	-2,96942	5,00E-05	0,0004193	yes
PDE4A	PDE4A	PDE4A	chr19:105274	shneg	shCHD1	OK	21,2067	14,8142	-0,517539	-2,79295	5,00E-05	0,0004193	yes
MALL	MALL	MALL	chr2:1108414	shneg	shCHD1	OK	45,3514	31,6786	-0,517638	-2,87941	5,00E-05	0,0004193	yes
RSPH3	RSPH3	RSPH3	chr6:1593982	shneg	shCHD1	OK	10,3573	7,23196	-0,518187	-2,44421	5,00E-05	0,0004193	yes
CHST3	CHST3	CHST3	chr10:737241	shneg	shCHD1	OK	34,9555	24,393	-0,519049	-2,97395	5,00E-05	0,0004193	yes
TAOK3	TAOK3	TAOK3	chr12:118587	shneg	shCHD1	OK	3,46005	2,4145	-0,519067	-2,45423	5,00E-05	0,0004193	yes
HBXIP	HBXIP	HBXIP	chr1:1109438	shneg	shCHD1	OK	60,3914	42,1337	-0,519368	-2,63496	5,00E-05	0,0004193	yes
FGFR3	FGFR3	FGFR3	chr4:1795038	shneg	shCHD1	OK	9,47093	6,60723	-0,51946	-2,6112	5,00E-05	0,0004193	yes
BTBD19	BTBD19	BTBD19	chr1:4527415	shneg	shCHD1	OK	14,8221	10,3358	-0,520104	-2,5237	5,00E-05	0,0004193	yes
LOC654433	LOC654433	LOC654433	chr2:1139735	shneg	shCHD1	OK	91,4927	63,7867	-0,520401	-2,80992	5,00E-05	0,0004193	yes
CPT1C	CPT1C	CPT1C	chr19:501943	shneg	shCHD1	OK	2,27641	1,58686	-0,520582	-1,75445	0,0017	0,00909693	yes
SPATA20	SPATA20	SPATA20	chr17:486245	shneg	shCHD1	OK	63,7598	44,4424	-0,520712	-2,92164	5,00E-05	0,0004193	yes
CLCN3	CLCN3	CLCN3	chr4:1705416	shneg	shCHD1	OK	10,6373	7,40891	-0,521799	-2,76615	5,00E-05	0,0004193	yes
C16orf5	C16orf5	C16orf5	chr16:456067	shneg	shCHD1	OK	3,90045	2,71605	-0,522132	-2,11103	0,00065	0,0040329	yes
C5orf38	C5orf38	C5orf38	chr5:2752261	shneg	shCHD1	OK	4,94163	3,44011	-0,522534	-1,45989	0,01245	0,0476418	yes
FAM169A	FAM169A	FAM169A	chr5:7407339	shneg	shCHD1	OK	5,56307	3,8716	-0,522951	-2,5659	5,00E-05	0,0004193	yes
SPRY1	SPRY1	SPRY1	chr4:1243179	shneg	shCHD1	OK	65,9294	45,8823	-0,522985	-2,91342	5,00E-05	0,0004193	yes
NCS1	NCS1	NCS1	chr9:1329348	shneg	shCHD1	OK	53,9421	37,5211	-0,52371	-2,99565	5,00E-05	0,0004193	yes
COL4A1	COL4A1	COL4A1	chr13:110801	shneg	shCHD1	OK	11,78	8,19389	-0,523715	-2,81004	5,00E-05	0,0004193	yes
OBFC1	OBFC1	OBFC1	chr10:105637	shneg	shCHD1	OK	11,4706	7,97705	-0,524008	-2,80179	5,00E-05	0,0004193	yes
STC2	STC2	STC2	chr5:1727417	shneg	shCHD1	OK	0,875577	0,608717	-0,524463	-1,62965	0,00525	0,0232593	yes
PPARG	PPARG	PPARG	chr3:1232934	shneg	shCHD1	OK	18,2805	12,7082	-0,524553	-2,57675	5,00E-05	0,0004193	yes
KIF5A	KIF5A	KIF5A	chr12:579438	shneg	shCHD1	OK	2,19283	1,5243	-0,524647	-2,04813	0,0004	0,00266202	yes
NAGPA	NAGPA	NAGPA	chr16:507484	shneg	shCHD1	OK	5,45885	3,79445	-0,524706	-2,23973	0,0001	0,00078473	yes
LOC339535	LOC339535	LOC339535	chr1:2386436	shneg	shCHD1	OK	3,6351	2,52613	-0,525066	-1,68891	0,00405	0,0187979	yes
LRRC37B	LRRC37B	LRRC37B	chr17:303481	shneg	shCHD1	OK	4,1474	2,88189	-0,525195	-2,21125	0,00015	0,00112331	yes
HEATR6	HEATR6	HEATR6	chr17:581205	shneg	shCHD1	OK	9,32691	6,47972	-0,525468	-2,62838	5,00E-05	0,0004193	yes
ZNF773	ZNF773	ZNF773	chr19:580113	shneg	shCHD1	OK	2,40135	1,6676	-0,526075	-1,68147	0,0036	0,0170733	yes
CEP44	CEP44	CEP44	chr4:1751578	shneg	shCHD1	OK	10,1718	7,06232	-0,52636	-2,08103	0,00035	0,00236888	yes
ZNF25	ZNF25	ZNF25	chr10:382387	shneg	shCHD1	OK	2,16229	1,5006	-0,527028	-2,01248	0,0006	0,00377139	yes
ERBB3	ERBB3	ERBB3	chr12:564738	shneg	shCHD1	OK	7,24518	5,02684	-0,527372	-1,6246	0,0041	0,0189794	yes
SLC16A3	SLC16A3	SLC16A3	chr17:801862	shneg	shCHD1	OK	46,4688	32,2243	-0,528116	-2,84894	5,00E-05	0,0004193	yes
RNF135	RNF135	RNF135	chr17:292979	shneg	shCHD1	OK	8,98026	6,2258	-0,528497	-2,39237	5,00E-05	0,0004193	yes
CCDC57	CCDC57	CCDC57	chr17:800593	shneg	shCHD1	OK	11,7429	8,14049	-0,528602	-2,62412	5,00E-05	0,0004193	yes
DLG1	DLG1	DLG1	chr3:1967694	shneg	shCHD1	OK	17,301	11,9886	-0,529194	-2,82139	5,00E-05	0,0004193	yes
ZNF816	ZNF816	ZNF816	chr19:534303	shneg	shCHD1	OK	3,4276	2,37507	-0,529231	-1,78828	0,00245	0,0123735	yes
CPT1A	CPT1A	CPT1A	chr11:685220	shneg	shCHD1	OK	20,0411	13,8863	-0,529294	-2,68033	5,00E-05	0,0004193	yes
AICDA	AICDA	AICDA	chr12:875476	shneg	shCHD1	OK	3,17027	2,1964	-0,529464	-2,07622	0,0006	0,00377139	yes
LOC10028776	LOC10028776	LOC10028776	chrX:1020240	shneg	shCHD1	OK	2,97476	2,06041	-0,529842	-1,79946	0,00165	0,00886	yes
MT1F	MT1F	MT1F	chr16:566918	shneg	shCHD1	OK	56,5555	39,1712	-0,529874	-2,45437	5,00E-05	0,0004193	yes
STK17B	STK17B	STK17B	chr2:1969983	shneg	shCHD1	OK	1,34624	0,932156	-0,530291	-1,94711	0,00125	0,00702633	yes
ZNF836	ZNF836	ZNF836	chr19:526581	shneg	shCHD1	OK	1,58534	1,09731	-0,530825	-1,71666	0,00295	0,0144329	yes
FCGRT	FCGRT	FCGRT	chr19:500155	shneg	shCHD1	OK	44,6849	30,9262	-0,530955	-2,80294	5,00E-05	0,0004193	yes
C14orf93	C14orf93	C14orf93	chr14:234561	shneg	shCHD1	OK	5,78041	4,00055	-0,530974	-2,32169	5,00E-05	0,0004193	yes
FCHO1	FCHO1	FCHO1	chr19:178585	shneg	shCHD1	OK	6,57643	4,55124	-0,531045	-2,49513	5,00E-05	0,0004193	yes
ATP11C	ATP11C	ATP11C	chrX:1388085	shneg	shCHD1	OK	5,6172	3,88668	-0,531312	-2,63874	5,00E-05	0,0004193	yes
PRKACB	PRKACB	PRKACB	chr1:8454374	shneg	shCHD1	OK	23,318	16,1273	-0,53194	-2,82233	5,00E-05	0,0004193	yes
IMMP2L	IMMP2L	IMMP2L	chr7:1103031	shneg	shCHD1	OK	7,58494	5,23993	-0,533589	-1,9597	0,0007	0,00428773	yes

HSPA4L	HSPA4L	HSPA4L	chr4:1287034	shneg	shCHD1	OK	3,27136	2,25886	-0,534294	-2,24172	0,00035	0,00236888	yes
CEND1	CEND1	CEND1	chr11:787109	shneg	shCHD1	OK	10,5504	7,28253	-0,534787	-2,49557	5,00E-05	0,0004193	yes
MYL5	MYL5	MYL5	chr4:671710-(	shneg	shCHD1	OK	11,6297	8,02658	-0,534958	-1,72829	0,0033	0,0158557	yes
SIGIRR	SIGIRR	SIGIRR	chr11:405715	shneg	shCHD1	OK	84,3212	58,1762	-0,535466	-2,96601	5,00E-05	0,0004193	yes
APOL6	APOL6	APOL6	chr22:360444	shneg	shCHD1	OK	4,77562	3,29304	-0,536267	-2,77187	5,00E-05	0,0004193	yes
SSPO	SSPO	SSPO	chr7:1494731	shneg	shCHD1	OK	3,41313	2,35274	-0,536754	-2,79481	5,00E-05	0,0004193	yes
CDCP1	CDCP1	CDCP1	chr3:4512376	shneg	shCHD1	OK	105,559	72,75	-0,537032	-3,03764	5,00E-05	0,0004193	yes
SESN1	SESN1	SESN1	chr6:1093076	shneg	shCHD1	OK	3,19544	2,20201	-0,537189	-2,13346	0,0004	0,00266202	yes
KAZALD1	KAZALD1	KAZALD1	chr10:102820	shneg	shCHD1	OK	11,7926	8,12578	-0,537303	-2,60078	5,00E-05	0,0004193	yes
SCD5	SCD5	SCD5	chr4:8355068	shneg	shCHD1	OK	20,5994	14,178	-0,538948	-2,66287	5,00E-05	0,0004193	yes
SSNA1	SSNA1	SSNA1	chr9:1400692	shneg	shCHD1	OK	48,0632	33,0659	-0,539589	-1,85789	0,00125	0,00702633	yes
ACBD4	ACBD4	ACBD4	chr17:432099	shneg	shCHD1	OK	7,82866	5,38538	-0,539718	-2,33318	0,0001	0,00078473	yes
ZNF75D	ZNF75D	ZNF75D	chrX:1344197	shneg	shCHD1	OK	8,97977	6,17183	-0,54098	-2,61767	5,00E-05	0,0004193	yes
B4GALT1	B4GALT1	B4GALT1	chr9:3311063	shneg	shCHD1	OK	17,7927	12,2207	-0,541953	-2,89319	5,00E-05	0,0004193	yes
ARRDC2	ARRDC2	ARRDC2	chr19:181119	shneg	shCHD1	OK	18,5338	12,7248	-0,542521	-2,75939	5,00E-05	0,0004193	yes
CHD1	CHD1	CHD1	chr5:9819090	shneg	shCHD1	OK	11,1849	7,67829	-0,542694	-2,8846	5,00E-05	0,0004193	yes
DOK4	DOK4	DOK4	chr16:574965	shneg	shCHD1	OK	223,781	153,594	-0,542971	-2,73318	5,00E-05	0,0004193	yes
DLL1	DLL1	DLL1	chr6:1705912	shneg	shCHD1	OK	4,58791	3,14887	-0,543003	-2,55068	5,00E-05	0,0004193	yes
MED13	MED13	MED13	chr17:600199	shneg	shCHD1	OK	11,0586	7,58826	-0,543328	-2,97978	5,00E-05	0,0004193	yes
SEC14L2	SEC14L2	SEC14L2	chr22:307929	shneg	shCHD1	OK	33,0935	22,7077	-0,543366	-2,63384	5,00E-05	0,0004193	yes
CYP51A1	CYP51A1	CYP51A1	chr7:9174146	shneg	shCHD1	OK	23,2974	15,9798	-0,543919	-2,8805	5,00E-05	0,0004193	yes
LOC650623	LOC650623	LOC650623	chr10:814427	shneg	shCHD1	OK	1,59808	1,09594	-0,544166	-2,2046	0,0002	0,00144867	yes
CCDC28A	CCDC28A	CCDC28A	chr6:1390463	shneg	shCHD1	OK	16,7918	11,5114	-0,544692	-2,58174	5,00E-05	0,0004193	yes
LRP10	LRP10	LRP10	chr14:233409	shneg	shCHD1	OK	99,4374	68,1672	-0,544711	-3,15227	5,00E-05	0,0004193	yes
NEK1	NEK1	NEK1	chr4:1703144	shneg	shCHD1	OK	2,88119	1,97476	-0,544986	-2,5134	5,00E-05	0,0004193	yes
TCEAL4	TCEAL4	TCEAL4	chrX:1028404	shneg	shCHD1	OK	93,9389	64,3831	-0,545041	-2,96892	5,00E-05	0,0004193	yes
ZNF426	ZNF426	ZNF426	chr19:963868	shneg	shCHD1	OK	2,4674	1,69089	-0,545208	-1,7968	0,0021	0,0108578	yes
NME4	NME4	NME4	chr16:447191	shneg	shCHD1	OK	183,118	125,447	-0,545691	-3,04767	5,00E-05	0,0004193	yes
PNPLA2	PNPLA2	PNPLA2	chr11:818900	shneg	shCHD1	OK	40,415	27,6828	-0,545904	-2,95132	5,00E-05	0,0004193	yes
ZGLP1	ZGLP1	ZGLP1	chr19:104154	shneg	shCHD1	OK	1,78845	1,22498	-0,545948	-1,46813	0,01165	0,0451385	yes
KCND1	KCND1	KCND1	chrX:4881863	shneg	shCHD1	OK	7,21221	4,93982	-0,545984	-2,70819	5,00E-05	0,0004193	yes
LGALS3	LGALS3	LGALS3	chr14:555959	shneg	shCHD1	OK	203,564	139,382	-0,546443	-3,0092	5,00E-05	0,0004193	yes
FAM102B	FAM102B	FAM102B	chr1:1091029	shneg	shCHD1	OK	3,01767	2,06566	-0,546832	-2,56552	5,00E-05	0,0004193	yes
CAMK2D	CAMK2D	CAMK2D	chr4:1143721	shneg	shCHD1	OK	27,3091	18,6884	-0,54724	-2,59529	5,00E-05	0,0004193	yes
FXYD6	FXYD6	FXYD6	chr11:117690	shneg	shCHD1	OK	85,0238	58,1775	-0,547407	-1,87832	0,00125	0,00702633	yes
GPR135	GPR135	GPR135	chr14:599302	shneg	shCHD1	OK	5,15295	3,52485	-0,547838	-2,14526	0,0004	0,00266202	yes
STOM	STOM	STOM	chr9:1241013	shneg	shCHD1	OK	6,09961	4,1717	-0,548082	-2,57402	5,00E-05	0,0004193	yes
B4GALNT3	B4GALNT3	B4GALNT3	chr12:569542	shneg	shCHD1	OK	54,0662	36,9741	-0,548211	-3,09718	5,00E-05	0,0004193	yes
PMFBP1	PMFBP1	PMFBP1	chr16:721529	shneg	shCHD1	OK	1,58812	1,08592	-0,548404	-1,78655	0,00235	0,0119595	yes
FAM126B	FAM126B	FAM126B	chr2:2018384	shneg	shCHD1	OK	5,03127	3,43972	-0,54863	-2,8126	5,00E-05	0,0004193	yes
GOLGA1	GOLGA1	GOLGA1	chr9:1276405	shneg	shCHD1	OK	6,17866	4,22233	-0,549253	-2,68007	5,00E-05	0,0004193	yes
CHRM3	CHRM3	CHRM3	chr1:2397923	shneg	shCHD1	OK	2,6723	1,82527	-0,549973	-2,00368	0,00065	0,0040329	yes
LOC399715	LOC399715	LOC399715	chr10:631964	shneg	shCHD1	OK	11,4243	7,80092	-0,550394	-2,7308	5,00E-05	0,0004193	yes
SEMA4F	SEMA4F	SEMA4F	chr2:7488139	shneg	shCHD1	OK	7,81316	5,33137	-0,551401	-2,72484	5,00E-05	0,0004193	yes
OSTF1	OSTF1	OSTF1	chr9:7770339	shneg	shCHD1	OK	9,65221	6,58213	-0,552305	-2,36524	0,0002	0,00144867	yes
OBSCN	OBSCN	OBSCN	chr1:2283958	shneg	shCHD1	OK	2,22928	1,51812	-0,554293	-2,83715	5,00E-05	0,0004193	yes
SLC27A3	SLC27A3	SLC27A3	chr1:1537477	shneg	shCHD1	OK	3,94327	2,68524	-0,554344	-2,20656	0,0002	0,00144867	yes
TSPYL2	TSPYL2	TSPYL2	chrX:5311154	shneg	shCHD1	OK	20,5743	14,0104	-0,55435	-2,89897	5,00E-05	0,0004193	yes
SPTB	SPTB	SPTB	chr14:652130	shneg	shCHD1	OK	23,8035	16,1989	-0,55528	-3,05967	5,00E-05	0,0004193	yes
UBE2L6	UBE2L6	UBE2L6	chr11:573191	shneg	shCHD1	OK	68,9381	46,8936	-0,55591	-2,97357	5,00E-05	0,0004193	yes
FLJ35390	FLJ35390	FLJ35390	chr7:4406848	shneg	shCHD1	OK	4,36953	2,96982	-0,557102	-2,14047	0,00025	0,00176854	yes
AQP5	AQP5	AQP5	chr12:503552	shneg	shCHD1	OK	4,82573	3,27956	-0,557247	-2,1332	0,00055	0,00350455	yes
ZNF581	ZNF581	ZNF581	chr19:561549	shneg	shCHD1	OK	31,3696	21,3113	-0,557749	-2,76054	5,00E-05	0,0004193	yes
BBS12	BBS12	BBS12	chr4:1236538	shneg	shCHD1	OK	2,70738	1,83917	-0,557845	-2,16032	5,00E-05	0,0004193	yes
ITFG1	ITFG1	ITFG1	chr16:471892	shneg	shCHD1	OK	16,6683	11,3162	-0,558717	-2,77686	5,00E-05	0,0004193	yes
AKAP7	AKAP7	AKAP7	chr6:1314568	shneg	shCHD1	OK	1,14907	0,780051	-0,558831	-1,43552	0,0098	0,0392479	yes
PPM1D	PPM1D	PPM1D	chr17:586775	shneg	shCHD1	OK	11,0373	7,49197	-0,558971	-2,90412	5,00E-05	0,0004193	yes
ADAMTS13	ADAMTS13	ADAMTS13	chr9:1362432	shneg	shCHD1	OK	6,28816	4,26819	-0,559015	-1,76046	0,00265	0,0132017	yes
SLC27A1	SLC27A1	SLC27A1	chr19:175812	shneg	shCHD1	OK	7,20354	4,88948	-0,559025	-2,69118	5,00E-05	0,0004193	yes
ARMCX1	ARMCX1	ARMCX1	chrX:1008055	shneg	shCHD1	OK	1,5674	1,06376	-0,559206	-1,46292	0,0132	0,0499627	yes
PLXNA3	PLXNA3	PLXNA3	chrX:1536866	shneg	shCHD1	OK	21,2629	14,4303	-0,559244	-3,07201	5,00E-05	0,0004193	yes
TMEM14A	TMEM14A	TMEM14A	chr6:5253588	shneg	shCHD1	OK	43,1433	29,2698	-0,559725	-2,79759	5,00E-05	0,0004193	yes
ZNF586	ZNF586	ZNF586	chr19:582810	shneg	shCHD1	OK	3,56218	2,41619	-0,560025	-2,03282	0,00035	0,00236888	yes
RASAL2	RASAL2	RASAL2	chr1:1780606	shneg	shCHD1	OK	33,667	22,8333	-0,560195	-3,21072	5,00E-05	0,0004193	yes
MAP7	MAP7	MAP7	chr6:1366634	shneg	shCHD1	OK	2,1613	1,46482	-0,561174	-2,17626	5,00E-05	0,0004193	yes
SEPSECS	SEPSECS	SEPSECS	chr4:2512162	shneg	shCHD1	OK	2,33464	1,5813	-0,562087	-2,53632	5,00E-05	0,0004193	yes
ZNF573	ZNF573	ZNF573	chr19:382292	shneg	shCHD1	OK	1,59203	1,07827	-0,562147	-1,49156	0,00655	0,0279776	yes
EEA1	EEA1	EEA1	chr12:931662	shneg	shCHD1	OK	3,36449	2,27834	-0,562404	-2,71919	5,00E-05	0,0004193	yes
AASDH	AASDH	AASDH	chr4:5720445	shneg	shCHD1	OK	5,3501	3,61865	-0,564117	-2,6646	5,00E-05	0,0004193	yes
FYN	FYN	FYN	chr6:1119815	shneg	shCHD1	OK	1,16359	0,786967	-0,564205	-1,53451	0,00645	0,0276097	yes
HLA-DMA	HLA-DMA	HLA-DMA	chr6_ssto_ha	shneg	shCHD1	OK	5,99327	4,04894	-0,565799	-1,89782	0,0012	0,00680293	yes
WDR72	WDR72	WDR72	chr15:538059	shneg	shCHD1	OK	1,28895	0,870164	-0,566834	-2,28834	5,00E-05	0,0004193	yes
PLBD1	PLBD1	PLBD1	chr12:146565	shneg	shCHD1	OK	79,7042	53,8024	-0,566985	-3,15633	5,00E-05	0,0004193	yes
CILP2	CILP2	CILP2	chr19:196490	shneg	shCHD1	OK	2,09409	1,41349	-0,567063	-2,22352	5,00E-05	0,0004193	yes
TMC4	TMC4	TMC4	chr19:546638	shneg	shCHD1	OK	1,54962	1,04592	-0,567139	-1,57823	0,00645	0,0276097	yes
PEX11A	PEX11A	PEX11A	chr15:902262	shneg	shCHD1	OK	5,58514	3,76707	-0,56815	-1,88017	0,0017	0,00909693	yes
SAMD9	SAMD9	SAMD9	chr7:9272882	shneg	shCHD1	OK	2,07533	1,39971	-0,568213	-2,6222	5,00E-05	0,0004193	yes
TMEM25	TMEM25	TMEM25	chr11:118401	shneg	shCHD1	OK	9,14287	6,15879	-0,57	-2,01507	0,0009	0,00533451	yes
RFTN1	RFTN1	RFTN1	chr3:1635735	shneg	shCHD1	OK	1,88742	1,27081	-0,570671	-1,87509	0,00145	0,00793286	yes
CTSA	CTSA	CTSA	chr20:445172	shneg	shCHD1	OK	74,2895	50,017	-0,570742	-2,1187	0,0003	0,0020738	yes
UCP3	UCP3	UCP3	chr11:737113	shneg	shCHD1	OK	2,98373	2,00883	-0,570763	-2,04171	0,0009	0,00533451	yes
ZHX2	ZHX2	ZHX2	chr8:1237939	shneg	shCHD1	OK	3,31068	2,22893	-0,570778	-2,64241	5,00E-05	0,0004193	yes
C15orf52	C15orf52	C15orf52	chr15:406236	shneg	shCHD1	OK	26,4394	17,7978	-0,570992	-3,18113	5,00E-05	0,0004193	yes
DUSP18	DUSP18	DUSP18	chr22:310580	shneg	shCHD1	OK	3,88068	2,61161	-0,571369	-2,27397	5,00E-05	0,0004193	yes
GDPD5	GDPD5	GDPD5	chr11:751456	shneg	shCHD1	OK	2,39082	1,60764	-0,57256	-2,10679	0,0004	0,00266202	yes
SLFN5	SLFN5	SLFN5	chr17:335700	shneg	shCHD1	OK	4,31853	2,90387	-0,572563	-2,74309	5,00E-05	0,0004193	yes
ACCN2	ACCN2	ACCN2	chr12:504514	shneg	shCHD1	OK	110,434	74,2549	-0,57262	-3,37908	5,00E-05	0,0004193	yes
OLFML2A	OLFML2A	OLFML2A	chr9:1275394	shneg	shCHD1	OK	6,32545	4,25307	-0,572664	-2,90802	5,00E-05	0,0004193	yes
TNFSF10	TNFSF10	TNFSF10	chr3:1722232	shneg	shCHD1	OK	6,10309	4,10199	-0,573216	-2,38321	5,00E-05	0,0004193	yes
TXNIP	TXNIP	TXNIP	chr1:1454384	shneg	shCHD1	OK	11,8666	7,97525	-0,573304	-2,82032	5,00E-05	0,0004193	yes
DHRS3	DHRS3	DHRS3	chr1:1262793	shneg	shCHD1	OK	88,592	59,5215	-0,573765	-3,20409	5,00E-05	0,0004193	yes
IRS2	IRS2	IRS2	chr13:110406	shneg	shCHD1	OK	8,78163	5,89733	-0,574426	-3,02128	5,00E-05	0,0004193	yes
GPR137B	GPR137B	GPR137B	chr1:2363058	shneg	shCHD1	OK	26,4258	17,7438	-0,574631	-2,97331	5,00E-05	0,0004193	yes

TMX4	TMX4	TMX4	chr20:796171	shneg	shCHD1	OK	6,22238	4,17626	-0,575255	-2,6391	5,00E-05	0,0004193	yes
SLC45A1	SLC45A1	SLC45A1	chr1:8384389	shneg	shCHD1	OK	6,9724	4,67828	-0,575678	-2,65596	5,00E-05	0,0004193	yes
MAN1A2	MAN1A2	MAN1A2	chr1:1179100	shneg	shCHD1	OK	11,7842	7,90547	-0,575932	-2,96973	5,00E-05	0,0004193	yes
PLK3	PLK3	PLK3	chr1:4526603	shneg	shCHD1	OK	7,58135	5,08571	-0,576005	-2,60951	5,00E-05	0,0004193	yes
SLC12A6	SLC12A6	SLC12A6	chr15:345172	shneg	shCHD1	OK	4,12179	2,76468	-0,576161	-1,76318	0,0019	0,00996052	yes
ANXA9	ANXA9	ANXA9	chr1:1509544	shneg	shCHD1	OK	2,35784	1,58149	-0,576187	-1,66731	0,00455	0,0207047	yes
ARMC2	ARMC2	ARMC2	chr6:1091696	shneg	shCHD1	OK	3,03872	2,03803	-0,576287	-2,38334	5,00E-05	0,0004193	yes
ISG20	ISG20	ISG20	chr15:891820	shneg	shCHD1	OK	16,2025	10,8637	-0,576704	-2,56516	5,00E-05	0,0004193	yes
ALOX12B	ALOX12B	ALOX12B	chr17:797595	shneg	shCHD1	OK	1,55307	1,04107	-0,577055	-1,61757	0,00665	0,0283439	yes
DSG2	DSG2	DSG2	chr18:290780	shneg	shCHD1	OK	0,792155	0,530852	-0,577473	-1,73778	0,0042	0,0193522	yes
KIAA1211	KIAA1211	KIAA1211	chr4:5703636	shneg	shCHD1	OK	5,81063	3,8935	-0,577628	-2,92761	5,00E-05	0,0004193	yes
KCTD2	KCTD2	KCTD2	chr17:730432	shneg	shCHD1	OK	11,3481	7,60282	-0,577848	-2,92925	5,00E-05	0,0004193	yes
KCNMB4	KCNMB4	KCNMB4	chr12:707600	shneg	shCHD1	OK	1,39937	0,93733	-0,578145	-2,04411	0,0005	0,00321686	yes
KLHL2	KLHL2	KLHL2	chr4:1661287	shneg	shCHD1	OK	5,5415	3,70992	-0,578888	-2,6231	5,00E-05	0,0004193	yes
TMTC2	TMTC2	TMTC2	chr12:830809	shneg	shCHD1	OK	2,68771	1,79847	-0,579608	-2,60022	5,00E-05	0,0004193	yes
PIGQ	PIGQ	PIGQ	chr16:619967	shneg	shCHD1	OK	13,7059	9,16772	-0,580161	-2,90547	5,00E-05	0,0004193	yes
PNPLA7	PNPLA7	PNPLA7	chr9:1403544	shneg	shCHD1	OK	4,95081	3,3072	-0,582052	-2,80345	5,00E-05	0,0004193	yes
PER3	PER3	PER3	chr1:7844762	shneg	shCHD1	OK	2,67932	1,7897	-0,582149	-2,70623	5,00E-05	0,0004193	yes
CARD10	CARD10	CARD10	chr22:378863	shneg	shCHD1	OK	13,45	8,98175	-0,582539	-3,01688	5,00E-05	0,0004193	yes
CRISPLD2	CRISPLD2	CRISPLD2	chr16:848535	shneg	shCHD1	OK	2,82992	1,88897	-0,583164	-2,60035	5,00E-05	0,0004193	yes
HELQ	HELQ	HELQ	chr4:8432849	shneg	shCHD1	OK	2,68408	1,79131	-0,583413	-2,35036	0,00025	0,00176854	yes
CCDC106	CCDC106	CCDC106	chr19:561589	shneg	shCHD1	OK	10,1699	6,78581	-0,583715	-2,7592	5,00E-05	0,0004193	yes
AVPI1	AVPI1	AVPI1	chr10:994371	shneg	shCHD1	OK	2,44227	1,62911	-0,584141	-1,49963	0,01065	0,0419524	yes
MXD1	MXD1	MXD1	chr2:7014217	shneg	shCHD1	OK	2,36402	1,57688	-0,584175	-2,61969	5,00E-05	0,0004193	yes
RPS6KA4	RPS6KA4	RPS6KA4	chr11:641266	shneg	shCHD1	OK	33,7626	22,5191	-0,584273	-3,16758	5,00E-05	0,0004193	yes
STXBP4	STXBP4	STXBP4	chr17:530461	shneg	shCHD1	OK	1,86528	1,24404	-0,584356	-2,49784	5,00E-05	0,0004193	yes
SERTAD1	SERTAD1	SERTAD1	chr19:409284	shneg	shCHD1	OK	12,6855	8,46022	-0,584418	-2,63059	5,00E-05	0,0004193	yes
SGSH	SGSH	SGSH	chr17:781830	shneg	shCHD1	OK	9,83622	6,55916	-0,584593	-2,82949	5,00E-05	0,0004193	yes
LMCD1	LMCD1	LMCD1	chr3:8543510	shneg	shCHD1	OK	11,6768	7,78416	-0,58503	-2,75568	5,00E-05	0,0004193	yes
PTPRH	PTPRH	PTPRH	chr19:556926	shneg	shCHD1	OK	9,17376	6,11455	-0,585265	-2,90691	5,00E-05	0,0004193	yes
HCFC2	HCFC2	HCFC2	chr12:104458	shneg	shCHD1	OK	2,32482	1,54859	-0,586161	-2,64128	5,00E-05	0,0004193	yes
CCDC76	CCDC76	CCDC76	chr1:1005987	shneg	shCHD1	OK	5,58811	3,72191	-0,586318	-2,42679	5,00E-05	0,0004193	yes
NYAP1	NYAP1	NYAP1	chr7:1000815	shneg	shCHD1	OK	11,4468	7,6229	-0,586531	-2,94567	5,00E-05	0,0004193	yes
LOC10013089	LOC10013089	LOC10013089	chr6:9996886	shneg	shCHD1	OK	4,42057	2,9434	-0,586748	-2,22061	0,00015	0,00112331	yes
GPR162	GPR162	GPR162	chr12:693096	shneg	shCHD1	OK	21,0659	14,0254	-0,586874	-2,77418	5,00E-05	0,0004193	yes
ACHE	ACHE	ACHE	chr7:1004876	shneg	shCHD1	OK	3,7379	2,48842	-0,586999	-2,23155	5,00E-05	0,0004193	yes
CD47	CD47	CD47	chr3:1077619	shneg	shCHD1	OK	13,2997	8,85329	-0,587111	-3,128	5,00E-05	0,0004193	yes
C2CD3	C2CD3	C2CD3	chr11:737454	shneg	shCHD1	OK	22,1863	14,7656	-0,587436	-3,2706	5,00E-05	0,0004193	yes
PPAP2B	PPAP2B	PPAP2B	chr1:5696041	shneg	shCHD1	OK	1,31747	0,876697	-0,587623	-1,74037	0,00385	0,0180379	yes
ARHGEF5	ARHGEF5	ARHGEF5	chr7:1440524	shneg	shCHD1	OK	1,00639	0,669682	-0,587641	-1,93986	0,00185	0,00975716	yes
ZNF761	ZNF761	ZNF761	chr19:539352	shneg	shCHD1	OK	4,44901	2,95974	-0,58801	-1,82601	0,00145	0,00793286	yes
RGS12	RGS12	RGS12	chr4:3315873	shneg	shCHD1	OK	5,2418	3,48712	-0,588026	-2,75483	5,00E-05	0,0004193	yes
ELFN2	ELFN2	ELFN2	chr22:377639	shneg	shCHD1	OK	28,5162	18,9578	-0,588987	-3,36418	5,00E-05	0,0004193	yes
ETHE1	ETHE1	ETHE1	chr19:440108	shneg	shCHD1	OK	46,6604	31,0168	-0,58915	-2,94789	5,00E-05	0,0004193	yes
CBLN3	CBLN3	CBLN3	chr14:248957	shneg	shCHD1	OK	3,69965	2,45903	-0,589301	-2,26437	0,0001	0,00078473	yes
ZNF616	ZNF616	ZNF616	chr19:526176	shneg	shCHD1	OK	3,04127	2,02055	-0,589925	-2,33788	5,00E-05	0,0004193	yes
TMEM63B	TMEM63B	TMEM63B	chr6:4409537	shneg	shCHD1	OK	23,8855	15,8636	-0,590415	-3,14962	5,00E-05	0,0004193	yes
DGKE	DGKE	DGKE	chr17:549114	shneg	shCHD1	OK	2,88411	1,91494	-0,590826	-2,82146	5,00E-05	0,0004193	yes
C17orf28	C17orf28	C17orf28	chr17:729468	shneg	shCHD1	OK	5,72741	3,80253	-0,590925	-2,78003	5,00E-05	0,0004193	yes
IDUA	IDUA	IDUA	chr4:972862-[illegible]	shneg	shCHD1	OK	7,54869	5,01152	-0,590979	-2,09584	0,0002	0,00144867	yes
SH3TC1	SH3TC1	SH3TC1	chr4:8201059	shneg	shCHD1	OK	3,8659	2,56627	-0,59113	-2,76314	5,00E-05	0,0004193	yes
ABCA7	ABCA7	ABCA7	chr19:104010	shneg	shCHD1	OK	10,4348	6,92319	-0,591893	-3,13237	5,00E-05	0,0004193	yes
KLHDC8B	KLHDC8B	KLHDC8B	chr3:4920901	shneg	shCHD1	OK	17,4305	11,5622	-0,592198	-2,9094	5,00E-05	0,0004193	yes
TMEM129	TMEM129	TMEM129	chr4:1717678	shneg	shCHD1	OK	15,8056	10,4827	-0,592428	-3,00768	5,00E-05	0,0004193	yes
EHD2	EHD2	EHD2	chr19:482166	shneg	shCHD1	OK	24,395	16,1678	-0,593458	-3,19546	5,00E-05	0,0004193	yes
FAT1	FAT1	FAT1	chr4:1875089	shneg	shCHD1	OK	153,698	101,823	-0,594032	-3,59404	5,00E-05	0,0004193	yes
SOX9	SOX9	SOX9	chr17:701171	shneg	shCHD1	OK	151,449	100,311	-0,594355	-3,51369	5,00E-05	0,0004193	yes
ZNF518A	ZNF518A	ZNF518A	chr10:978894	shneg	shCHD1	OK	3,79119	2,51056	-0,594641	-2,91733	5,00E-05	0,0004193	yes
FGF5	FGF5	FGF5	chr4:8118774	shneg	shCHD1	OK	5,73082	3,79477	-0,59473	-2,9276	5,00E-05	0,0004193	yes
S100A10	S100A10	S100A10	chr1:1519553	shneg	shCHD1	OK	741,452	490,918	-0,59487	-3,53332	5,00E-05	0,0004193	yes
HAGHL	HAGHL	HAGHL	chr16:777265	shneg	shCHD1	OK	13,4468	8,90115	-0,595197	-2,73318	5,00E-05	0,0004193	yes
SCARB2	SCARB2	SCARB2	chr4:7707989	shneg	shCHD1	OK	41,938	27,7384	-0,596374	-3,37768	5,00E-05	0,0004193	yes
PAPLN	PAPLN	PAPLN	chr14:737042	shneg	shCHD1	OK	4,16527	2,75302	-0,597398	-2,86179	5,00E-05	0,0004193	yes
ANAPC2	ANAPC2	ANAPC2	chr9:1400692	shneg	shCHD1	OK	18,8708	12,4632	-0,59848	-2,38099	5,00E-05	0,0004193	yes
TMEM102	TMEM102	TMEM102	chr17:733876	shneg	shCHD1	OK	3,16588	2,09089	-0,598488	-2,01511	0,0007	0,00428773	yes
KLC4	KLC4	KLC4	chr6:4302737	shneg	shCHD1	OK	14,6805	9,69428	-0,598695	-2,69037	5,00E-05	0,0004193	yes
FAM117A	FAM117A	FAM117A	chr17:477876	shneg	shCHD1	OK	3,57138	2,35829	-0,598742	-2,27225	5,00E-05	0,0004193	yes
SLC38A4	SLC38A4	SLC38A4	chr12:471585	shneg	shCHD1	OK	44,8369	29,6002	-0,599077	-3,36642	5,00E-05	0,0004193	yes
CES4A	CES4A	CES4A	chr16:670224	shneg	shCHD1	OK	4,53633	2,99477	-0,599078	-2,30737	0,0001	0,00078473	yes
BTN3A2	BTN3A2	BTN3A2	chr6:2636539	shneg	shCHD1	OK	6,39248	4,21991	-0,599163	-2,83911	5,00E-05	0,0004193	yes
EXOC1	EXOC1	EXOC1	chr4:5671981	shneg	shCHD1	OK	11,9958	7,91581	-0,599723	-3,05694	5,00E-05	0,0004193	yes
DDX60	DDX60	DDX60	chr4:1691374	shneg	shCHD1	OK	6,3125	4,16465	-0,600016	-3,01336	5,00E-05	0,0004193	yes
DCST2	DCST2	DCST2	chr1:1549910	shneg	shCHD1	OK	1,20055	0,791726	-0,600625	-1,47654	0,0125	0,04782	yes
TMLHE	TMLHE	TMLHE	chrX:1546962	shneg	shCHD1	OK	16,7725	11,0457	-0,60262	-2,23044	0,0001	0,00078473	yes
CKB	CKB	CKB	chr14:103985	shneg	shCHD1	OK	53,1126	34,9721	-0,602849	-3,19334	5,00E-05	0,0004193	yes
B3GNT3	B3GNT3	B3GNT3	chr19:179059	shneg	shCHD1	OK	1,67561	1,10311	-0,603114	-1,80551	0,00295	0,0144329	yes
IGFBP7	IGFBP7	IGFBP7	chr4:5784510	shneg	shCHD1	OK	889,105	585,18	-0,603473	-3,25903	5,00E-05	0,0004193	yes
GJA3	GJA3	GJA3	chr13:207123	shneg	shCHD1	OK	3,7792	2,48731	-0,603496	-2,87015	5,00E-05	0,0004193	yes
KRT80	KRT80	KRT80	chr12:525627	shneg	shCHD1	OK	64,6519	42,544	-0,603736	-3,46569	5,00E-05	0,0004193	yes
METTL14	METTL14	METTL14	chr4:1196065	shneg	shCHD1	OK	10,3513	6,81163	-0,603744	-2,89196	5,00E-05	0,0004193	yes
ZNF615	ZNF615	ZNF615	chr19:524945	shneg	shCHD1	OK	1,29127	0,849676	-0,603806	-1,92499	0,0013	0,00726051	yes
HOXA11	HOXA11	HOXA11	chr7:2722077	shneg	shCHD1	OK	4,41194	2,89883	-0,605944	-2,57598	5,00E-05	0,0004193	yes
ZNF461	ZNF461	ZNF461	chr19:371282	shneg	shCHD1	OK	1,74695	1,14773	-0,606065	-1,97724	0,00065	0,0040329	yes
NOG	NOG	NOG	chr17:546710	shneg	shCHD1	OK	1,99374	1,30941	-0,606555	-1,64921	0,00515	0,0229918	yes
DBP	DBP	DBP	chr19:491338	shneg	shCHD1	OK	8,23741	5,40641	-0,60752	-2,67173	5,00E-05	0,0004193	yes
FMNL1	FMNL1	FMNL1	chr17:432992	shneg	shCHD1	OK	7,38361	4,84422	-0,608062	-2,97261	5,00E-05	0,0004193	yes
VPS13A	VPS13A	VPS13A	chr9:7979167	shneg	shCHD1	OK	11,6656	7,65339	-0,608087	-3,2667	5,00E-05	0,0004193	yes
DPP7	DPP7	DPP7	chr9:1400049	shneg	shCHD1	OK	109,073	71,5342	-0,608589	-3,40588	5,00E-05	0,0004193	yes
EML1	EML1	EML1	chr14:100259	shneg	shCHD1	OK	29,8948	19,5959	-0,609341	-3,38192	5,00E-05	0,0004193	yes
ETFDH	ETFDH	ETFDH	chr4:1595932	shneg	shCHD1	OK	6,35733	4,16572	-0,609856	-2,79942	5,00E-05	0,0004193	yes
ADAM22	ADAM22	ADAM22	chr7:8756356	shneg	shCHD1	OK	8,92565	5,84841	-0,609912	-2,14644	0,0003	0,0020738	yes
NUDT14	NUDT14	NUDT14	chr14:105639	shneg	shCHD1	OK	64,0847	41,9689	-0,610659	-3,10901	5,00E-05	0,0004193	yes
SERPINA1	SERPINA1	SERPINA1	chr14:948430	shneg	shCHD1	OK	2,05225	1,34383	-0,610858	-1,99304	0,0004	0,00266202	yes

TNFRSF21	TNFRSF21	TNFRSF21	chr6:4719926	shneg	shCHD1	OK	1,21519	0,795412	-0,611407	-1,82291	0,0028	0,0138449	yes
SOCS1	SOCS1	SOCS1	chr16:113482	shneg	shCHD1	OK	24,2491	15,8714	-0,6115	-2,92173	5,00E-05	0,0004193	yes
RNASET2	RNASET2	RNASET2	chr6:1673430	shneg	shCHD1	OK	19,1451	12,5261	-0,612034	-2,94831	5,00E-05	0,0004193	yes
TMEM175	TMEM175	TMEM175	chr4:926261-9	shneg	shCHD1	OK	6,84531	4,47727	-0,612495	-2,60251	5,00E-05	0,0004193	yes
RIBC1	RIBC1	RIBC1	chrX:5344983	shneg	shCHD1	OK	4,78035	3,12641	-0,612608	-2,09584	0,0005	0,00321686	yes
USP53	USP53	USP53	chr4:1201337	shneg	shCHD1	OK	1,97719	1,2919	-0,613952	-2,74702	5,00E-05	0,0004193	yes
LGALS3BP	LGALS3BP	LGALS3BP	chr17:769673	shneg	shCHD1	OK	144,198	94,1613	-0,614842	-3,55006	5,00E-05	0,0004193	yes
UGGT2	UGGT2	UGGT2	chr13:964538	shneg	shCHD1	OK	5,38527	3,51533	-0,615359	-2,94065	5,00E-05	0,0004193	yes
C4orf33	C4orf33	C4orf33	chr4:1300148	shneg	shCHD1	OK	3,22569	2,10495	-0,615826	-1,8999	0,00105	0,00607369	yes
MGC23270	MGC23270	MGC23270	chr14:105287	shneg	shCHD1	OK	1,48156	0,965841	-0,617262	-1,69283	0,00485	0,0218981	yes
JAK3	JAK3	JAK3	chr19:179355	shneg	shCHD1	OK	3,24809	2,11517	-0,618819	-2,87257	5,00E-05	0,0004193	yes
FYCO1	FYCO1	FYCO1	chr3:4595939	shneg	shCHD1	OK	4,11463	2,67492	-0,621266	-3,08262	5,00E-05	0,0004193	yes
ZNF223	ZNF223	ZNF223	chr19:445561	shneg	shCHD1	OK	2,10735	1,36973	-0,621541	-1,91814	0,0014	0,00768951	yes
PRKAA2	PRKAA2	PRKAA2	chr1:5711098	shneg	shCHD1	OK	4,85905	3,15741	-0,621934	-3,17509	5,00E-05	0,0004193	yes
C9orf102	C9orf102	C9orf102	chr9:9856836	shneg	shCHD1	OK	3,07622	1,99795	-0,622641	-1,70093	0,0073	0,030664	yes
MTMR4	MTMR4	MTMR4	chr17:565668	shneg	shCHD1	OK	6,73496	4,37226	-0,623291	-3,13654	5,00E-05	0,0004193	yes
PSAP	PSAP	PSAP	chr10:735760	shneg	shCHD1	OK	881,508	571,754	-0,62458	-3,76771	5,00E-05	0,0004193	yes
TMEM53	TMEM53	TMEM53	chr1:4511950	shneg	shCHD1	OK	4,74353	3,07557	-0,625108	-2,23039	0,0001	0,00078473	yes
ATP6AP1	ATP6AP1	ATP6AP1	chrX:1536569	shneg	shCHD1	OK	104,811	67,9422	-0,625407	-3,5488	5,00E-05	0,0004193	yes
C1orf85	C1orf85	C1orf85	chr1:1562624	shneg	shCHD1	OK	22,9321	14,8644	-0,625502	-2,9224	5,00E-05	0,0004193	yes
PIGS	PIGS	PIGS	chr17:268804	shneg	shCHD1	OK	17,1638	11,1254	-0,625519	-3,21122	5,00E-05	0,0004193	yes
MMRN2	MMRN2	MMRN2	chr10:886952	shneg	shCHD1	OK	3,71736	2,4091	-0,625787	-2,89962	5,00E-05	0,0004193	yes
BHLHB9	BHLHB9	BHLHB9	chrX:1019756	shneg	shCHD1	OK	3,2654	2,11538	-0,626342	-2,75948	5,00E-05	0,0004193	yes
INPP5D	INPP5D	INPP5D	chr2:2339250	shneg	shCHD1	OK	3,59588	2,32776	-0,627406	-2,90859	5,00E-05	0,0004193	yes
CRIP2	CRIP2	CRIP2	chr14:105941	shneg	shCHD1	OK	101,813	65,8882	-0,627829	-3,42513	5,00E-05	0,0004193	yes
ZNF600	ZNF600	ZNF600	chr19:532687	shneg	shCHD1	OK	3,53373	2,28653	-0,62803	-2,43611	5,00E-05	0,0004193	yes
MEGF9	MEGF9	MEGF9	chr9:1233631	shneg	shCHD1	OK	2,23971	1,44879	-0,628463	-2,86911	5,00E-05	0,0004193	yes
PLEKHF1	PLEKHF1	PLEKHF1	chr19:301563	shneg	shCHD1	OK	19,4947	12,6097	-0,628553	-3,0658	5,00E-05	0,0004193	yes
BAHD1	BAHD1	BAHD1	chr15:407334	shneg	shCHD1	OK	20,7269	13,3866	-0,630714	-3,41213	5,00E-05	0,0004193	yes
SLC40A1	SLC40A1	SLC40A1	chr2:1904253	shneg	shCHD1	OK	1,06242	0,686092	-0,630875	-1,70888	0,00415	0,0191853	yes
NIPSNAP3A	NIPSNAP3A	NIPSNAP3A	chr9:1075099	shneg	shCHD1	OK	10,6113	6,85061	-0,631303	-2,88396	5,00E-05	0,0004193	yes
RPL10	RPL10	RPL10	chrX:1536265	shneg	shCHD1	OK	735,113	474,555	-0,63139	-3,67996	5,00E-05	0,0004193	yes
SLC9A3R2	SLC9A3R2	SLC9A3R2	chr16:207686	shneg	shCHD1	OK	14,9501	9,6501	-0,631535	-3,03098	5,00E-05	0,0004193	yes
PTCH1	PTCH1	PTCH1	chr9:9820526	shneg	shCHD1	OK	9,36629	6,04478	-0,631788	-3,20031	5,00E-05	0,0004193	yes
LAMB2	LAMB2	LAMB2	chr3:4915854	shneg	shCHD1	OK	34,9442	22,5385	-0,632662	-3,58603	5,00E-05	0,0004193	yes
DST	DST	DST	chr6:5632278	shneg	shCHD1	OK	70,6394	45,5516	-0,632974	-3,76651	5,00E-05	0,0004193	yes
ZNF846	ZNF846	ZNF846	chr19:986815	shneg	shCHD1	OK	4,35024	2,80211	-0,63458	-2,47274	5,00E-05	0,0004193	yes
CA11	CA11	CA11	chr19:491412	shneg	shCHD1	OK	129,409	83,2925	-0,635675	-3,5337	5,00E-05	0,0004193	yes
BEGAIN	BEGAIN	BEGAIN	chr14:101003	shneg	shCHD1	OK	15,3912	9,90196	-0,636319	-3,19134	5,00E-05	0,0004193	yes
RGL1	RGL1	RGL1	chr1:1836052	shneg	shCHD1	OK	1,16719	0,750598	-0,636926	-2,12837	0,00025	0,00176854	yes
WFS1	WFS1	WFS1	chr4:6271576	shneg	shCHD1	OK	5,03977	3,24035	-0,63721	-2,95704	5,00E-05	0,0004193	yes
C3orf72	C3orf72	C3orf72	chr3:1386660	shneg	shCHD1	OK	4,68018	3,00788	-0,637816	-2,99531	5,00E-05	0,0004193	yes
LINS	LINS	LINS	chr15:101109	shneg	shCHD1	OK	3,07216	1,97436	-0,637867	-2,33467	5,00E-05	0,0004193	yes
BCORL1	BCORL1	BCORL1	chrX:1291391	shneg	shCHD1	OK	6,85155	4,4004	-0,638796	-3,28664	5,00E-05	0,0004193	yes
TAAR9	TAAR9	TAAR9	chr6:1328594	shneg	shCHD1	OK	4,32788	2,77891	-0,63914	-1,7903	0,00285	0,0140225	yes
UHMK1	UHMK1	UHMK1	chr1:1624669	shneg	shCHD1	OK	20,4927	13,1564	-0,639341	-3,56438	5,00E-05	0,0004193	yes
ZBTB42	ZBTB42	ZBTB42	chr14:105266	shneg	shCHD1	OK	7,03683	4,51337	-0,640719	-3,13569	5,00E-05	0,0004193	yes
MAPK11	MAPK11	MAPK11	chr22:507021	shneg	shCHD1	OK	47,5359	30,4408	-0,643014	-3,50527	5,00E-05	0,0004193	yes
CHN1	CHN1	CHN1	chr2:1756640	shneg	shCHD1	OK	3,09508	1,98157	-0,643336	-2,23943	0,00015	0,00112331	yes
CLDN7	CLDN7	CLDN7	chr17:715537	shneg	shCHD1	OK	20,5645	13,1514	-0,644934	-2,06243	0,0006	0,00377139	yes
DCAF6	DCAF6	DCAF6	chr1:1678859	shneg	shCHD1	OK	29,271	18,7174	-0,645087	-1,98706	0,0008	0,0048114	yes
BCL9	BCL9	BCL9	chr1:1470131	shneg	shCHD1	OK	7,91463	5,0608	-0,645156	-3,32548	5,00E-05	0,0004193	yes
NHLRC4	NHLRC4	NHLRC4	chr16:617031	shneg	shCHD1	OK	1,94159	1,24125	-0,645447	-1,7991	0,00245	0,0123735	yes
C5orf42	C5orf42	C5orf42	chr5:3710632	shneg	shCHD1	OK	7,35459	4,70115	-0,645632	-3,41891	5,00E-05	0,0004193	yes
BRMS1	BRMS1	BRMS1	chr11:661048	shneg	shCHD1	OK	53,2703	34,0429	-0,645976	-3,42709	5,00E-05	0,0004193	yes
EML5	EML5	EML5	chr14:890811	shneg	shCHD1	OK	0,74663	0,477119	-0,646044	-2,08734	0,00055	0,00350455	yes
TCEA2	TCEA2	TCEA2	chr20:626884	shneg	shCHD1	OK	7,44797	4,7594	-0,646065	-2,74435	5,00E-05	0,0004193	yes
POLD4	POLD4	POLD4	chr11:670853	shneg	shCHD1	OK	69,6601	44,4675	-0,646464	-2,48859	5,00E-05	0,0004193	yes
HDAC10	HDAC10	HDAC10	chr22:506836	shneg	shCHD1	OK	20,7686	13,2669	-0,646572	-3,34903	5,00E-05	0,0004193	yes
ID2	ID2	ID2	chr2:8822112	shneg	shCHD1	OK	87,1528	55,6681	-0,646696	-3,52701	5,00E-05	0,0004193	yes
HSD17B14	HSD17B14	HSD17B14	chr19:493162	shneg	shCHD1	OK	27,6372	17,6378	-0,647939	-3,14041	5,00E-05	0,0004193	yes
SLC12A5	SLC12A5	SLC12A5	chr20:446503	shneg	shCHD1	OK	1,22372	0,78056	-0,648695	-2,35542	0,0002	0,00144867	yes
SFN	SFN	SFN	chr1:2718963	shneg	shCHD1	OK	195,682	124,771	-0,649224	-3,6839	5,00E-05	0,0004193	yes
KIAA1109	KIAA1109	KIAA1109	chr4:1230917	shneg	shCHD1	OK	7,64976	4,87618	-0,649662	-3,56541	5,00E-05	0,0004193	yes
LOC10049946	LOC10049946	LOC10049946	chr17:703994	shneg	shCHD1	OK	22,0605	14,06	-0,649872	-3,32187	5,00E-05	0,0004193	yes
RBMS3	RBMS3	RBMS3	chr3:2932280	shneg	shCHD1	OK	1,9121	1,21818	-0,650426	-1,89012	0,00125	0,00702633	yes
MYO7A	MYO7A	MYO7A	chr11:768393	shneg	shCHD1	OK	1,34472	0,855928	-0,651739	-2,63555	5,00E-05	0,0004193	yes
ZNF80	ZNF80	ZNF80	chr3:1139534	shneg	shCHD1	OK	1,94234	1,23615	-0,651949	-2,13269	0,0002	0,00144867	yes
DCXR	DCXR	DCXR	chr17:799937	shneg	shCHD1	OK	43,8056	27,8657	-0,652629	-3,19226	5,00E-05	0,0004193	yes
MRC2	MRC2	MRC2	chr17:607047	shneg	shCHD1	OK	29,6336	18,8491	-0,652737	-3,66789	5,00E-05	0,0004193	yes
FUT8	FUT8	FUT8	chr14:658773	shneg	shCHD1	OK	9,46151	6,01731	-0,652953	-3,22459	5,00E-05	0,0004193	yes
C9orf64	C9orf64	C9orf64	chr9:8655322	shneg	shCHD1	OK	3,64678	2,31772	-0,653913	-2,52376	5,00E-05	0,0004193	yes
NBEAL1	NBEAL1	NBEAL1	chr2:2038796	shneg	shCHD1	OK	3,81395	2,42205	-0,655056	-3,11204	5,00E-05	0,0004193	yes
RGS3	RGS3	RGS3	chr9:1162070	shneg	shCHD1	OK	10,1618	6,45	-0,655777	-2,74482	5,00E-05	0,0004193	yes
GUCA1C	GUCA1C	GUCA1C	chr3:1086266	shneg	shCHD1	OK	13,4965	8,56437	-0,656169	-2,68635	5,00E-05	0,0004193	yes
MST1R	MST1R	MST1R	chr3:4992443	shneg	shCHD1	OK	2,51555	1,59515	-0,657183	-2,81917	5,00E-05	0,0004193	yes
MAPT	MAPT	MAPT	chr17_ctg5_h	shneg	shCHD1	OK	5,53462	3,50825	-0,657733	-3,1753	5,00E-05	0,0004193	yes
OAS1	OAS1	OAS1	chr12:113344	shneg	shCHD1	OK	76,6079	48,5128	-0,659127	-3,58846	5,00E-05	0,0004193	yes
CPD	CPD	CPD	chr17:287059	shneg	shCHD1	OK	15,7192	9,95415	-0,65916	-3,61795	5,00E-05	0,0004193	yes
NAB2	NAB2	NAB2	chr12:574826	shneg	shCHD1	OK	27,0752	17,1395	-0,659644	-1,9204	0,0016	0,00862477	yes
ZBTB37	ZBTB37	ZBTB37	chr1:1738374	shneg	shCHD1	OK	2,93744	1,85912	-0,65994	-1,90261	0,0018	0,00951868	yes
LOC728084	LOC728084	LOC728084	chr12:894049	shneg	shCHD1	OK	1,41416	0,894995	-0,659994	-1,73681	0,0037	0,0174466	yes
GIPR	GIPR	GIPR	chr19:461715	shneg	shCHD1	OK	2,85621	1,80763	-0,660003	-2,13263	0,00055	0,00350455	yes
C11orf35	C11orf35	C11orf35	chr11:537521	shneg	shCHD1	OK	10,2311	6,47246	-0,66058	-2,28885	0,0002	0,00144867	yes
HFE	HFE	HFE	chr6:2608750	shneg	shCHD1	OK	11,4521	7,2449	-0,66058	-3,11368	5,00E-05	0,0004193	yes
GBA	GBA	GBA	chr1:1552042	shneg	shCHD1	OK	17,4844	11,0558	-0,661274	-3,31481	5,00E-05	0,0004193	yes
FLJ42627	FLJ42627	FLJ42627	chr16:268898	shneg	shCHD1	OK	5,75929	3,64057	-0,661727	-3,34491	5,00E-05	0,0004193	yes
DNER	DNER	DNER	chr2:2302223	shneg	shCHD1	OK	4,34531	2,74674	-0,661735	-2,99455	5,00E-05	0,0004193	yes
C9orf172	C9orf172	C9orf172	chr9:1397388	shneg	shCHD1	OK	11,5809	7,31556	-0,662703	-3,23923	5,00E-05	0,0004193	yes
CGN	CGN	CGN	chr1:1514838	shneg	shCHD1	OK	3,64751	2,304	-0,662767	-3,09546	5,00E-05	0,0004193	yes
HIST1H2AG	HIST1H2AG	HIST1H2AG	chr6:2710081	shneg	shCHD1	OK	10,5619	6,67071	-0,662961	-1,72799	0,0037	0,0174466	yes
MID2	MID2	MID2	chrX:1070690	shneg	shCHD1	OK	6,95161	4,38947	-0,6633	-3,38899	5,00E-05	0,0004193	yes
TBCK	TBCK	TBCK	chr4:1069672	shneg	shCHD1	OK	4,91953	3,10375	-0,664507	-1,63072	0,00485	0,0218981	yes

ENPP1	ENPP1	ENPP1	chr6:1321291 shneg	shCHD1	OK	1,83769	1,15916	-0,664818	-3,00523	5,00E-05	0,0004193	yes
SIK1	SIK1	SIK1	chr21:448343 shneg	shCHD1	OK	18,2056	11,4802	-0,665232	-3,5745	5,00E-05	0,0004193	yes
NFATC4	NFATC4	NFATC4	chr14:248361 shneg	shCHD1	OK	17,3188	10,9029	-0,667628	-3,58137	5,00E-05	0,0004193	yes
TSPAN13	TSPAN13	TSPAN13	chr7:1679335 shneg	shCHD1	OK	2,88463	1,81463	-0,668711	-2,08496	0,0005	0,00321686	yes
CHMP6	CHMP6	CHMP6	chr17:789656 shneg	shCHD1	OK	8,80365	5,53762	-0,668837	-2,99231	5,00E-05	0,0004193	yes
SSX2IP	SSX2IP	SSX2IP	chr1:8510938 shneg	shCHD1	OK	15,9256	10,0061	-0,670467	-3,61384	5,00E-05	0,0004193	yes
ACSF2	ACSF2	ACSF2	chr17:485035 shneg	shCHD1	OK	21,6788	13,6013	-0,672544	-3,35886	5,00E-05	0,0004193	yes
PAX2	PAX2	PAX2	chr10:102505 shneg	shCHD1	OK	26,2819	16,4759	-0,673711	-3,65503	5,00E-05	0,0004193	yes
AP1AR	AP1AR	AP1AR	chr4:1131528 shneg	shCHD1	OK	13,7362	8,61092	-0,673746	-3,37119	5,00E-05	0,0004193	yes
TTPA	TTPA	TTPA	chr8:6397204 shneg	shCHD1	OK	0,920113	0,575947	-0,675874	-1,52051	0,01055	0,0416174	yes
LOC10050607	LOC10050607	LOC10050607	chr14:313437 shneg	shCHD1	OK	9,07797	5,67972	-0,67655	-1,89082	0,002	0,0104181	yes
LHX4	LHX4	LHX4	chr1:1801994 shneg	shCHD1	OK	2,49578	1,55958	-0,678338	-1,56034	0,00705	0,0297484	yes
LOC158376	LOC158376	LOC158376	chr9:3590947 shneg	shCHD1	OK	9,14396	5,71091	-0,679099	-2,96684	5,00E-05	0,0004193	yes
CC2D2A	CC2D2A	CC2D2A	chr4:1547148 shneg	shCHD1	OK	3,60282	2,24807	-0,680438	-2,16799	0,00015	0,00112331	yes
CYP27B1	CYP27B1	CYP27B1	chr12:581561 shneg	shCHD1	OK	14,1442	8,82494	-0,680552	-3,34851	5,00E-05	0,0004193	yes
AGFG2	AGFG2	AGFG2	chr7:1001368 shneg	shCHD1	OK	32,6843	20,3769	-0,681665	-3,79951	5,00E-05	0,0004193	yes
ZC3H12A	ZC3H12A	ZC3H12A	chr1:3794011 shneg	shCHD1	OK	7,05395	4,39671	-0,682008	-3,20648	5,00E-05	0,0004193	yes
ADAM9	ADAM9	ADAM9	chr8:3885450 shneg	shCHD1	OK	108,494	67,6039	-0,682431	-4,0055	5,00E-05	0,0004193	yes
KLF4	KLF4	KLF4	chr9:1102471 shneg	shCHD1	OK	0,841124	0,5232	-0,684957	-1,56665	0,0097	0,0389594	yes
PUS7L	PUS7L	PUS7L	chr12:441224 shneg	shCHD1	OK	3,16094	1,96482	-0,685962	-2,95834	5,00E-05	0,0004193	yes
GCLC	GCLC	GCLC	chr6:5336213 shneg	shCHD1	OK	26,3039	16,3356	-0,687259	-3,73696	5,00E-05	0,0004193	yes
C11orf1	C11orf1	C11orf1	chr11:111744 shneg	shCHD1	OK	4,90105	3,04334	-0,687435	-1,77191	0,00245	0,0123735	yes
DSE	DSE	DSE	chr6:1166012 shneg	shCHD1	OK	10,6139	6,58953	-0,687702	-3,47216	5,00E-05	0,0004193	yes
SDC1	SDC1	SDC1	chr2:2040055 shneg	shCHD1	OK	301,342	187,036	-0,688087	-4,13272	5,00E-05	0,0004193	yes
SOBP	SOBP	SOBP	chr6:1078113 shneg	shCHD1	OK	0,834843	0,517761	-0,689217	-2,18209	0,00035	0,00236888	yes
LRRC45	LRRC45	LRRC45	chr17:799812 shneg	shCHD1	OK	14,3331	8,88779	-0,689449	-3,3971	5,00E-05	0,0004193	yes
KANK2	KANK2	KANK2	chr19:112749 shneg	shCHD1	OK	79,8471	49,4731	-0,690595	-4,05438	5,00E-05	0,0004193	yes
SCD	SCD	SCD	chr10:102106 shneg	shCHD1	OK	184,654	114,369	-0,691127	-4,13849	5,00E-05	0,0004193	yes
FP588	FP588	FP588	chr9:3586027 shneg	shCHD1	OK	0,743226	0,460318	-0,691171	-1,93825	0,00165	0,00886	yes
FBXL5	FBXL5	FBXL5	chr4:1560600 shneg	shCHD1	OK	11,0866	6,86328	-0,691849	-3,46522	5,00E-05	0,0004193	yes
C9orf167	C9orf167	C9orf167	chr9:1401722 shneg	shCHD1	OK	23,6845	14,6544	-0,692609	-3,73549	5,00E-05	0,0004193	yes
RAB9B	RAB9B	RAB9B	chrX:1030772 shneg	shCHD1	OK	0,859475	0,53158	-0,693169	-1,79137	0,00255	0,0127904	yes
SGCB	SGCB	SGCB	chr4:5288686 shneg	shCHD1	OK	17,8937	11,0618	-0,693863	-3,69718	5,00E-05	0,0004193	yes
ANKRD50	ANKRD50	ANKRD50	chr4:1255852 shneg	shCHD1	OK	4,69307	2,90043	-0,694265	-3,42758	5,00E-05	0,0004193	yes
SLC28A1	SLC28A1	SLC28A1	chr15:854279 shneg	shCHD1	OK	16,6453	10,2837	-0,694749	-3,04501	5,00E-05	0,0004193	yes
MAGI3	MAGI3	MAGI3	chr1:1139334 shneg	shCHD1	OK	1,28166	0,791392	-0,695552	-2,65138	5,00E-05	0,0004193	yes
RCAN1	RCAN1	RCAN1	chr21:358887 shneg	shCHD1	OK	1,58893	0,980943	-0,695812	-1,83271	0,00145	0,00793286	yes
DBT	DBT	DBT	chr1:1006524 shneg	shCHD1	OK	3,4882	2,15291	-0,696194	-3,47884	5,00E-05	0,0004193	yes
TP53INP1	TP53INP1	TP53INP1	chr8:9593819 shneg	shCHD1	OK	1,86693	1,15113	-0,697617	-2,86557	5,00E-05	0,0004193	yes
CHMP4C	CHMP4C	CHMP4C	chr8:8264468 shneg	shCHD1	OK	4,31297	2,65742	-0,698656	-2,5287	5,00E-05	0,0004193	yes
GPR132	GPR132	GPR132	chr14:105515 shneg	shCHD1	OK	1,93801	1,19293	-0,700066	-2,46395	5,00E-05	0,0004193	yes
GLCCI1	GLCCI1	GLCCI1	chr7:8008373 shneg	shCHD1	OK	1,18927	0,731746	-0,700658	-2,29462	5,00E-05	0,0004193	yes
ANKRD62P1-F	ANKRD62P1-F	ANKRD62P1-F	chr22:171345 shneg	shCHD1	OK	2,16781	1,33292	-0,701651	-1,76414	0,00445	0,020336	yes
NEO1	NEO1	NEO1	chr15:733448 shneg	shCHD1	OK	4,68193	2,87813	-0,701974	-3,45253	5,00E-05	0,0004193	yes
PRICKLE4	PRICKLE4	PRICKLE4	chr6:4174849 shneg	shCHD1	OK	14,4213	8,85986	-0,702843	-3,37676	5,00E-05	0,0004193	yes
SPINK5	SPINK5	SPINK5	chr5:1474435 shneg	shCHD1	OK	5,01289	3,07893	-0,703215	-3,18552	5,00E-05	0,0004193	yes
KLHL35	KLHL35	KLHL35	chr11:751334 shneg	shCHD1	OK	3,6394	2,23498	-0,703439	-2,42216	0,0001	0,00078473	yes
ANKAR	ANKAR	ANKAR	chr2:1905407 shneg	shCHD1	OK	0,796797	0,488378	-0,706215	-1,87483	0,0019	0,00996052	yes
EGFL7	EGFL7	EGFL7	chr9:1395533 shneg	shCHD1	OK	121,369	74,1653	-0,710584	-3,91615	5,00E-05	0,0004193	yes
LOC10028731	LOC10028731	LOC10028731	chr12:309486 shneg	shCHD1	OK	2,64408	1,61538	-0,710893	-2,13654	0,00035	0,00236888	yes
TMEM19	TMEM19	TMEM19	chr12:720798 shneg	shCHD1	OK	7,30486	4,45413	-0,713714	-3,50857	5,00E-05	0,0004193	yes
TUBA8	TUBA8	TUBA8	chr22:185934 shneg	shCHD1	OK	1,02346	0,623656	-0,714638	-1,39866	0,00945	0,0380871	yes
MICALCL	MICALCL	MICALCL	chr11:123084 shneg	shCHD1	OK	3,6873	2,24688	-0,714646	-2,95878	5,00E-05	0,0004193	yes
MYO15B	MYO15B	MYO15B	chr17:735841 shneg	shCHD1	OK	2,17343	1,3235	-0,715611	-2,18733	0,0003	0,0020738	yes
HEXIM1	HEXIM1	HEXIM1	chr17:432246 shneg	shCHD1	OK	29,7768	18,1098	-0,717419	-3,98406	5,00E-05	0,0004193	yes
DNAJB14	DNAJB14	DNAJB14	chr4:1008207 shneg	shCHD1	OK	11,4342	6,95404	-0,717434	-3,43768	5,00E-05	0,0004193	yes
UGT2B7	UGT2B7	UGT2B7	chr4:6996219 shneg	shCHD1	OK	3,2368	1,96764	-0,718101	-2,34095	0,00015	0,00112331	yes
TMEM98	TMEM98	TMEM98	chr17:312549 shneg	shCHD1	OK	1,83199	1,11286	-0,719146	-1,71569	0,003	0,0146313	yes
TTYH2	TTYH2	TTYH2	chr17:722096 shneg	shCHD1	OK	0,986385	0,599082	-0,719398	-1,86041	0,00155	0,00838119	yes
LRRC34	LRRC34	LRRC34	chr3:1695112 shneg	shCHD1	OK	5,08001	3,08407	-0,719998	-2,76238	5,00E-05	0,0004193	yes
LOC10028947	LOC10028947	LOC10028947	chr20:175401 shneg	shCHD1	OK	3,97184	2,41083	-0,720281	-1,5998	0,00785	0,0326299	yes
LAG3	LAG3	LAG3	chr12:688166 shneg	shCHD1	OK	2,18652	1,32653	-0,720978	-2,05118	0,001	0,0058183	yes
RNF208	RNF208	RNF208	chr9:1401146 shneg	shCHD1	OK	12,4843	7,56228	-0,72322	-3,0366	5,00E-05	0,0004193	yes
NGEF	NGEF	NGEF	chr2:2337433 shneg	shCHD1	OK	36,5992	22,1563	-0,724096	-3,92276	5,00E-05	0,0004193	yes
DHRS7	DHRS7	DHRS7	chr14:606114 shneg	shCHD1	OK	45,3029	27,4059	-0,725119	-3,76381	5,00E-05	0,0004193	yes
PLEKHG5	PLEKHG5	PLEKHG5	chr1:6521213 shneg	shCHD1	OK	90,5057	54,724	-0,725834	-3,79786	5,00E-05	0,0004193	yes
HSD17B11	HSD17B11	HSD17B11	chr4:8825769 shneg	shCHD1	OK	36,1738	21,8643	-0,726368	-3,79689	5,00E-05	0,0004193	yes
PCDH10	PCDH10	PCDH10	chr4:1340704 shneg	shCHD1	OK	2,65715	1,60595	-0,726455	-3,27805	5,00E-05	0,0004193	yes
FGF2	FGF2	FGF2	chr4:1237478 shneg	shCHD1	OK	12,1982	7,36689	-0,727544	-3,59271	5,00E-05	0,0004193	yes
FUCA1	FUCA1	FUCA1	chr1:2417157 shneg	shCHD1	OK	20,7573	12,5127	-0,730225	-3,67356	5,00E-05	0,0004193	yes
RCN3	RCN3	RCN3	chr19:500308 shneg	shCHD1	OK	5,89167	3,54888	-0,731312	-2,93763	5,00E-05	0,0004193	yes
ST6GALNAC2	ST6GALNAC2	ST6GALNAC2	chr17:745614 shneg	shCHD1	OK	3,89257	2,34454	-0,731417	-2,67951	5,00E-05	0,0004193	yes
ZMAT1	ZMAT1	ZMAT1	chrX:1011372 shneg	shCHD1	OK	9,47683	5,70477	-0,732236	-3,383	5,00E-05	0,0004193	yes
FAM55D	FAM55D	FAM55D	chr11:114441 shneg	shCHD1	OK	3,34463	2,01276	-0,732669	-2,34578	5,00E-05	0,0004193	yes
NET1	NET1	NET1	chr10:545451 shneg	shCHD1	OK	264,825	159,347	-0,732867	-4,36624	5,00E-05	0,0004193	yes
ARRDC4	ARRDC4	ARRDC4	chr15:985039 shneg	shCHD1	OK	1,35397	0,814286	-0,733592	-2,35096	5,00E-05	0,0004193	yes
NPNT	NPNT	NPNT	chr4:1068165 shneg	shCHD1	OK	114,309	68,7373	-0,733769	-4,30713	5,00E-05	0,0004193	yes
ACSM3	ACSM3	ACSM3	chr16:207753 shneg	shCHD1	OK	9,44106	5,67068	-0,735427	-2,06464	0,0007	0,00428773	yes
LPAL2	LPAL2	LPAL2	chr6:1608875 shneg	shCHD1	OK	1,21999	0,731949	-0,737051	-1,59047	0,00635	0,0272991	yes
C5orf39	C5orf39	C5orf39	chr5:4303918 shneg	shCHD1	OK	4,21246	2,52426	-0,738805	-2,22265	0,00045	0,00293585	yes
SPHK1	SPHK1	SPHK1	chr17:743806 shneg	shCHD1	OK	6,06318	3,62801	-0,740895	-3,00572	5,00E-05	0,0004193	yes
EP400	EP400	EP400	chr12:132434 shneg	shCHD1	OK	32,4671	19,4207	-0,741378	-4,32419	5,00E-05	0,0004193	yes
CLDN4	CLDN4	CLDN4	chr7:7324519 shneg	shCHD1	OK	182,323	109,016	-0,741948	-4,27672	5,00E-05	0,0004193	yes
FGF13	FGF13	FGF13	chrX:1377137 shneg	shCHD1	OK	19,7923	11,8047	-0,745578	-3,77044	5,00E-05	0,0004193	yes
ABCA9	ABCA9	ABCA9	chr17:669707 shneg	shCHD1	OK	1,00497	0,598959	-0,746628	-2,53658	5,00E-05	0,0004193	yes
MAFG	MAFG	MAFG	chr17:798761 shneg	shCHD1	OK	16,1809	9,64319	-0,74671	-3,99153	5,00E-05	0,0004193	yes
ATAD3C	ATAD3C	ATAD3C	chr1:1385068 shneg	shCHD1	OK	10,5007	6,25749	-0,746827	-3,72399	5,00E-05	0,0004193	yes
SMPDL3A	SMPDL3A	SMPDL3A	chr6:1231099 shneg	shCHD1	OK	1,54232	0,917979	-0,748564	-1,91073	0,00205	0,0106507	yes
FAM171B	FAM171B	FAM171B	chr2:1875587 shneg	shCHD1	OK	5,90902	3,51411	-0,749758	-3,42146	5,00E-05	0,0004193	yes
PCDH1	PCDH1	PCDH1	chr5:1412326 shneg	shCHD1	OK	84,245	50,0531	-0,751131	-4,31496	5,00E-05	0,0004193	yes
CRIP1	CRIP1	CRIP1	chr14:105953 shneg	shCHD1	OK	534,48	317,518	-0,751294	-4,18645	5,00E-05	0,0004193	yes
HPGD	HPGD	HPGD	chr4:1754113 shneg	shCHD1	OK	225,068	133,644	-0,751963	-4,45274	5,00E-05	0,0004193	yes
C16orf62	C16orf62	C16orf62	chr16:195667 shneg	shCHD1	OK	1,79995	1,06852	-0,752336	-2,63811	5,00E-05	0,0004193	yes

LTBP3	LTBP3	LTBP3	chr11:652925	shneg	shCHD1	OK	71,6126	42,4814	-0,753384	-3,68164	5,00E-05	0,0004193	yes
GAA	GAA	GAA	chr17:780753	shneg	shCHD1	OK	28,9237	17,1345	-0,755348	-4,0711	5,00E-05	0,0004193	yes
GRN	GRN	GRN	chr17:424224	shneg	shCHD1	OK	247,064	146,255	-0,756395	-4,44879	5,00E-05	0,0004193	yes
TMEM40	TMEM40	TMEM40	chr3:1277539	shneg	shCHD1	OK	7,65139	4,52151	-0,758917	-3,23045	5,00E-05	0,0004193	yes
MANBA	MANBA	MANBA	chr4:1035526	shneg	shCHD1	OK	18,9312	11,1817	-0,759627	-3,97212	5,00E-05	0,0004193	yes
PIGG	PIGG	PIGG	chr4:419223-[illegible]	shneg	shCHD1	OK	10,7192	6,33044	-0,759821	-2,71374	5,00E-05	0,0004193	yes
TBC1D8B	TBC1D8B	TBC1D8B	chrX:1060459	shneg	shCHD1	OK	6,60683	3,90033	-0,760361	-3,54109	5,00E-05	0,0004193	yes
MIRLET7BHG	MIRLET7BHG	MIRLET7BHG	chr22:464818	shneg	shCHD1	OK	1,84642	1,08978	-0,760694	-2,86979	5,00E-05	0,0004193	yes
DPH5	DPH5	DPH5	chr1:1014551	shneg	shCHD1	OK	18,9297	11,1719	-0,760777	-3,67246	5,00E-05	0,0004193	yes
LOC728431	LOC728431	LOC728431	chr1:3792047	shneg	shCHD1	OK	7,51276	4,43352	-0,760892	-3,00073	5,00E-05	0,0004193	yes
SOX18	SOX18	SOX18	chr20:626790	shneg	shCHD1	OK	12,087	7,13085	-0,76131	-3,52313	5,00E-05	0,0004193	yes
EPCAM	EPCAM	EPCAM	chr2:4759628	shneg	shCHD1	OK	42,1149	24,8414	-0,761584	-4,00813	5,00E-05	0,0004193	yes
XK	XK	XK	chrX:3754513	shneg	shCHD1	OK	2,2147	1,30583	-0,762146	-3,16261	5,00E-05	0,0004193	yes
MARK1	MARK1	MARK1	chr1:2207015	shneg	shCHD1	OK	1,77838	1,04759	-0,763497	-2,98075	5,00E-05	0,0004193	yes
KCNK4	KCNK4	KCNK4	chr11:640587	shneg	shCHD1	OK	1,53625	0,904763	-0,763805	-1,76826	0,00395	0,0184012	yes
TMEM150C	TMEM150C	TMEM150C	chr4:8340560	shneg	shCHD1	OK	3,32987	1,9602	-0,764463	-2,5024	5,00E-05	0,0004193	yes
N4BP2L1	N4BP2L1	N4BP2L1	chr13:329748	shneg	shCHD1	OK	2,15294	1,26458	-0,767652	-2,59309	5,00E-05	0,0004193	yes
LRRC73	LRRC73	LRRC73	chr6:4347470	shneg	shCHD1	OK	3,85763	2,26293	-0,76952	-2,79083	5,00E-05	0,0004193	yes
NPTXR	NPTXR	NPTXR	chr22:392144	shneg	shCHD1	OK	22,7503	13,3411	-0,770006	-4,25098	5,00E-05	0,0004193	yes
C1orf228	C1orf228	C1orf228	chr1:4514039	shneg	shCHD1	OK	1,31784	0,772707	-0,770183	-1,60141	0,0087	0,0355373	yes
NNT	NNT	NNT	chr5:4360279	shneg	shCHD1	OK	14,2465	8,33821	-0,772799	-4,05536	5,00E-05	0,0004193	yes
LOC338817	LOC338817	LOC338817	chr12:117009	shneg	shCHD1	OK	1,97407	1,15374	-0,774855	-2,72916	5,00E-05	0,0004193	yes
C10orf107	C10orf107	C10orf107	chr10:634227	shneg	shCHD1	OK	2,9892	1,74664	-0,775182	-1,99017	0,001	0,0058183	yes
PBXIP1	PBXIP1	PBXIP1	chr1:1549165	shneg	shCHD1	OK	38,2856	22,3637	-0,775642	-4,25487	5,00E-05	0,0004193	yes
FOXL2	FOXL2	FOXL2	chr3:1386630	shneg	shCHD1	OK	1,02861	0,600726	-0,775915	-1,8795	0,0024	0,0121606	yes
IDS	IDS	IDS	chrX:1485602	shneg	shCHD1	OK	47,6315	27,8115	-0,776234	-3,23202	5,00E-05	0,0004193	yes
SLC46A3	SLC46A3	SLC46A3	chr13:292742	shneg	shCHD1	OK	3,43231	2,00122	-0,778302	-3,21609	5,00E-05	0,0004193	yes
ZNF699	ZNF699	ZNF699	chr19:940598	shneg	shCHD1	OK	1,91331	1,11518	-0,778791	-2,10019	0,0006	0,00377139	yes
ITGA3	ITGA3	ITGA3	chr17:481333	shneg	shCHD1	OK	168,649	98,2817	-0,779027	-4,63635	5,00E-05	0,0004193	yes
SRPK3	SRPK3	SRPK3	chrX:1530464	shneg	shCHD1	OK	1,64519	0,958649	-0,779179	-1,78722	0,00175	0,00931439	yes
YPEL2	YPEL2	YPEL2	chr17:574090	shneg	shCHD1	OK	5,29036	3,08233	-0,779347	-3,82288	5,00E-05	0,0004193	yes
BMF	BMF	BMF	chr15:403800	shneg	shCHD1	OK	13,3753	7,79233	-0,779447	-4,02782	5,00E-05	0,0004193	yes
ZNF572	ZNF572	ZNF572	chr8:1259855	shneg	shCHD1	OK	1,05505	0,614528	-0,779759	-2,02174	0,0008	0,0048114	yes
PLA2R1	PLA2R1	PLA2R1	chr2:1607972	shneg	shCHD1	OK	6,24445	3,63132	-0,78208	-3,83093	5,00E-05	0,0004193	yes
SCPEP1	SCPEP1	SCPEP1	chr17:550554	shneg	shCHD1	OK	19,1899	11,1571	-0,782382	-3,83876	5,00E-05	0,0004193	yes
MFNG	MFNG	MFNG	chr22:378651	shneg	shCHD1	OK	2,93607	1,70605	-0,783224	-2,4305	5,00E-05	0,0004193	yes
LAMA5	LAMA5	LAMA5	chr20:608841	shneg	shCHD1	OK	78,8404	45,8047	-0,783439	-4,66703	5,00E-05	0,0004193	yes
TP53I13	TP53I13	TP53I13	chr17:278957	shneg	shCHD1	OK	44,9929	26,1236	-0,784342	-4,0636	5,00E-05	0,0004193	yes
SGCE	SGCE	SGCE	chr7:9421453	shneg	shCHD1	OK	9,3964	5,45259	-0,785165	-3,57418	5,00E-05	0,0004193	yes
NR1D2	NR1D2	NR1D2	chr3:2398675	shneg	shCHD1	OK	5,13509	2,97868	-0,785716	-3,8002	5,00E-05	0,0004193	yes
SLC30A1	SLC30A1	SLC30A1	chr1:2117483	shneg	shCHD1	OK	7,22472	4,18901	-0,786331	-3,45952	5,00E-05	0,0004193	yes
PSD4	PSD4	PSD4	chr2:1139315	shneg	shCHD1	OK	10,732	6,21795	-0,787411	-4,06012	5,00E-05	0,0004193	yes
CA4	CA4	CA4	chr17:582273	shneg	shCHD1	OK	25,412	14,7181	-0,787919	-3,77701	5,00E-05	0,0004193	yes
GNPDA2	GNPDA2	GNPDA2	chr4:4470416	shneg	shCHD1	OK	6,78399	3,9257	-0,789186	-3,35348	5,00E-05	0,0004193	yes
ZNF468	ZNF468	ZNF468	chr19:533417	shneg	shCHD1	OK	4,16578	2,40717	-0,791251	-3,60977	5,00E-05	0,0004193	yes
PDIA2	PDIA2	PDIA2	chr16:333117	shneg	shCHD1	OK	2,16674	1,25153	-0,79183	-2,07873	0,001	0,0058183	yes
DENND1B	DENND1B	DENND1B	chr1:1974738	shneg	shCHD1	OK	2,28462	1,31766	-0,793981	-2,87098	5,00E-05	0,0004193	yes
GLI2	GLI2	GLI2	chr2:1215548	shneg	shCHD1	OK	6,86587	3,95417	-0,796069	-4,03179	5,00E-05	0,0004193	yes
ANKRD36	ANKRD36	ANKRD36	chr2:9777923	shneg	shCHD1	OK	3,08768	1,77453	-0,799085	-3,65783	5,00E-05	0,0004193	yes
TMEM8B	TMEM8B	TMEM8B	chr9:3582922	shneg	shCHD1	OK	4,5523	2,61242	-0,801206	-3,29812	5,00E-05	0,0004193	yes
ZCCHC4	ZCCHC4	ZCCHC4	chr4:2531439	shneg	shCHD1	OK	13,1548	7,54621	-0,801761	-3,94552	5,00E-05	0,0004193	yes
MAMSTR	MAMSTR	MAMSTR	chr19:492162	shneg	shCHD1	OK	15,8907	9,10985	-0,802688	-3,81871	5,00E-05	0,0004193	yes
ELOVL6	ELOVL6	ELOVL6	chr4:1109702	shneg	shCHD1	OK	3,51609	2,0156	-0,802762	-3,33381	5,00E-05	0,0004193	yes
PCLO	PCLO	PCLO	chr7:8238332	shneg	shCHD1	OK	2,48588	1,42389	-0,803924	-4,05518	5,00E-05	0,0004193	yes
FHL1	FHL1	FHL1	chrX:1352288	shneg	shCHD1	OK	265,727	152,108	-0,804849	-4,74401	5,00E-05	0,0004193	yes
ARNTL	ARNTL	ARNTL	chr11:132993	shneg	shCHD1	OK	1,36212	0,779605	-0,805043	-2,00726	0,00025	0,00176854	yes
ABAT	ABAT	ABAT	chr16:876844	shneg	shCHD1	OK	1,51341	0,865818	-0,805665	-2,83329	5,00E-05	0,0004193	yes
ITGB2	ITGB2	ITGB2	chr21:463058	shneg	shCHD1	OK	11,2985	6,46385	-0,805668	-3,48581	5,00E-05	0,0004193	yes
ARHGDIG	ARHGDIG	ARHGDIG	chr16:330605	shneg	shCHD1	OK	4,34255	2,47679	-0,810074	-2,08674	0,00045	0,00293585	yes
HOXD9	HOXD9	HOXD9	chr2:1769874	shneg	shCHD1	OK	2,5612	1,4596	-0,811248	-2,36203	0,0003	0,0020738	yes
EMP1	EMP1	EMP1	chr12:133496	shneg	shCHD1	OK	6,5284	3,71827	-0,812096	-3,68291	5,00E-05	0,0004193	yes
FAM173A	FAM173A	FAM173A	chr16:771157	shneg	shCHD1	OK	21,6616	12,308	-0,815552	-2,82151	5,00E-05	0,0004193	yes
DFNB31	DFNB31	DFNB31	chr9:1171643	shneg	shCHD1	OK	12,2654	6,96461	-0,816483	-3,97192	5,00E-05	0,0004193	yes
ZBTB41	ZBTB41	ZBTB41	chr1:1971228	shneg	shCHD1	OK	3,15809	1,7914	-0,817961	-3,84212	5,00E-05	0,0004193	yes
SEMA4G	SEMA4G	SEMA4G	chr10:102732	shneg	shCHD1	OK	54,575	30,9463	-0,818475	-3,84393	5,00E-05	0,0004193	yes
TMEM65	TMEM65	TMEM65	chr8:1253231	shneg	shCHD1	OK	1,96153	1,11194	-0,818907	-3,05182	5,00E-05	0,0004193	yes
FHL2	FHL2	FHL2	chr2:1059772	shneg	shCHD1	OK	3,91002	2,2155	-0,819548	-2,61362	5,00E-05	0,0004193	yes
SLC3A1	SLC3A1	SLC3A1	chr2:4450259	shneg	shCHD1	OK	189,015	107,098	-0,819571	-3,82566	5,00E-05	0,0004193	yes
CPEB2	CPEB2	CPEB2	chr4:1500429	shneg	shCHD1	OK	0,822377	0,465901	-0,819775	-2,5626	5,00E-05	0,0004193	yes
LINC00176	LINC00176	LINC00176	chr20:626656	shneg	shCHD1	OK	0,937307	0,530949	-0,819948	-2,48918	0,00025	0,00176854	yes
AGAP2	AGAP2	AGAP2	chr12:581180	shneg	shCHD1	OK	1,78189	1,00905	-0,820413	-1,60188	0,00695	0,0293888	yes
DUSP9	DUSP9	DUSP9	chrX:1529078	shneg	shCHD1	OK	1,3978	0,790737	-0,821888	-2,05984	0,00095	0,00559279	yes
FABP5	FABP5	FABP5	chr8:8219271	shneg	shCHD1	OK	132,292	74,8296	-0,822042	-4,34181	5,00E-05	0,0004193	yes
MANEA	MANEA	MANEA	chr6:9602537	shneg	shCHD1	OK	6,40969	3,62189	-0,823513	-3,98113	5,00E-05	0,0004193	yes
SLC35A3	SLC35A3	SLC35A3	chr1:1004355	shneg	shCHD1	OK	7,68769	4,3409	-0,824555	-3,77673	5,00E-05	0,0004193	yes
FLJ39653	FLJ39653	FLJ39653	chr4:1622828	shneg	shCHD1	OK	1,91828	1,08316	-0,82457	-2,42341	0,0001	0,00078473	yes
HEXIM2	HEXIM2	HEXIM2	chr17:432382	shneg	shCHD1	OK	9,67925	5,46416	-0,824896	-3,41361	5,00E-05	0,0004193	yes
MR1	MR1	MR1	chr1:1810025	shneg	shCHD1	OK	1,63867	0,924385	-0,825957	-3,53925	5,00E-05	0,0004193	yes
ALPK1	ALPK1	ALPK1	chr4:1132184	shneg	shCHD1	OK	2,41599	1,36257	-0,826289	-3,57001	5,00E-05	0,0004193	yes
AGL	AGL	AGL	chr1:1003156	shneg	shCHD1	OK	4,14344	2,33529	-0,827224	-3,94495	5,00E-05	0,0004193	yes
CDKN2D	CDKN2D	CDKN2D	chr19:106771	shneg	shCHD1	OK	4,93117	2,77922	-0,827253	-2,47697	5,00E-05	0,0004193	yes
PLCD4	PLCD4	PLCD4	chr2:2194724	shneg	shCHD1	OK	3,04772	1,71748	-0,827434	-3,23521	5,00E-05	0,0004193	yes
CTSO	CTSO	CTSO	chr4:1568452	shneg	shCHD1	OK	9,69387	5,45939	-0,828332	-4,02831	5,00E-05	0,0004193	yes
RRAGB	RRAGB	RRAGB	chrX:5574410	shneg	shCHD1	OK	8,25098	4,64292	-0,829535	-3,74847	5,00E-05	0,0004193	yes
S100A6	S100A6	S100A6	chr1:1535070	shneg	shCHD1	OK	1229,55	691,864	-0,829566	-4,90383	5,00E-05	0,0004193	yes
TNFRSF14	TNFRSF14	TNFRSF14	chr1:2487207	shneg	shCHD1	OK	24,6876	13,8742	-0,831378	-4,08237	5,00E-05	0,0004193	yes
HRCT1	HRCT1	HRCT1	chr9:3590618	shneg	shCHD1	OK	8,12851	4,56742	-0,831613	-2,78148	5,00E-05	0,0004193	yes
JAG2	JAG2	JAG2	chr14:105608	shneg	shCHD1	OK	35,0279	19,6637	-0,832966	-4,63345	5,00E-05	0,0004193	yes
CCL16	CCL16	CCL16	chr17:343035	shneg	shCHD1	OK	1,45143	0,814619	-0,833275	-1,66223	0,0069	0,0292395	yes
WNK4	WNK4	WNK4	chr17:409326	shneg	shCHD1	OK	1,34134	0,752346	-0,83421	-2,63108	5,00E-05	0,0004193	yes
PARD3B	PARD3B	PARD3B	chr2:2054105	shneg	shCHD1	OK	0,979887	0,549507	-0,834477	-3,00923	5,00E-05	0,0004193	yes
TTC39B	TTC39B	TTC39B	chr9:1517084	shneg	shCHD1	OK	1,55096	0,869341	-0,835165	-2,38364	5,00E-05	0,0004193	yes
P2RY2	P2RY2	P2RY2	chr11:729293	shneg	shCHD1	OK	1,57589	0,882556	-0,836408	-2,21075	0,00015	0,00112331	yes

FLJ90757	FLJ90757	FLJ90757	chr17:790029	shneg	shCHD1	OK	14,5573	8,14749	-0,837319	-4,38112	5,00E-05	0,0004193 yes
MXD4	MXD4	MXD4	chr4:2249159	shneg	shCHD1	OK	23,3711	13,0658	-0,838923	-4,48923	5,00E-05	0,0004193 yes
BDNF	BDNF	BDNF	chr11:275283	shneg	shCHD1	OK	0,778441	0,434731	-0,840464	-1,5071	0,0019	0,00996052 yes
UAP1L1	UAP1L1	UAP1L1	chr9:1399719	shneg	shCHD1	OK	27,4856	15,3376	-0,841598	-4,50711	5,00E-05	0,0004193 yes
C10orf116	C10orf116	C10orf116	chr10:887281	shneg	shCHD1	OK	115,746	64,4702	-0,844257	-4,1927	5,00E-05	0,0004193 yes
FIBCD1	FIBCD1	FIBCD1	chr9:1337778	shneg	shCHD1	OK	0,873599	0,485825	-0,846533	-1,92737	0,0015	0,00814879 yes
MAN2B2	MAN2B2	MAN2B2	chr4:6576901	shneg	shCHD1	OK	16,7483	9,30413	-0,848069	-4,46991	5,00E-05	0,0004193 yes
ERGIC1	ERGIC1	ERGIC1	chr5:1722612	shneg	shCHD1	OK	141,338	78,5106	-0,848194	-4,93785	5,00E-05	0,0004193 yes
PCSK9	PCSK9	PCSK9	chr1:5550514	shneg	shCHD1	OK	18,3805	10,1914	-0,850816	-4,45126	5,00E-05	0,0004193 yes
MDGA1	MDGA1	MDGA1	chr6:3760028	shneg	shCHD1	OK	10,4483	5,78958	-0,851735	-4,57991	5,00E-05	0,0004193 yes
ULK1	ULK1	ULK1	chr12:132379	shneg	shCHD1	OK	31,4319	17,4028	-0,85291	-4,75377	5,00E-05	0,0004193 yes
GRIN3B	GRIN3B	GRIN3B	chr19:100043	shneg	shCHD1	OK	14,99	8,29835	-0,853103	-2,06664	0,00065	0,0040329 yes
C4orf34	C4orf34	C4orf34	chr4:3955254	shneg	shCHD1	OK	10,852	6,00723	-0,853195	-3,82844	5,00E-05	0,0004193 yes
ARMCX5-GPR	ARMCX5-GPR	ARMCX5-GPR	chrX:1018540	shneg	shCHD1	OK	3,27734	1,81342	-0,853812	-2,22072	0,0002	0,00144867 yes
MXRA8	MXRA8	MXRA8	chr1:1288070	shneg	shCHD1	OK	5,48912	3,03665	-0,854091	-3,55862	5,00E-05	0,0004193 yes
PPM1J	PPM1J	PPM1J	chr1:1132526	shneg	shCHD1	OK	3,22846	1,77912	-0,859686	-2,62425	5,00E-05	0,0004193 yes
ARHGEF37	ARHGEF37	ARHGEF37	chr5:1489611	shneg	shCHD1	OK	1,83219	1,00949	-0,859933	-3,25631	5,00E-05	0,0004193 yes
C1R	C1R	C1R	chr12:718751	shneg	shCHD1	OK	3,08879	1,7018	-0,859983	-2,8965	5,00E-05	0,0004193 yes
PTGDS	PTGDS	PTGDS	chr9:1398719	shneg	shCHD1	OK	5,89792	3,24234	-0,863173	-2,30439	0,00045	0,00293585 yes
C11orf54	C11orf54	C11orf54	chr11:934747	shneg	shCHD1	OK	26,0647	14,316	-0,864474	-4,47844	5,00E-05	0,0004193 yes
UPK1A	UPK1A	UPK1A	chr19:361577	shneg	shCHD1	OK	1,97288	1,08244	-0,866018	-1,803	0,0031	0,0150558 yes
CCDC114	CCDC114	CCDC114	chr19:487997	shneg	shCHD1	OK	13,2433	7,26162	-0,866905	-4,3299	5,00E-05	0,0004193 yes
CHD5	CHD5	CHD5	chr1:6161846	shneg	shCHD1	OK	6,38599	3,49628	-0,869089	-4,50973	5,00E-05	0,0004193 yes
CACNG4	CACNG4	CACNG4	chr17:649609	shneg	shCHD1	OK	149,035	81,5916	-0,869163	-5,09698	5,00E-05	0,0004193 yes
PITX2	PITX2	PITX2	chr4:1115385	shneg	shCHD1	OK	30,2494	16,5574	-0,869433	-4,35332	5,00E-05	0,0004193 yes
FAM13A	FAM13A	FAM13A	chr4:8963093	shneg	shCHD1	OK	2,88051	1,57615	-0,869925	-3,81883	5,00E-05	0,0004193 yes
BMP5	BMP5	BMP5	chr6:5562023	shneg	shCHD1	OK	44,3448	24,2619	-0,870076	-4,66868	5,00E-05	0,0004193 yes
UCP2	UCP2	UCP2	chr11:736857	shneg	shCHD1	OK	21,81	11,9312	-0,87025	-4,25507	5,00E-05	0,0004193 yes
RXFP4	RXFP4	RXFP4	chr1:1559114	shneg	shCHD1	OK	2,67374	1,46203	-0,870888	-1,98187	0,00155	0,00838119 yes
GPC1	GPC1	GPC1	chr2:2413751	shneg	shCHD1	OK	48,495	26,4778	-0,873053	-4,85212	5,00E-05	0,0004193 yes
NPW	NPW	NPW	chr16:206952	shneg	shCHD1	OK	22,5068	12,2868	-0,873254	-4,01047	5,00E-05	0,0004193 yes
KCNIP4	KCNIP4	KCNIP4	chr4:2073023	shneg	shCHD1	OK	1,51199	0,822979	-0,877516	-1,87043	0,0015	0,00814879 yes
SUPT3H	SUPT3H	SUPT3H	chr6:4479646	shneg	shCHD1	OK	5,36854	2,92157	-0,877789	-2,25388	0,00045	0,00293585 yes
LTBP2	LTBP2	LTBP2	chr14:749648	shneg	shCHD1	OK	21,1826	11,5232	-0,878338	-4,92496	5,00E-05	0,0004193 yes
LOC153684	LOC153684	LOC153684	chr5:4304223	shneg	shCHD1	OK	9,68261	5,26489	-0,878992	-4,10529	5,00E-05	0,0004193 yes
LOC10026816	LOC10026816	LOC10026816	chr5:1723817	shneg	shCHD1	OK	1,57568	0,856488	-0,879472	-1,59124	0,0051	0,0228051 yes
GPRC5C	GPRC5C	GPRC5C	chr17:724276	shneg	shCHD1	OK	107,532	58,4356	-0,879852	-4,87331	5,00E-05	0,0004193 yes
SMPDL3B	SMPDL3B	SMPDL3B	chr1:2826150	shneg	shCHD1	OK	3,47707	1,88881	-0,880392	-2,81102	5,00E-05	0,0004193 yes
ALDH3B1	ALDH3B1	ALDH3B1	chr11:677760	shneg	shCHD1	OK	7,98616	4,32482	-0,884863	-4,16114	5,00E-05	0,0004193 yes
LTK	LTK	LTK	chr15:417958	shneg	shCHD1	OK	2,80182	1,51691	-0,885225	-3,14006	5,00E-05	0,0004193 yes
LDHD	LDHD	LDHD	chr16:751457	shneg	shCHD1	OK	0,981319	0,53017	-0,888266	-1,65053	0,0041	0,0189794 yes
LOC10013235	LOC10013235	LOC10013235	chr5:4306528	shneg	shCHD1	OK	3,33854	1,80258	-0,889157	-2,76524	5,00E-05	0,0004193 yes
AASS	AASS	AASS	chr7:1217135	shneg	shCHD1	OK	1,4695	0,792781	-0,890332	-3,31584	5,00E-05	0,0004193 yes
CYBRD1	CYBRD1	CYBRD1	chr2:1723788	shneg	shCHD1	OK	22,226	11,976	-0,892103	-4,82752	5,00E-05	0,0004193 yes
EEPD1	EEPD1	EEPD1	chr7:3619283	shneg	shCHD1	OK	2,54204	1,36922	-0,892634	-3,71513	5,00E-05	0,0004193 yes
ASPSCR1	ASPSCR1	ASPSCR1	chr17:799354	shneg	shCHD1	OK	19,7226	10,6202	-0,893047	-4,34067	5,00E-05	0,0004193 yes
RIN1	RIN1	RIN1	chr11:660995	shneg	shCHD1	OK	67,4715	36,2222	-0,897405	-5,01171	5,00E-05	0,0004193 yes
SAMD13	SAMD13	SAMD13	chr1:8476404	shneg	shCHD1	OK	3,01934	1,61953	-0,898656	-2,26987	0,00015	0,00112331 yes
NPAS1	NPAS1	NPAS1	chr19:475241	shneg	shCHD1	OK	1,83502	0,983311	-0,900076	-2,32004	0,0002	0,00144867 yes
SLC5A3	SLC5A3	SLC5A3	chr21:354458	shneg	shCHD1	OK	6,53858	3,50372	-0,900091	-3,40449	5,00E-05	0,0004193 yes
FAM179B	FAM179B	FAM179B	chr14:454314	shneg	shCHD1	OK	2,14168	1,14631	-0,901752	-3,89909	5,00E-05	0,0004193 yes
ENTPD8	ENTPD8	ENTPD8	chr9:1403178	shneg	shCHD1	OK	2,58895	1,38528	-0,902189	-1,88838	0,0012	0,00680293 yes
SMAD7	SMAD7	SMAD7	chr18:464462	shneg	shCHD1	OK	16,3984	8,76848	-0,903153	-4,32685	5,00E-05	0,0004193 yes
CCDC78	CCDC78	CCDC78	chr16:771157	shneg	shCHD1	OK	5,80897	3,10459	-0,903882	-2,15579	0,0004	0,00266202 yes
AXL	AXL	AXL	chr19:417251	shneg	shCHD1	OK	7,32835	3,91479	-0,904555	-4,38505	5,00E-05	0,0004193 yes
WASF3	WASF3	WASF3	chr13:271318	shneg	shCHD1	OK	0,815402	0,435186	-0,905879	-2,46019	5,00E-05	0,0004193 yes
PYGB	PYGB	PYGB	chr20:252287	shneg	shCHD1	OK	223,4	119,132	-0,90707	-5,17645	5,00E-05	0,0004193 yes
SH3PXD2A	SH3PXD2A	SH3PXD2A	chr10:105353	shneg	shCHD1	OK	1,54173	0,821919	-0,907482	-4,40479	5,00E-05	0,0004193 yes
ZNF844	ZNF844	ZNF844	chr19:121755	shneg	shCHD1	OK	0,836233	0,445642	-0,908019	-1,95628	0,0013	0,00726051 yes
TLE4	TLE4	TLE4	chr9:8218687	shneg	shCHD1	OK	13,1267	6,96343	-0,914631	-4,75777	5,00E-05	0,0004193 yes
COL1A1	COL1A1	COL1A1	chr17:482614	shneg	shCHD1	OK	3,56765	1,89239	-0,914764	-4,2866	5,00E-05	0,0004193 yes
TPK1	TPK1	TPK1	chr7:1441490	shneg	shCHD1	OK	0,7444	0,394396	-0,916431	-1,58233	0,0039	0,018217 yes
AXIN2	AXIN2	AXIN2	chr17:635246	shneg	shCHD1	OK	17,3086	9,15997	-0,918072	-4,8364	5,00E-05	0,0004193 yes
LPCAT2	LPCAT2	LPCAT2	chr16:555429	shneg	shCHD1	OK	7,04437	3,72185	-0,920451	-4,53194	5,00E-05	0,0004193 yes
S100A5	S100A5	S100A5	chr1:1535096	shneg	shCHD1	OK	14,4178	7,60793	-0,922277	-3,24805	5,00E-05	0,0004193 yes
CPQ	CPQ	CPQ	chr8:9765749	shneg	shCHD1	OK	1,06015	0,558978	-0,923405	-1,78827	0,00325	0,0156858 yes
PTPRA	PTPRA	PTPRA	chr20:282137	shneg	shCHD1	OK	31,2351	16,4626	-0,923974	-3,91019	5,00E-05	0,0004193 yes
ZNF701	ZNF701	ZNF701	chr19:530735	shneg	shCHD1	OK	1,10814	0,583367	-0,925665	-2,98683	5,00E-05	0,0004193 yes
MAPK12	MAPK12	MAPK12	chr22:506913	shneg	shCHD1	OK	77,3453	40,6702	-0,927341	-5,07789	5,00E-05	0,0004193 yes
TRIM6	TRIM6	TRIM6	chr11:561733	shneg	shCHD1	OK	3,05555	1,60616	-0,927812	-2,01518	0,00125	0,00702633 yes
ETV7	ETV7	ETV7	chr6:3632199	shneg	shCHD1	OK	1,01664	0,532469	-0,933039	-1,24559	0,00585	0,0255919 yes
LINC00086	LINC00086	LINC00086	chrX:1345558	shneg	shCHD1	OK	1,53238	0,801623	-0,93478	-2,82694	5,00E-05	0,0004193 yes
LOC285084	LOC285084	LOC285084	chr2:1751907	shneg	shCHD1	OK	2,85075	1,48696	-0,938981	-3,47743	5,00E-05	0,0004193 yes
C1orf126	C1orf126	C1orf126	chr1:1492521	shneg	shCHD1	OK	3,00519	1,56604	-0,940328	-3,93147	5,00E-05	0,0004193 yes
EOMES	EOMES	EOMES	chr3:2775788	shneg	shCHD1	OK	2,17703	1,13376	-0,941248	-2,96056	5,00E-05	0,0004193 yes
APOL1	APOL1	APOL1	chr22:366491	shneg	shCHD1	OK	6,92312	3,59549	-0,945233	-4,30602	5,00E-05	0,0004193 yes
AGPAT4	AGPAT4	AGPAT4	chr6:1615510	shneg	shCHD1	OK	1,31527	0,682973	-0,94546	-3,57243	5,00E-05	0,0004193 yes
ARTN	ARTN	ARTN	chr1:4439899	shneg	shCHD1	OK	4,11236	2,13119	-0,948309	-3,24761	5,00E-05	0,0004193 yes
RAB31	RAB31	RAB31	chr18:970822	shneg	shCHD1	OK	1,36023	0,704383	-0,949419	-2,91173	5,00E-05	0,0004193 yes
NPDC1	NPDC1	NPDC1	chr9:1399339	shneg	shCHD1	OK	65,9133	34,0694	-0,952093	-5,09505	5,00E-05	0,0004193 yes
TMEM238	TMEM238	TMEM238	chr19:558906	shneg	shCHD1	OK	6,02301	3,11244	-0,952438	-2,2368	0,0009	0,00533451 yes
CDKL2	CDKL2	CDKL2	chr4:7650170	shneg	shCHD1	OK	1,14361	0,589858	-0,955151	-2,91795	5,00E-05	0,0004193 yes
LMBRD2	LMBRD2	LMBRD2	chr5:3610341	shneg	shCHD1	OK	4,40255	2,26307	-0,960057	-4,10735	5,00E-05	0,0004193 yes
C9orf89	C9orf89	C9orf89	chr9:9585844	shneg	shCHD1	OK	94,4653	48,5373	-0,960689	-5,01539	5,00E-05	0,0004193 yes
CYP27C1	CYP27C1	CYP27C1	chr2:1279414	shneg	shCHD1	OK	4,74771	2,43564	-0,962933	-4,52592	5,00E-05	0,0004193 yes
ABCA5	ABCA5	ABCA5	chr17:671441	shneg	shCHD1	OK	1,8537	0,950181	-0,964137	-3,59508	5,00E-05	0,0004193 yes
NAP1L5	NAP1L5	NAP1L5	chr4:8951364	shneg	shCHD1	OK	2,21924	1,13749	-0,964212	-2,1931	0,00085	0,00508358 yes
PIK3R3	PIK3R3	PIK3R3	chr1:4650581	shneg	shCHD1	OK	3,57289	1,83062	-0,964757	-4,41688	5,00E-05	0,0004193 yes
SCN9A	SCN9A	SCN9A	chr2:1670516	shneg	shCHD1	OK	12,3277	6,30684	-0,966917	-5,26437	5,00E-05	0,0004193 yes
METRN	METRN	METRN	chr16:765172	shneg	shCHD1	OK	40,9804	20,947	-0,968193	-4,73234	5,00E-05	0,0004193 yes
TRPM8	TRPM8	TRPM8	chr2:2348260	shneg	shCHD1	OK	16,262	8,30051	-0,97023	-5,20287	5,00E-05	0,0004193 yes
Sep 04	Sep 04	Sep 04	chr17:565976	shneg	shCHD1	OK	1,39759	0,711407	-0,974199	-1,71901	0,00095	0,00559279 yes
GRIN2D	GRIN2D	GRIN2D	chr19:488981	shneg	shCHD1	OK	4,71002	2,39198	-0,977529	-4,66758	5,00E-05	0,0004193 yes

RNASE4	RNASE4	RNASE4	chr14:211523	shneg	shCHD1	OK	8,93406	4,53211	-0,979132	-2,93549	5,00E-05	0,0004193	yes
CCDC88B	CCDC88B	CCDC88B	chr11:641076	shneg	shCHD1	OK	3,53484	1,79079	-0,98105	-4,38231	5,00E-05	0,0004193	yes
ARRB1	ARRB1	ARRB1	chr11:749711	shneg	shCHD1	OK	45,4392	23,0031	-0,982108	-5,67261	5,00E-05	0,0004193	yes
PION	PION	PION	chr7:7694006	shneg	shCHD1	OK	2,51095	1,27023	-0,983141	-3,48747	5,00E-05	0,0004193	yes
RNF152	RNF152	RNF152	chr18:594823	shneg	shCHD1	OK	3,03836	1,53654	-0,98361	-2,91998	5,00E-05	0,0004193	yes
FAM115C	FAM115C	FAM115C	chr7:1433180	shneg	shCHD1	OK	2,26948	1,14762	-0,983718	-3,83028	5,00E-05	0,0004193	yes
TMEM158	TMEM158	TMEM158	chr3:4526595	shneg	shCHD1	OK	10,0465	5,06668	-0,987575	-4,39818	5,00E-05	0,0004193	yes
C14orf28	C14orf28	C14orf28	chr14:453665	shneg	shCHD1	OK	1,49869	0,754206	-0,990675	-2,71009	5,00E-05	0,0004193	yes
NEUROD1	NEUROD1	NEUROD1	chr2:1825408	shneg	shCHD1	OK	3,03358	1,51979	-0,997145	-3,64565	5,00E-05	0,0004193	yes
MYCBPAP	MYCBPAP	MYCBPAP	chr17:485857	shneg	shCHD1	OK	0,824499	0,41283	-0,997971	-2,20145	0,0009	0,00533451	yes
IGFBP6	IGFBP6	IGFBP6	chr12:534914	shneg	shCHD1	OK	597,254	298,835	-0,998993	-5,8244	5,00E-05	0,0004193	yes
SFT2D1	SFT2D1	SFT2D1	chr6:1667335	shneg	shCHD1	OK	36,7062	18,3559	-0,999783	-4,4976	5,00E-05	0,0004193	yes
MGC16703	MGC16703	MGC16703	chr22:213540	shneg	shCHD1	OK	3,92856	1,95543	-1,00651	-2,10895	0,0007	0,00428773	yes
FAM113B	FAM113B	FAM113B	chr12:476022	shneg	shCHD1	OK	1,86215	0,924807	-1,00975	-2,05807	0,0009	0,00533451	yes
TAPBPL	TAPBPL	TAPBPL	chr12:656117	shneg	shCHD1	OK	10,0709	4,99996	-1,01021	-2,5886	0,0001	0,00078473	yes
RAMP1	RAMP1	RAMP1	chr2:2387681	shneg	shCHD1	OK	6,66513	3,29382	-1,01687	-2,95669	5,00E-05	0,0004193	yes
C9orf117	C9orf117	C9orf117	chr9:1304692	shneg	shCHD1	OK	4,05496	2,0019	-1,01832	-1,96372	0,00055	0,00350455	yes
LRP1	LRP1	LRP1	chr12:575222	shneg	shCHD1	OK	9,85607	4,86527	-1,01849	-5,61623	5,00E-05	0,0004193	yes
STEAP2	STEAP2	STEAP2	chr7:8984099	shneg	shCHD1	OK	2,21076	1,08551	-1,02616	-3,10577	5,00E-05	0,0004193	yes
LOC339166	LOC339166	LOC339166	chr17:567555	shneg	shCHD1	OK	2,87817	1,40715	-1,03238	-2,98522	5,00E-05	0,0004193	yes
TOB1	TOB1	TOB1	chr17:489395	shneg	shCHD1	OK	17,9395	8,70059	-1,04395	-4,94698	5,00E-05	0,0004193	yes
GALNTL1	GALNTL1	GALNTL1	chr14:697266	shneg	shCHD1	OK	1,58976	0,7679	-1,04982	-3,08271	5,00E-05	0,0004193	yes
SAPCD2	SAPCD2	SAPCD2	chr9:1399565	shneg	shCHD1	OK	38,0406	18,3222	-1,05395	-5,76389	5,00E-05	0,0004193	yes
DUSP1	DUSP1	DUSP1	chr5:1721950	shneg	shCHD1	OK	84,3862	40,5555	-1,05711	-5,78	5,00E-05	0,0004193	yes
UBA7	UBA7	UBA7	chr3:4984263	shneg	shCHD1	OK	1,86675	0,896102	-1,0588	-3,29627	5,00E-05	0,0004193	yes
C1orf88	C1orf88	C1orf88	chr1:1118891	shneg	shCHD1	OK	1,90479	0,912325	-1,06201	-2,86492	0,0001	0,00078473	yes
FGD3	FGD3	FGD3	chr9:9570960	shneg	shCHD1	OK	3,48666	1,66629	-1,06521	-4,2654	5,00E-05	0,0004193	yes
HES4	HES4	HES4	chr1:934341-[illegible]	shneg	shCHD1	OK	9,29437	4,43999	-1,0658	-3,57226	5,00E-05	0,0004193	yes
WBSCR28	WBSCR28	WBSCR28	chr7:7327548	shneg	shCHD1	OK	2,37212	1,13275	-1,06635	-1,85432	0,00285	0,0140225	yes
ALK	ALK	ALK	chr2:2941563	shneg	shCHD1	OK	1,09293	0,521542	-1,06735	-3,51671	5,00E-05	0,0004193	yes
EBF4	EBF4	EBF4	chr20:267352	shneg	shCHD1	OK	30,6704	14,5929	-1,07158	-5,68175	5,00E-05	0,0004193	yes
LHFP	LHFP	LHFP	chr13:399170	shneg	shCHD1	OK	3,75042	1,78426	-1,07172	-3,68691	5,00E-05	0,0004193	yes
EML6	EML6	EML6	chr2:5495214	shneg	shCHD1	OK	0,905352	0,430381	-1,07286	-3,6095	5,00E-05	0,0004193	yes
SLC44A1	SLC44A1	SLC44A1	chr9:1080069	shneg	shCHD1	OK	17,2347	8,18776	-1,07378	-5,64935	5,00E-05	0,0004193	yes
FLG	FLG	FLG	chr1:1522746	shneg	shCHD1	OK	0,962081	0,455247	-1,07951	-4,44515	5,00E-05	0,0004193	yes
CUEDC1	CUEDC1	CUEDC1	chr17:559403	shneg	shCHD1	OK	128,34	60,6731	-1,08084	-6,14281	5,00E-05	0,0004193	yes
CHST13	CHST13	CHST13	chr3:1262431	shneg	shCHD1	OK	1,56402	0,738318	-1,08294	-2,33618	0,0005	0,00321686	yes
FAM129A	FAM129A	FAM129A	chr1:1847601	shneg	shCHD1	OK	4,13441	1,95103	-1,08344	-5,34223	5,00E-05	0,0004193	yes
SLC16A5	SLC16A5	SLC16A5	chr17:730840	shneg	shCHD1	OK	74,7912	35,257	-1,08496	-5,94906	5,00E-05	0,0004193	yes
ANPEP	ANPEP	ANPEP	chr15:903281	shneg	shCHD1	OK	474,582	223,616	-1,08563	-6,53153	5,00E-05	0,0004193	yes
ICA1	ICA1	ICA1	chr7:8152814	shneg	shCHD1	OK	8,32864	3,92035	-1,0871	-4,57412	5,00E-05	0,0004193	yes
FLT3LG	FLT3LG	FLT3LG	chr19:499774	shneg	shCHD1	OK	4,31578	2,02579	-1,09114	-2,73279	5,00E-05	0,0004193	yes
CALML3	CALML3	CALML3	chr10:556692	shneg	shCHD1	OK	1,49149	0,699786	-1,09177	-1,96245	0,0028	0,0138449	yes
C5AR1	C5AR1	C5AR1	chr19:478131	shneg	shCHD1	OK	5,9648	2,79846	-1,09184	-4,53525	5,00E-05	0,0004193	yes
SECTM1	SECTM1	SECTM1	chr17:802788	shneg	shCHD1	OK	22,7208	10,6579	-1,09209	-5,49835	5,00E-05	0,0004193	yes
NTN4	NTN4	NTN4	chr12:960515	shneg	shCHD1	OK	22,0361	10,2732	-1,10099	-5,80213	5,00E-05	0,0004193	yes
ZNF133	ZNF133	ZNF133	chr20:182691	shneg	shCHD1	OK	6,7114	3,11951	-1,10529	-4,63689	5,00E-05	0,0004193	yes
ITGB8	ITGB8	ITGB8	chr7:2037072	shneg	shCHD1	OK	0,927415	0,43047	-1,1073	-3,90828	5,00E-05	0,0004193	yes
NFIA	NFIA	NFIA	chr1:6154294	shneg	shCHD1	OK	0,977832	0,452889	-1,11043	-4,07196	5,00E-05	0,0004193	yes
TCEAL1	TCEAL1	TCEAL1	chrX:1028836	shneg	shCHD1	OK	21,3917	9,90571	-1,11072	-5,12593	5,00E-05	0,0004193	yes
RRAGD	RRAGD	RRAGD	chr6:9007433	shneg	shCHD1	OK	2,42746	1,12112	-1,11451	-4,48713	5,00E-05	0,0004193	yes
GBP3	GBP3	GBP3	chr1:8947235	shneg	shCHD1	OK	3,2245	1,48662	-1,11704	-4,14804	5,00E-05	0,0004193	yes
ICAM4	ICAM4	ICAM4	chr19:103976	shneg	shCHD1	OK	11,6218	5,34383	-1,12088	-4,70395	5,00E-05	0,0004193	yes
SOWAHB	SOWAHB	SOWAHB	chr4:7781608	shneg	shCHD1	OK	0,790172	0,362598	-1,1238	-2,29331	0,0007	0,00428773	yes
ZNF347	ZNF347	ZNF347	chr19:536419	shneg	shCHD1	OK	1,40683	0,643936	-1,12746	-3,48195	5,00E-05	0,0004193	yes
IGF1R	IGF1R	IGF1R	chr15:991927	shneg	shCHD1	OK	0,744457	0,340636	-1,12796	-4,00179	5,00E-05	0,0004193	yes
ABHD15	ABHD15	ABHD15	chr17:278876	shneg	shCHD1	OK	8,94856	4,09345	-1,12834	-5,53942	5,00E-05	0,0004193	yes
OSBPL6	OSBPL6	OSBPL6	chr2:1790592	shneg	shCHD1	OK	1,13498	0,519091	-1,12861	-1,73705	0,0049	0,0220881	yes
VPS16	VPS16	VPS16	chr20:282137	shneg	shCHD1	OK	28,8407	13,0904	-1,13959	-3,95291	5,00E-05	0,0004193	yes
HGFAC	HGFAC	HGFAC	chr4:3443725	shneg	shCHD1	OK	1,56523	0,708579	-1,14337	-2,63493	0,0001	0,00078473	yes
PRDM5	PRDM5	PRDM5	chr4:1216159	shneg	shCHD1	OK	1,24893	0,565077	-1,14418	-2,61118	0,0001	0,00078473	yes
AHNAK2	AHNAK2	AHNAK2	chr14:105403	shneg	shCHD1	OK	46,2779	20,9274	-1,14493	-6,81008	5,00E-05	0,0004193	yes
TTLL7	TTLL7	TTLL7	chr1:8433505	shneg	shCHD1	OK	5,78994	2,61588	-1,14625	-5,12415	5,00E-05	0,0004193	yes
CD177	CD177	CD177	chr19:438578	shneg	shCHD1	OK	0,822273	0,371237	-1,14728	-2,14966	0,00225	0,0115391	yes
MSX2	MSX2	MSX2	chr5:1741515	shneg	shCHD1	OK	0,987828	0,445719	-1,14813	-2,21547	0,00075	0,00455593	yes
TRIM69	TRIM69	TRIM69	chr15:450285	shneg	shCHD1	OK	1,62178	0,729144	-1,15331	-2,2101	0,00025	0,00176854	yes
LMTK3	LMTK3	LMTK3	chr19:489885	shneg	shCHD1	OK	0,878442	0,394582	-1,15462	-3,11213	5,00E-05	0,0004193	yes
HSD3B7	HSD3B7	HSD3B7	chr16:309965	shneg	shCHD1	OK	43,7724	19,655	-1,15513	-6,15318	5,00E-05	0,0004193	yes
EFCAB4A	EFCAB4A	EFCAB4A	chr11:827584	shneg	shCHD1	OK	25,4576	11,3903	-1,1603	-5,72162	5,00E-05	0,0004193	yes
PDE7B	PDE7B	PDE7B	chr6:1361728	shneg	shCHD1	OK	1,01248	0,452626	-1,16151	-3,44651	5,00E-05	0,0004193	yes
DHDH	DHDH	DHDH	chr19:494369	shneg	shCHD1	OK	3,92486	1,75017	-1,16515	-2,89395	5,00E-05	0,0004193	yes
PADI2	PADI2	PADI2	chr1:1739325	shneg	shCHD1	OK	97,841	43,5653	-1,16726	-6,77598	5,00E-05	0,0004193	yes
SLC44A5	SLC44A5	SLC44A5	chr1:7566781	shneg	shCHD1	OK	6,78701	2,99732	-1,17911	-4,9992	5,00E-05	0,0004193	yes
ARMC12	ARMC12	ARMC12	chr6:3570485	shneg	shCHD1	OK	2,44999	1,08099	-1,18042	-2,47351	5,00E-05	0,0004193	yes
TNFRSF18	TNFRSF18	TNFRSF18	chr1:1138887	shneg	shCHD1	OK	10,7491	4,742	-1,18064	-4,49873	5,00E-05	0,0004193	yes
FSCN2	FSCN2	FSCN2	chr17:794954	shneg	shCHD1	OK	1,19618	0,525083	-1,18782	-2,04354	0,0007	0,00428773	yes
RGS11	RGS11	RGS11	chr16:318309	shneg	shCHD1	OK	9,21105	4,04241	-1,18815	-5,33715	5,00E-05	0,0004193	yes
PIK3IP1	PIK3IP1	PIK3IP1	chr22:316775	shneg	shCHD1	OK	2,74475	1,20203	-1,1912	-3,67972	5,00E-05	0,0004193	yes
GLDN	GLDN	GLDN	chr15:516337	shneg	shCHD1	OK	0,905863	0,396554	-1,19177	-3,26262	5,00E-05	0,0004193	yes
GLRA3	GLRA3	GLRA3	chr4:1755631	shneg	shCHD1	OK	6,57883	2,8791	-1,19221	-5,47021	5,00E-05	0,0004193	yes
LOC149837	LOC149837	LOC149837	chr20:547921	shneg	shCHD1	OK	1,01152	0,442013	-1,19437	-2,94071	5,00E-05	0,0004193	yes
NAV2	NAV2	NAV2	chr11:193722	shneg	shCHD1	OK	2,54457	1,10267	-1,20642	-5,55827	5,00E-05	0,0004193	yes
XG	XG	XG	chrX:2670092	shneg	shCHD1	OK	104,127	44,916	-1,21305	-6,92535	5,00E-05	0,0004193	yes
DENND2D	DENND2D	DENND2D	chr1:1117298	shneg	shCHD1	OK	1,09222	0,469823	-1,21708	-2,30533	0,00115	0,00656221	yes
ENPEP	ENPEP	ENPEP	chr4:1113972	shneg	shCHD1	OK	0,941493	0,404423	-1,21908	-3,3492	5,00E-05	0,0004193	yes
METTL7B	METTL7B	METTL7B	chr12:560753	shneg	shCHD1	OK	24,8164	10,5878	-1,22889	-4,7986	5,00E-05	0,0004193	yes
ARHGAP24	ARHGAP24	ARHGAP24	chr4:8639628	shneg	shCHD1	OK	2,48208	1,05623	-1,23262	-4,74632	5,00E-05	0,0004193	yes
FAM155A	FAM155A	FAM155A	chr13:107820	shneg	shCHD1	OK	3,58346	1,52094	-1,23639	-5,09643	5,00E-05	0,0004193	yes
LOC284837	LOC284837	LOC284837	chr21:452256	shneg	shCHD1	OK	2,01903	0,852905	-1,2432	-4,46702	5,00E-05	0,0004193	yes
MFAP3L	MFAP3L	MFAP3L	chr4:1709077	shneg	shCHD1	OK	1,71894	0,725908	-1,24366	-4,61097	5,00E-05	0,0004193	yes
ZNF575	ZNF575	ZNF575	chr19:440373	shneg	shCHD1	OK	1,12272	0,473796	-1,24466	-2,20667	0,00165	0,00886	yes
CLIC3	CLIC3	CLIC3	chr9:1398890	shneg	shCHD1	OK	1,94067	0,817935	-1,2465	-1,78909	0,0123	0,0472104	yes
S100A4	S100A4	S100A4	chr1:1535160	shneg	shCHD1	OK	3212,65	1352,19	-1,24846	-7,42528	5,00E-05	0,0004193	yes

C6orf132	C6orf132	C6orf132	chr6:4206885	shneg	shCHD1	OK	8,71135	3,62739	-1,26397	-6,43396	5,00E-05	0,0004193	yes
ITGA2B	ITGA2B	ITGA2B	chr17:424495	shneg	shCHD1	OK	2,08252	0,864662	-1,26812	-4,01192	5,00E-05	0,0004193	yes
MGAT5B	MGAT5B	MGAT5B	chr17:748647	shneg	shCHD1	OK	11,4343	4,74527	-1,2688	-6,25707	5,00E-05	0,0004193	yes
ASS1	ASS1	ASS1	chr9:1333200	shneg	shCHD1	OK	1416,89	586,617	-1,27223	-7,6941	5,00E-05	0,0004193	yes
MAFG-AS1	MAFG-AS1	MAFG-AS1	chr17:798857	shneg	shCHD1	OK	5,90816	2,43683	-1,2777	-4,76454	5,00E-05	0,0004193	yes
ADORA2A	ADORA2A	ADORA2A	chr22:248235	shneg	shCHD1	OK	23,3845	9,63695	-1,2789	-4,62673	5,00E-05	0,0004193	yes
ARMC7	ARMC7	ARMC7	chr17:731060	shneg	shCHD1	OK	19,4045	7,90865	-1,29489	-4,68524	5,00E-05	0,0004193	yes
TMEM229B	TMEM229B	TMEM229B	chr14:679369	shneg	shCHD1	OK	3,8048	1,54794	-1,29747	-5,52293	5,00E-05	0,0004193	yes
LOC678655	LOC678655	LOC678655	chr12:654816	shneg	shCHD1	OK	1,91154	0,777389	-1,29803	-2,98638	5,00E-05	0,0004193	yes
PTPRJ	PTPRJ	PTPRJ	chr11:480021	shneg	shCHD1	OK	52,3174	21,2702	-1,29846	-7,3301	5,00E-05	0,0004193	yes
ANKRD65	ANKRD65	ANKRD65	chr1:1353799	shneg	shCHD1	OK	2,06582	0,838747	-1,30041	-2,75202	5,00E-05	0,0004193	yes
C10orf47	C10orf47	C10orf47	chr10:118653	shneg	shCHD1	OK	11,7732	4,77328	-1,30245	-6,33519	5,00E-05	0,0004193	yes
TRIM58	TRIM58	TRIM58	chr1:2480205	shneg	shCHD1	OK	1,93968	0,783479	-1,30785	-4,7795	5,00E-05	0,0004193	yes
ABCC6	ABCC6	ABCC6	chr16:162434	shneg	shCHD1	OK	3,27989	1,32441	-1,30829	-3,50765	5,00E-05	0,0004193	yes
PROS1	PROS1	PROS1	chr3:9359188	shneg	shCHD1	OK	30,229	12,1364	-1,31659	-7,04342	5,00E-05	0,0004193	yes
SUSD3	SUSD3	SUSD3	chr9:9582098	shneg	shCHD1	OK	48,5122	19,2465	-1,33375	-6,62401	5,00E-05	0,0004193	yes
TTC9B	TTC9B	TTC9B	chr19:407219	shneg	shCHD1	OK	1,88098	0,745857	-1,33451	-1,77907	0,01015	0,040279	yes
GCA	GCA	GCA	chr2:1632005	shneg	shCHD1	OK	2,37603	0,940967	-1,33634	-4,31449	5,00E-05	0,0004193	yes
THBS3	THBS3	THBS3	chr1:1551653	shneg	shCHD1	OK	32,748	12,9435	-1,33917	-7,12474	5,00E-05	0,0004193	yes
PTPN6	PTPN6	PTPN6	chr12:705573	shneg	shCHD1	OK	3,32416	1,31108	-1,34223	-4,13358	5,00E-05	0,0004193	yes
TNFSF15	TNFSF15	TNFSF15	chr9:1175469	shneg	shCHD1	OK	2,53001	0,996807	-1,34375	-5,6242	5,00E-05	0,0004193	yes
MTMR11	MTMR11	MTMR11	chr1:1499005	shneg	shCHD1	OK	24,1484	9,4703	-1,35045	-6,81072	5,00E-05	0,0004193	yes
PTCH2	PTCH2	PTCH2	chr1:4528551	shneg	shCHD1	OK	2,97467	1,16504	-1,35236	-5,06701	5,00E-05	0,0004193	yes
DKK3	DKK3	DKK3	chr11:119845	shneg	shCHD1	OK	25,8969	10,0366	-1,36752	-6,93554	5,00E-05	0,0004193	yes
HNF4G	HNF4G	HNF4G	chr8:7645220	shneg	shCHD1	OK	2,48934	0,958876	-1,37635	-4,97629	5,00E-05	0,0004193	yes
PTRH1	PTRH1	PTRH1	chr9:1304692	shneg	shCHD1	OK	55,998	21,5606	-1,37698	-6,09593	5,00E-05	0,0004193	yes
PCDHA4	PCDHA4	PCDHA4	chr5:1401658	shneg	shCHD1	OK	0,919125	0,353206	-1,37975	-1,95031	0,0046	0,0208912	yes
SLAIN1	SLAIN1	SLAIN1	chr13:782719	shneg	shCHD1	OK	0,91839	0,352615	-1,38101	-1,98018	0,0001	0,00078473	yes
KIAA1324	KIAA1324	KIAA1324	chr1:1096565	shneg	shCHD1	OK	2,96892	1,13652	-1,38532	-6,09999	5,00E-05	0,0004193	yes
STRA13	STRA13	STRA13	chr17:799765	shneg	shCHD1	OK	105,542	40,0733	-1,3971	-7,04846	5,00E-05	0,0004193	yes
PNMT	PNMT	PNMT	chr17:378245	shneg	shCHD1	OK	2,63318	0,995212	-1,40373	-2,8482	5,00E-05	0,0004193	yes
KCNMB1	KCNMB1	KCNMB1	chr5:1697808	shneg	shCHD1	OK	1,09439	0,413237	-1,40509	-2,17024	0,0018	0,00951868	yes
FAM113A	FAM113A	FAM113A	chr20:281597	shneg	shCHD1	OK	58,8749	21,9847	-1,42115	-7,59955	5,00E-05	0,0004193	yes
ITGB4	ITGB4	ITGB4	chr17:737175	shneg	shCHD1	OK	187,003	69,5379	-1,42719	-8,51479	5,00E-05	0,0004193	yes
DPEP1	DPEP1	DPEP1	chr16:896797	shneg	shCHD1	OK	4,68509	1,73551	-1,43272	-4,38686	5,00E-05	0,0004193	yes
GIMAP2	GIMAP2	GIMAP2	chr7:1503827	shneg	shCHD1	OK	2,48512	0,920265	-1,43319	-3,15486	5,00E-05	0,0004193	yes
PLEKHB1	PLEKHB1	PLEKHB1	chr11:733572	shneg	shCHD1	OK	0,955816	0,353758	-1,43397	-1,75234	0,00035	0,00236888	yes
GCNT4	GCNT4	GCNT4	chr5:7432328	shneg	shCHD1	OK	2,45186	0,906255	-1,43589	-4,72736	5,00E-05	0,0004193	yes
NMU	NMU	NMU	chr4:5646139	shneg	shCHD1	OK	20,4049	7,50171	-1,44362	-5,67861	5,00E-05	0,0004193	yes
P2RY6	P2RY6	P2RY6	chr11:729755	shneg	shCHD1	OK	5,00759	1,83758	-1,44631	-4,51779	5,00E-05	0,0004193	yes
ARHGEF35	ARHGEF35	ARHGEF35	chr7:1438831	shneg	shCHD1	OK	1,54634	0,566569	-1,44853	-3,40819	5,00E-05	0,0004193	yes
BTN1A1	BTN1A1	BTN1A1	chr6:2650149	shneg	shCHD1	OK	1,87531	0,684871	-1,45323	-3,945	5,00E-05	0,0004193	yes
UPK3B	UPK3B	UPK3B	chr7:7613974	shneg	shCHD1	OK	8,85261	3,18815	-1,47339	-5,66354	5,00E-05	0,0004193	yes
ABCA8	ABCA8	ABCA8	chr17:668634	shneg	shCHD1	OK	4,26178	1,51251	-1,49451	-6,73637	5,00E-05	0,0004193	yes
CAMK2B	CAMK2B	CAMK2B	chr7:4425674	shneg	shCHD1	OK	2,84625	1,00276	-1,50509	-5,50408	5,00E-05	0,0004193	yes
NAPSA	NAPSA	NAPSA	chr19:508617	shneg	shCHD1	OK	0,979929	0,344367	-1,50873	-1,88468	0,00965	0,0388146	yes
LOC400891	LOC400891	LOC400891	chr22:214002	shneg	shCHD1	OK	16,7987	5,89177	-1,51158	-7,9067	5,00E-05	0,0004193	yes
LY75	LY75	LY75	chr2:1606251	shneg	shCHD1	OK	1,94019	0,68038	-1,51178	-4,35055	5,00E-05	0,0004193	yes
TSPAN6	TSPAN6	TSPAN6	chrX:9988379	shneg	shCHD1	OK	11,349	3,9767	-1,51292	-6,62709	5,00E-05	0,0004193	yes
AMDHD1	AMDHD1	AMDHD1	chr12:963370	shneg	shCHD1	OK	0,856169	0,297311	-1,52592	-2,43322	0,00155	0,00838119	yes
ATP10D	ATP10D	ATP10D	chr4:4748740	shneg	shCHD1	OK	1,41048	0,48918	-1,52775	-5,19014	5,00E-05	0,0004193	yes
CPXM1	CPXM1	CPXM1	chr20:277471	shneg	shCHD1	OK	22,1091	7,63201	-1,53451	-7,56592	5,00E-05	0,0004193	yes
TRPS1	TRPS1	TRPS1	chr8:1164207	shneg	shCHD1	OK	1,01782	0,350765	-1,53691	-5,44324	5,00E-05	0,0004193	yes
F10	F10	F10	chr13:113777	shneg	shCHD1	OK	1,02351	0,351202	-1,54316	-2,06566	0,00335	0,0160296	yes
GRAMD2	GRAMD2	GRAMD2	chr15:724521	shneg	shCHD1	OK	1,30376	0,442513	-1,55889	-3,79397	5,00E-05	0,0004193	yes
MIR100HG	MIR100HG	MIR100HG	chr11:121959	shneg	shCHD1	OK	3,42891	1,15885	-1,56506	-5,47695	5,00E-05	0,0004193	yes
CRABP2	CRABP2	CRABP2	chr1:1566693	shneg	shCHD1	OK	132,954	44,7795	-1,57001	-8,42236	5,00E-05	0,0004193	yes
EPS8	EPS8	EPS8	chr12:157730	shneg	shCHD1	OK	3,24959	1,0925	-1,57263	-6,02917	5,00E-05	0,0004193	yes
S100A2	S100A2	S100A2	chr1:1535335	shneg	shCHD1	OK	72,3527	24,3033	-1,57389	-7,85618	5,00E-05	0,0004193	yes
CNR1	CNR1	CNR1	chr6:8884958	shneg	shCHD1	OK	0,804774	0,269973	-1,57577	-3,4381	5,00E-05	0,0004193	yes
ZNF350	ZNF350	ZNF350	chr19:524675	shneg	shCHD1	OK	1,47903	0,49565	-1,57726	-3,38472	5,00E-05	0,0004193	yes
LOC401164	LOC401164	LOC401164	chr4:1893767	shneg	shCHD1	OK	4,72928	1,57001	-1,59085	-2,17359	0,0008	0,0048114	yes
CDA	CDA	CDA	chr1:2091544	shneg	shCHD1	OK	8,22685	2,72341	-1,59492	-4,64325	5,00E-05	0,0004193	yes
TNC	TNC	TNC	chr9:1177818	shneg	shCHD1	OK	10,3423	3,40652	-1,60219	-8,37608	5,00E-05	0,0004193	yes
ABHD11-AS1	ABHD11-AS1	ABHD11-AS1	chr7:7314939	shneg	shCHD1	OK	4,23322	1,38925	-1,60745	-1,97001	0,004	0,018603	yes
BDH2	BDH2	BDH2	chr4:1039987	shneg	shCHD1	OK	3,81176	1,24929	-1,60935	-5,71439	5,00E-05	0,0004193	yes
MIA2	MIA2	MIA2	chr14:397031	shneg	shCHD1	OK	2,41195	0,787256	-1,6153	-4,25192	5,00E-05	0,0004193	yes
GOLGA7B	GOLGA7B	GOLGA7B	chr10:996099	shneg	shCHD1	OK	48,6442	15,3043	-1,66833	-9,30554	5,00E-05	0,0004193	yes
AMIGO2	AMIGO2	AMIGO2	chr12:474694	shneg	shCHD1	OK	29,2912	9,176	-1,67453	-8,72933	5,00E-05	0,0004193	yes
LEPREL1	LEPREL1	LEPREL1	chr3:1896745	shneg	shCHD1	OK	62,5058	19,495	-1,68089	-8,9839	5,00E-05	0,0004193	yes
EGR4	EGR4	EGR4	chr2:7351805	shneg	shCHD1	OK	1,07222	0,332837	-1,68771	-3,03048	5,00E-05	0,0004193	yes
CCDC162P	CCDC162P	CCDC162P	chr6:1096155	shneg	shCHD1	OK	7,51225	2,31881	-1,69586	-3,93168	5,00E-05	0,0004193	yes
P2RX6	P2RX6	P2RX6	chr22:213694	shneg	shCHD1	OK	2,13692	0,653617	-1,70901	-4,50609	5,00E-05	0,0004193	yes
LINC00475	LINC00475	LINC00475	chr9:9490374	shneg	shCHD1	OK	3,13082	0,956018	-1,71143	-4,66168	5,00E-05	0,0004193	yes
VWA1	VWA1	VWA1	chr1:1370902	shneg	shCHD1	OK	94,9751	28,9108	-1,71594	-9,84582	5,00E-05	0,0004193	yes
TSPAN1	TSPAN1	TSPAN1	chr1:4664074	shneg	shCHD1	OK	38,936	11,7906	-1,72346	-8,53499	5,00E-05	0,0004193	yes
ENPP4	ENPP4	ENPP4	chr6:4609770	shneg	shCHD1	OK	4,9778	1,49287	-1,73742	-7,41636	5,00E-05	0,0004193	yes
CRTAC1	CRTAC1	CRTAC1	chr10:996099	shneg	shCHD1	OK	3,31757	0,967361	-1,778	-1,61002	0,00635	0,0272991	yes
HOXD13	HOXD13	HOXD13	chr2:1769575	shneg	shCHD1	OK	1,23851	0,360826	-1,77923	-3,39014	5,00E-05	0,0004193	yes
PMEPA1	PMEPA1	PMEPA1	chr20:562234	shneg	shCHD1	OK	49,337	14,2923	-1,78743	-9,84587	5,00E-05	0,0004193	yes
FBXO32	FBXO32	FBXO32	chr8:1245101	shneg	shCHD1	OK	1,76912	0,509808	-1,79501	-6,31529	5,00E-05	0,0004193	yes
FOXP2	FOXP2	FOXP2	chr7:1137263	shneg	shCHD1	OK	2,23353	0,638904	-1,80565	-3,57704	5,00E-05	0,0004193	yes
GPR173	GPR173	GPR173	chrX:5307850	shneg	shCHD1	OK	3,90995	1,11496	-1,81016	-7,01382	5,00E-05	0,0004193	yes
SSC5D	SSC5D	SSC5D	chr19:559998	shneg	shCHD1	OK	10,6834	3,04416	-1,81125	-8,58066	5,00E-05	0,0004193	yes
IFI16	IFI16	IFI16	chr1:1589796	shneg	shCHD1	OK	0,826723	0,23382	-1,822	-2,90603	5,00E-05	0,0004193	yes
CTNNA2	CTNNA2	CTNNA2	chr2:7974005	shneg	shCHD1	OK	1,05377	0,296205	-1,83089	-3,75201	5,00E-05	0,0004193	yes
HOXD4	HOXD4	HOXD4	chr2:1770161	shneg	shCHD1	OK	2,61899	0,73587	-1,83149	-3,51413	5,00E-05	0,0004193	yes
C7orf69	C7orf69	C7orf69	chr7:4781424	shneg	shCHD1	OK	5,7941	1,61979	-1,83878	-3,00598	5,00E-05	0,0004193	yes
CACNA1I	CACNA1I	CACNA1I	chr22:399667	shneg	shCHD1	OK	0,736621	0,205181	-1,84402	-5,30839	5,00E-05	0,0004193	yes
PRICKLE2	PRICKLE2	PRICKLE2	chr3:6405363	shneg	shCHD1	OK	1,04887	0,289523	-1,85708	-5,24914	5,00E-05	0,0004193	yes
APOL3	APOL3	APOL3	chr22:365363	shneg	shCHD1	OK	0,988266	0,271575	-1,86355	-2,49183	5,00E-05	0,0004193	yes
CCDC80	CCDC80	CCDC80	chr3:1123234	shneg	shCHD1	OK	6,16576	1,69238	-1,86523	-8,17631	5,00E-05	0,0004193	yes
SLC16A4	SLC16A4	SLC16A4	chr1:1109054	shneg	shCHD1	OK	13,9408	3,81559	-1,86933	-8,45007	5,00E-05	0,0004193	yes

HOXD-AS1	HOXD-AS1	HOXD-AS1	chr2:1770379 shneg	shCHD1	OK	0,867928	0,23625	-1,87726	-3,68947	5,00E-05	0,0004193	yes
LINC00478	LINC00478	LINC00478	chr21:174428 shneg	shCHD1	OK	4,65674	1,25593	-1,89057	-6,97149	5,00E-05	0,0004193	yes
C19orf77	C19orf77	C19orf77	chr19:347440 shneg	shCHD1	OK	4,50397	1,21045	-1,89565	-3,83722	5,00E-05	0,0004193	yes
ABCA1	ABCA1	ABCA1	chr9:1075432 shneg	shCHD1	OK	1,60729	0,429173	-1,905	-7,29114	5,00E-05	0,0004193	yes
LOC10013041	LOC10013041	LOC10013041	chr1:852952-8 shneg	shCHD1	OK	3,86921	1,03226	-1,90623	-2,10601	0,0034	0,0162243	yes
PADI3	PADI3	PADI3	chr1:1757559 shneg	shCHD1	OK	52,5499	13,9552	-1,91288	-10,3732	5,00E-05	0,0004193	yes
LILRB1	LILRB1	LILRB1	chr19:551286 shneg	shCHD1	OK	6,06691	1,60351	-1,91973	-7,25182	5,00E-05	0,0004193	yes
PIP5KL1	PIP5KL1	PIP5KL1	chr9:1306838 shneg	shCHD1	OK	8,44134	2,22836	-1,92149	-5,7099	5,00E-05	0,0004193	yes
COL9A3	COL9A3	COL9A3	chr20:614484 shneg	shCHD1	OK	30,3258	7,89154	-1,94216	-6,80619	5,00E-05	0,0004193	yes
MCHR1	MCHR1	MCHR1	chr22:410751 shneg	shCHD1	OK	1,04053	0,264599	-1,97543	-3,26856	5,00E-05	0,0004193	yes
FAT4	FAT4	FAT4	chr4:1262375 shneg	shCHD1	OK	0,869511	0,217808	-1,99715	-7,2862	5,00E-05	0,0004193	yes
TMEM88B	TMEM88B	TMEM88B	chr1:1361507 shneg	shCHD1	OK	17,7548	4,43279	-2,00192	-4,87937	5,00E-05	0,0004193	yes
CXorf57	CXorf57	CXorf57	chrX:1058551 shneg	shCHD1	OK	0,804038	0,199909	-2,00792	-3,54667	5,00E-05	0,0004193	yes
GPR56	GPR56	GPR56	chr16:576539 shneg	shCHD1	OK	4,01344	0,997338	-2,00869	-7,05613	5,00E-05	0,0004193	yes
PLCB4	PLCB4	PLCB4	chr20:904970 shneg	shCHD1	OK	7,87406	1,95479	-2,0101	-9,64815	5,00E-05	0,0004193	yes
SMARCA2	SMARCA2	SMARCA2	chr9:2015341 shneg	shCHD1	OK	2,33899	0,578857	-2,01461	-7,09627	5,00E-05	0,0004193	yes
GLI1	GLI1	GLI1	chr12:578539 shneg	shCHD1	OK	27,1843	6,67142	-2,02671	-10,2725	5,00E-05	0,0004193	yes
NIPSNAP3B	NIPSNAP3B	NIPSNAP3B	chr9:1075264 shneg	shCHD1	OK	0,903187	0,221319	-2,0289	-2,40524	0,0033	0,0158557	yes
PRPH	PRPH	PRPH	chr12:496889 shneg	shCHD1	OK	2,0501	0,472387	-2,11765	-4,13866	5,00E-05	0,0004193	yes
HOXD-AS2	HOXD-AS2	HOXD-AS2	chr2:1769995 shneg	shCHD1	OK	4,8129	1,10816	-2,11874	-3,17766	5,00E-05	0,0004193	yes
KRT20	KRT20	KRT20	chr17:390321 shneg	shCHD1	OK	6,51106	1,45311	-2,16375	-6,70073	5,00E-05	0,0004193	yes
SSBP2	SSBP2	SSBP2	chr5:8025650 shneg	shCHD1	OK	1,09734	0,242936	-2,17536	-2,1259	0,00045	0,00293585	yes
DMRT2	DMRT2	DMRT2	chr9:1050345 shneg	shCHD1	OK	1,01939	0,222106	-2,19839	-2,93162	5,00E-05	0,0004193	yes
KLF8	KLF8	KLF8	chrX:5625882 shneg	shCHD1	OK	1,19421	0,258459	-2,20805	-5,38582	5,00E-05	0,0004193	yes
CACNA1A	CACNA1A	CACNA1A	chr19:133172 shneg	shCHD1	OK	19,333	4,11771	-2,23116	-11,9388	5,00E-05	0,0004193	yes
JSRP1	JSRP1	JSRP1	chr19:225224 shneg	shCHD1	OK	1,67328	0,355693	-2,23398	-2,59277	0,00185	0,00975716	yes
C9orf125	C9orf125	C9orf125	chr9:1042376 shneg	shCHD1	OK	6,8899	1,46257	-2,23598	-7,57731	5,00E-05	0,0004193	yes
ADAMTS15	ADAMTS15	ADAMTS15	chr11:130318 shneg	shCHD1	OK	14,3224	2,89769	-2,3053	-11,8322	5,00E-05	0,0004193	yes
AK5	AK5	AK5	chr1:7774766 shneg	shCHD1	OK	8,51097	1,71913	-2,30764	-9,47418	5,00E-05	0,0004193	yes
STARD13	STARD13	STARD13	chr13:336772 shneg	shCHD1	OK	0,821538	0,163958	-2,325	-3,23416	5,00E-05	0,0004193	yes
HOXD8	HOXD8	HOXD8	chr2:1769944 shneg	shCHD1	OK	2,86972	0,563235	-2,3491	-4,56564	5,00E-05	0,0004193	yes
BAHCC1	BAHCC1	BAHCC1	chr17:793735 shneg	shCHD1	OK	2,15779	0,401016	-2,42782	-9,06946	5,00E-05	0,0004193	yes
NEUROG2	NEUROG2	NEUROG2	chr4:1134346 shneg	shCHD1	OK	19,0302	3,4787	-2,45167	-10,9818	5,00E-05	0,0004193	yes
SCG5	SCG5	SCG5	chr15:329338 shneg	shCHD1	OK	7,152	1,29694	-2,46324	-6,26403	5,00E-05	0,0004193	yes
NES	NES	NES	chr1:1566385 shneg	shCHD1	OK	17,5164	3,15265	-2,47407	-12,6063	5,00E-05	0,0004193	yes
ZNF462	ZNF462	ZNF462	chr9:1096253 shneg	shCHD1	OK	3,13746	0,532536	-2,55865	-9,9039	5,00E-05	0,0004193	yes
FAM46C	FAM46C	FAM46C	chr1:1181486 shneg	shCHD1	OK	0,987062	0,167471	-2,55923	-5,62262	5,00E-05	0,0004193	yes
SCEL	SCEL	SCEL	chr13:781098 shneg	shCHD1	OK	8,77028	1,48701	-2,56021	-9,88864	5,00E-05	0,0004193	yes
H19	H19	H19	chr11:201640 shneg	shCHD1	OK	39,17	6,49447	-2,59247	-12,9506	5,00E-05	0,0004193	yes
SFRP5	SFRP5	SFRP5	chr10:995265 shneg	shCHD1	OK	0,749249	0,123031	-2,60643	-2,54274	0,01265	0,0482745	yes
IFI27	IFI27	IFI27	chr14:945770 shneg	shCHD1	OK	120,055	19,46	-2,62511	-12,3472	5,00E-05	0,0004193	yes
RNF128	RNF128	RNF128	chrX:1059370 shneg	shCHD1	OK	20,9839	3,2436	-2,69362	-12,6553	5,00E-05	0,0004193	yes
CXXC11	CXXC11	CXXC11	chr2:2428118 shneg	shCHD1	OK	0,75147	0,105819	-2,82812	-2,32771	0,0088	0,0358932	yes
SAMD11	SAMD11	SAMD11	chr1:861120-8 shneg	shCHD1	OK	4,80576	0,64728	-2,8923	-2,3725	0,0034	0,0162243	yes
VIT	VIT	VIT	chr2:3692383 shneg	shCHD1	OK	14,768	1,83891	-3,00555	-11,1988	5,00E-05	0,0004193	yes
PLCH2	PLCH2	PLCH2	chr1:2407753 shneg	shCHD1	OK	30,583	3,68911	-3,05139	-15,4903	5,00E-05	0,0004193	yes
LILRA2	LILRA2	LILRA2	chr19:550852 shneg	shCHD1	OK	27,7526	3,30164	-3,07136	-12,5892	5,00E-05	0,0004193	yes
NOTUM	NOTUM	NOTUM	chr17:799103 shneg	shCHD1	OK	169,935	19,9807	-3,08831	-16,927	5,00E-05	0,0004193	yes
KSR2	KSR2	KSR2	chr12:117890 shneg	shCHD1	OK	1,99548	0,232059	-3,10417	-11,8184	5,00E-05	0,0004193	yes
ACY3	ACY3	ACY3	chr11:674100 shneg	shCHD1	OK	1,37624	0,152754	-3,17144	-2,53222	0,0058	0,0254211	yes
FLRT3	FLRT3	FLRT3	chr20:139761 shneg	shCHD1	OK	1,11806	0,12048	-3,21413	-4,26459	5,00E-05	0,0004193	yes
GLI3	GLI3	GLI3	chr7:4200054 shneg	shCHD1	OK	1,26182	0,127218	-3,31013	-7,68974	5,00E-05	0,0004193	yes
NRP2	NRP2	NRP2	chr2:2065472 shneg	shCHD1	OK	2,13926	0,192695	-3,47272	-5,1153	5,00E-05	0,0004193	yes
LOC375295	LOC375295	LOC375295	chr2:1774943 shneg	shCHD1	OK	6,92168	0,419817	-4,04329	-6,27169	5,00E-05	0,0004193	yes
