# Supplementary material for: CHD1 loss negatively influences metastasis-free survival in R0-resected prostate cancer patients and promotes spontaneous metastasis in vivo
Source: Cancer Gene Ther. 2021 Jan 7;29(1):49–61. doi: 10.1038/s41417-020-00288-z (PMC8761572; doi:10.1038/s41417-020-00288-z)
Supplement: Supplementary file 11 — Ct values qPCR RUNX3 [file 41417_2020_288_MOESM11_ESM.pdf]

RUNX qPCR

	RUNX3	SNRPD3	TG - Mittelwert HG	2^[-(TG-Mittelwert HG)]	Normalisiert	Mittelwert
		**21,77**			**0,0490**	
ARCaP NT1	26,16	22,09	4,39	0,0476956	0,974080574	1
ARCaP NT1	26,17	21,72	4,40	0,047366143	0,967352108	
ARCaP NT1	26,04	21,50	4,27	0,051832472	1,058567318	
		**22,03**				
ARCaP sh2_1	25,71	22,22	3,68	0,078020659	1,593405013	1,586295467
ARCaP sh2_1	25,75	21,85	3,72	0,07588718	1,549833269	
ARCaP sh2_1	25,69	22,02	3,66	0,079109787	1,615648118	
		**21,97**			**0,0324**	
ARCaP NT2	26,73	21,91	4,96	0,032128557	0,992980843	1
ARCaP NT2	26,69	21,95	4,92	0,033031814	1,020897334	
ARCaP NT2	26,74	22,06	4,97	0,031906629	0,986121823	
		**21,78**				
ARCaP sh2_2	25,63	21,73	3,60	0,082469244	2,548834659	2,616656904
ARCaP sh2_2	25,51	21,91	3,48	0,089622203	2,769907483	
ARCaP sh2_2	25,64	21,69	3,61	0,081899588	2,531228572	
		**21,90**			**0,0278**	
ARCaP NT3	26,88	21,65	5,11	0,028955877	1,042895791	1
ARCaP NT3	26,88	22,04	5,11	0,028955877	1,042895791	
ARCaP NT3	27,07	22,00	5,30	0,025382887	0,914208417	
		**21,98**				
ARCaP sh2_3	26,14	22,08	4,11	0,057911754	2,085791583	2,175869013
ARCaP sh2_3	26,01	21,87	3,98	0,063372467	2,282468591	
ARCaP sh2_3	26,09	21,98	4,06	0,059954007	2,159346864	
		**22,61**			**0,0187**	
PC3 NT1	27,35	22,70	5,58	0,020905118	1,116220205	1
PC3 NT1	27,60	22,58	5,83	0,017579039	0,938625569	
PC3 NT1	27,59	22,55	5,82	0,017701311	0,945154226	
		**22,52**				
PC3 sh2_1	26,12	22,39	4,09	0,058720172	3,135339494	3,179464667
PC3 sh2_1	26,07	22,43	4,04	0,060790934	3,245907003	
PC3 sh2_1	26,11	22,73	4,08	0,059128603	3,157147505	
		**22,29**			**0,0195**	
PC3 NT2	27,58	22,37	5,81	0,017824433	0,914265148	1
PC3 NT2	27,17	22,27	5,40	0,023683071	1,214771132	
PC3 NT2	27,65	22,23	5,88	0,016980232	0,87096372	
		**22,14**				
PC3 sh2_2	26,03	22,15	4,00	0,0625	3,205800237	3,304950924
PC3 sh2_2	26,00	22,16	3,97	0,063813258	3,273160915	
PC3 sh2_2	25,93	22,12	3,90	0,066985841	3,435891621	
		**22,24**			**0,0168**	
PC3 NT3	27,77	22,07	6,00	0,015625	0,929050354	1
PC3 NT3	27,55	22,25	5,78	0,018198962	1,082096151	
PC3 NT3	27,68	22,40	5,91	0,016630784	0,988853495	
		**22,65**				
PC3 sh2_3	26,50	22,65	4,47	0,045122787	2,682965861	2,95667412
PC3 sh2_3	26,26	22,54	4,23	0,053289681	3,168562993	
PC3 sh2_3	26,33	22,76	4,30	0,050765775	3,018493506	

Colour	Legend
yellow	mean Housekeeping
blue	mean Delta Delta CT
lavender	Ct-values
