# Supplementary material for: CHD1 loss negatively influences metastasis-free survival in R0-resected prostate cancer patients and promotes spontaneous metastasis in vivo
Source: Cancer Gene Ther. 2021 Jan 7;29(1):49–61. doi: 10.1038/s41417-020-00288-z (PMC8761572; doi:10.1038/s41417-020-00288-z)
Supplement: Supplementary file 12 — Ct values qPCR PMEPA1a [file 41417_2020_288_MOESM12_ESM.pdf]

PMEPA1a qPCR

	target gene (TG)	housekeeping gene (HG)	TG - HG	ΔCt - control	
Probe	PMEPA1a	SNRPD3	ΔCt	ΔΔCt	2^-(ΔΔCt)
ARCAP M neg 1	24,89086586	22,64	2,250865862	0	1
ARCAP M neg 1	24,8897354	22,69	2,199735401	-0,051130461	1,03607645
ARCAP M neg 1	24,84319289	22,67	2,173192893	-0,077672969	1,055314471
ARCAP M neg 2	24,72938237	22,13	2,599382371	0,348516509	0,785391283
ARCAP M neg 2	24,70257037	22,47	2,232570368	-0,018295493	1,01276222
ARCAP M neg 2	24,69707717	22,2	2,49707717	0,246211308	0,843107613
ARCAP M neg 3	24,9898547	22,68	2,309854705	0,058988843	0,959936685
ARCAP M neg 3	25,07342806	22,68	2,393428062	0,142562201	0,905908848
ARCAP M neg 3	24,83919112	22,61	2,229191119	-0,021674742	1,015137211
ARCAP M CHD1 1	26,67252347	22,23	4,442523469	2,191657607	0,218899777
ARCAP M CHD1 1	26,74628673	22,15	4,596286726	2,345420864	0,196769584
ARCAP M CHD1 1	26,79759161	22,13	4,66759161	2,416725748	0,187280715
ARCAP M CHD1 2	26,26814551	21,93	4,338145513	2,087279651	0,235323997
ARCAP M CHD1 2	26,30896226	22,28	4,02896226	1,778096398	0,29156786
ARCAP M CHD1 2	26,26245038	22,03	4,232450379	1,981584518	0,253211614
ARCAP M CHD1 3	26,81144957	22,62	4,191449569	1,940583708	0,260511017
ARCAP M CHD1 3	27,04048134	22,68	4,360481345	2,109615483	0,231708764
ARCAP M CHD1 3	27,64067082	22,63	5,010670819	2,759804958	0,147644042
PC3 neg 1	31,02084475	22,19	8,830844746	0,495538887	0,709296686
PC3 neg 1	30,73530586	22,4	8,335305859	0	1
PC3 neg 1	30,41798905	22,32	8,097989051	-0,237316808	1,178798237
PC3 neg 2	30,47159603	21,81	8,661596025	0,326290166	0,797584807
PC3 neg 2	30,33258077	22,41	7,922580775	-0,412725084	1,33119792
PC3 neg 2	30,07087858	22,29	7,780878581	-0,554427278	1,468585517
PC3 neg 3	30,66498917	22,31	8,35498917	0,019683311	0,986449218
PC3 neg 3	31,24087563	21,93	9,310875633	0,975569774	0,508538965
PC3 neg 3	30,33148446	22,57	7,761484459	-0,5738214	1,488460978
PC3 CHD1 1	31,69410258	22,77	8,924102579	0,58879672	0,664897233
PC3 CHD1 1	32,25054546	22,67	9,580545461	1,245239602	0,421837834
PC3 CHD1 1	31,79173532	22,71	9,081735323	0,746429464	0,596076968
PC3 CHD1 2	31,82513078	22,41	9,415130783	1,079824924	0,47308623
PC3 CHD1 2	31,82237985	22,53	9,292379852	0,957073993	0,515100558
PC3 CHD1 2	32,6761471	22,65	10,0261471	1,690841246	0,309746257
PC3 CHD1 3	31,70344249	21,84	9,863442488	1,528136629	0,346724905
PC3 CHD1 3	31,69949736	22,01	9,689497359	1,354191501	0,391153966
PC3 CHD1 3	30,8437777	22,21	8,633777703	0,298471844	0,81311322
